# Polyploid evolution: The ultimate way to grasp the nettle

**DOI:** 10.1371/journal.pone.0218389

**Published:** 2019-07-01

**Authors:** Ludmila Rejlová, Jindřich Chrtek, Pavel Trávníček, Magdalena Lučanová, Petr Vít, Tomáš Urfus

**Affiliations:** 1 Institute of Botany, The Czech Academy of Sciences, Průhonice, Czech Republic; 2 Department of Botany, Faculty of Science, Charles University, Prague, Czech Republic; 3 Department of Botany, Faculty of Science University of South Bohemia, České Budějovice, Czech Republic; 4 Faculty of Environmental Sciences, Czech University of Life Sciences Prague, Prague, Czech Republic; University of Delhi, INDIA

## Abstract

Polyploidy is one of the major forces of plant evolution and widespread mixed-ploidy species offer an opportunity to evaluate its significance. We therefore selected the cosmopolitan species *Urtica dioica* (stinging nettle), examined its cytogeography and pattern of absolute genome size, and assessed correlations with bioclimatic and ecogeographic data (latitude, longitude, elevation). We evaluated variation in ploidy level using an extensive dataset of 7012 samples from 1317 populations covering most of the species’ distribution area. The widespread tetraploid cytotype (87%) was strongly prevalent over diploids (13%). A subsequent analysis of absolute genome size proved a uniform Cx-value of core *U. dioica* (except for *U. d*. subsp. *cypria*) whereas other closely related species, namely *U. bianorii*, *U. kioviensis* and *U. simensis*, differed significantly. We detected a positive correlation between relative genome size and longitude and latitude in the complete dataset of European populations and a positive correlation between relative genome size and longitude in a reduced dataset of diploid accessions (the complete dataset of diploids excluding *U. d*. subsp. *kurdistanica*). In addition, our data indicate an affinity of most diploids to natural and near-natural habitats and that the tetraploid cytotype and a small part of diploids (population from the Po river basin in northern Italy) tend to inhabit synanthropic sites. To sum up, the pattern of ploidy variation revealed by our study is in many aspects unique to the stinging nettle, being most likely first of all driven by the greater ecological plasticity and invasiveness of the tetraploid cytotype.

## Introduction

Polyploidy, sometimes referred to as whole-genome multiplication, is generally considered a major force in plant evolution, producing novelties which may eventually lead to single-step speciation, that is, saltation [1–4]. Moreover, the substantial success of angiosperms, the largest clade of land plants, is attributed to polyploidy [5]. Probably 15% (but at least 2–4%) of all speciation events in angiosperms are estimated to have involved polyploidization [6, 7]. Different ploidy levels can either correspond to already discrete lineages or species [8], or constitute intraspecific variation [9]. Newly established polyploid lineages frequently undergo subsequent diploidization [10–12], which is usually followed by genome downsizing [13–15]. Nevertheless, distinct cytotypes frequently coexist in sympatry and, according to the current state of knowledge, at least 16% of all vascular plant species consist of multiple cytotypes [16].

Polyploidy directly affects a number of key biological features (e.g. cell and plant size and duration of mitosis) ultimately associated with distinct physiology and ecology [17, 18]. Such novelties frequently result in improved adaptation potential, fitness, etc. [19–21], which is further mirrored, for example, by broader ecogeographic and climatic niches of polyploids compared to their diploid or lower-ploidy progenitors [22–24]. Specific features of plants have repeatedly been linked to polyploidy (e.g. phenology, mycorrhizal colonization, pollinator behaviour, herbivore predation, salinity tolerance and migration potential [25–30]). Moreover, polyploid cytotypes tend to inhabit a broad range of synanthropic habitats, in contrast to their diploid congeners (*Arabidopsis arenosa* (L.) Lawalrée, *Cardamine amara* L., *Centaurea stoebe* L., *Solidago gigantea* Ait., etc. [31–35]), and their greater ecological plasticity and synanthropic affinity can increase their invasive potential [36, 37].

The essential first step when gaining insight into the evolution of polyploid plants is cytogeography, the study of cytotype diversity and its past and predicted future distribution patterns [13]. Knowledge of the cytotype distribution pattern usually reveals phenomena such as environmental segregation or reproductive isolation of cytotypes [38–40].

Despite the undisputed evolutionary significance of polyploidy, there is a lack of comprehensive cytogeographical studies, with only a few focusing on widespread weedy plants [41] even though they represent highly suitable model taxa for investigating the evolutionary potential of polyploids (e.g. *Mercurialis annua* L., *Tripleurospermum inodorum* (L.) Sch. Bip., *Senecio inaequidens* DC. [42–45]). Surprisingly, the stinging nettle *Urtica dioica* L., one of the most troublesome polyploid weeds, remains considerably understudied, despite being highly important in agriculture, the textile and cosmetics industries [46–48], and medicine [49, 50]. The species represents a nitrophilous, synanthropic and invasive species with a cosmopolitan distribution [51–53]. *Urtica dioica* is characterized by huge variation mirrored by a high number of intraspecific taxa distinguished either solely based on morphological characters (e.g. various types of indumentum [54–56]) or with consideration for sexual morphs (predominantly stochastically occurring [54, 55, 57]). Finally, polyploidy is a truly substantial source of variation in *U. dioica*. Published diploid chromosome counts frequently refer to plants from relict or semi-natural habitats (e.g. alluvial forests [58–60]) whereas tetraploids have been reported to occur in habitats of various types, even highly synanthropic ones [58, 61–63]. However, even though relatively many chromosome counts have been published (e.g. [64]), the distribution pattern has so far only been studied marginally and locally. Moreover, ploidy levels were not directly considered in recent phylogenetic reconstructions [65–68].

We have adopted the only current taxonomic treatment of *U. dioica* consisting of several subspecies. Apart from the nominate tetraploid and widely distributed subspecies *U. d*. subsp. *dioica*, all subspecies are supposed to be diploid and somewhat restricted in their distribution area: *U. d*. subsp. *kurdistanica* (found in alpine habitats of Anatolia and Near Eastern mountain ranges [69, 70]), *U. d*. subsp. *pubescens* (scattered in lowlands from Italy across the Balkan Peninsula to the delta of the river Volga [71–73]), *U. d*. subsp. *sondenii* (tundra marches [59, 73, 74]) and *U. d*. subsp. *subinermis* (alluvial forests, floodplain forests [54, 55, 71, 75]). Finally, the unique steno-endemic *U. dioica* subsp. *cypria* is treated as a subspecies of *U. dioica* (a single population in Cyprus, population UP1219; S2 Table) even though its morphology is distinct [72, 76]. However, the infraspecific phylogeny of *U. dioica* is still largely unresolved [68] and the ranks of its infraspecific taxa also remain a matter of debate [58]. Three recent phylogenies [65, 67, 68] also place four other taxa within the crown clade of *Urtica* (corresponding to *U. dioica*): *U. atrovirens* Req., *U. bianorii* (Knoche) Paiva, *U. kioviensis* Rogow. and *U. simensis* Hochst. ex A. Rich.

The present study aims to assess the ploidy and genome size variation within *U. dioica* across Europe (with contiguous areas of West Asia). We placed particular emphasis on the following questions: (1) What is the general cytogeographic pattern of *U. dioica* in Europe (with contiguous areas of West Asia)? (2) Is genome size a suitable taxonomic marker for resolving current taxonomic ambiguities? (3) Do certain cytotypes occur in particular habitats?

## Materials and methods

### Materials

#### Plant material

**Fig 1:**
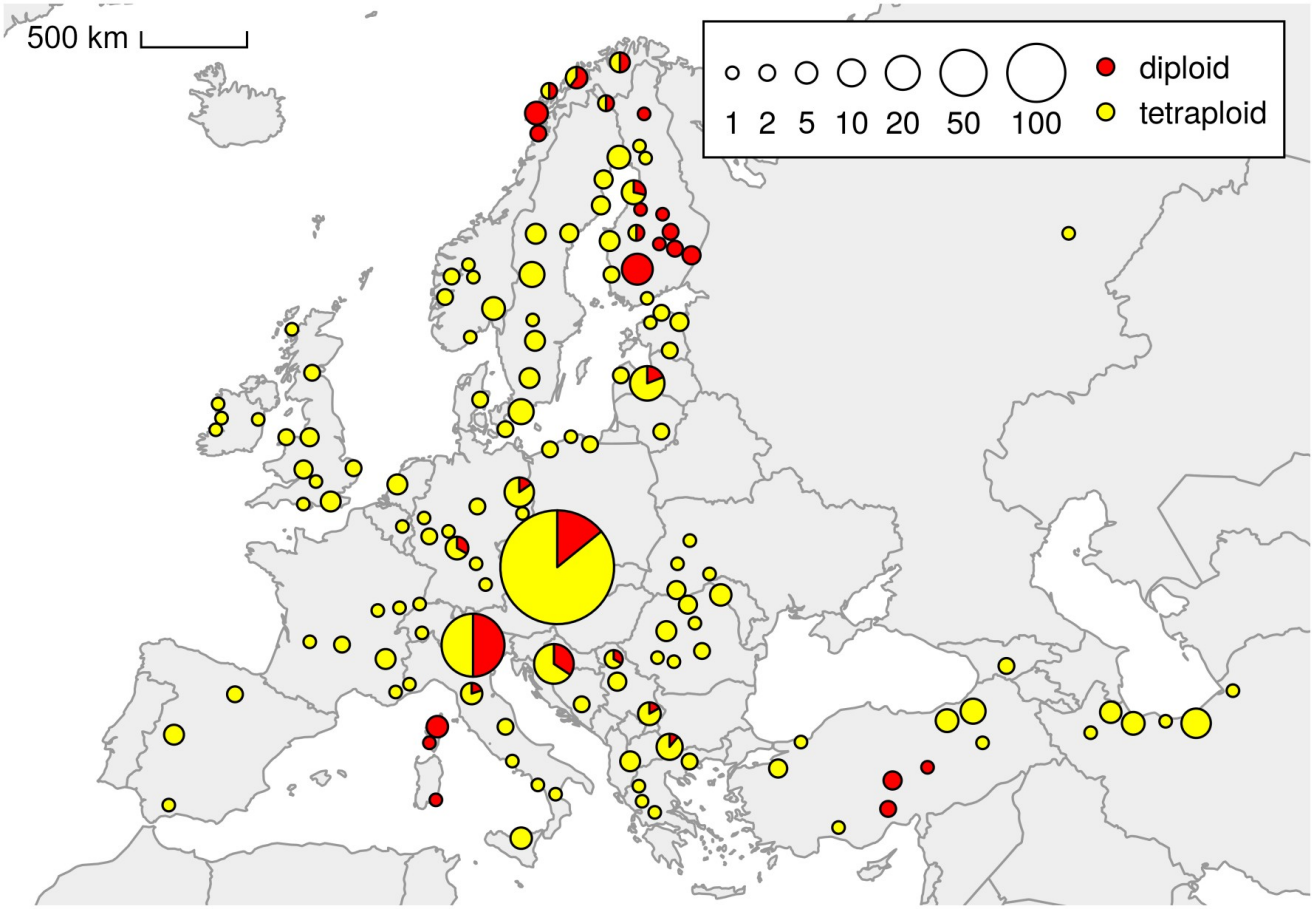
Distribution of two dominant cytotypes of *Urtica dioica* in Europe and West Asia. Map of all samples based on flow cytometric analyses of 1305 populations. The size of the circles reflects the number of populations.

Plants were collected between 2012 and 2018 at 1317 localities (1305 localities of *U. dioica* and 12 localities of closely related species) across Europe and West Asia (Fig 1, S1 Table, S1, S2 and S6 Figs). Although the sampling was primarily random, we focused partially on relict and semi-natural habitats (e.g. ravine and alluvial forests, alpine vegetation and tundra marches, Mediterranean mountains) because (partly allegedly) diploid taxa (*U. d*. subsp. *kurdistanica*, subsp. *pubescens*, subsp. *sondenii*, subsp. *subinermis*) were often reported from such habitats [54, 55, 59, 69–75]. In total, 7012 plants (6977 individuals of *U. dioica* and 35 individuals of closely related species) were sampled (5–10 plants per population; the distance between sampled plants was at least 3 m to avoid re-sampling of the same clone). As a rule, fresh leaves were used for flow cytometric analyses, in some cases silica-gel-dried leaves were used (~10% of samples). A subset of plants was transferred to the experimental garden of the Institute of Botany of the Czech Academy of Sciences in Průhonice (N49.99474, E14.56617, 320 m a.s.l.) for further cultivation and chromosome counting. Voucher specimens will be deposited in the Herbarium of the Charles University, Prague (PRC). GPS coordinates, the elevation and type of habitat were recorded for each population (S1 Table). The study did not necessitate any specific permissions and did not involve endangered or protected species.

### Methods

#### Flow cytometry

Cytotypes were identified by means of flow cytometry, a technique enabling us to analyse large numbers of samples over a short period and to collect appropriate many samples of all taxa and cytotypes [77]. Relative genome size was ascertained for all plants (S1 Table) and absolute genome size was estimated for a subset of samples (Table 1).

**Table 1:** Summary of absolute genome size of *Urtica dioica* and closely related species (2C-values in pg) and detected numbers of (somatic) chromosomes.

Taxon	No. of individuals analysed/No. of populations	Mean Cx-value (pg) ± SD *	2C-value range (pg)	Chrom. number(2n)	Difference compared to 2x (%)	Difference compared to 4x (%)
*U. d*. subsp. *dioica*	32/27	0.55 ± 0.04^*E*^	2.08-2.20	52	–	–
*U. d*. subsp. *dioica* – 3x	1/1	0.54	–	39	–	–
*U. d*. subsp. *kurdistanica*	6/3	0.59 ± 0.01C^*C*^	1.15-1.20	–	–	–
*U. d*. subsp. *pubescens*	16/14	0.58 ± 0.03C^*C*^	1.10-1.21	26	–	–
*U. d*. subsp. *sondenii*	4/2	0.57 ± 0.01^*CD*^	1.12-1.15	26	–	–
*U. d*. subsp. *subinermis*	19/13	0.58 ± 0.03^*C*^	1.10-1.25	26	–	–
**Closely related species**:						
*U. atrovirens*	4/3	0.60 ± 0.004^*C*^	1.18-1.19	–	3.5	45.4
*U. bianorii*	1/1	0.83^*A*^	–	–	43.5	24.3
*U. d*. subsp. *cypria*	6/1	0.55 ± 0.005^*DE*^	1.65-1.67	–	44.4	23.9
*U. kioviensis*	5/4	0.71 ± 0.025^*B*^	1.36-1.43	–	22.6	35.3
*U. simensis*	1/1	0.74^*B*^	–	–	28.7	32.1

* Different letters indicate groups of taxa that are significantly different in Tukey HSD test.

Sample preparation followed a simplified two-step protocol [78]. A part of a petiole was chopped together with the internal reference standard *Bellis perennis* L. (2C = 3.38 pg; [79]) using a sharp razor blade in a plastic Petri dish containing 500 μl of the ice-cold buffer Otto I (0.1-M monohydrate citric acid and 0.5% Tween 20). The suspension was filtered through a 42-μm nylon mesh and the isolated nuclei were stained for 5 minutes with 1 ml of the buffer Otto II (0.4-M Na_2_HPO_4_ ⋅ 12H_2_O) supplemented with the fluorochrome 4’,6-diamidino-2-phenylindole (DAPI; final concentration 4 μg ⋅ ml^−1^) and ß-mercaptoethanol (final concentration 2 μl ⋅ ml^−1^).

Absolute genome size was estimated using the intercalating fluorochrome propidium iodide (PI) supplemented with RNase IIA (both at final concentrations of 50 μg ⋅ ml^−1^). Each sample were analysed three times on three consecutive days to rule out diurnal fluctuation. If the deviation among all particular measurements of the same individual exceeded the threshold of 3%, additional analyses were conducted [78].

To assess heteroploid hybridization, seeds from the mixed population (i.e. population UP0466) were also analysed. Achenes were removed from the pericarp and chopped in the single-phase seeds buffer LBO1 (15M Tris, 2M Na_2_ EDTA, 0.5M spermine tetrahydrochloride, 80M KCl, 20M NaCl, 0.1% Triton X-100, stored at −20°C [80]) together with the fluorochrome 4’,6-diamidino-2-phenylindole (DAPI) and ß-mercaptoethanol.

All samples were incubated for 5–10 minutes at room temperature before being run through each of two flow cytometers (relative genome size: CyFlow ML equipped with a 365-nm UV LED as the light source; absolute genome size: CyFlow SL with a diode-pumped 532-nm solid-state green laser; both Partec GmbH, Münster, Germany). The resulting histograms were evaluated in Partec FloMax 2.3 software (Partec GmbH, Münster, Germany). Only analyses providing peaks with a coefficient of variation of less than 3% for fresh and 5% for silica-dried material were processed further.

One-way analysis of variance (ANOVA), followed by Tukey’s honest significant difference (HDS) test, was used to test the significance of genome size differences between the taxa analysed. Values of genome size were log-transformed before the analysis. All statistical analyses were performed and all plots were produced in the R statistical environment [81].

#### Chromosome counts

Chromosome counts were determined from root tips of germinating seeds and cultivated individuals. Selected samples were processed according to the modified protocol of Mandáková & Lysak [82].

Fresh roots (~1 cm long) were put into 1.5-ml Eppendorf tubes with distilled water and placed into a container with ice-flakes for 24 hours. Afterwards they were put into a freshly prepared fixative (ethanol: acetic acid, 3: 1, v: v) and stored overnight in a refrigerator (~4°C). The material was stored at −20°C in the fixative until further use.

The root tips were washed twice in distilled water (each time for 5 min), then a citrate buffer was applied and roots were washed in an orbital shaker (twice for 5 min). Subsequently, the buffer was sucked out of the sample and a 0.3% mixture of pectolytic enzymes (pectolyase, cellulase, cytohelicase) was added, followed by incubation in an incubator (37°C, 120 min). Then the enzyme mixture was replaced with the same citrate buffer.

The white tip of the root meristem was cut under a stereomicroscope, excess buffer was removed, and the sample was sprinkled with 60% acetic acid with an incubation time of 1–2 min. The root meristem was disintegrated using dissecting needles and the obtained meristematic suspension was covered by a cover-slip. The slide was moved 2–3 times above a flame and then the material was carefully squashed.

The slides were placed into a freezer (~−80°C) and after 10 minutes in the freezer the cover-slips were separated from the slides by razor. The samples were subsequently dyed with 15 *μ*l of Vectashield with 4′,6-diamidino-2-phenylindole (DAPI). The preparations were covered with new cover-slips and fixed with nail polish.

Chromosomes were observed under a Nikon Eclipse E600 microscope equipped with a Nikon DS-Qi1Mc camera, and images were acquired using NIS-Elements AR software.

#### Ecological relations

We used exactly recorded locations of all populations to get a basic grasp of the ecological preferences of major cytotypes (the diploid and tetraploid cytotypes of *U. dioica*). To evaluate the ecological relations of major cytotypes, we applied simple modelling using the Bioclim algorithm according to Chumová et al. [83]. In the first step, georeferenced data were spatially stratified to avoid discrepancies caused by unequal sampling (R package ‘spThin’, Aiello-Lemmens et al. [84]; a 20-km and 5-km threshold distance for tetraploid and diploid population, respectively, was used). The resulting 576 localities were used for the extraction of bioclimatic data. Data from raster layers for all 19 bioclimatic variables were extracted using the ‘extract’ function in the ‘raster’ R package [85]. Principal trends in the variation of bioclimatic variables were detected by PCA. Mutually uncorrelated variables were identified by stepwise forward selection and subjected to linear discriminant analysis. All analyses were conducted using the ‘MorphoTools’ R package for multivariate data handling [86]. In addition, correlations of relative fluorescence intensity value with elevation (dataset divided into two elevation ranges: 0–500 and >500 m above sea level), latitude and longitude were quantified by fitting a linear or quadratic function. To assess the affinity of both cytotypes to human-affected habitats, we adopted a four-level scale of synanthropy (sensu Tüxen 1956 [87]), which was arbitrarily assigned to each sampling locality. Subsequently, Pearson’s chi-square test [88] was used to determine the dependence between the degree of synanthropy and ploidy level.

## Results

### Flow cytometry

**Fig 2:**
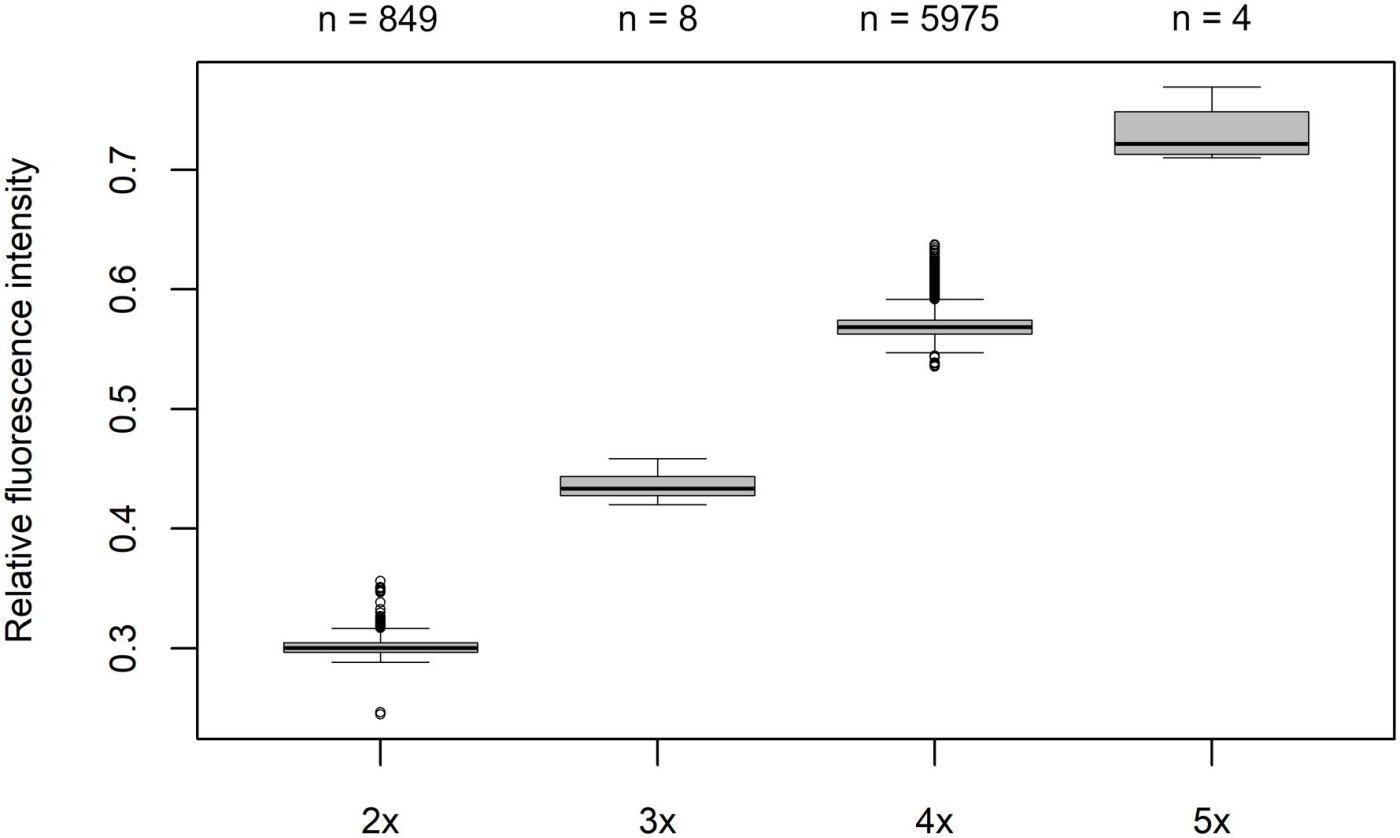
Box-and-whisker plot of relative fluorescence intensity of *Urtica dioica*. The numbers above the boxes indicate the numbers of individuals analysed.

We determined the relative genome size of 6836 plants from 1295 populations (176 individuals were excluded due to poor-quality flow-cytometric histograms). Our results confirm the occurrence of two dominant DNA ploidy levels having the following mean relative fluorescence intensity values (± SD): 2x = 0.30 ± 0.01 (range: 0.24–0.36, 50% variation, n = 849), 4x = 0.57 ± 0.01 (range: 0.54–0.64, 18.5% variation, n = 5975). The average coefficient of variance (CV) was 1.68% (particular CVs are given in S1 Table). The tetraploid cytotype strongly prevailed over the diploid one (2x = 13%, 4x = 87%). Diploids were found frequently in mixed populations with prevailing tetraploids. For the first time we managed to detect a few triploid (8) and pentaploid (4) individuals in a mixed-ploidy population of diploids and tetraploids (3x = 0.44 ± 0.01 (range: 0.42–0.46, 9.5% variation, n = 8), 5x = 0.73 ± 0.02 (range: 0.71–0.77, 8.5% variation, n = 4); Fig 2, S3 and S4 Figs).

To assess the potential for heteroploid hybridization, we analysed 70 achenes from the mixed population (i.e. population UP0466; S1 Table). From diploid maternal plants (33 seeds overall), 82% of the progeny (27 achenes) was diploid (with 3x endosperm) and 18% (6) triploid (4x endosperm). Tetraploid maternal individuals (37 seeds overall) produced 94% (35 achenes) of tetraploid seeds (with 6x endosperm) and one triploid (5x endosperm) and one pentaploid seed (9x endosperm, S5 Fig).

**Fig 3:**
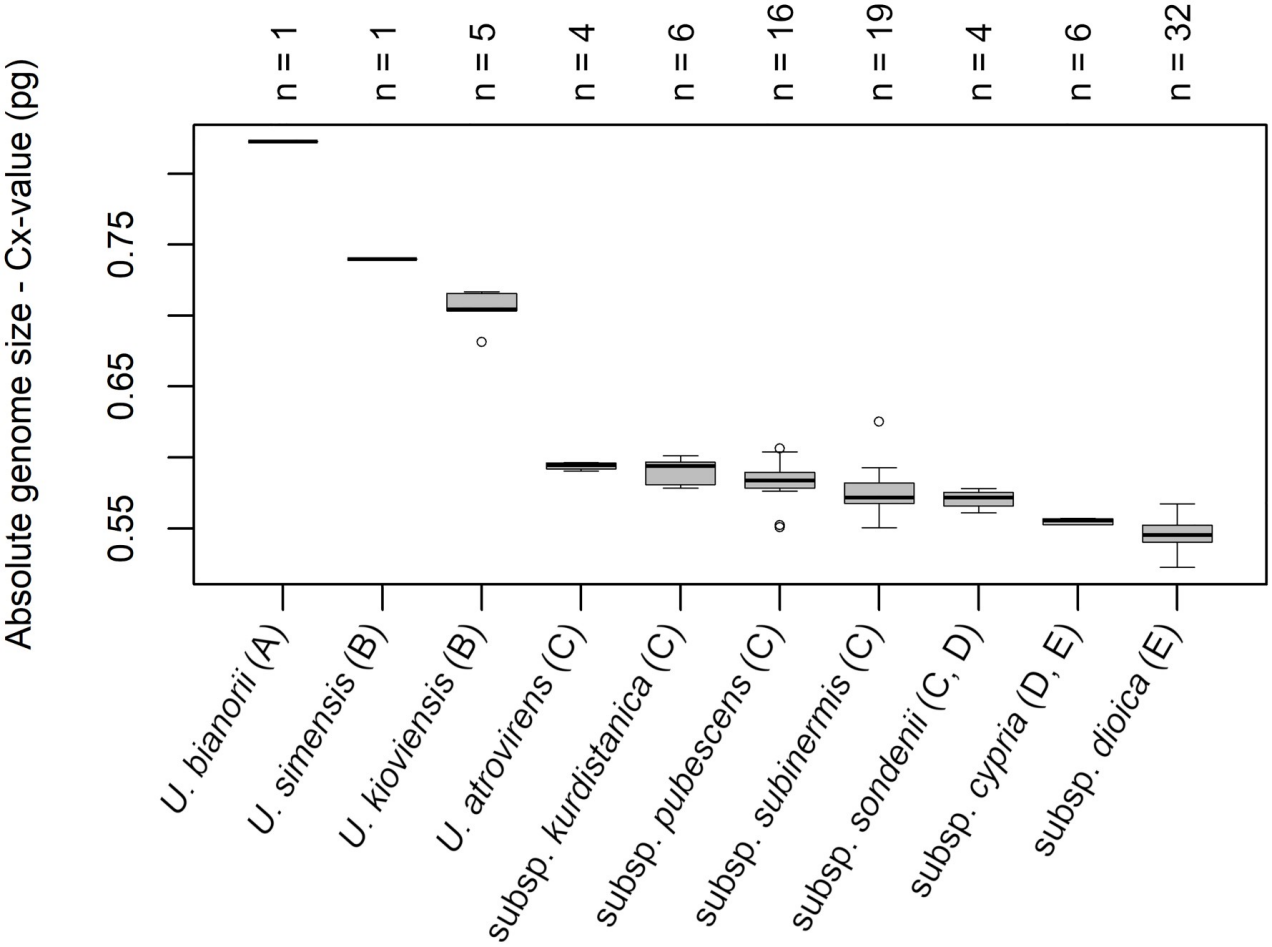
Box-and-whisker plot of absolute genome size of *Urtica dioica* and closely related species. Absolute genome size of the 4x cytotype (*U. d*. subsp. *dioica*), the 2x cytotype (*U. d*. subsp. *kurdistanica*, subsp. *pubescens*, subsp. *sondenii*, subsp. *subinermis*) and closely related species (*U. atrovirens*, *U. bianorii*, *U. d*. subsp. *cypria*, *U. kioviensis* and *U. simensis*). The letters A–E show the grouping based on a one-way analysis of variance (ANOVA) followed by Tukey’s test (HDS). The numbers above the boxes indicate the numbers of individuals analysed.

To calibrate the measurements and detect differences between particular diploid subspecies, we also estimated absolute genome size for a reduced set of accessions (78 plants from 60 populations—*U. dioica*; 17 plants from 10 populations—closely related species; S2 Table, S6 Fig). Core diploid subspecies (*U. d*. subsp. *kurdistanica*, subsp. *pubescens*, subsp. *sondenii* and subsp. *subinermis*) did not differ from each other in absolute genome size whereas the other closely related species (i.e. species closely related to *U. dioica* crown clade in recent phylogenies) *U. bianorii*, *U. d*. subsp. *cypria*, *U. kioviensis* and *U. simensis* had significantly greater DNA content. Only *U. atrovirens*, which is also ranked among the closely related species of *U. dioica*, was assigned to the group of core diploid subspecies. We, for the first time, estimated the absolute genome size of *U. d*. subsp. *kurdistanica*, *U. d*. subsp. *cypria* and the triploid cytotype of *U. dioica* (a plant morphologically identical with *U. d*. subsp. *dioica*). Absolute genome size was determined for all the mentioned species and subjected to ANOVA and Tukey’s HDS test (p <0.001; Fig 3, Table 1, S7 Fig).

### Chromosome counts

**Fig 4:**
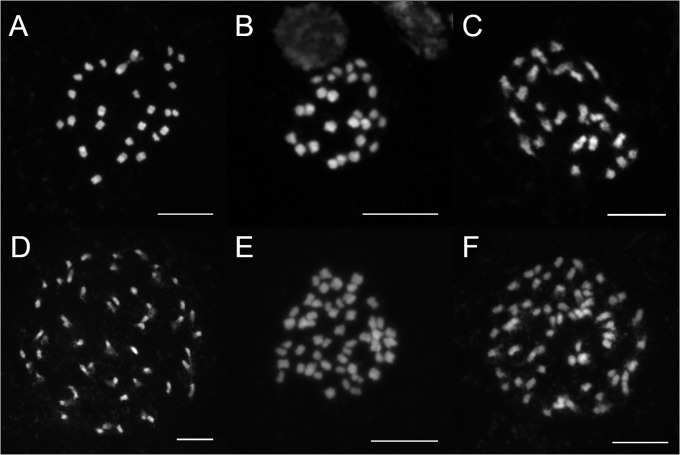
Microphotographs of somatic metaphases of *Urtica dioica*. (A) *U. d*. subsp. *pubescens* (population UP0009, Italy) − 2n = 2x = 26; (B) *U. d*. subsp. *sondenii* (population UP0584, Finland) − 2n = 2x = 26; (C) *U. d*. subsp. *subinermis* (population UP0059, Czech Republic) − 2n = 2x = 26; (D) *U. d*. subsp. *dioica* (population UP0033, France) − 2n = 4x = 52; (E) *U. d*. subsp. *dioica* (population UP0718, Iran) − 2n = 4x = 52; (F) Pentaploid cytotype of *U. dioica* (population UP0770, Czech Republic) − 2n = 5x = 65 (morphologically identical with *U. d*. subsp. *dioica*).

All DNA ploidy levels were verified by subsequent chromosome counts. Ten plants were checked for their chromosome numbers using fluorescent karyology. The chromosome number of 2n = 26 was ascertained for diploids (three plants classified as *U. d*. subsp. *pubescens*, subsp. *sondenii* and subsp. *subinermis*, respectively), 2n = 39 was ascertained for triploids (one plant morphologically identical with *U. d*. subsp. *dioica*), 2n = 52 was ascertained for tetraploids (five plants assigned to *U. d*. subsp. *dioica*) and 2n = 65 was ascertained for pentaploids (one plant also morphologically identical with *U. d*. subsp. *dioica*; Fig 4, Table 1).

### Ecological relations

#### Bioclimatic and geographic pattern

**Fig 5:**
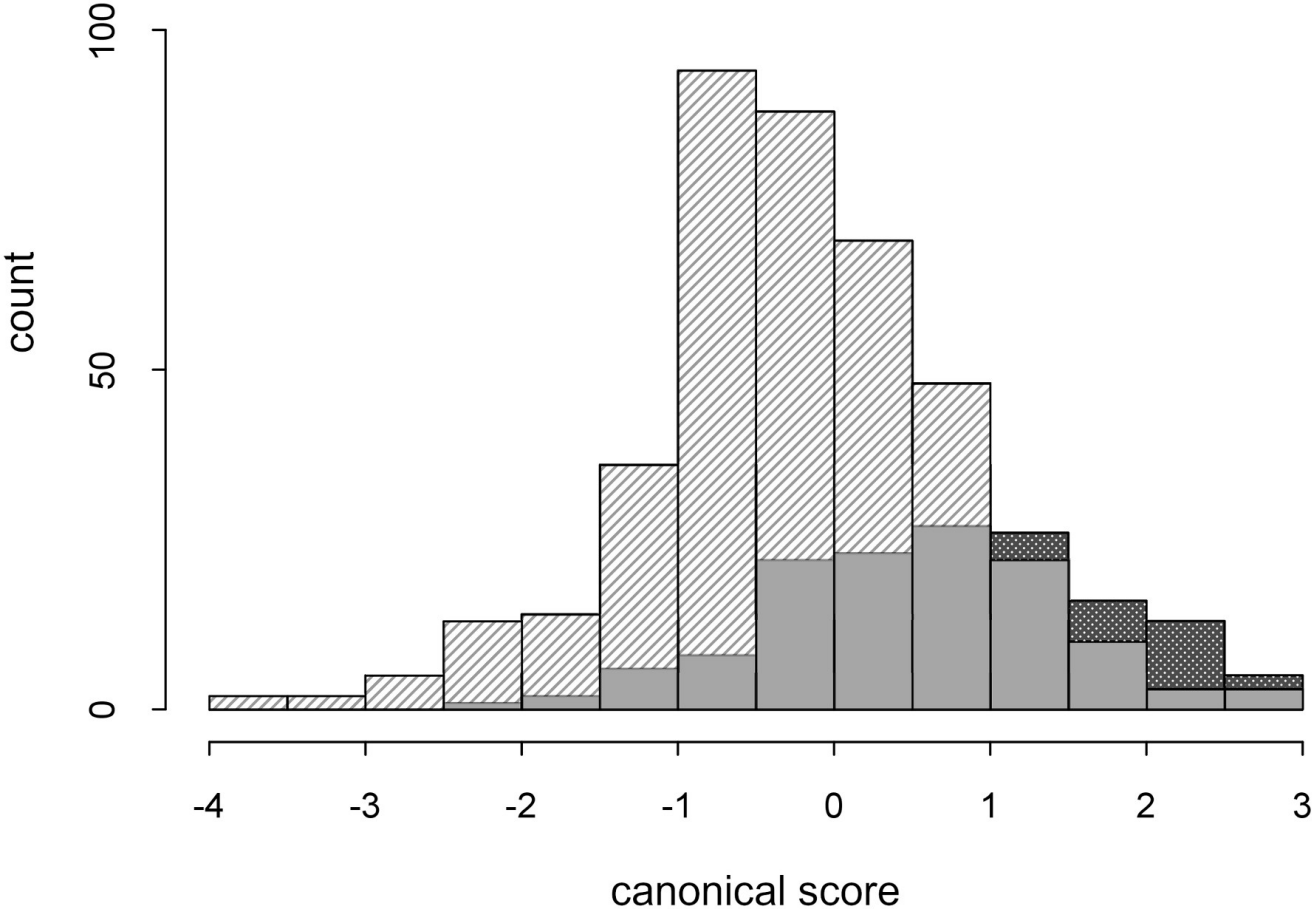
Discriminant analysis of two groups (DA)—Stepwise forward selection using basic modelling (Bioclim algorithm). Bioclimatic data related to the ploidy levels (diploid and tetraploid) of individual populations—stratified data. The features contributing the most to the variability were BIO3, 5 and 17; striped area—tetraploid cytotype, dotted area—diploid cytotype, grey area—overlapping of both cytotypes.

To verify the habitat and ecological preferences of individual ploidy levels, we used basic modelling. Our analysis of bioclimatic data in relation to the ploidy levels of individual populations and their exactly recorded positions shows that the variability of individual ploidy levels is interdependent. This is also confirmed by field observations, as in most cases diploid individuals grow in mixed populations with the tetraploid cytotype, which evinces a high degree of plasticity (e.g. ecological or morphological) shared with diploid individuals. In the stepwise selection analysis, the following features were the most contributing to group separation: BIO3 (Isothermality = BIO2/BIO7 * 100; this quantifies how much day-to-night temperatures oscillate relative to the summer-to- winter (annual) oscillations), BIO5 (Max Temperature of Warmest Month) and BIO17 (Precipitation of Driest Quarter; Fig 5).

**Fig 6:**
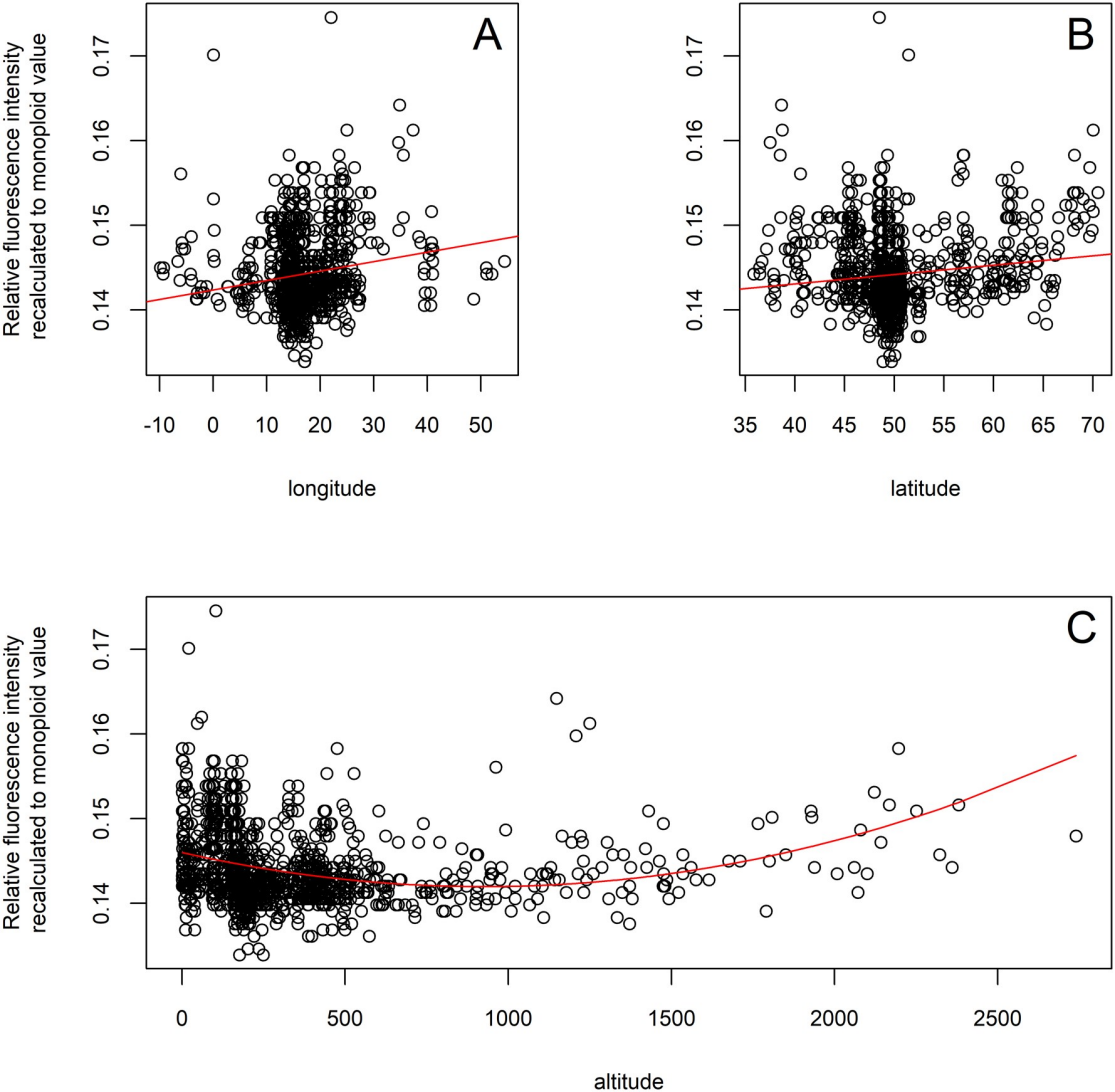
Relations between relative fluorescence intensity and longitude, latitude and elevation fitted with a linear or quadratic function. (A) Correlation of relative fluorescence intensity with longitude fitted with a linear function (complete dataset of European populations); (B) Correlation of relative fluorescence intensity with latitude fitted with a linear function (complete dataset of European populations); (C) Correlation of relative fluorescence intensity with elevation fitted with a quadratic function.

We found a positive correlation between relative fluorescence intensity and longitude (cor = 0.161, p <0.001); Fig 6A) and latitude (cor = 0.133, p <0.001; Fig 6B) in the complete dataset of European populations. In the reduced dataset of diploid accessions (the complete dataset of diploids excluding *U. d*. subsp. *kurdistanica*), we detected a positive correlation between relative fluorescence intensity and longitude (cor = 0.296, p <0.001). Diploid taxa growing in relict habitats preferred lower elevations compared to the ubiquitous tetraploid cytotype. Correlations of relative fluorescence intensity with elevation were significant for each of the datasets fitted with a linear function (0–500 m above sea level: cor = −0.305, p <0.001; >500: cor = 0.344, p <0.001) and in all data fitted with a quadratic function (cor = 0.208, p <0.001; Fig 6C).

#### Affinity to synanthropic habitats

**Fig 7:**
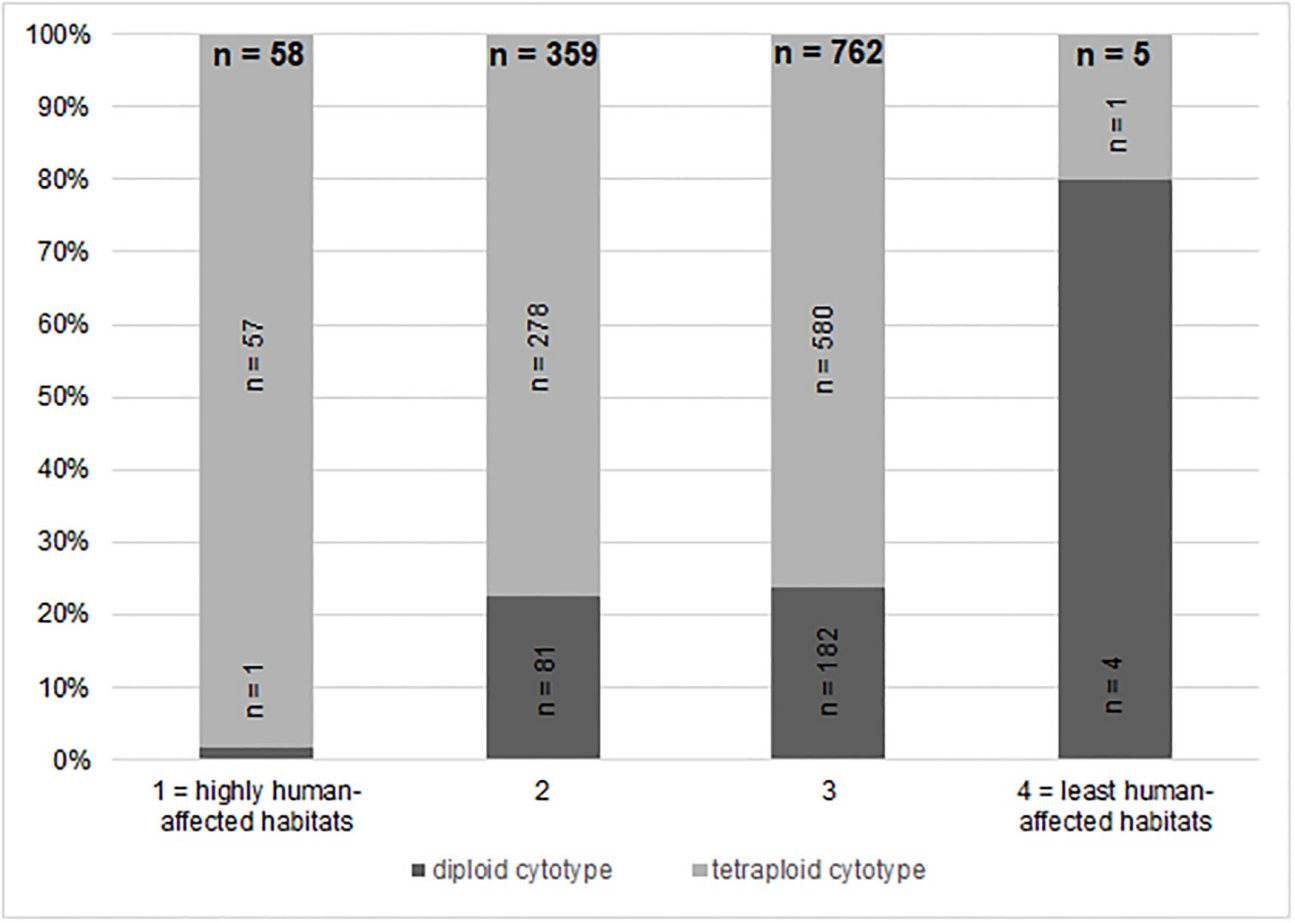
Ratio of individuals in habitats of different types—Four-level scale of synanthropy. Relative proportions of the two major cytotypes (diploid and tetraploid) captured in different types of human-affected habitats. For definitions and examples see Table 3. The numbers in columns indicate the number of populations depending on habitat type, corresponding to Table 2.

To determine habitat preferences, especially of relict diploids, we used the data from assessment of individual locations. Using Pearson’s chi-squared test, we have determined with a high degree of confidence that the probability of occurrence of a diploid population depends on the type of environment (p <0.001). The distribution of diploid and tetraploid populations with respect to the environment is presented as in a contingency table (Table 2, Fig 7), along with associated standard residuals. The diploid cytotype of *U. dioica* (*U. d*. subsp. *kurdistanica*, subsp. *sondenii* and subsp. *subinermis*) tends to occur in less human-affected habitats (habitat type 3 and 4 on the four-level scale of synanthropy; Table 3). A special case is the diploid subspecies *U. d*. subsp. *pubescens* from the Po river basin, which occurs exclusively in highly synanthropic and strongly human-affected locations (habitat type 1 and 2 on the four-level scale of synanthropy; mode value for all diploid subspecies of *U. dioica*: 3; mode value for *U. d*. subsp. *pubescens*: 2). This stands in contrast to the tetraploid cytotype, which occurs in habitats of all types, although it prefers environments with an increased degree of synanthropy.

**Table 2:** Contingency table with standard residuals of diploid and tetraploid populations depending on habitat type.

Ploidy level	Environment type 1	Environment type 2	Environment type 3	Environment type 4
Diploids (2x)	1 (-3.90)	81 (-0.04)	182 (1.38)	4 (3.07)
Tetraploids (4x)	57 (3.90)	278 (0.04)	580 (-1.38)	1 (-3.07)

**Table 3:** Evaluation of the affinity of particular *Urtica dioica* cytotypes to human-affected habitats.

Level	Vegetation types and habitats	Degree of influence by man
**1**	intensively managed habitats (agricultural, ruderal, etc.), road margins, urbanized areas	highly nitrophilous and intensively human-affected locations
**2**	extensively cultivated landscapes, agricultural marginal habitats, cultivated and plantation-like forests	partly synanthropic and extensively cultivated locations
**3**	semi-natural vegetation, recent vegetation +/- corresponding to the potential natural vegetation *	semi-natural habitats
**4**	tundra marches, Mediterranean alpine zones, natural alluvial associations, other relict habitats	least human-affected habitats (~primary habitats)

* sensu Tüxen 1956 [87]

## Discussion

During our large-scale screening of *Urtica dioica* across Europe and West Asia, we found two major ploidy levels: widely distributed tetraploids and less frequent diploids. We have not proved any strong correlation supporting either the generally suggested hypothesis that polyploids are more abundant at higher elevations and latitudes or the idea that diploids are confined to Southern European glacial refugia whereas polyploids occur across broader geographic ranges [89], often shifted to harsh environments. Instead, our results suggest that diploid plants show some degree of affinity to habitats less affected by human activities, in contrast to tetraploids, which tend to grow in human-made or strongly influenced habitats. Furthermore, our study has revealed a significant difference in absolute genome size between *U. dioica* and its closely related species *U. bianorii*, *U. dioica* subsp. *cypria*, *U. kioviensis* and *U. simensis* [72, 76, 90–95].

We are aware that the frequency of diploids detected would be significantly lower had we chosen an entirely random sampling strategy instead of partly preferentially targeting relict and natural habitats. Additional occurrences of the alluvial diploid cytotype (~*U. d*. subsp. *subinermis*) can be expected in Western Europe (especially in France and the United Kingdom). On the other hand, we sampled numerous relict habitats in Spain and northern Iran, and detected only tetraploids there, so the occurrence of a diploid cytotype is, in concordance with previously published chromosome counts [94, 96, 97], less probable in these two countries.

### Major and minor cytotypes of *Urtica dioica*

The widely distributed tetraploids and the less frequent diploids possess the chromosome numbers of 2n = 2x = 26 and 2n = 4x = 52, respectively, ascertained here and also reported previously [61–63]. However, for the first time we managed to capture a small percentage of very rare triploid (8 individuals) and pentaploid (4 individuals) cytotypes, both in mixed-ploidy populations of diploids and tetraploids. The origin of these minor cytotypes is discussed below.

For some plants, we detected abnormal values of relative genome size (8 tetraploid individuals, range of 2C pg: 1.66–1.74), which could be explained by aneuploidy. The values might correspond to a loss of four chromosomes, i.e. to the frequently reported chromosome number 2n = 48 [61–64, 98–102]. Unfortunately, we did not succeed in cultivating any of these aberrant individuals, so we cannot confirm any hypothetical aneuploid counts.

Data from our screening of seeds from both diploid and tetraploid maternal plants from a mixed-ploidy field population in southern Moravia (south-eastern Czech Republic—UP0466; S1 Table) suggest that gene flow between the two cytotypes can occur. Besides the ploidy level of the embryo, we also paid special attention to the ploidy of the endosperm in order to decipher the contribution of the paternal cytotype and thus to determine the seed formation pathway [80, 103]. The greater frequency of triploid embryos in seeds of diploid maternal plants might be in line with the greater frequency of tetraploid plants and thus the larger greater amount and pressure of diploid pollen grains (from tetraploid plants). Another explanation, not mutually exclusive with the previous, supposes that the spatial pattern of (often large) male and female clones of both cytotypes at the site may play a role. Although our data indicate the origin of triploid seeds via heteroploid crosses, we cannot fully exclude the ability of diploid plants to produce triploid seeds via unreduced gametes (i.e. reduced gamete fusion with a male unreduced gamete from diploid plants), in general, the formation of unreduced gametes is not frequent [104, 105]. The frequency of triploid seeds (18% from diploid and 3% from tetraploid maternal plants) also contradicts the frequency of adult triploid plants in mixed diploid-tetraploid populations (8 individuals). The most plausible explanation seems to be a triploid block (lower fitness of or strong selection against triploid seedlings, or lower germination rates of triploid seeds, or their inability to germinate, compared to diploid and tetraploid ones [106]). The detection of a pentaploid individual (with 9x endosperm) in the offspring of a tetraploid plant indicates the formation of an unreduced gamete at the 4x level and its fusion with a reduced (x) gamete from a diploid plant. Alternatively, pentaploids might originate from crosses between tetraploids and hexaploids, but we detected neither adult hexaploid plants nor pentaploids with the embryo: endosperm ploidy ratio indicating this hybridization history (5x embryo: 7x endosperm). A combination of a more extensive seed screen (incl. experimental hybridization) and molecular analyses should be carried out to assess the rate of gene flow. Nevertheless, we have confirmed the possibility of heteroploid hybridization, which might cause genetic erosion and therefore pose a threat to the far less abundant diploid populations.

### Diploids as indicators of natural habitats versus synathrophic invasive tetraploids?

We detected geographically stratified elevational and ecological segregation. In Central Europe, the Balkans and the Baltic region, diploids are likely confined to lowland alluvial, especially white willow, gallery forests. In addition, river banks and the surroundings of water bodies, together with forest-tundra stands and ravine forests, are the predominating habitats of diploids in Northern Europe. By contrast, diploids in Anatolia tend to occupy natural habitats at higher elevations (e.g. screes). The species assembly of ancient Central European semi-natural alluvial forests was formed in the Early Holocene. Since the Neolithic period, the floodplains of lowland rivers experienced vast changes caused by erosion, soil deposition and eutrophication. The human-driven decline of woodlands, especially in the Medieval period, and changes in species composition led to the fragmentation of semi-natural woodlands, which are currently confined to more or less small patches within agricultural landscapes [107, 108]. The diploid cytotype of *U. dioica* is restricted to well preserved alluvial forests in Central and Western Europe, so diploids may also indicate relict habitats of this type. The rather narrow ecological niche of diploids compared to tetraploids might indicate, besides other phenomena, ploidy-related drought tolerance and greater plasticity in polyploids allowing tetraploid to occupy a broader spectrum of habitats [109–111]. Similar ecological diploid-polyploid differentiation has been described in the grass species *Deschampsia cespitosa* (L.) P. Beauv. (tussock grass) in Britain [112] and *Dactylis glomerata* L. (cock’s-foot) in Spanish Galicia [22]. Diploids appeared to be restricted mainly to low-density forest-floor habitats in woodlands of mostly ancient, semi-natural origin whereas tetraploids were found in varied habitats, but they predominated in open places such as in meadows, pastures, plantations, their verges and waste grounds. Based on our observations, both cytotypes are ecologically differentiated, but tetraploids do not exhibit local adaptation. Instead, they have greater fitness across both diploid- and polyploid-occupied regions.

In contrast to diploids in Central Europe, Anatolian diploids occur at higher elevations compared to tetraploids and also in different habitats. Instead of European lowland and ravine forest they mostly occur in mountain screes. Analogously to Europe, however, the vast majority of Anatolian populations (from various habitats, including screes) are formed by tetraploids. Surprisingly, even in Anatolia a single diploid was found in an alluvial population (Cappadocia—population UP0038; S1 Table).

In general, however, we have not confirmed the frequently made assumption that polyploids are more abundant at higher elevations and latitudes because of their potentially greater ecological tolerance and colonization ability [1, 17, 113–116]. However, considering only the invasiveness of polyploids, our results are well in agreement with general suggestions. The widespread tetraploid cytotype of *U. dioica* is also often supposed to be an allopolyploid or a group of allopolyploids with different evolutionary histories (e.g. [117]). Polyploidization and hybridization likely went hand in hand, resulting in rapid divergence of the neopolyploid. Tetraploids were possibly predisposed to spread into ranges thanks to their potential for subsequent adaptation due to greater genetic diversity, higher survival rates and better fitness ascribed to the heterosis effect, restoring sexual reproduction following hybridization [37]. A more or less stable occurrence of diploids in semi-natural habitats and tetraploids in mainly human-made habitats, together with a recent spread of tetraploids, has also been reported for *Centaurea stoebe* [32, 33, 118] and *Seseli libanotis* (L.) W.D.J.Koch [119]. Although a positive correlation between invasiveness and ploidy seems to be in conflict with a negative correlation between invasiveness and genome size [120], it is their interaction that underlies their actual effects on plant phenotypes and physiology, and, ultimately, on invasion success [36].

### Taxonomic consequences

In the two most recent phylogenies [67, 68], the crown clade of *Urtica* (predominantly formed by *U. dioica*) consist of different additional related taxa, depending on the molecular markers used. Based on a concatenated tree (combining nuclear and plastid markers [68]), *U. dioica* in the strict sense, an exclusively Eurasian group including *U. dioica* (except for subsp. *cypria*), *U. kioviensis* from western Eurasia and *U. platyphylla* Wedd. from Northeastern Eurasia is a sister group to Mediterranean endemics (*U. atrovirens*, *U. bianorii*) and two African species (*U. massaica* Mildbr., *U. simensis*). Together these taxa form a well-supported cluster. Our genome size data partly support this concept. *Urtica bianorii* and *U. d*. subsp. *cypria* clearly fall outside of the *U. dioca* s.str. group in published phylogenies, which is in concordance with our genome size data. *Urtica kioviensis*, which could not be separated from *U. dioica* s.str. in previous phylogenies [68], could be reliably separated from the Eurasian *U. dioica* s.str. clade using genotyping-by-sequencing data [121], and this separation is well supported and justified by our genome size estimations. Only *U. atrovirens*, which is ranked close to *U. dioica* s.str., did not significantly differ from diploid subspecies of *U. dioica* even though it is distinctive morphologically [93, 94]. We have thus confirmed that genome size can significantly contribute to the delineation and detection of taxa, and that differences between genome size values may be indicative of genetic distance (see e.g. [103, 122, 123]).

In addition to other already discussed reasons to recognize several intraspecific taxa of *U. dioica* at the subspecies level (i.e. extreme morphologic forms and sexual morphs), polyploidy evidently shapes the structured pattern confining diploid cytotypes to relict habitats (e.g. alluvial forests, tundra marches or Mediterranean alpine zones). The diploid subspecies (subsp. *kurdistanica*, subsp. *pubescens*, subsp. *sondenii* and subsp. *subinermis*) are more or less morphologically, ecologically and geographically defined and capture a considerable part of the morphological diversity present in Western Eurasian *Urtica dioica*. However, any clear delineation of some of them is anything but straightforward and even molecular approaches have failed to resolve infraspecific relationships [67, 68]. Although published chromosome counts/ploidy levels are very scarce, ploidy is widely accepted as a trait in the delineation of *Urtica dioica* subsp. *dioica* (tetraploid) and the rest of the subspecies [56]. Here we generally confirm that the diploid level (with the chromosome number of 2n = 26) is associated with plants morphologically assigned to *U. d*. subsp. *subinermis*, *U. d*. subsp. *sondenii*, *U. d*. subsp. *pubescens* and *U. d*. subsp. *kurdistanica*. We did not find any significant differences in genome size between the subspecies, so genome size cannot serve as a supportive character in the delineation of homoploid taxa as in some another plant groups [123–126].

One particular matter for debate is the delimitation and geographic distribution of *U. d*. subsp. *pubescens*. Geltman [127, 128] regards it as an endemic of wetland territories in the Volga delta and its surroundings and in the lower Dnieper region whereas in its wide circumscription the species occupies a geographic area spanning Southern and Eastern Europe, western Turkey [72], Georgia and Azerbaijan [121]. According to Weigend [71] it can be identified by its green-grey leaf colour, a distinctly hairy stem and leaves on both sides, and based on the ratio of the width to the length of the lamina. However, minor morphological differences between populations (unpublished data) and, in addition, genetic differences between European and West Asian populations [121] may indicate a mosaic-like structure and different evolutionary histories within subsp. *pubescens* in its broad circumscription. We carried out an extensive screening of ploidy levels in populations of ‘hairy’ nettles from the Po river basin (northern Italy), tentatively assigned to *U. d*. subsp. *pubescens*. Across the basin and in adjacent mountain valleys, we found mostly diploid plants, even though this area is surrounded by expanses dominated by tetraploids (even from the south, i.e. on the slopes of the Apennines). Worth mentioning are two aspects: First, this is the only large area in our study that is most likely occupied nearly exclusively by diploid plants (S1 Fig); otherwise, diploids occur as a rule in mixed-ploidy populations, accompanied by tetraploids. Second, the Po river diploids regularly occur both in a wide range of highly synanthropic types of habitats and in semi-natural alluvial vegetation. Diploid populations might have survived the last glaciation in an refugium extending along the lower elevations of the southern Alps and in adjoining areas, as demonstrated for many alpine plants as well as for beech (*Fagus*) and some insect species [129–132]. Alternatively, diploids might have survived in more southerly located refuges in the Apennine Peninsula [133, 134]. In any case, the Po river diploids definitely deserve a further biosystematic/taxonomic evaluation.

The genome size of *U. d*. subsp. *kurdistanica* corresponds to 2n = 26 (diploid level)—the same as in the other diploid subspecies. However, chromosome counts are not available for this subspecies, so certain deviations from this number cannot be fully excluded. Nevertheless, ours is the first DNA ploidy level estimation for this subspecies. Both localities visited over the course of our study (Mt. Erciyes Dâgi (Argaeus) in Cappadocia the Gusguta valley in the Bolkar Dağlari Mts. in southern Anatolia) are also mentioned by Weigend [72], which confirms the taxonomical identity of the plants under study. They occur on high-mountain screes that are only marginally influenced by human activities (pastures) and therefore fall within the broad concept that diploids tend to inhabit natural or semi-natural habitats. Long-term survival of these diploid populations seems to be a plausible explanation at least for two reasons: In Anatolia there was no major Pleistocene ice-sheet similar to those covering the European Alps or Scandinavia and only mountain peaks exceeding the height of ca 2200 m were glaciated [135, 136]. Furthermore, higher elevations provided moist conditions contrasting with the drier climate that prevailed in lower elevations of Anatolia during glacial periods [137].

We did not find diploids among a total of 80 plants from Iran. Weigend [72] reported two subspecies of *U. dioica* from this country, namely subsp. *dioica* and subsp. *kurdistanica*. Our plants can be more or less identified as subsp. *dioica*, and their ploidy is thus in line with the general picture of diploid subsp. *kurdistanica* and tetraploid subsp. *dioica*. Still, several populations (north of Tehran—Mt. Damavand and its surroundings) formed a unique monoecious population and their inflorescences consisted of equal numbers of male and female flowers (male in the upper part of the inflorescence), which does not correspond to the morphological description of either subsp. *dioica* or subsp. *kurdistanica*.

Finally, despite our extensive screening, we failed to find diploid plants at several localities of the morphologically defined taxon *U. d*. subsp. *subinermis*. This subspecies therefore has to be considered only supposedly diploid, as no chromosome counts are presented in the respective papers. This applies, for example, to the Neusiedler See lake (northeastern Austria), where precisely defined localities of plants morphologically assigned to *U. d*. subsp. *subinermis* are mentioned by Geltman [127] and Weigend [71]. Tetraploid plants found over the course of our study (5975 plants sampled) morphologically resemble *U. d*. subsp. *subinermis*, which raises the question as to whether (auto)polyploidization has taken place in this subspecies, which would make the pattern of genomic evolution within the diploid-tetraploid complex of *Urtica dioica* considerably more complicated.

## Conclusion

Our large-scale cytogeographic screening of *Urtica dioica* has revealed a complex pattern across a major part of the species’ distribution range, consisting of a widespread tetraploid cytotype, low-abundant scattered diploids and sporadically occurring triploid and pentaploid plants. We have not found any differences in genome size (Cx-values) between most subspecies of *U. dioica* (*U. d*. subsp. *dioica*, subsp. *kurdistanica*, subsp. *pubescens*, subsp. *sondenii* and subsp. *subinermis*). On the other hand, *U. d*. subsp. *cypria* does differ in genome size from the rest of *U. dioica*. Moreover, Cx-values of closely related species (*U. bianorii*, *U. kioviensis* and *U. simensis*) clearly differ from those of *U. dioica*, and genome size can thus serve as a valuable supportive character in the delimitation of *U. dioica*. We have also found positive correlations between genome size and longitude and latitude in our complete dataset of European populations and a positive correlation of genome size with longitude in a reduced dataset of diploid accessions (the complete dataset of diploids excluding *U. d*. subsp. *kurdistanica*). Diploid taxa growing in relict habitats are more frequent at lower elevations. In addition, our study has revealed a significant affinity of diploids to less human-influenced semi-natural habitats (this does not apply diploids from the Po river basin, assigned to *U. d*. subsp. *pubescens*) and (in the European range) to lower elevations. The tetraploid cytotype, by contrast, tends to thrive even in highly synanthropic sites and is able to expand to higher elevations.

## Supporting Information

S1 Table List of analyses of *Urtica dioica* (sorted by population identification number). For each population, the following information is provided: geographic coordinates in the WGS-84 system, elevation, country abbreviation, collector’s initials, number of analysed plants in simultaneous analyses, relative fluorescence intensity, DNA-ploidy level, and coefficient of variance of the standard and sample peaks.
(PDF)

S2 Table List of analyses (absolute genome size) of *Urtica dioica* (sorted by taxon and population identification number). For each population, the following information is provide: geographic coordinates in the WGS-84 system, elevation, country abbreviation, collector’s initials, absolute genome size—2C-value (pg), ploidy level, and coefficient of variance of standard and sample peaks.
(PDF)

S1 Fig Map of locations of *Urtica dioica* samples collected in the Po river basin (northern Italy). The size of the circles reflects the number of populations. The blue line indicates the outline of the Po river basin.
(TIF)

S2 Fig Map of locations of *Urtica dioica* samples collected in the Czech Republic and Slovakia. The size of the circles reflects the number of populations.
(TIF)

S3 Fig Relative fluorescence intensity variation in *Urtica dioica*. Two dominant ploidy levels were detected (red—2x and yellow—4x).
(TIF)

S4 Fig Flow cytometric histogram of all detected cytotypes of *Urtica dioica*. Simultaneous analysis—from the left: 2x—diploid cytotype, 3x—triploid, 4x—tetraploid, 5x—pentaploid, *Bellis perennis*—the internal standard.
(TIF)

S5 Fig Proportions of cytotypes of *Urtica dioica* seeds. (A) Ratio of diploid and triploid seeds from a 2x maternal plant (from the mixed-ploidy population); (B) Ratio of triploid, tetraploid and pentaploid seeds from a 4x maternal plant (from a mixed-ploidy population).
(TIF)

S6 Fig Map of locations of closely related species. Species closely related to the *U. dioica* clade in recent phylogenies, namely: *U. atrovirens*, *U. bianorii*, *U. kioviensis*. The top-left section shows the one population of *U. simensis* in Ethiopia. The size of the circles reflects the number of populations. For more details see S2 Table.
(TIF)

S7 Fig Absolute genome size variation in *Urtica dioica* and closely related species. Diploid cytotype—*U. d*. subsp. *kurdistanica*, subsp. *pubescens*, subsp. *sondenii* and subsp. *subinermis*; tetraploid cytotype—*U. d*. subsp. *dioica*; closely related species—*U. atrovirens*, *U. bianorii*, *U. d*. subsp. *cypria*, *U. kioviensis* and *U. simensis*). Numbers of analysed individuals are presented in parentheses.
(TIF)
