# Supplementary material for: Polyploid evolution: The ultimate way to grasp the nettle
Source: PLoS One. 2019 Jul 1;14(7):e0218389. doi: 10.1371/journal.pone.0218389 (PMC6602185; doi:10.1371/journal.pone.0218389)
Supplement: S1 Table — For each population, the following information is provided: geographic coordinates in the WGS-84 system, elevation, country abbreviation, collector’s initials, number of analysed plants in simultaneous analyses, relative fluorescence intensity, DNA-ploidy level, and coefficient of variance of the standard and sample peaks. (PDF) [file pone.0218389.s001.pdf]

Table S1. List of analysis of *Urtica dioica* (sorted by identification number of population). For each population the information is provided about geography coordinates in WGS-84 system, altitude and abbreviation of country, collectors initials, number of analysed plant in simultaneous analysis, relative fluroscence intesity, DNA-ploidy level, coefficient of variance of standard and samples.

ID number of population	ID number of analysis	Taxon	Collector	Relative fluorescence intensi	DNA-ploidy level	CV of standard	CV of sample	N. of individuals in analysis	Latitude (WGS-84)	Longitude (WGS-84)	Altitude (m a.s.l.)	Country (ISO 3166-1)
UP0001	U0001	subsp. *dioica*	PT, TU	0.562	4	1.3	1.9	4	N48.55522	E11.58817	468	DEU
	U0002	subsp. *dioica*	PT, TU	0.562	4	1.3	1.9	4	N48.55522	E11.58817	468	DEU
UP0002	U0003	subsp. *dioica*	PT, TU	0.560	4	1.2	2.8	5	N47.83019	E11.90803	706	DEU
	U0004	subsp. *dioica*	PT, TU	0.560	4	1.2	2.8	5	N47.83019	E11.90803	706	DEU
UP0003	U0005	subsp. *dioica*	PT, TU	0.561	4	1.3	2.4	1	N47.45217	E11.86506	556	AUT
	U0006	subsp. *dioica*	PT, TU	0.569	4	1.2	1.9	1	N47.45217	E11.86506	556	AUT
	U0007	subsp. *dioica*	PT, TU	0.561	4	1.0	1.6	1	N47.45217	E11.86506	556	AUT
	U3305	subsp. *dioica*	PT, TU	0.573	4	1.1	1.6	1	N47.45217	E11.86506	556	AUT
	U3306	subsp. *dioica*	PT, TU	0.559	4	1.3	1.8	1	N47.45217	E11.86506	556	AUT
UP0004	U0008	subsp. *dioica*	PT, TU	0.557	4	1.3	1.8	3	N47.00861	E11.50800	1790	AUT
	U0009	subsp. *dioica*	PT, TU	0.557	4	1.3	1.8	3	N47.00861	E11.50800	1790	AUT
UP0005	U0010	subsp. *pubescens*	PT, TU	0.299	2	1.2	1.7	2	N46.57342	E11.52164	444	ITA
	U0011	subsp. *pubescens*	PT, TU	0.299	2	1.2	1.7	2	N46.57342	E11.52164	444	ITA
	U0012	subsp. *pubescens*	PT, TU	0.333	2	1.2	2.8	1	N46.57342	E11.52164	444	ITA
UP0006	U0013	subsp. *pubescens*	PT, TU	0.300	2	1.5	2.5	2	N46.56411	E11.51614	433	ITA
	U0014	subsp. *pubescens*	PT, TU	0.300	2	1.5	2.5	2	N46.56411	E11.51614	433	ITA
	U0015	subsp. *pubescens*	PT, TU	0.301	2	1.2	2.2	3	N46.56411	E11.51614	433	ITA
	U0016	subsp. *pubescens*	PT, TU	0.294	2	1.3	2.6	4	N46.56411	E11.51614	433	ITA
	U0017	subsp. *pubescens*	PT, TU	0.301	2	1.2	2.2	5	N46.56411	E11.51614	433	ITA

ID number of population	ID number of analysis	Taxon	Collector	Relative fluorescence intensi	DNA-ploidy level	CV of standard	CV of sample	N. of individuals in analysis	Latitude (WGS-84)	Longitude (WGS-84)	Altitude (m a.s.l.)	Country (ISO 3166-1)
	U0018	subsp. *pubescens*	PT, TU	0.296	2	1.4	2.1	6	N46.56411	E11.51614	433	ITA
UP0007	U0019	subsp. *pubescens*	PT, TU	0.296	2	1.3	2.0	4	N46.37231	E11.27297	230	ITA
	U0020	subsp. *pubescens*	PT, TU	0.296	2	1.3	2.0	4	N46.37231	E11.27297	230	ITA
	U0021	subsp. *pubescens*	PT, TU	0.296	2	1.3	2.0	4	N46.37231	E11.27297	230	ITA
	U0022	subsp. *pubescens*	PT, TU	0.296	2	1.3	2.0	4	N46.37231	E11.27297	230	ITA
UP0008	U0023	subsp. *pubescens*	PT, TU	0.307	2	1.2	2.3	1	N46.34064	E11.28606	235	ITA
	U0024	subsp. *pubescens*	PT, TU	0.299	2	1.4	2.3	1	N46.34064	E11.28606	235	ITA
	U0025	subsp. *pubescens*	PT, TU	0.303	2	1.3	2.3	1	N46.34064	E11.28606	235	ITA
	U0026	subsp. *pubescens*	PT, TU	0.296	2	1.1	1.8	1	N46.34064	E11.28606	235	ITA
	U0027	subsp. *pubescens*	PT, TU	0.295	2	0.9	2.7	1	N46.34064	E11.28606	235	ITA
	U0028	subsp. *pubescens*	PT, TU	0.298	2	1.5	2.0	1	N46.34064	E11.28606	235	ITA
UP0009	U0029	subsp. *pubescens*	PT, TU	0.298	2	1.2	2.2	1	N45.69819	E10.92406	124	ITA
	U0030	subsp. *pubescens*	PT, TU	0.297	2	1.5	1.6	1	N45.69819	E10.92406	124	ITA
	U0031	subsp. *pubescens*	PT, TU	0.299	2	1.0	1.9	1	N45.69819	E10.92406	124	ITA
	U0032	subsp. *pubescens*	PT, TU	0.299	2	1.5	2.7	1	N45.69819	E10.92406	124	ITA
UP0010	U0033	subsp. *dioica*	PT, TU	0.573	4	1.3	1.7	1	N45.64967	E10.93133	872	ITA
	U0034	subsp. *dioica*	PT, TU	0.566	4	1.2	1.9	1	N45.64967	E10.93133	872	ITA
	U0035	subsp. *dioica*	PT, TU	0.562	4	1.2	1.9	1	N45.64967	E10.93133	872	ITA
	U0036	subsp. *dioica*	PT, TU	0.570	4	1.6	1.8	1	N45.64967	E10.93133	872	ITA
	U0037	subsp. *dioica*	PT, TU	0.561	4	1.4	1.9	1	N45.64967	E10.93133	872	ITA
UP0011	U0038	subsp. *pubescens*	PT, TU	0.301	2	1.4	2.5	1	N45.28419	E11.54436	14	ITA
	U0039	subsp. *pubescens*	PT, TU	0.303	2	1.1	2.4	1	N45.28419	E11.54436	14	ITA
	U0040	subsp. *pubescens*	PT, TU	0.293	2	1.2	2.2	1	N45.28419	E11.54436	14	ITA

ID number of population	ID number of analysis	Taxon	Collector	Relative fluorescence intensi[illegible]	DNA-ploidy level	CV of standard	CV of sample	N. of individuals in analysis	Latitude (WGS-84)	Longitude (WGS-84)	Altitude (m a.s.l.)	Country (ISO 3166-1)
UP0012	U0041	subsp. *pubescens*	PT, TU	0.292	2	1.1	2.0	1	N45.29367	E11.62086	206	ITA
	U0042	subsp. *pubescens*	PT, TU	0.297	2	1.1	2.4	1	N45.29367	E11.62086	206	ITA
	U0043	subsp. *pubescens*	PT, TU	0.298	2	1.0	2.0	1	N45.29367	E11.62086	206	ITA
	U0044	subsp. *pubescens*	PT, TU	0.294	2	1.5	2.8	1	N45.29367	E11.62086	206	ITA
	U0045	subsp. *pubescens*	PT, TU	0.294	2	1.6	2.2	1	N45.29367	E11.62086	206	ITA
UP0013	U0046	subsp. *pubescens*	PT, TU	0.296	2	1.5	2.4	1	N44.91133	E11.59153	8	ITA
	U0047	subsp. *pubescens*	PT, TU	0.294	2	1.7	2.2	1	N44.91133	E11.59153	8	ITA
	U0048	subsp. *pubescens*	PT, TU	0.293	2	1.2	2.1	1	N44.91133	E11.59153	8	ITA
	U0049	subsp. *pubescens*	PT, TU	0.296	2	1.1	2.2	1	N44.91133	E11.59153	8	ITA
	U0050	subsp. *pubescens*	PT, TU	0.297	2	1.7	2.1	1	N44.91133	E11.59153	8	ITA
UP0014	U0051	subsp. *pubescens*	PT, TU	0.301	2	1.3	1.7	1	N44.76683	E11.85939	1	ITA
	U0052	subsp. *pubescens*	PT, TU	0.301	2	1.6	2.4	1	N44.76683	E11.85939	1	ITA
UP0015	U0053	subsp. *pubescens*	PT, TU	0.299	2	1.2	2.0	1	N44.60553	E12.09756	1	ITA
	U0054	subsp. *pubescens*	PT, TU	0.303	2	1.5	2.3	1	N44.60553	E12.09756	1	ITA
	U0055	subsp. *pubescens*	PT, TU	0.301	2	1.2	2.2	1	N44.60553	E12.09756	1	ITA
UP0016	U0056	subsp. *dioica*	PT, TU	0.588	4	1.3	1.8	1	N44.54508	E12.06253	2	ITA
	U0057	subsp. *dioica*	PT, TU	0.573	4	1.3	2.4	1	N44.54508	E12.06253	2	ITA
	U0058	subsp. *dioica*	PT, TU	0.579	4	0.9	1.6	1	N44.54508	E12.06253	2	ITA
UP0017	U0059	subsp. *pubescens*	PT, TU	0.295	2	1.5	2.2	1	N44.24497	E11.19314	346	ITA
	U0060	subsp. *dioica*	PT, TU	0.577	4	1.2	1.7	1	N44.24497	E11.19314	346	ITA
	U0061	subsp. *dioica*	PT, TU	0.577	4	1.4	1.4	1	N44.24497	E11.19314	346	ITA
UP0018	U0062	subsp. *dioica*	PT, TU	0.578	4	1.4	2.0	1	N44.00511	E11.00950	831	ITA
	U0063	subsp. *dioica*	PT, TU	0.563	4	1.5	1.7	1	N44.00511	E11.00950	831	ITA

ID number of population	ID number of analysis	Taxon	Collector	Relative fluorescence intensi	DNA-ploidy level	CV of standard	CV of sample	N. of individuals in analysis	Latitude (WGS-84)	Longitude (WGS-84)	Altitude (m a.s.l.)	Country (ISO 3166-1)
UP0019	U0064	subsp. *pubescens*	PT, TU	0.292	2	1.3	2.6	1	N45.04953	E10.84967	20	ITA
	U0065	subsp. *pubescens*	PT, TU	0.295	2	1.3	2.2	1	N45.04953	E10.84967	20	ITA
	U0066	subsp. *pubescens*	PT, TU	0.293	2	1.3	2.0	1	N45.04953	E10.84967	20	ITA
	U0067	subsp. *pubescens*	PT, TU	0.292	2	1.2	2.1	1	N45.04953	E10.84967	20	ITA
	U0068	subsp. *pubescens*	PT, TU	0.295	2	1.1	2.1	1	N45.04953	E10.84967	20	ITA
UP0020	U0069	subsp. *pubescens*	PT, TU	0.301	2	1.3	2.0	1	N45.54856	E10.81933	101	ITA
	U0070	subsp. *pubescens*	PT, TU	0.305	2	1.5	2.6	1	N45.54856	E10.81933	101	ITA
	U0071	subsp. *pubescens*	PT, TU	0.300	2	1.2	2.0	1	N45.54856	E10.81933	101	ITA
	U0072	subsp. *pubescens*	PT, TU	0.298	2	1.4	2.0	1	N45.54856	E10.81933	101	ITA
	U3304	subsp. *pubescens*	PT, TU	0.301	2	1.3	2.7	1	N45.54856	E10.81933	101	ITA
UP0021	U0073	subsp. *dioica*	PT, TU	0.556	4	1.4	1.4	5	N45.91701	E15.98376	886	HRV
UP0022	U0074	subsp. *dioica*	PT, TU	0.566	4	1.2	1.9	1	N47.06294	E11.47494	1349	AUT
	U0075	subsp. *dioica*	PT, TU	0.566	4	1.4	2.2	1	N47.06294	E11.47494	1349	AUT
	U0076	subsp. *dioica*	PT, TU	0.566	4	1.2	2.2	1	N47.06294	E11.47494	1349	AUT
UP0023	U0094	subsp. *dioica*	PT, TU	0.567	4	1.1	1.4	5	N45.90171	E15.94975	992	HRV
UP0024	U0095	subsp. *subinermis*	PT, TU	0.296	2	1.3	1.8	2	N44.88181	E15.89882	216	BIH
UP0025	U0096	subsp. *dioica*	PT, TU	0.566	4	1.3	1.6	4	N46.37277	E15.99590	198	SVN
UP0026	U0097	subsp. *dioica*	PT, TU	0.568	4	1.4	2.0	1	N48.12678	E16.60739	157	AUT
	U0098	subsp. *dioica*	PT, TU	0.572	4	1.7	2.8	1	N48.12678	E16.60739	157	AUT
	U0099	subsp. *dioica*	PT, TU	0.562	4	1.2	1.8	1	N48.12678	E16.60739	157	AUT
	U3197	subsp. *dioica*	PT, TU	0.568	4	1.3	1.9	1	N48.12678	E16.60739	157	AUT
UP0027	U0100	subsp. *dioica*	PT, TU	0.584	4	1.5	2.5	1	N47.86714	E16.83800	119	AUT
	U0101	subsp. *dioica*	PT, TU	0.561	4	1.5	1.8	1	N47.86714	E16.83800	119	AUT

ID number of population	ID number of analysis	Taxon	Collector	Relative fluorescence intensi	DNA-ploidy level	CV of standard	CV of sample	N. of individuals in analysis	Latitude (WGS-84)	Longitude (WGS-84)	Altitude (m a.s.l.)	Country (ISO 3166-1)
	U0102	subsp. *dioica*	PT, TU	0.565	4	1.1	1.6	1	N47.86714	E16.83800	119	AUT
UP0028	U0103	subsp. *dioica*	PT, TU	0.560	4	1.9	1.8	1	N47.92331	E16.72031	115	AUT
	U0104	subsp. *dioica*	PT, TU	0.575	4	1.2	1.6	1	N47.92331	E16.72031	115	AUT
	U0105	subsp. *dioica*	PT, TU	0.565	4	1.3	1.8	1	N47.92331	E16.72031	115	AUT
	U0106	subsp. *dioica*	PT, TU	0.568	4	0.9	1.8	1	N47.92331	E16.72031	115	AUT
	U0107	subsp. *dioica*	PT, TU	0.563	4	1.2	1.7	1	N47.92331	E16.72031	115	AUT
UP0029	U0108	subsp. *dioica*	PT, TU	0.568	4	1.2	1.5	1	N48.33775	E16.06386	177	AUT
	U0109	subsp. *dioica*	PT, TU	0.571	4	1.2	1.6	1	N48.33775	E16.06386	177	AUT
	U0110	subsp. *dioica*	PT, TU	0.557	4	1.3	1.7	1	N48.33775	E16.06386	177	AUT
	U3199	subsp. *dioica*	PT, TU	0.564	4	1.2	1.7	1	N48.33775	E16.06386	177	AUT
UP0030	U0111	subsp. *dioica*	PT, ZC	0.571	4	1.1	1.8	1	N45.58577	E2.79005	973	FRA
	U0112	subsp. *dioica*	PT, ZC	0.571	4	1.1	1.8	1	N45.58577	E2.79005	973	FRA
	U0113	subsp. *dioica*	PT, ZC	0.571	4	1.1	1.8	1	N45.58577	E2.79005	973	FRA
UP0031	U0114	subsp. *dioica*	PT, ZC	0.561	4	1.3	1.9	1	N43.71287	E6.50527	901	FRA
	U0115	subsp. *dioica*	PT, ZC	0.561	4	1.3	1.9	1	N43.71287	E6.50527	901	FRA
	U0116	subsp. *dioica*	PT, ZC	0.561	4	1.3	1.9	1	N43.71287	E6.50527	901	FRA
UP0032	U0117	subsp. *dioica*	PT, ZC	0.569	4	1.5	2.3	1	N44.99695	E5.60290	1079	FRA
	U0118	subsp. *dioica*	PT, ZC	0.569	4	1.5	2.3	1	N44.99695	E5.60290	1079	FRA
	U0119	subsp. *dioica*	PT, ZC	0.569	4	1.5	2.3	1	N44.99695	E5.60290	1079	FRA
UP0033	U0120	subsp. *dioica*	PT, ZC	0.571	4	1.7	1.7	1	N46.36237	E6.51936	422	FRA
	U0121	subsp. *dioica*	PT, ZC	0.571	4	1.7	1.7	1	N46.36237	E6.51936	422	FRA
	U1262	subsp. *dioica*	PT, ZC	0.571	4	1.7	1.7	1	N46.36237	E6.51936	422	FRA
UP0034	U0122	subsp. *dioica*	PT, ZC	0.566	4	1.7	2.6	1	N46.59936	E6.31844	1615	CHE

ID number of population	ID number of analysis	Taxon	Collector	Relative fluorescence intensi	DNA-ploidy level	CV of standard	CV of sample	N. of individuals in analysis	Latitude (WGS-84)	Longitude (WGS-84)	Altitude (m a.s.l.)	Country (ISO 3166-1)
	U0123	subsp. *dioica*	PT, ZC	0.566	4	1.7	2.6	1	N46.59936	E6.31844	1615	CHE
	U0124	subsp. *dioica*	PT, ZC	0.579	4	1.8	3.3	1	N46.59936	E6.31844	1615	CHE
UP0035	U0125	subsp. *dioica*	PT, ZC	0.595	4	1.8	2.1	1	N46.52395	E6.58139	370	CHE
	U0126	subsp. *dioica*	PT, ZC	0.595	4	1.8	2.1	1	N46.52395	E6.58139	370	CHE
	U0127	subsp. *dioica*	PT, ZC	0.577	4	1.6	3.2	1	N46.52395	E6.58139	370	CHE
UP0036	U0135	subsp. *dioica*	TU	0.592	4	1.5	2.2	1	N39.82700	E16.31808	279	ITA
	U0517	subsp. *dioica*	TU	0.613	4	1.3	2.6	1	N39.82700	E16.31808	279	ITA
	U0532	subsp. *dioica*	TU	0.598	4	1.1	2.3	1	N39.82700	E16.31808	279	ITA
	U0533	subsp. *dioica*	TU	0.597	4	1.4	2.5	1	N39.82700	E16.31808	279	ITA
	U0534	subsp. *dioica*	TU	0.607	4	1.2	2.4	1	N39.82700	E16.31808	279	ITA
	U0535	subsp. *dioica*	TU	0.598	4	1.6	2.7	1	N39.82700	E16.31808	279	ITA
	U0536	subsp. *dioica*	TU	0.615	4	1.4	2.5	1	N39.82700	E16.31808	279	ITA
UP0037	U0139	subsp. *dioica*	TU	0.588	4	1.3	1.7	1	N37.13425	E31.80033	1227	TUR
	U0521	subsp. *dioica*	TU	0.587	4	0.8	1.6	1	N37.13425	E31.80033	1227	TUR
	U0522	subsp. *dioica*	TU	0.588	4	1.3	1.7	1	N37.13425	E31.80033	1227	TUR
	U0523	subsp. *dioica*	TU	0.597	4	0.9	1.8	1	N37.13425	E31.80033	1227	TUR
	U0547	subsp. *dioica*	TU	0.588	4	1.3	1.7	1	N37.13425	E31.80033	1227	TUR
	U0548	subsp. *dioica*	TU	0.588	4	1.3	1.7	1	N37.13425	E31.80033	1227	TUR
	U0549	subsp. *dioica*	TU	0.588	4	1.3	1.7	1	N37.13425	E31.80033	1227	TUR
	U3535	subsp. *dioica*	TU	0.588	4	1.3	1.7	1	N37.13425	E31.80033	1227	TUR
UP0038	U0140	subsp. *subinermis*	TU	0.329	2	1.7	3.1	1	N38.63836	E34.82100	1148	TUR
	U0524	subsp. *subinermis*	TU	0.324	2	1.0	3.2	1	N38.63836	E34.82100	1148	TUR
	U0525	subsp. *subinermis*	TU	0.356	2	2.6	4.5	1	N38.63836	E34.82100	1148	TUR

ID number of population	ID number of analysis	Taxon	Collector	Relative fluorescence intensi	DNA-ploidy level	CV of standard	CV of sample	N. of individuals in analysis	Latitude (WGS-84)	Longitude (WGS-84)	Altitude (m a.s.l.)	Country (ISO 3166-1)
	U0526	subsp. *subinermis*	TU	0.322	2	0.9	2.9	1	N38.63836	E34.82100	1148	TUR
	U0527	subsp. *subinermis*	TU	0.319	2	0.9	2.9	1	N38.63836	E34.82100	1148	TUR
	U0528	subsp. *subinermis*	TU	0.318	2	1.2	3.5	1	N38.63836	E34.82100	1148	TUR
	U0530	subsp. *subinermis*	TU	0.338	2	1.2	3.7	1	N38.63836	E34.82100	1148	TUR
	U0550	subsp. *subinermis*	TU	0.333	2	0.8	3.6	1	N38.63836	E34.82100	1148	TUR
	U3536	subsp. *subinermis*	TU	0.323	2	1.0	2.6	1	N38.63836	E34.82100	1148	TUR
UP0039	U0142	subsp. *dioica*	TU	0.616	4	1.4	3.0	1	N40.11336	E29.07897	1430	TUR
	U0531	subsp. *dioica*	TU	0.594	4	1.0	2.0	1	N40.11336	E29.07897	1430	TUR
	U0562	subsp. *dioica*	TU	0.596	4	1.3	2.4	1	N40.11336	E29.07897	1430	TUR
	U0564	subsp. *dioica*	TU	0.604	4	0.9	3.3	1	N40.11336	E29.07897	1430	TUR
UP0040	U0143	subsp. *dioica*	TU	0.571	4	1.4	2.9	1	N41.03778	E30.68261	46	TUR
	U0143	subsp. *dioica*	TU	0.571	4	1.1	2.5	1	N41.03778	E30.68261	46	TUR
	U0144	subsp. *dioica*	TU	0.607	4	0.9	2.4	1	N41.03778	E30.68261	46	TUR
	U0144	subsp. *dioica*	TU	0.609	4	1.4	3.3	1	N41.03778	E30.68261	46	TUR
	U0145	subsp. *dioica*	TU	0.591	4	1.8	2.6	1	N41.03778	E30.68261	46	TUR
	U0145	subsp. *dioica*	TU	0.594	4	1.3	1.9	1	N41.03778	E30.68261	46	TUR
	U0565	subsp. *dioica*	TU	0.591	4	1.2	2.4	1	N41.03778	E30.68261	46	TUR
	U0566	subsp. *dioica*	TU	0.598	4	1.6	2.2	1	N41.03778	E30.68261	46	TUR
	U0567	subsp. *dioica*	TU	0.589	4	1.5	3.6	1	N41.03778	E30.68261	46	TUR
UP0041	U0146	subsp. *dioica*	PT	0.584	4	0.9	1.5	1	N50.13757	E15.99272	291	CZE
	U0147	subsp. *dioica*	PT	0.597	4	1.2	2.9	1	N50.13757	E15.99272	291	CZE
UP0042	U0148	subsp. *subinermis*	LR	0.305	2	0.8	3.0	1	N48.68281	E16.93925	153	CZE
UP0043	U0149	subsp. *dioica*	LR	0.573	4	1.1	1.5	1	N48.68231	E16.94206	160	CZE

ID number of population	ID number of analysis	Taxon	Collector	Relative fluorescence intensi	DNA-ploidy level	CV of standard	CV of sample	N. of individuals in analysis	Latitude (WGS-84)	Longitude (WGS-84)	Altitude (m a.s.l.)	Country (ISO 3166-1)
UP0044	U0150	subsp. *subinermis*	LR	0.303	2	1.8	1.9	1	N48.68253	E16.94192	157	CZE
UP0045	U0151	subsp. *subinermis*	LR	0.303	2	1.1	2.0	1	N48.66864	E16.93900	154	CZE
	U0152	subsp. *subinermis*	LR	0.306	2	1.3	2.3	1	N48.66864	E16.93900	154	CZE
UP0046	U0153	subsp. *dioica*	LR	0.577	4	1.6	2.1	1	N48.68192	E16.94289	166	CZE
UP0047	U0154	subsp. *dioica*	LR	0.578	4	0.9	4.7	1	N48.66022	E16.96514	177	CZE
	U0155	subsp. *dioica*	LR	0.579	4	0.9	4.5	1	N48.66022	E16.96514	177	CZE
	U3375	subsp. *dioica*	LR	0.565	4	1.1	1.3	1	N48.66022	E16.96514	177	CZE
UP0048	U0156	subsp. *dioica*	LR	0.577	4	0.9	1.5	1	N48.61758	E16.94042	155	CZE
	U0157	subsp. *dioica*	LR	0.564	4	1.6	2.4	1	N48.61758	E16.94042	155	CZE
UP0049	U0158	subsp. *subinermis*	LR	0.301	2	0.9	1.8	1	N48.61847	E16.94081	155	CZE
	U0159	subsp. *subinermis*	LR	0.310	2	1.3	3.4	1	N48.61847	E16.94081	155	CZE
UP0050	U0160	subsp. *subinermis*	LR	0.297	2	1.2	2.6	1	N48.61850	E16.94103	156	CZE
	U0161	subsp. *subinermis*	LR	0.304	2	1.3	2.3	1	N48.61850	E16.94103	156	CZE
UP0051	U0162	subsp. *subinermis*	LR	0.303	2	1.2	2.2	1	N48.61906	E16.93828	151	CZE
	U0163	subsp. *subinermis*	LR	0.301	2	1.0	1.7	1	N48.61906	E16.93828	151	CZE
UP0052	U0164	subsp. *subinermis*	LR	0.299	2	1.8	2.8	1	N48.61964	E16.93839	156	CZE
	U0165	subsp. *subinermis*	LR	0.308	2	1.5	3.5	1	N48.61964	E16.93839	156	CZE
UP0053	U0166	subsp. *subinermis*	LR	0.302	2	0.8	1.9	1	N48.62306	E16.93511	152	CZE
	U0167	subsp. *subinermis*	LR	0.290	2	1.7	2.7	1	N48.62306	E16.93511	152	CZE
UP0054	U0168	subsp. *dioica*	LR	0.569	4	1.3	1.6	1	N48.62547	E16.93483	152	CZE
UP0055	U0169	subsp. *subinermis*	LR	0.309	2	1.2	2.6	1	N48.63050	E16.95122	150	CZE
UP0056	U0170	subsp. *subinermis*	LR	0.300	2	1.7	2.7	1	N48.63069	E16.95033	150	CZE
	U1336	subsp. *dioica*	LR	0.570	4	1.7	2.3	1	N48.63069	E16.95033	150	CZE

ID number of population	ID number of analysis	Taxon	Collector	Relative fluorescence intensi	DNA-ploidy level	CV of standard	CV of sample	N. of individuals in analysis	Latitude (WGS-84)	Longitude (WGS-84)	Altitude (m a.s.l.)	Country (ISO 3166-1)
	U1337	subsp. *dioica*	LR	0.594	4	1.3	2.6	1	N48.63069	E16.95033	150	CZE
UP0057	U0171	subsp. *dioica*	LR	0.581	4	1.2	1.3	1	N48.63972	E16.95794	156	CZE
	U0172	subsp. *dioica*	LR	0.575	4	1.1	1.8	1	N48.63972	E16.95794	156	CZE
UP0058	U0173	subsp. *subinermis*	LR	0.310	2	0.9	2.6	1	N48.64958	E16.96333	171	CZE
UP0059	U0174	subsp. *subinermis*	LR	0.308	2	1.0	2.7	1	N48.66172	E16.95553	154	CZE
UP0060	U0175	subsp. *subinermis*	LR	0.308	2	0.9	2.7	1	N48.70031	E16.96467	165	CZE
	U0176	subsp. *subinermis*	LR	0.306	2	1.0	2.9	1	N48.70031	E16.96467	165	CZE
	U3373	subsp. *dioica*	LR	0.572	4	1.0	1.9	1	N48.70031	E16.96467	165	CZE
UP0061	U0177	subsp. *dioica*	LR	0.573	4	1.3	4.0	1	N48.66000	E16.93594	153	CZE
UP0062	U0178	subsp. *dioica*	LR	0.568	4	1.2	1.2	1	N48.65686	E16.93314	151	CZE
	U0179	subsp. *subinermis*	LR	0.306	2	0.9	2.5	1	N48.65686	E16.93314	151	CZE
	U3374	subsp. *dioica*	LR	0.614	4	1.3	2.7	1	N48.65686	E16.93314	151	CZE
UP0063	U0180	subsp. *dioica*	LR	0.577	4	0.8	1.4	1	N48.62083	E16.94406	158	CZE
	U0181	subsp. *dioica*	LR	0.577	4	1.8	1.5	1	N48.62083	E16.94406	158	CZE
UP0064	U0182	subsp. *subinermis*	LR	0.301	2	1.1	2.1	1	N48.64000	E16.94053	151	CZE
	U0183	subsp. *subinermis*	LR	0.311	2	1.3	3.7	1	N48.64000	E16.94053	151	CZE
UP0065	U0184	subsp. *subinermis*	LR	0.324	2	2.2	4.6	1	N48.64742	E16.93908	152	CZE
	U0185	subsp. *dioica*	LR	0.565	4	1.2	1.4	1	N48.64742	E16.93908	152	CZE
	U0620	subsp. *subinermis*	LR	0.314	2	2.7	4.2	1	N48.64742	E16.93908	152	CZE
UP0066	U0186	subsp. *subinermis*	LR	0.305	2	0.9	1.9	1	N48.65292	E16.94225	159	CZE
UP0067	U0187	subsp. *subinermis*	LR	0.309	2	1.1	1.9	1	N48.65369	E16.94275	166	CZE
UP0068	U0188	subsp. *subinermis*	LR	0.309	2	0.8	2.5	1	N48.65456	E16.94178	161	CZE
UP0069	U0189	subsp. *subinermis*	LR	0.304	2	1.2	2.2	1	N48.67939	E16.96081	152	CZE

ID number of population	ID number of analysis	Taxon	Collector	Relative fluorescence intensi	DNA-ploidy level	CV of standard	CV of sample	N. of individuals in analysis	Latitude (WGS-84)	Longitude (WGS-84)	Altitude (m a.s.l.)	Country (ISO 3166-1)
	U0619	subsp. *subinermis*	LR	0.299	2	0.8	2.0	1	N48.67939	E16.96081	152	CZE
UP0070	U0190	subsp. *subinermis*	LR	0.300	2	0.9	2.7	1	N48.67969	E16.95192	151	CZE
UP0071	U0191	subsp. *subinermis*	LR	0.293	2	1.5	2.8	1	N50.28453	E14.50793	168	CZE
UP0072	U0192	subsp. *dioica*	LR	0.570	4	1.3	1.7	1	N50.27928	E14.51105	169	CZE
UP0073	U0193	subsp. *subinermis*	LR	0.293	2	1.5	2.3	1	N50.28708	E14.51275	168	CZE
UP0074	U0194	subsp. *subinermis*	LR	0.293	2	1.4	2.7	1	N50.28723	E14.50978	168	CZE
UP0075	U0195	subsp. *subinermis*	LR	0.294	2	1.2	2.8	1	N50.28649	E14.51192	168	CZE
UP0076	U0196	subsp. *subinermis*	LR	0.296	2	1.4	2.4	1	N50.18081	E14.78856	184	CZE
	U1039	subsp. *subinermis*	LR	0.290	2	1.5	2.5	1	N50.18081	E14.78856	183	CZE
	U1040	subsp. *subinermis*	LR	0.302	2	1.4	2.2	1	N50.18081	E14.78856	183	CZE
	U1041	subsp. *dioica*	LR	0.565	4	1.9	1.6	1	N50.18081	E14.78856	183	CZE
UP0077	U0197	subsp. *subinermis*	LR	0.291	2	1.5	3.7	1	N50.18072	E14.78853	184	CZE
	U1036	subsp. *subinermis*	LR	0.293	2	1.1	2.2	1	N50.18072	E14.78853	184	CZE
	U1037	subsp. *subinermis*	LR	0.299	2	1.2	2.3	1	N50.18072	E14.78853	184	CZE
	U1038	subsp. *subinermis*	LR	0.298	2	1.1	2.5	1	N50.18072	E14.78853	184	CZE
UP0078	U0198	subsp. *dioica*	LR	0.566	4	1.2	1.8	1	N50.18098	E14.78585	184	CZE
UP0079	U0199	subsp. *dioica*	LR	0.572	4	1.9	2.8	1	N50.18042	E14.79227	184	CZE
UP0080	U0200	subsp. *dioica*	LR	0.565	4	1.4	1.9	1	N50.18140	E14.78342	184	CZE
UP0081	U0201	subsp. *subinermis*	LR	0.295	2	1.5	2.3	1	N50.18209	E14.78067	184	CZE
UP0082	U0202	subsp. *subinermis*	LR	0.290	2	1.3	2.8	1	N50.39769	E14.08208	167	CZE
	U0203	subsp. *subinermis*	LR	0.290	2	1.3	2.8	1	N50.39769	E14.08208	167	CZE
	U0643	subsp. *subinermis*	LR	0.295	2	1.1	3.4	1	N50.39769	E14.08208	167	CZE
	U0644	subsp. *subinermis*	LR	0.291	2	1.4	2.9	1	N50.39769	E14.08208	167	CZE

ID number of population	ID number of analysis	Taxon	Collector	Relative fluorescence intensi	DNA-ploidy level	CV of standard	CV of sample	N. of individuals in analysis	Latitude (WGS-84)	Longitude (WGS-84)	Altitude (m a.s.l.)	Country (ISO 3166-1)
	U0645	subsp. *dioica*	LR	0.571	4	1.5	2.0	1	N50.39769	E14.08208	167	CZE
	U0646	subsp. *subinermis*	LR	0.295	2	1.5	2.7	1	N50.39769	E14.08208	167	CZE
UP0083	U0204	subsp. *subinermis*	LR	0.296	2	1.2	2.7	1	N50.39681	E14.08744	167	CZE
	U0908	subsp. *subinermis*	LR	0.304	2	1.2	2.6	1	N50.39681	E14.08744	169	CZE
	U0909	subsp. *subinermis*	LR	0.301	2	2.0	2.7	1	N50.39681	E14.08744	169	CZE
	U0910	subsp. *subinermis*	LR	0.300	2	1.4	2.8	1	N50.39681	E14.08744	169	CZE
	U0911	subsp. *subinermis*	LR	0.293	2	1.5	2.5	1	N50.39681	E14.08744	169	CZE
	U0912	subsp. *subinermis*	LR	0.297	2	1.2	2.9	1	N50.39681	E14.08744	169	CZE
	U0913	subsp. *subinermis*	LR	0.295	2	1.3	3.1	1	N50.39681	E14.08744	169	CZE
UP0084	U0205	subsp. *subinermis*	LR	0.296	2	1.3	2.9	1	N50.39711	E14.08478	167	CZE
	U0206	subsp. *subinermis*	LR	0.296	2	1.3	2.9	1	N50.39711	E14.08478	167	CZE
	U0984	subsp. *subinermis*	LR	0.294	2	2.2	2.9	1	N50.39711	E14.08478	168	CZE
	U0985	subsp. *subinermis*	LR	0.295	2	1.7	3.5	1	N50.39711	E14.08478	168	CZE
	U0986	subsp. *dioica*	LR	0.568	4	1.3	2.3	1	N50.39711	E14.08478	168	CZE
	U0987	subsp. *subinermis*	LR	0.291	2	1.4	2.4	1	N50.39711	E14.08478	168	CZE
	U0988	subsp. *subinermis*	LR	0.293	2	1.3	2.8	1	N50.39711	E14.08478	168	CZE
	U0989	subsp. *subinermis*	LR	0.292	2	1.5	3.1	1	N50.39711	E14.08478	168	CZE
UP0085	U0207	subsp. *dioica*	LR	0.570	4	1.5	2.2	1	N50.39611	E14.07474	167	CZE
UP0086	U0208	subsp. *dioica*	LR	0.559	4	1.4	1.7	1	N50.39353	E14.07360	167	CZE
UP0087	U0209	subsp. *dioica* aneuploid	LR	0.501	–	1.5	1.8	1	N50.39613	E14.08119	167	CZE
UP0088	U0210	subsp. *dioica*	LR	0.564	4	1.3	1.9	1	N50.39478	E14.08373	167	CZE
UP0089	U0211	subsp. *dioica*	LR	0.569	4	1.3	1.7	1	N50.39305	E14.08064	167	CZE
UP0090	U0212	subsp. *subinermis*	LR	0.290	2	1.2	2.5	1	N50.17308	E14.85827	189	CZE

ID number of population	ID number of analysis	Taxon	Collector	Relative fluorescence intensi	DNA-ploidy level	CV of standard	CV of sample	N. of individuals in analysis	Latitude (WGS-84)	Longitude (WGS-84)	Altitude (m a.s.l.)	Country (ISO 3166-1)
UP0091	U0213	subsp. *subinermis*	LR	0.293	2	1.5	4.9	1	N50.17310	E14.85843	189	CZE
	U0214	subsp. *dioica*	LR	0.565	4	1.5	2.6	1	N50.17310	E14.85843	189	CZE
UP0092	U0215	subsp. *subinermis*	LR	0.306	2	1.1	3.7	1	N50.17085	E14.86102	190	CZE
	U3673	subsp. *dioica*	LR	0.558	4	1.2	2.3	1	N50.17085	E14.86102	190	CZE
UP0093	U0216	subsp. *dioica*	LR	0.621	4	1.5	3.2	1	N50.32237	E14.46868	160	CZE
	U0217	subsp. *dioica*	LR	0.582	4	1.8	2.8	1	N50.32237	E14.46868	160	CZE
UP0094	U0218	subsp. *dioica*	LR	0.559	4	1.8	2.2	1	N50.42394	E14.13639	166	CZE
UP0095	U0219	subsp. *dioica*	LR	0.564	4	1.1	1.6	1	N50.41921	E14.13407	166	CZE
UP0096	U0220	subsp. *dioica*	LR	0.558	4	1.7	1.5	1	N50.43053	E14.14561	166	CZE
UP0097	U0221	subsp. *dioica*	LR	0.558	4	1.4	2.1	1	N50.42288	E14.14347	166	CZE
UP0098	U0222	subsp. *dioica*	LR	0.574	4	1.5	1.9	1	N50.41996	E14.14203	166	CZE
UP0099	U0223	subsp. *dioica*	LR	0.555	4	1.1	2.2	1	N50.41632	E14.12938	166	CZE
UP0100	U0224	subsp. *dioica*	LR	0.568	4	1.3	1.4	1	N50.41264	E14.12133	166	CZE
UP0101	U0225	subsp. *dioica*	LR	0.564	4	1.7	1.7	1	N50.42683	E14.14666	166	CZE
UP0102	U0226	subsp. *subinermis*	LR	0.296	2	1.2	2.9	1	N50.24703	E14.54956	2	CZE
	U0811	subsp. *dioica*	LR	0.556	4	1.3	2.3	4	N50.24703	E14.54956	165	CZE
	U0812	subsp. *dioica*	LR	0.556	4	1.3	2.3	4	N50.24703	E14.54956	165	CZE
	U0813	subsp. *dioica*	LR	0.556	4	1.3	2.3	4	N50.24703	E14.54956	165	CZE
	U0814	subsp. *dioica*	LR	0.556	4	1.3	2.3	4	N50.24703	E14.54956	165	CZE
UP0103	U0227	subsp. *subinermis*	LR	0.296	2	1.3	2.8	1	N50.24606	E14.54842	2	CZE
	U0966	subsp. *dioica*	LR	0.562	4	1.4	1.8	1	N50.24606	E14.54842	166	CZE
	U0967	subsp. *subinermis*	LR	0.311	2	1.0	2.8	1	N50.24606	E14.54842	166	CZE
	U0968	subsp. *dioica*	LR	0.562	4	1.6	2.3	1	N50.24606	E14.54842	166	CZE

ID number of population	ID number of analysis	Taxon	Collector	Relative fluorescence intensi	DNA-ploidy level	CV of standard	CV of sample	N. of individuals in analysis	Latitude (WGS-84)	Longitude (WGS-84)	Altitude (m a.s.l.)	Country (ISO 3166-1)
	U3628	subsp. *subinermis*	LR	0.296	2	1.7	3.0	1	N50.24606	E14.54842	166	CZE
UP0104	U0228	subsp. *subinermis*	LR	0.294	2	1.2	2.9	1	N50.24522	E14.54767	2	CZE
	U0721	subsp. *subinermis*	LR	0.292	2	1.4	2.8	1	N50.24522	E14.54767	164	CZE
	U0722	subsp. *subinermis*	LR	0.292	2	1.4	2.4	1	N50.24522	E14.54767	164	CZE
	U0723	subsp. *subinermis*	LR	0.309	2	1.6	4.6	1	N50.24522	E14.54767	164	CZE
	U0724	subsp. *dioica*	LR	0.563	4	1.4	1.8	1	N50.24522	E14.54767	164	CZE
	U3629	subsp. *subinermis*	LR	0.297	2	1.9	2.2	1	N50.24522	E14.54767	164	CZE
UP0105	U0229	subsp. *subinermis*	LR	0.293	2	1.7	2.8	1	N50.24383	E14.54761	2	CZE
	U1323	subsp. *dioica*	LR	0.549	4	1.6	2.5	5	N50.24383	E14.54761	164	CZE
	U1324	subsp. *dioica*	LR	0.549	4	1.6	2.5	5	N50.24383	E14.54761	164	CZE
	U1325	subsp. *dioica*	LR	0.549	4	1.6	2.5	5	N50.24383	E14.54761	164	CZE
	U1326	subsp. *dioica*	LR	0.549	4	1.6	2.5	5	N50.24383	E14.54761	164	CZE
	U1327	subsp. *dioica*	LR	0.549	4	1.6	2.5	5	N50.24383	E14.54761	164	CZE
UP0106	U0230	subsp. *subinermis*	LR	0.294	2	1.6	2.8	1	N50.24169	E14.54850	2	CZE
	U0735	subsp. *dioica*	LR	0.562	4	1.6	2.2	4	N50.24169	E14.54850	166	CZE
	U0736	subsp. *dioica*	LR	0.562	4	1.6	2.2	4	N50.24169	E14.54850	166	CZE
	U0737	subsp. *dioica*	LR	0.562	4	1.6	2.2	4	N50.24169	E14.54850	166	CZE
	U0738	subsp. *dioica*	LR	0.562	4	1.6	2.2	4	N50.24169	E14.54850	166	CZE
UP0107	U0235	subsp. *dioica*	JC	0.569	4	1.3	2.5	1	N47.40778	E25.51889	761	ROU
UP0108	U0236	subsp. *dioica*	HD	0.569	4	0.9	2.4	1	N47.82103	E15.29414	790	AUT
	U0237	subsp. *dioica*	HD	0.616	4	1.3	4.5	1	N47.82103	E15.29414	790	AUT
	U1388	subsp. *dioica*	LR	0.584	4	1.1	3.5	1	N47.82103	E15.29414	790	AUT
	U1389	subsp. *dioica*	LR	0.536	4	1.7	2.3	1	N47.82103	E15.29414	790	AUT

ID number of population	ID number of analysis	Taxon	Collector	Relative fluorescence intensi	DNA-ploidy level	CV of standard	CV of sample	N. of individuals in analysis	Latitude (WGS-84)	Longitude (WGS-84)	Altitude (m a.s.l.)	Country (ISO 3166-1)
	U1390	subsp. *dioica*	LR	0.602	4	1.2	4.6	1	N47.82103	E15.29414	790	AUT
	U1391	subsp. *dioica*	LR	0.598	4	2.2	3.7	1	N47.82103	E15.29414	790	AUT
	U1392	subsp. *dioica*	LR	0.607	4	1.1	3.8	1	N47.82103	E15.29414	790	AUT
	U1393	subsp. *dioica*	LR	0.592	4	0.8	2.5	1	N47.82103	E15.29414	790	AUT
	U1394	subsp. *dioica*	LR	0.601	4	1.5	4.0	1	N47.82103	E15.29414	790	AUT
	U1395	subsp. *dioica*	LR	0.596	4	1.7	2.4	1	N47.82103	E15.29414	790	AUT
UP0109	U0238	subsp. *dioica*	FK	0.604	4	1.7	3.3	1	N54.18744	E15.64299	14	POL
	U1550	subsp. *dioica*	FK	0.599	4	1.1	1.6	1	N54.18744	E15.64299	14	POL
	U1551	subsp. *dioica*	FK	0.594	4	1.0	3.5	1	N54.18744	E15.64299	14	POL
UP0110	U0239	subsp. *dioica*	EZ, FK	0.580	4	1.0	1.9	1	N55.43510	E13.91231	3	SWE
	U1562	subsp. *dioica*	EZ, FK	0.591	4	1.5	2.3	1	N55.43510	E13.91231	3	SWE
	U1563	subsp. *dioica*	EZ, FK	0.590	4	1.3	2.9	1	N55.43510	E13.91231	3	SWE
	U1564	subsp. *dioica*	EZ, FK	0.595	4	0.9	2.8	1	N55.43510	E13.91231	3	SWE
	U1565	subsp. *dioica*	EZ, FK	0.596	4	1.1	1.8	1	N55.43510	E13.91231	3	SWE
	U1566	subsp. *dioica*	EZ, FK	0.607	4	1.9	2.8	1	N55.43510	E13.91231	3	SWE
	U1567	subsp. *dioica*	EZ, FK	0.574	4	1.2	1.8	1	N55.43510	E13.91231	3	SWE
UP0111	U0241	subsp. *dioica*	EZ, FK	0.557	4	1.6	2.4	1	N55.65750	E14.26973	38	SWE
	U1559	subsp. *dioica*	EZ, FK	0.585	4	1.1	1.9	1	N55.65750	E14.26973	38	SWE
	U1561	subsp. *dioica*	EZ, FK	0.602	4	1.4	2.4	1	N55.65750	E14.26973	38	SWE
UP0112	U0242	subsp. *dioica*	FK	0.599	4	1.1	2.3	1	N54.46198	E18.56100	11	POL
	U1570	subsp. *dioica*	EZ, FK	0.576	4	1.3	2.6	1	N54.46198	E18.56100	11	POL
	U1571	subsp. *dioica*	EZ, FK	0.574	4	1.0	3.9	1	N54.46198	E18.56100	11	POL
	U1572	subsp. *dioica*	EZ, FK	0.581	4	1.9	2.4	1	N54.46198	E18.56100	11	POL

ID number of population	ID number of analysis	Taxon	Collector	Relative fluorescence intensi	DNA-ploidy level	CV of standard	CV of sample	N. of individuals in analysis	Latitude (WGS-84)	Longitude (WGS-84)	Altitude (m a.s.l.)	Country (ISO 3166-1)
	U1573	subsp. *dioica*	EZ, FK	0.588	4	0.9	3.1	1	N54.46198	E18.56100	11	POL
UP0113	U0243	subsp. *dioica*	EZ, FK	0.584	4	1.0	3.5	1	N54.28356	E16.13995	2	POL
	U1558	subsp. *dioica*	EZ, FK	0.562	4	1.2	1.5	1	N54.28356	E16.13995	2	POL
	U1568	subsp. *dioica*	EZ, FK	0.578	4	1.0	3.1	1	N54.28356	E16.13995	2	POL
	U1569	subsp. *dioica*	EZ, FK	0.576	4	0.9	3.3	1	N54.28356	E16.13995	2	POL
UP0114	U0252	subsp. *dioica*	FK	0.561	4	1.7	2.4	1	N43.68972	E18.26778	1575	BIH
	U0253	subsp. *dioica*	FK	0.555	4	1.2	1.8	1	N43.68972	E18.26778	1575	BIH
	U0254	subsp. *dioica*	FK	0.604	4	1.3	3.3	1	N43.68972	E18.26778	1575	BIH
	U0255	subsp. *dioica*	FK	0.566	4	1.5	2.2	1	N43.68972	E18.26778	1575	BIH
	U0256	subsp. *dioica*	FK	0.564	4	1.1	2.8	1	N43.68972	E18.26778	1575	BIH
	U0257	subsp. *dioica*	FK	0.574	4	1.3	2.3	1	N43.68972	E18.26778	1575	BIH
UP0115	U0258	subsp. *dioica*	FK	0.572	4	0.8	1.4	1	N55.65798	E14.26849	44	SWE
	U0259	subsp. *dioica*	FK	0.573	4	1.0	1.4	1	N55.65798	E14.26849	44	SWE
UP0116	U0260	subsp. *pubescens*	LR	0.302	2	1.3	1.7	1	N46.11230	E13.11230	129	ITA
	U0261	subsp. *pubescens*	LR	0.300	2	0.6	1.4	1	N46.11230	E13.11230	129	ITA
	U0262	subsp. *dioica* triploid	LR	0.446	3	0.8	1.0	1	N46.11230	E13.11230	129	ITA
UP0117	U0264	subsp. *dioica*	RB	0.578	4	0.8	2.3	1	N46.43976	E13.75280	1424	SVN
	U0265	subsp. *dioica*	RB	0.584	4	0.8	2.4	1	N46.43976	E13.75280	1424	SVN
	U0266	subsp. *dioica*	RB	0.576	4	0.8	1.6	1	N46.43976	E13.75280	1424	SVN
	U0267	subsp. *dioica*	RB	0.567	4	1.2	1.9	1	N46.43976	E13.75280	1424	SVN
UP0118	U0268	subsp. *dioica*	RB	0.617	4	0.8	1.4	1	N46.44113	E13.74176	1808	SVN
	U0269	subsp. *dioica*	RB	0.589	4	0.9	2.0	1	N46.44113	E13.74176	1808	SVN
	U0270	subsp. *dioica*	RB	0.597	4	1.0	1.8	1	N46.44113	E13.74176	1808	SVN

ID number of population	ID number of analysis	Taxon	Collector	Relative fluorescence intensi	DNA-ploidy level	CV of standard	CV of sample	N. of individuals in analysis	Latitude (WGS-84)	Longitude (WGS-84)	Altitude (m a.s.l.)	Country (ISO 3166-1)
UP0119	U0271	subsp. *dioica*	RB	0.574	4	0.8	2.3	1	N46.44071	E13.72535	1676	SVN
	U0272	subsp. *dioica*	RB	0.586	4	1.9	2.8	1	N46.44071	E13.72535	1676	SVN
UP0120	U0273	subsp. *dioica*	RB	0.577	4	1.1	2.0	1	N46.43723	E13.71337	1303	SVN
	U0274	subsp. *dioica*	RB	0.590	4	1.0	1.9	1	N46.43723	E13.71337	1303	SVN
	U0275	subsp. *dioica*	RB	0.595	4	0.9	2.8	1	N46.43723	E13.71337	1303	SVN
	U0276	subsp. *dioica*	RB	0.592	4	1.4	1.9	1	N46.43723	E13.71337	1303	SVN
UP0121	U0277	subsp. *dioica*	RB	0.589	4	1.3	1.8	1	N45.86386	E14.26443	458	SVN
	U0278	subsp. *dioica*	RB	0.581	4	1.1	2.2	1	N45.86386	E14.26443	458	SVN
	U0279	subsp. *dioica*	RB	0.620	4	1.5	2.4	1	N45.86386	E14.26443	458	SVN
UP0122	U0280	subsp. *dioica*	RB	0.598	4	1.7	2.5	1	N45.87358	E14.24645	509	SVN
	U0281	subsp. *dioica*	RB	0.601	4	1.7	2.9	1	N45.87358	E14.24645	509	SVN
	U0282	subsp. *dioica*	RB	0.591	4	1.0	2.5	1	N45.87358	E14.24645	509	SVN
UP0123	U0283	subsp. *dioica*	RB	0.585	4	1.4	1.6	1	N44.37374	E15.46575	1375	HRV
	U0284	subsp. *dioica*	RB	0.564	4	1.1	2.1	1	N44.37374	E15.46575	1375	HRV
	U0285	subsp. *dioica*	RB	0.575	4	1.0	2.6	1	N44.37374	E15.46575	1375	HRV
	U0286	subsp. *dioica*	RB	0.579	4	1.2	1.9	1	N44.37374	E15.46575	1375	HRV
UP0124	U0287	subsp. *dioica*	RB	0.583	4	1.0	1.4	1	N45.82611	E14.24901	454	SVN
	U0288	subsp. *dioica*	RB	0.581	4	0.8	1.2	1	N45.82611	E14.24901	454	SVN
	U0289	subsp. *dioica*	RB	0.572	4	1.2	2.2	1	N45.82611	E14.24901	454	SVN
	U0290	subsp. *dioica*	RB	0.571	4	0.9	3.9	1	N45.82611	E14.24901	377	SVN
	U0291	subsp. *dioica*	RB	0.598	4	1.2	2.8	1	N45.82611	E14.24901	454	SVN
UP0125	U0292	subsp. *dioica*	RB	0.599	4	0.9	2.1	1	N44.76531	E15.21866	501	HRV
	U0293	subsp. *dioica*	RB	0.606	4	1.2	2.3	1	N44.76531	E15.21866	501	HRV

ID number of population	ID number of analysis	Taxon	Collector	Relative fluorescence intensi	DNA-ploidy level	CV of standard	CV of sample	N. of individuals in analysis	Latitude (WGS-84)	Longitude (WGS-84)	Altitude (m a.s.l.)	Country (ISO 3166-1)
UP0126	U0294	subsp. *dioica*	LR	0.606	4	1.2	2.8	1	N48.39592	E21.75039	98	SVK
	U0365	subsp. *subinermis*	LR	0.296	2	1.9	2.3	1	N48.39592	E21.75039	98	SVK
	U0366	subsp. *subinermis*	LR	0.296	2	1.5	2.4	1	N48.39592	E21.75039	98	SVK
UP0127	U0295	subsp. *dioica*	LR	0.584	4	1.2	2.5	1	N48.49319	E22.07675	99	SVK
	U0371	subsp. *subinermis*	LR	0.308	2	1.3	4.1	1	N48.49319	E22.07675	99	SVK
	U0372	subsp. *subinermis*	LR	0.301	2	1.0	2.2	1	N48.49319	E22.07675	99	SVK
	U0373	subsp. *subinermis*	LR	0.297	2	1.0	1.9	1	N48.49319	E22.07675	99	SVK
	U0374	subsp. *subinermis*	LR	0.298	2	1.7	1.8	1	N48.49319	E22.07675	99	SVK
	U0375	subsp. *subinermis*	LR	0.298	2	1.2	2.6	1	N48.49319	E22.07675	99	SVK
	U0376	subsp. *subinermis*	LR	0.298	2	1.2	1.9	1	N48.49319	E22.07675	99	SVK
	U0377	subsp. *dioica*	LR	0.568	4	1.3	1.7	1	N48.49319	E22.07675	99	SVK
UP0128	U0296	subsp. *dioica*	LR	0.578	4	1.3	4.2	1	N48.52582	E22.05363	100	SVK
	U0297	subsp. *dioica*	LR	0.574	4	1.6	2.3	1	N48.52582	E22.05363	100	SVK
	U0298	subsp. *dioica*	LR	0.582	4	1.6	3.1	1	N48.52582	E22.05363	100	SVK
UP0129	U0299	subsp. *subinermis*	LR	0.297	2	1.3	2.6	1	N48.49417	E21.91414	102	SVK
	U0300	subsp. *subinermis*	LR	0.302	2	1.2	2.2	1	N48.49417	E21.91414	102	SVK
	U0301	subsp. *subinermis*	LR	0.290	2	1.3	2.8	1	N48.49417	E21.91414	102	SVK
	U0302	subsp. *subinermis*	LR	0.300	2	1.4	2.3	1	N48.49417	E21.91414	102	SVK
	U0303	subsp. *subinermis*	LR	0.303	2	1.3	2.4	1	N48.49417	E21.91414	102	SVK
	U0304	subsp. *subinermis*	LR	0.301	2	1.9	1.8	1	N48.49417	E21.91414	102	SVK
	U0420	subsp. *subinermis*	LR	0.301	2	1.4	2.2	1	N48.49417	E21.91414	102	SVK
	U0421	subsp. *subinermis*	LR	0.295	2	1.5	2.0	1	N48.49417	E21.91414	102	SVK
UP0130	U0306	subsp. *subinermis*	LR	0.290	2	1.3	2.2	1	N48.56303	E21.92361	99	SVK

ID number of population	ID number of analysis	Taxon	Collector	Relative fluorescence intensi	DNA-ploidy level	CV of standard	CV of sample	N. of individuals in analysis	Latitude (WGS-84)	Longitude (WGS-84)	Altitude (m a.s.l.)	Country (ISO 3166-1)
	U0307	subsp. *subinermis*	LR	0.294	2	1.3	2.4	1	N48.56303	E21.92361	99	SVK
	U0308	subsp. *subinermis*	LR	0.293	2	1.4	2.4	1	N48.56303	E21.92361	99	SVK
UP0131	U0309	subsp. *dioica*	LR	0.568	4	1.4	1.9	1	N48.56621	E21.94052	107	SVK
	U0310	subsp. *subinermis*	LR	0.295	2	1.4	2.4	1	N48.56621	E21.94052	107	SVK
	U0433	subsp. *subinermis*	LR	0.294	2	1.1	2.4	1	N48.56621	E21.94052	107	SVK
UP0132	U0311	subsp. *subinermis*	LR	0.300	2	1.4	2.7	6	N48.49647	E21.87483	99	SVK
UP0133	U0312	subsp. *subinermis*	LR	0.296	2	1.3	1.8	1	N48.49114	E21.84125	99	SVK
	U0313	subsp. *subinermis*	LR	0.296	2	1.6	1.9	1	N48.49114	E21.84125	99	SVK
	U0314	subsp. *subinermis*	LR	0.300	2	1.1	2.1	1	N48.49114	E21.84125	99	SVK
	U0315	subsp. *subinermis*	LR	0.294	2	1.4	2.0	1	N48.49114	E21.84125	99	SVK
	U0316	subsp. *dioica*	LR	0.574	4	1.3	1.7	1	N48.49114	E21.84125	99	SVK
	U0317	subsp. *subinermis*	LR	0.303	2	1.3	2.2	1	N48.49114	E21.84125	99	SVK
	U0318	subsp. *subinermis*	LR	0.295	2	1.3	2.0	1	N48.49114	E21.84125	99	SVK
	U0319	subsp. *subinermis*	LR	0.300	2	1.2	2.2	1	N48.49114	E21.84125	99	SVK
	U0437	subsp. *subinermis*	LR	0.308	2	0.9	4.2	1	N48.49114	E21.84125	99	SVK
	U0438	subsp. *dioica*	LR	0.571	4	1.2	1.7	1	N48.49114	E21.84125	99	SVK
UP0134	U0320	subsp. *subinermis*	LR	0.304	2	1.7	1.9	1	N48.48933	E21.86347	98	SVK
	U0321	subsp. *subinermis*	LR	0.308	2	1.3	4.1	1	N48.48933	E21.86347	98	SVK
	U0322	subsp. *subinermis*	LR	0.295	2	1.1	2.0	1	N48.48933	E21.86347	98	SVK
	U0323	subsp. *subinermis*	LR	0.305	2	1.0	2.7	1	N48.48933	E21.86347	98	SVK
	U0324	subsp. *dioica*	LR	0.569	4	1.0	1.4	1	N48.48933	E21.86347	98	SVK
	U0325	subsp. *dioica*	LR	0.575	4	1.3	3.8	1	N48.48933	E21.86347	98	SVK
	U0326	subsp. *dioica*	LR	0.583	4	1.3	1.6	1	N48.48933	E21.86347	98	SVK

ID number of population	ID number of analysis	Taxon	Collector	Relative fluorescence intensi	DNA-ploidy level	CV of standard	CV of sample	N. of individuals in analysis	Latitude (WGS-84)	Longitude (WGS-84)	Altitude (m a.s.l.)	Country (ISO 3166-1)
	U0327	subsp. *subinermis*	LR	0.310	2	1.0	4.5	1	N48.48933	E21.86347	98	SVK
	U0328	subsp. *dioica*	LR	0.573	4	1.0	1.4	1	N48.48933	E21.86347	98	SVK
	U0329	subsp. *dioica*	LR	0.594	4	1.2	2.2	1	N48.48933	E21.86347	98	SVK
	U0330	subsp. *dioica*	LR	0.566	4	1.2	1.6	1	N48.48933	E21.86347	98	SVK
	U3499	subsp. *subinermis*	LR	0.299	2	1.5	2.3	5	N48.48933	E21.86347	98	SVK
UP0135	U0331	subsp. *dioica*	LR	0.560	4	1.3	1.8	1	N48.48892	E21.85806	97	SVK
	U0332	subsp. *subinermis*	LR	0.296	2	1.1	2.5	1	N48.48892	E21.85806	97	SVK
	U0333	subsp. *subinermis*	LR	0.290	2	1.9	1.7	1	N48.48892	E21.85806	97	SVK
	U0334	subsp. *subinermis*	LR	0.294	2	1.3	2.2	1	N48.48892	E21.85806	97	SVK
	U0335	subsp. *subinermis*	LR	0.295	2	1.4	2.1	1	N48.48892	E21.85806	97	SVK
	U0336	subsp. *dioica*	LR	0.568	4	1.9	1.8	1	N48.48892	E21.85806	97	SVK
	U0337	subsp. *subinermis*	LR	0.295	2	1.1	1.8	1	N48.48892	E21.85806	97	SVK
	U0338	subsp. *subinermis*	LR	0.291	2	1.2	1.9	1	N48.48892	E21.85806	97	SVK
	U0439	subsp. *subinermis*	LR	0.293	2	1.3	2.4	1	N48.48892	E21.85806	97	SVK
	U0440	subsp. *subinermis*	LR	0.297	2	1.1	2.7	1	N48.48892	E21.85806	97	SVK
UP0136	U0339	subsp. *dioica*	LR	0.561	4	0.9	1.4	1	N48.48475	E21.84961	98	SVK
	U0340	subsp. *subinermis*	LR	0.290	2	1.3	2.3	1	N48.48475	E21.84961	98	SVK
	U0341	subsp. *subinermis*	LR	0.293	2	1.4	1.9	1	N48.48475	E21.84961	98	SVK
	U0342	subsp. *subinermis*	LR	0.292	2	1.4	2.2	1	N48.48475	E21.84961	98	SVK
	U0343	subsp. *subinermis*	LR	0.289	2	1.2	1.8	1	N48.48475	E21.84961	98	SVK
	U0344	subsp. *subinermis*	LR	0.292	2	1.3	1.7	1	N48.48475	E21.84961	98	SVK
	U0345	subsp. *dioica*	LR	0.562	4	1.2	1.5	1	N48.48475	E21.84961	98	SVK
	U0346	subsp. *dioica*	LR	0.572	4	1.2	1.9	1	N48.48475	E21.84961	98	SVK

ID number of population	ID number of analysis	Taxon	Collector	Relative fluorescence intensi	DNA-ploidy level	CV of standard	CV of sample	N. of individuals in analysis	Latitude (WGS-84)	Longitude (WGS-84)	Altitude (m a.s.l.)	Country (ISO 3166-1)
UP0137	U0347	subsp. *subinermis*	LR	0.301	2	1.2	2.6	1	N48.43256	E21.81092	96	SVK
	U0348	subsp. *subinermis*	LR	0.301	2	1.3	2.2	1	N48.43256	E21.81092	96	SVK
	U3495	subsp. *subinermis*	LR	0.302	2	1.0	2.7	1	N48.43256	E21.81092	96	SVK
UP0138	U0349	subsp. *subinermis*	LR	0.298	2	1.1	2.1	1	N48.43264	E21.81125	97	SVK
	U0350	subsp. *subinermis*	LR	0.299	2	1.5	2.5	1	N48.43264	E21.81125	97	SVK
	U0351	subsp. *subinermis*	LR	0.315	2	1.4	2.9	1	N48.43264	E21.81125	97	SVK
UP0139	U0352	subsp. *dioica*	LR	0.576	4	1.3	1.9	4	N48.43898	E22.05241	99	SVK
	U0353	subsp. *dioica*	LR	0.576	4	1.3	1.9	4	N48.43898	E22.05241	99	SVK
	U0354	subsp. *dioica*	LR	0.576	4	1.3	1.9	4	N48.43898	E22.05241	99	SVK
UP0140	U0355	subsp. *dioica*	LR	0.560	4	1.2	1.6	1	N48.37817	E21.75256	96	SVK
UP0141	U0356	subsp. *dioica*	LR	0.567	4	1.2	2.1	1	N48.38456	E21.72042	98	SVK
	U0357	subsp. *dioica*	LR	0.569	4	1.4	1.9	2	N48.38456	E21.72042	98	SVK
	U0358	subsp. *dioica*	LR	0.569	4	1.4	1.9	2	N48.38456	E21.72042	98	SVK
UP0142	U0359	subsp. *dioica*	LR	0.569	4	1.5	2.2	1	N48.38431	E21.71758	98	SVK
	U0360	subsp. *dioica*	LR	0.569	4	1.5	2.2	1	N48.38431	E21.71758	98	SVK
	U0361	subsp. *dioica*	LR	0.569	4	1.5	2.2	1	N48.38431	E21.71758	98	SVK
	U0362	subsp. *subinermis*	LR	0.300	2	1.5	2.2	1	N48.38431	E21.71758	98	SVK
	U3487	subsp. *dioica*	LR	0.562	4	1.6	1.8	1	N48.38431	E21.71758	98	SVK
UP0143	U0363	subsp. *dioica*	LR	0.571	4	1.7	1.6	2	N48.39672	E21.80689	99	SVK
	U0364	subsp. *dioica*	LR	0.571	4	1.7	1.6	2	N48.39672	E21.80689	99	SVK
UP0144	U0367	subsp. *dioica*	LR	0.556	4	1.7	1.8	1	N48.40111	E21.77503	97	SVK
	U0368	subsp. *dioica*	LR	0.559	4	1.8	1.8	1	N48.40111	E21.77503	97	SVK
	U0369	subsp. *subinermis*	LR	0.297	2	1.3	2.5	1	N48.40111	E21.77503	97	SVK

ID number of population	ID number of analysis	Taxon	Collector	Relative fluorescence intensi	DNA-ploidy level	CV of standard	CV of sample	N. of individuals in analysis	Latitude (WGS-84)	Longitude (WGS-84)	Altitude (m a.s.l.)	Country (ISO 3166-1)
	U3489	subsp. *dioica*	LR	0.572	4	1.2	1.8	4	N48.40111	E21.77503	97	SVK
UP0145	U0370	subsp. *subinermis*	LR	0.295	2	1.9	2.5	1	N48.45681	E21.80497	96	SVK
UP0146	U0378	subsp. *dioica*	LR	0.572	4	1.1	1.9	1	N48.50069	E22.07319	97	SVK
UP0147	U0379	subsp. *dioica*	LR	0.567	4	1.0	2.0	7	N48.50556	E22.05856	104	SVK
	U0380	subsp. *dioica*	LR	0.567	4	1.0	2.0	7	N48.50556	E22.05856	104	SVK
	U0381	subsp. *dioica*	LR	0.567	4	1.0	2.0	7	N48.50556	E22.05856	104	SVK
	U0382	subsp. *dioica*	LR	0.567	4	1.0	2.0	7	N48.50556	E22.05856	104	SVK
	U0383	subsp. *dioica*	LR	0.567	4	1.0	2.0	7	N48.50556	E22.05856	104	SVK
	U0384	subsp. *dioica*	LR	0.567	4	1.0	2.0	7	N48.50556	E22.05856	104	SVK
	U0385	subsp. *dioica*	LR	0.567	4	1.0	2.0	7	N48.50556	E22.05856	104	SVK
UP0148	U0386	subsp. *subinermis*	LR	0.308	2	1.1	3.1	1	N48.47631	E22.10681	106	SVK
	U0387	subsp. *subinermis*	LR	0.294	2	1.2	2.4	1	N48.47631	E22.10681	106	SVK
	U0388	subsp. *subinermis*	LR	0.297	2	1.1	2.6	1	N48.47631	E22.10681	106	SVK
	U0389	subsp. *subinermis*	LR	0.293	2	1.2	2.1	1	N48.47631	E22.10681	106	SVK
	U0390	subsp. *subinermis*	LR	0.296	2	1.1	2.2	1	N48.47631	E22.10681	106	SVK
	U0391	subsp. *subinermis*	LR	0.301	2	1.2	2.2	1	N48.47631	E22.10681	106	SVK
	U0392	subsp. *subinermis*	LR	0.296	2	1.3	2.5	1	N48.47631	E22.10681	106	SVK
	U0393	subsp. *subinermis*	LR	0.298	2	1.7	2.1	1	N48.47631	E22.10681	106	SVK
	U0394	subsp. *subinermis*	LR	0.295	2	1.1	2.4	1	N48.47631	E22.10681	106	SVK
UP0149	U0395	subsp. *subinermis*	LR	0.300	2	1.3	2.5	1	N48.47397	E22.10978	101	SVK
	U0396	subsp. *subinermis*	LR	0.297	2	1.4	2.0	1	N48.47397	E22.10978	101	SVK
	U0397	subsp. *subinermis*	LR	0.299	2	1.9	1.9	1	N48.47397	E22.10978	101	SVK
	U0398	subsp. *subinermis*	LR	0.299	2	1.4	2.1	1	N48.47397	E22.10978	101	SVK

ID number of population	ID number of analysis	Taxon	Collector	Relative fluorescence intensi	DNA-ploidy level	CV of standard	CV of sample	N. of individuals in analysis	Latitude (WGS-84)	Longitude (WGS-84)	Altitude (m a.s.l.)	Country (ISO 3166-1)
	U3491	subsp. *dioica*	LR	0.571	4	1.4	1.9	1	N48.47397	E22.10978	101	SVK
UP0150	U0399	subsp. *dioica* triploid	LR	0.428	3	1.4	2.0	1	N48.47567	E22.08292	99	SVK
	U0400	subsp. *subinermis*	LR	0.302	2	1.1	2.9	1	N48.47567	E22.08292	99	SVK
	U0401	subsp. *subinermis*	LR	0.299	2	1.4	2.3	1	N48.47567	E22.08292	99	SVK
	U0402	subsp. *subinermis*	LR	0.330	2	1.4	2.5	1	N48.47567	E22.08292	99	SVK
	U0403	subsp. *dioica*	LR	0.566	4	1.1	1.8	1	N48.47567	E22.08292	99	SVK
	U0404	subsp. *dioica*	LR	0.549	4	1.3	1.6	1	N48.47567	E22.08292	99	SVK
	U3492	subsp. *dioica*	LR	0.572	4	1.1	1.7	1	N48.47567	E22.08292	99	SVK
	U3493	subsp. *dioica*	LR	0.566	4	1.6	2.0	1	N48.47567	E22.08292	99	SVK
UP0151	U0405	subsp. *dioica*	LR	0.569	4	1.5	2.2	4	N48.50586	E22.03997	99	SVK
	U0406	subsp. *dioica*	LR	0.569	4	1.5	2.2	4	N48.50586	E22.03997	99	SVK
	U0407	subsp. *dioica*	LR	0.569	4	1.5	2.2	4	N48.50586	E22.03997	99	SVK
	U0408	subsp. *dioica*	LR	0.569	4	1.5	2.2	4	N48.50586	E22.03997	99	SVK
UP0152	U0409	subsp. *subinermis*	LR	0.347	2	1.3	1.9	1	N48.50389	E21.99969	104	SVK
	U0410	subsp. *subinermis*	LR	0.351	2	1.3	2.0	1	N48.50389	E21.99969	104	SVK
	U0411	subsp. *subinermis*	LR	0.348	2	1.1	1.6	1	N48.50389	E21.99969	104	SVK
	U0412	subsp. *subinermis*	LR	0.350	2	1.3	1.8	1	N48.50389	E21.99969	104	SVK
	U0413	subsp. *subinermis*	LR	0.347	2	1.2	1.9	1	N48.50389	E21.99969	104	SVK
UP0153	U0414	subsp. *dioica*	LR	0.560	4	1.5	2.8	6	N48.50342	E21.99950	106	SVK
	U0415	subsp. *dioica*	LR	0.560	4	1.5	2.8	6	N48.50342	E21.99950	106	SVK
	U0416	subsp. *dioica*	LR	0.560	4	1.5	2.8	6	N48.50342	E21.99950	106	SVK
	U0417	subsp. *dioica*	LR	0.560	4	1.5	2.8	6	N48.50342	E21.99950	106	SVK
	U0418	subsp. *dioica*	LR	0.560	4	1.5	2.8	6	N48.50342	E21.99950	106	SVK

ID number of population	ID number of analysis	Taxon	Collector	Relative fluorescence intensi	DNA-ploidy level	CV of standard	CV of sample	N. of individuals in analysis	Latitude (WGS-84)	Longitude (WGS-84)	Altitude (m a.s.l.)	Country (ISO 3166-1)
	U0419	subsp. *dioica*	LR	0.560	4	1.5	2.8	6	N48.50342	E21.99950	106	SVK
UP0154	U0422	subsp. *dioica*	LR	0.568	4	0.9	1.5	1	N48.49397	E21.88883	98	SVK
	U0423	subsp. *subinermis*	LR	0.297	2	1.3	1.7	1	N48.49397	E21.88883	98	SVK
UP0155	U0424	subsp. *dioica*	LR	0.570	4	1.2	1.7	1	N48.51689	E21.82989	97	SVK
	U0425	subsp. *dioica*	LR	0.571	4	1.4	2.5	4	N48.52953	E21.85953	98	SVK
	U0426	subsp. *dioica*	LR	0.571	4	1.4	2.5	4	N48.52953	E21.85953	98	SVK
	U0427	subsp. *dioica*	LR	0.571	4	1.4	2.5	4	N48.52953	E21.85953	98	SVK
	U0428	subsp. *dioica*	LR	0.571	4	1.4	2.5	4	N48.52953	E21.85953	98	SVK
UP0157	U0429	subsp. *dioica*	LR	0.568	4	1.3	2.6	2	N48.53208	E21.87092	100	SVK
	U0430	subsp. *dioica*	LR	0.568	4	1.3	2.6	2	N48.53208	E21.87092	100	SVK
UP0158	U0431	subsp. *dioica*	LR	0.569	4	1.4	1.2	2	N48.57586	E21.81897	96	SVK
	U0432	subsp. *dioica*	LR	0.569	4	1.4	1.2	2	N48.57586	E21.81897	96	SVK
UP0159	U0434	subsp. *dioica*	LR	0.561	4	1.2	2.0	3	N48.53100	E21.95314	97	SVK
UP0159	U0435	subsp. *dioica*	LR	0.561	4	1.2	2.0	3	N48.53100	E21.95314	97	SVK
	U0436	subsp. *dioica*	LR	0.561	4	1.2	2.0	3	N48.53100	E21.95314	97	SVK
UP0160	U0441	subsp. *dioica*	LR	0.570	4	1.2	1.7	2	N48.48653	E21.82119	101	SVK
	U0442	subsp. *dioica*	LR	0.570	4	1.2	1.7	2	N48.48653	E21.82119	101	SVK
UP0161	U0443	subsp. *dioica*	PK	0.564	4	1.6	1.4	1	N50.02215	E15.33557	206	CZE
	U0444	subsp. *dioica*	PK	0.556	4	1.8	1.3	1	N50.02215	E15.33557	206	CZE
	U0445	subsp. *dioica*	PK	0.564	4	1.8	1.4	1	N50.02215	E15.33557	206	CZE
	U0446	subsp. *subinermis*	PK	0.299	2	1.8	1.6	1	N50.02215	E15.33557	206	CZE
	U0447	subsp. *dioica*	PK	0.559	4	1.3	1.5	1	N50.02215	E15.33557	206	CZE
	U0448	subsp. *dioica*	PK	0.564	4	1.1	1.4	1	N50.02215	E15.33557	206	CZE

ID number of population	ID number of analysis	Taxon	Collector	Relative fluorescence intensi	DNA-ploidy level	CV of standard	CV of sample	N. of individuals in analysis	Latitude (WGS-84)	Longitude (WGS-84)	Altitude (m a.s.l.)	Country (ISO 3166-1)
	U0449	subsp. *dioica*	PK	0.563	4	1.9	1.4	1	N50.02215	E15.33557	206	CZE
	U0450	subsp. *subinermis*	PK	0.295	2	1.2	2.2	1	N50.02215	E15.33557	206	CZE
	U0451	subsp. *dioica*	PK	0.569	4	1.0	1.3	1	N50.02215	E15.33557	206	CZE
	U0452	subsp. *dioica*	PK	0.577	4	1.2	1.5	1	N50.02215	E15.33557	206	CZE
	U0453	subsp. *dioica* triploid	PK	0.419	3	1.7	1.4	1	N50.02215	E15.33557	206	CZE
	U0454	subsp. *dioica*	PK	0.558	4	1.2	1.5	1	N50.02215	E15.33557	206	CZE
	U0455	subsp. *dioica*	PK	0.571	4	1.9	1.5	1	N50.02215	E15.33557	206	CZE
	U0456	subsp. *subinermis*	PK	0.300	2	1.1	1.8	1	N50.02215	E15.33557	206	CZE
	U0457	subsp. *subinermis*	PK	0.297	2	1.2	1.5	1	N50.02215	E15.33557	206	CZE
	U0458	subsp. *dioica*	PK	0.570	4	1.2	1.2	1	N50.02215	E15.33557	206	CZE
	U0459	subsp. *dioica*	PK	0.575	4	1.1	1.3	1	N50.02215	E15.33557	206	CZE
UP0162	U0460	subsp. *dioica*	FK	0.573	4	1.0	1.9	1	N37.92900	E14.64242	1476	ITA
	U0461	subsp. *dioica*	FK	0.570	4	1.3	1.7	1	N37.94089	E14.95978	1156	ITA
UP0164	U0462	subsp. *dioica*	MD	0.571	4	0.7	1.9	1	N48.38822	E20.35841	219	SVK
UP0165	U0463	subsp. *dioica*	JS	0.599	4	0.9	1.8	1	N42.49840	E13.55948	1475	ITA
UP0166	U0464	subsp. *dioica*	JS	0.553	4	0.9	2.2	1	N43.55795	E17.55951	1108	BIH
UP0167	U0465	subsp. *dioica*	JS	0.560	4	0.9	1.4	1	N40.21851	E15.30390	933	ITA
UP0168	U0466	subsp. *dioica*	MD	0.580	4	0.9	1.1	1	N47.64431	E18.65664	158	HUN
UP0169	U0467	subsp. *dioica*	MD	0.587	4	0.9	1.1	1	N48.62273	E18.40494	858	SVK
UP0170	U0468	subsp. *dioica*	MD	0.566	4	0.9	1.8	1	N48.95489	E18.41347	559	SVK
UP0171	U0469	subsp. *dioica*	MD	0.577	4	1.5	3.0	1	N48.34711	E21.83508	95	HUN
UP0172	U0470	subsp. *dioica*	MD	0.572	4	0.8	1.8	1	N48.98817	E20.77507	575	SVK
UP0173	U0471	subsp. *dioica*	MD	0.584	4	0.8	1.2	1	N49.04994	E20.94231	527	SVK

ID number of population	ID number of analysis	Taxon	Collector	Relative fluorescence intensi	DNA-ploidy level	CV of standard	CV of sample	N. of individuals in analysis	Latitude (WGS-84)	Longitude (WGS-84)	Altitude (m a.s.l.)	Country (ISO 3166-1)
UP0174	U0472	subsp. *dioica*	MD	0.578	4	1.0	2.2	1	N48.75956	E21.24889	217	SVK
UP0175	U0473	subsp. *dioica*	MD	0.577	4	0.7	1.5	1	N48.55978	E21.75992	98	SVK
UP0176	U0474	subsp. *dioica*	MD	0.573	4	0.7	1.4	1	N49.01931	E20.98331	440	SVK
UP0177	U0475	subsp. *dioica*	MD	0.576	4	0.9	1.5	1	N48.23083	E19.94181	215	SVK
UP0178	U0476	subsp. *dioica*	MD	0.567	4	0.8	1.3	1	N46.31056	E7.68744	623	CHE
UP0179	U0477	subsp. *dioica*	MD	0.580	4	1.2	2.1	1	N48.99053	E21.58161	142	SVK
UP0180	U0478	subsp. *dioica*	MD	0.593	4	0.7	1.4	1	N45.70981	E7.24778	624	ITA
UP0181	U0479	subsp. *dioica*	MD	0.584	4	1.0	2.3	1	N48.29758	E20.34151	151	HUN
UP0182	U0480	subsp. *dioica*	MD	0.603	4	1.5	3.5	1	N48.74836	E21.25531	213	SVK
	U3430	subsp. *dioica*	MD	0.565	4	0.7	1.6	1	N48.74836	E21.25531	214	SVK
UP0183	U0481	subsp. *dioica*	MD	0.586	4	1.6	1.3	1	N48.37356	E19.89053	199	SVK
UP0184	U0482	subsp. *dioica*	MD	0.574	4	0.7	2.2	1	N48.62754	E20.50255	258	SVK
UP0185	U0483	subsp. *subinermis*	MD	0.301	2	0.8	2.4	1	N48.56439	E21.92725	101	SVK
UP0186	U0484	subsp. *subinermis*	MD	0.308	2	1.0	1.7	1	N48.49103	E21.84189	99	SVK
UP0187	U0485	subsp. *subinermis*	MD	0.303	2	0.9	2.3	1	N46.16592	E14.68781	327	SVN
UP0188	U0486	subsp. *dioica*	MD	0.566	4	1.3	2.0	1	N48.43319	E19.59025	225	SVK
UP0189	U0487	subsp. *dioica*	JS	0.565	4	0.8	1.6	1	N48.43206	E21.98053	99	SVK
UP0190	U0488	subsp. *dioica*	MD	0.566	4	0.8	1.6	1	N48.24692	E20.07567	190	SVK
UP0191	U0489	subsp. *dioica*	MD	0.580	4	0.9	2.2	1	N48.39789	E20.24789	181	SVK
UP0192	U0490	subsp. *dioica*	MD	0.563	4	0.7	1.6	1	N49.04611	E20.94158	517	SVK
UP0193	U0491	subsp. *dioica*	MD	0.571	4	1.0	2.3	1	N49.05328	E21.51717	166	SVK
UP0194	U0492	subsp. *dioica*	MD	0.580	4	0.7	1.5	1	N48.32500	E20.27417	172	SVK
UP0195	U0493	subsp. *dioica*	FK	0.574	4	0.9	1.9	4	N61.63853	E8.05066	952	NOR

ID number of population	ID number of analysis	Taxon	Collector	Relative fluorescence intensi	DNA-ploidy level	CV of standard	CV of sample	N. of individuals in analysis	Latitude (WGS-84)	Longitude (WGS-84)	Altitude (m a.s.l.)	Country (ISO 3166-1)
	U0494	subsp. *dioica*	FK	0.574	4	0.9	1.9	4	N61.63853	E8.05066	952	NOR
	U0495	subsp. *dioica*	FK	0.574	4	0.9	1.9	4	N61.63853	E8.05066	952	NOR
UP0196	U0498	subsp. *dioica*	KK	0.573	4	1.4	1.3	1	N53.57794	W6.61408	139	IRL
	U0499	subsp. *dioica*	KK	0.590	4	1.3	1.0	1	N53.57794	W6.61408	139	IRL
UP0197	U0500	subsp. *dioica*	KK	0.581	4	0.9	1.1	1	N53.64978	W9.88042	7	IRL
	U0501	subsp. *dioica*	KK	0.579	4	0.5	1.0	1	N53.64978	W9.88042	7	IRL
UP0198	U0502	subsp. *dioica*	KK	0.576	4	1.2	1.8	2	N52.61472	W9.38231	2	IRL
	U0503	subsp. *dioica*	KK	0.576	4	1.2	1.8	2	N52.61472	W9.38231	2	IRL
UP0199	U0504	subsp. *dioica*	KK	0.580	4	0.7	1.3	2	N53.14589	W9.27217	18	IRL
	U0505	subsp. *dioica*	KK	0.580	4	0.7	1.3	2	N53.14589	W9.27217	18	IRL
UP0200	U0506	subsp. *dioica*	PT, ZC	0.562	4	1.1	1.5	1	N37.98109	E15.14355	1021	ITA
	U3476	subsp. *dioica*	PT, ZC	0.569	4	1.2	1.7	1	N37.98109	E15.14355	1021	ITA
	U3477	subsp. *dioica*	PT, ZC	0.571	4	1.6	2.0	1	N37.98109	E15.14355	1021	ITA
	U3478	subsp. *dioica*	PT, ZC	0.564	4	1.2	1.8	1	N37.98109	E15.14355	1021	ITA
	U3479	subsp. *dioica*	PT, ZC	0.568	4	1.4	1.7	1	N37.98109	E15.14355	1021	ITA
	U3480	subsp. *dioica*	PT, ZC	0.569	4	1.3	1.5	1	N37.98109	E15.14355	1021	ITA
UP0201	U0509	subsp. *dioica*	PT, ZC	0.577	4	1.4	1.4	1	N37.85794	E13.38822	1130	ITA
	U3481	subsp. *dioica*	PT, ZC	0.574	4	1.2	1.6	1	N37.85794	E13.38822	1130	ITA
	U3482	subsp. *dioica*	PT, ZC	0.580	4	1.2	1.6	1	N37.85794	E13.38822	1130	ITA
	U3483	subsp. *dioica*	PT, ZC	0.577	4	1.0	1.4	1	N37.85794	E13.38822	1130	ITA
	U3484	subsp. *dioica*	PT, ZC	0.580	4	1.6	1.8	1	N37.85794	E13.38822	1130	ITA
	U3485	subsp. *dioica*	PT, ZC	0.572	4	1.3	1.8	1	N37.85794	E13.38822	1130	ITA
UP0202	U0511	subsp. *dioica*	PT, ZC	0.572	4	1.2	1.7	1	N37.91019	E13.99032	860	ITA

ID number of population	ID number of analysis	Taxon	Collector	Relative fluorescence intensi	DNA-ploidy level	CV of standard	CV of sample	N. of individuals in analysis	Latitude (WGS-84)	Longitude (WGS-84)	Altitude (m a.s.l.)	Country (ISO 3166-1)
	U0512	subsp. *dioica*	PT, ZC	0.568	4	1.1	1.7	1	N37.91019	E13.99032	860	ITA
	U0513	subsp. *dioica*	PT, ZC	0.575	4	1.3	1.7	1	N37.91019	E13.99032	860	ITA
	U0514	subsp. *dioica*	PT, ZC	0.576	4	1.3	1.9	1	N37.91019	E13.99032	860	ITA
	U0515	subsp. *dioica*	PT, ZC	0.576	4	1.2	1.9	1	N37.91019	E13.99032	860	ITA
	U0516	subsp. *dioica*	PT, ZC	0.575	4	1.5	2.0	1	N37.91019	E13.99032	860	ITA
UP0203	U0551	subsp. *dioica*	TU	0.619	4	1.7	3.2	1	N40.10269	E29.12839	1928	TUR
	U0553	subsp. *dioica*	TU	0.598	4	0.8	2.6	1	N40.10269	E29.12839	1928	TUR
	U0554	subsp. *dioica*	TU	0.593	4	1.5	2.5	1	N40.10269	E29.12839	1928	TUR
UP0204	U0559	subsp. *dioica*	TU	0.623	4	1.2	4.4	1	N40.10264	E29.12814	1930	TUR
	U0560	subsp. *dioica*	TU	0.601	4	1.7	2.2	1	N40.10264	E29.12814	1930	TUR
	U0561	subsp. *dioica*	TU	0.581	4	1.2	2.8	1	N40.10264	E29.12814	1930	TUR
UP0205	U0568	subsp. *dioica*	TU	0.602	4	1.4	2.1	1	N43.27950	E22.06336	326	SRB
	U0568	subsp. *dioica*	TU	0.616	4	1.5	3.0	1	N43.27950	E22.06336	326	SRB
	U0569	subsp. *dioica*	TU	0.591	4	0.9	3.9	1	N43.27950	E22.06336	326	SRB
	U0570	subsp. *dioica*	TU	0.595	4	1.2	2.0	1	N43.27950	E22.06336	326	SRB
	U0570	subsp. *dioica*	TU	0.623	4	1.3	2.8	1	N43.27950	E22.06336	326	SRB
	U0572	subsp. *dioica*	TU	0.589	4	1.3	2.0	1	N43.27950	E22.06336	326	SRB
	U0574	subsp. *dioica*	TU	0.607	4	1.4	3.5	1	N43.27950	E22.06336	326	SRB
UP0206	U0575	subsp. *dioica*	LR	0.579	4	1.2	2.4	1	N52.40698	E13.05818	34	DEU
	U1402	subsp. *dioica*	LR	0.571	4	0.8	2.5	1	N52.40698	E13.05818	34	DEU
	U3102	subsp. *dioica*	LR	0.599	4	1.4	1.9	1	N52.40698	E13.05818	34	DEU
UP0207	U0577	subsp. *dioica*	LR	0.611	4	0.8	2.4	1	N56.94440	E24.12362	12	LVA
	U1403	subsp. *dioica*	LR	0.628	4	1.1	4.9	1	N56.94440	E24.12362	12	LVA

ID number of population	ID number of analysis	Taxon	Collector	Relative fluorescence intensi	DNA-ploidy level	CV of standard	CV of sample	N. of individuals in analysis	Latitude (WGS-84)	Longitude (WGS-84)	Altitude (m a.s.l.)	Country (ISO 3166-1)
	U1404	subsp. *dioica*	LR	0.625	4	1.2	4.3	1	N56.94440	E24.12362	12	LVA
	U1405	subsp. *dioica*	LR	0.637	4	1.9	2.9	1	N56.94440	E24.12362	12	LVA
UP0208	U0578	subsp. *dioica*	LR	0.560	4	1.1	1.7	1	N56.99637	E23.92158	12	LVA
UP0209	U0579	subsp. *dioica*	LR	0.578	4	1.2	2.8	1	N57.16542	E24.84953	90	LVA
	U0580	subsp. *dioica*	LR	0.592	4	1.3	3.4	1	N57.16542	E24.84953	90	LVA
	U1417	subsp. *dioica*	LR	0.593	4	0.9	2.5	1	N57.16542	E24.84953	90	LVA
UP0210	U0581	subsp. *dioica*	LR	0.568	4	0.8	2.7	1	N57.17635	E24.84768	23	LVA
	U0582	subsp. *dioica*	LR	0.596	4	1.4	3.9	1	N57.17635	E24.84768	23	LVA
	U0583	subsp. *dioica*	LR	0.582	4	1.0	2.7	1	N57.17635	E24.84768	23	LVA
	U1418	subsp. *dioica*	LR	0.550	4	0.9	1.4	1	N57.17635	E24.84768	23	LVA
	U1419	subsp. *dioica*	LR	0.578	4	0.9	3.7	1	N57.17635	E24.84768	23	LVA
UP0211	U0584	subsp. *subinermis*	LR	0.319	2	0.8	2.1	1	N56.65638	E23.73452	3	LVA
	U0585	subsp. *subinermis*	LR	0.315	2	0.7	2.4	1	N56.65638	E23.73452	3	LVA
	U0586	subsp. *subinermis*	LR	0.311	2	0.9	2.8	1	N56.65638	E23.73452	3	LVA
	U0587	subsp. *dioica*	LR	0.567	4	0.9	1.7	1	N56.65638	E23.73452	3	LVA
UP0212	U0588	subsp. *dioica*	LR	0.591	4	1.2	2.8	1	N56.62198	E23.27367	42	LVA
	U1423	subsp. *dioica*	LR	0.586	4	1.1	3.9	1	N56.62198	E23.27367	42	LVA
	U1424	subsp. *dioica*	LR	0.569	4	0.8	1.6	1	N56.62198	E23.27367	42	LVA
	U1425	subsp. *dioica*	LR	0.595	4	2.3	3.4	1	N56.62198	E23.27367	42	LVA
UP0213	U0589	subsp. *dioica*	LR	0.587	4	1.3	2.3	1	N56.40147	E24.17733	14	LVA
	U1432	subsp. *subinermis*	LR	0.307	2	1.8	3.5	1	N56.40147	E24.17733	14	LVA
	U1433	subsp. *subinermis*	LR	0.315	2	1.4	4.5	1	N56.40147	E24.17733	14	LVA
UP0214	U0590	subsp. *dioica*	LR	0.581	4	1.0	2.2	1	N56.91195	E23.45655	20	LVA

ID number of population	ID number of analysis	Taxon	Collector	Relative fluorescence intensi	DNA-ploidy level	CV of standard	CV of sample	N. of individuals in analysis	Latitude (WGS-84)	Longitude (WGS-84)	Altitude (m a.s.l.)	Country (ISO 3166-1)
	U0591	subsp. *dioica*	LR	0.572	4	0.8	1.6	1	N56.91195	E23.45655	20	LVA
	U1434	subsp. *dioica*	LR	0.573	4	0.8	1.8	1	N56.91195	E23.45655	20	LVA
	U1435	subsp. *dioica*	LR	0.583	4	1.3	2.7	1	N56.91195	E23.45655	20	LVA
UP0215	U0592	subsp. *dioica*	LR	0.580	4	1.7	2.9	1	N57.00145	E23.48883	2	LVA
	U1436	subsp. *subinermis*	LR	0.316	2	1.6	4.3	1	N57.00145	E23.48883	2	LVA
UP0216	U0593	subsp. *dioica*	LR	0.593	4	1.5	2.8	1	N56.96787	E21.98196	19	LVA
	U1437	subsp. *dioica*	LR	0.560	4	3.1	2.7	1	N56.96787	E21.98196	19	LVA
	U1438	subsp. *dioica*	LR	0.595	4	1.8	3.5	1	N56.96787	E21.98196	19	LVA
	U1439	subsp. *dioica*	LR	0.592	4	0.8	2.9	1	N56.96787	E21.98196	19	LVA
	U1440	subsp. *dioica*	LR	0.570	4	1.4	4.3	1	N56.96787	E21.98196	19	LVA
	U1441	subsp. *dioica*	LR	0.623	4	2.6	4.7	1	N56.96787	E21.98196	19	LVA
UP0217	U0594	subsp. *subinermis*	LR	0.299	2	1.1	2.4	1	N56.95191	E23.51314	11	LVA
	U0595	subsp. *subinermis*	LR	0.303	2	1.5	2.3	1	N56.95191	E23.51314	11	LVA
	U0596	subsp. *subinermis*	LR	0.300	2	1.3	3.4	1	N56.95191	E23.51314	11	LVA
	U0597	subsp. *subinermis*	LR	0.304	2	0.9	3.2	1	N56.95191	E23.51314	11	LVA
	U0598	subsp. *subinermis*	LR	0.296	2	1.5	2.9	1	N56.95191	E23.51314	11	LVA
	U0599	subsp. *subinermis*	LR	0.303	2	0.9	2.8	1	N56.95191	E23.51314	11	LVA
	U0600	subsp. *subinermis*	LR	0.308	2	0.8	2.5	1	N56.95191	E23.51314	11	LVA
	U1399	subsp. *subinermis*	LR	0.307	2	1.4	3.6	1	N56.95191	E23.51314	11	LVA
	U1400	subsp. *subinermis*	LR	0.303	2	0.9	2.4	1	N56.95191	E23.51314	11	LVA
	U1401	subsp. *subinermis*	LR	0.298	2	1.5	4.0	1	N56.95191	E23.51314	11	LVA
	U2230	subsp. *subinermis*	LR, SS	0.324	2	1.1	3.1	1	N56.95191	E23.51314	2	LVA
	U2231	subsp. *subinermis*	LR, SS	0.314	2	1.0	3.4	1	N56.95191	E23.51314	2	LVA

ID number of population	ID number of analysis	Taxon	Collector	Relative fluorescence intensi	DNA-ploidy level	CV of standard	CV of sample	N. of individuals in analysis	Latitude (WGS-84)	Longitude (WGS-84)	Altitude (m a.s.l.)	Country (ISO 3166-1)
	U2232	subsp. *subinermis*	LR, SS	0.321	2	0.9	4.4	1	N56.95191	E23.51314	2	LVA
	U2233	subsp. *subinermis*	LR, SS	0.324	2	1.4	4.8	1	N56.95191	E23.51314	2	LVA
	U2234	subsp. *subinermis*	LR, SS	0.304	2	0.9	3.9	1	N56.95191	E23.51314	2	LVA
	U2287	subsp. *subinermis*	LR, SS	0.307	2	0.7	1.9	1	N56.95191	E23.51314	2	LVA
UP0218	U0607	subsp. *dioica*	LR	0.557	4	1.5	1.8	1	N49.35188	E14.14547	355	CZE
	U0608	subsp. *subinermis*	LR	0.290	2	1.8	3.4	1	N49.35188	E14.14547	355	CZE
	U0609	subsp. *dioica*	LR	0.567	4	1.7	1.9	1	N49.35188	E14.14547	355	CZE
	U0610	subsp. *dioica*	LR	0.557	4	1.3	1.7	1	N49.35188	E14.14547	355	CZE
	U1632	subsp. *subinermis*	LR	0.307	2	1.2	3.5	1	N49.35188	E14.14547	355	CZE
	U3558	subsp. *dioica*	LR	0.571	4	0.9	2.8	1	N49.35188	E14.14547	354	CZE
	U3559	subsp. *subinermis*	LR	0.303	2	0.8	1.8	1	N49.35188	E14.14547	354	CZE
UP0219	U0611	subsp. *subinermis*	LR	0.308	2	0.9	2.5	1	N48.64450	E16.93106	151	CZE
	U0612	subsp. *subinermis*	LR	0.310	2	0.7	2.1	1	N48.64450	E16.93106	151	CZE
	U0613	subsp. *subinermis*	LR	0.309	2	0.9	3.8	1	N48.64450	E16.93106	151	CZE
	U1339	subsp. *subinermis*	LR	0.316	2	1.0	2.7	1	N48.64450	E16.93106	151	CZE
UP0220	U0614	subsp. *subinermis*	LR	0.308	2	0.8	2.4	1	N48.63342	E16.93131	150	CZE
	U0615	subsp. *subinermis*	LR	0.314	2	0.8	2.6	1	N48.63342	E16.93131	150	CZE
	U0616	subsp. *subinermis*	LR	0.305	2	0.9	2.4	1	N48.63342	E16.93131	150	CZE
	U1338	subsp. *subinermis*	LR	0.303	2	0.9	3.4	1	N48.63342	E16.93131	150	CZE
UP0221	U0617	subsp. *dioica*	LR	0.568	4	1.1	2.0	1	N48.66231	E16.96006	156	CZE
	U0618	subsp. *dioica*	LR	0.579	4	1.8	3.5	1	N48.66231	E16.96006	156	CZE
UP0222	U0621	subsp. *dioica*	JC	0.573	4	1.5	2.7	1	N38.89164	E21.87532	1116	GRC
	U0622	subsp. *dioica*	JC	0.575	4	1.3	2.3	1	N38.89164	E21.87532	1116	GRC

ID number of population	ID number of analysis	Taxon	Collector	Relative fluorescence intensi	DNA-ploidy level	CV of standard	CV of sample	N. of individuals in analysis	Latitude (WGS-84)	Longitude (WGS-84)	Altitude (m a.s.l.)	Country (ISO 3166-1)
	U0623	subsp. *dioica*	JC	0.576	4	1.3	2.5	1	N38.89164	E21.87532	1116	GRC
UP0223	U0624	subsp. *dioica*	JC	0.572	4	1.2	2.4	1	N40.05764	E21.08280	1773	GRC
	U0625	subsp. *dioica*	JC	0.593	4	1.4	2.3	1	N40.05764	E21.08280	1773	GRC
UP0224	U0626	subsp. *dioica*	JC	0.583	4	1.4	1.6	1	N39.40366	E21.20857	1353	GRC
	U0627	subsp. *dioica*	JC	0.582	4	1.2	2.6	1	N39.40366	E21.20857	1353	GRC
UP0225	U0629	subsp. *dioica*	JC	0.575	4	1.3	2.4	1	N40.91141	E24.14438	1259	GRC
UP0226	U0630	subsp. *dioica*	FK	0.590	4	1.3	1.8	1	N55.05460	E12.22547	1	DNK
	U0631	subsp. *dioica*	FK	0.588	4	1.2	2.3	1	N55.05460	E12.22547	1	DNK
	U0632	subsp. *dioica*	FK	0.610	4	1.3	2.8	1	N55.05460	E12.22547	1	DNK
	U0633	subsp. *dioica*	FK	0.619	4	1.4	3.0	1	N55.05460	E12.22547	1	DNK
	U0634	subsp. *dioica*	FK	0.603	4	1.4	3.3	1	N55.05460	E12.22547	1	DNK
UP0227	U0635	subsp. *dioica*	LR	0.603	4	1.5	4.3	1	N49.50166	E14.16706	437	CZE
UP0228	U0636a	subsp. *dioica*	LR	0.599	4	1.2	3.0	1	N49.51617	E14.02895	436	CZE
	U0636b	subsp. *dioica* aneuploid	LR	0.515	–	1.1	3.5	1	N49.51617	E14.02895	436	CZE
	U1621	subsp. *dioica*	LR	0.562	4	1.2	1.5	1	N49.51617	E14.02895	440	CZE
	U3593	subsp. *dioica* aneuploid	LR	0.493	–	0.8	1.6	1	N49.51617	E14.02895	440	CZE
	U3594	subsp. *dioica* aneuploid	LR	0.492	–	0.7	1.6	1	N49.51617	E14.02895	440	CZE
UP0229	U0637	subsp. *dioica* aneuploid	LR	0.511	–	1.2	2.5	1	N49.51559	E14.17692	385	CZE
UP0230	U0638	subsp. *dioica*	LR	0.593	4	1.5	3.3	1	N49.50997	E14.16918	403	CZE
UP0231	U0640	subsp. *dioica*	LR	0.597	4	1.5	2.8	1	N49.51100	E14.16913	388	CZE
UP0232	U0641	subsp. *dioica*	LR	0.577	4	1.2	2.5	1	N49.51172	E14.16955	367	CZE
UP0233	U0647	subsp. *dioica*	LR	0.561	4	1.4	2.2	2	N50.44772	E14.16108	158	CZE
	U0648	subsp. *dioica*	LR	0.561	4	1.4	2.2	2	N50.44772	E14.16108	158	CZE

ID number of population	ID number of analysis	Taxon	Collector	Relative fluorescence intensi	DNA-ploidy level	CV of standard	CV of sample	N. of individuals in analysis	Latitude (WGS-84)	Longitude (WGS-84)	Altitude (m a.s.l.)	Country (ISO 3166-1)
UP0234	U0649	subsp. *dioica*	LR	0.571	4	1.2	1.9	4	N49.32331	E14.70072	401	CZE
	U0650	subsp. *dioica*	LR	0.571	4	1.2	1.9	4	N49.32331	E14.70072	401	CZE
	U0651	subsp. *dioica*	LR	0.571	4	1.2	1.9	4	N49.32331	E14.70072	401	CZE
	U0652	subsp. *dioica*	LR	0.571	4	1.2	1.9	4	N49.32331	E14.70072	401	CZE
UP0235	U0653	subsp. *dioica*	LR	0.555	4	1.5	2.3	3	N50.39789	E14.08228	168	CZE
	U0654	subsp. *dioica*	LR	0.555	4	1.5	2.3	3	N50.39789	E14.08228	168	CZE
	U0655	subsp. *dioica*	LR	0.555	4	1.5	2.3	3	N50.39789	E14.08228	168	CZE
UP0236	U0656	subsp. *subinermis*	LR	0.297	2	1.8	2.6	1	N50.42700	E14.14594	164	CZE
	U0657	subsp. *subinermis*	LR	0.298	2	1.5	3.4	1	N50.42700	E14.14594	164	CZE
UP0237	U0658	subsp. *dioica*	LR	0.603	4	1.2	2.7	1	N49.21056	E13.50467	493	CZE
	U0659	subsp. *dioica*	LR	0.624	4	1.3	3.9	1	N49.21056	E13.50467	493	CZE
	U0660	subsp. *dioica*	LR	0.578	4	1.2	3.9	1	N49.21056	E13.50467	493	CZE
	U0661	subsp. *dioica*	LR	0.619	4	1.1	1.3	1	N49.21056	E13.50467	493	CZE
UP0238	U0662	subsp. *dioica*	LR	0.573	4	1.5	2.2	3	N49.32142	E14.70525	404	CZE
	U0663	subsp. *dioica*	LR	0.573	4	1.5	2.2	3	N49.32142	E14.70525	404	CZE
	U0664	subsp. *dioica*	LR	0.573	4	1.5	2.2	3	N49.32142	E14.70525	404	CZE
UP0239	U0665	subsp. *dioica*	LR	0.584	4	1.5	1.7	3	N49.32136	E14.70553	404	CZE
	U0666	subsp. *dioica*	LR	0.584	4	1.5	1.7	3	N49.32136	E14.70553	404	CZE
	U0667	subsp. *dioica*	LR	0.584	4	1.5	1.7	3	N49.32136	E14.70553	404	CZE
UP0240	U0668	subsp. *dioica*	LR	0.572	4	1.4	2.6	2	N50.39733	E14.08528	170	CZE
	U0669	subsp. *dioica*	LR	0.572	4	1.4	2.6	2	N50.39733	E14.08528	170	CZE
UP0241	U0670	subsp. *dioica*	LR	0.576	4	1.3	2.8	4	N50.45239	E14.16394	167	CZE
	U0671	subsp. *dioica*	LR	0.576	4	1.3	2.8	4	N50.45239	E14.16394	167	CZE

ID number of population	ID number of analysis	Taxon	Collector	Relative fluorescence intensi	DNA-ploidy level	CV of standard	CV of sample	N. of individuals in analysis	Latitude (WGS-84)	Longitude (WGS-84)	Altitude (m a.s.l.)	Country (ISO 3166-1)
	U0672	subsp. *dioica*	LR	0.576	4	1.3	2.8	4	N50.45239	E14.16394	167	CZE
	U0673	subsp. *dioica*	LR	0.576	4	1.3	2.8	4	N50.45239	E14.16394	167	CZE
UP0242	U0674	subsp. *dioica*	LR	0.564	4	1.2	2.0	4	N50.45181	E14.15969	160	CZE
	U0675	subsp. *dioica*	LR	0.564	4	1.2	2.0	4	N50.45181	E14.15969	160	CZE
	U0676	subsp. *dioica*	LR	0.564	4	1.2	2.0	4	N50.45181	E14.15969	160	CZE
	U0677	subsp. *dioica*	LR	0.564	4	1.2	2.0	4	N50.45181	E14.15969	160	CZE
UP0243	U0678	subsp. *dioica*	LR	0.569	4	1.4	2.5	3	N49.37944	E14.14217	426	CZE
	U0679	subsp. *dioica*	LR	0.569	4	1.4	2.5	3	N49.37944	E14.14217	426	CZE
	U0680	subsp. *dioica*	LR	0.569	4	1.4	2.5	3	N49.37944	E14.14217	426	CZE
UP0244	U0681	subsp. *dioica*	LR	0.556	4	1.2	1.9	5	N49.21192	E13.50492	498	CZE
	U0682	subsp. *dioica*	LR	0.556	4	1.2	1.9	5	N49.21192	E13.50492	498	CZE
	U0683	subsp. *dioica*	LR	0.556	4	1.2	1.9	5	N49.21192	E13.50492	498	CZE
	U0684	subsp. *dioica*	LR	0.556	4	1.2	1.9	5	N49.21192	E13.50492	498	CZE
UP0245	U0685	subsp. *dioica*	LR	0.566	4	1.3	1.8	1	N49.37989	E14.14444	415	CZE
	U0686	subsp. *dioica*	LR	0.581	4	1.1	1.8	1	N49.37989	E14.14444	415	CZE
	U0687	subsp. *dioica*	LR	0.557	4	1.2	1.6	1	N49.37989	E14.14444	415	CZE
UP0246	U0688	subsp. *dioica*	LR	0.572	4	1.1	1.9	4	N49.26539	E13.58014	511	CZE
	U0689	subsp. *dioica*	LR	0.572	4	1.1	1.9	4	N49.26539	E13.58014	511	CZE
	U0690	subsp. *dioica*	LR	0.572	4	1.1	1.9	4	N49.26539	E13.58014	511	CZE
	U0691	subsp. *dioica*	LR	0.572	4	1.1	1.9	4	N49.26539	E13.58014	511	CZE
UP0247	U0692	subsp. *dioica*	LR	0.581	4	1.4	2.3	2	N50.19203	E14.67086	172	CZE
	U0693	subsp. *dioica*	LR	0.581	4	1.4	2.3	2	N50.19203	E14.67086	172	CZE
UP0248	U0694	subsp. *dioica*	LR	0.569	4	1.3	1.9	4	N49.37900	E14.14022	438	CZE

ID number of population	ID number of analysis	Taxon	Collector	Relative fluorescence intensi	DNA-ploidy level	CV of standard	CV of sample	N. of individuals in analysis	Latitude (WGS-84)	Longitude (WGS-84)	Altitude (m a.s.l.)	Country (ISO 3166-1)
	U0695	subsp. *dioica*	LR	0.569	4	1.3	1.9	4	N49.37900	E14.14022	438	CZE
	U0696	subsp. *dioica*	LR	0.569	4	1.3	1.9	4	N49.37900	E14.14022	438	CZE
	U0697	subsp. *dioica*	LR	0.569	4	1.3	1.9	4	N49.37900	E14.14022	438	CZE
UP0249	U0698	subsp. *dioica*	LR	0.559	4	1.4	1.8	2	N50.00110	E14.44140	254	CZE
	U0699	subsp. *dioica*	LR	0.559	4	1.4	1.8	2	N50.00110	E14.44140	254	CZE
UP0250	U0700	subsp. *dioica*	LR	0.544	4	1.4	2.0	6	N49.25972	E13.94703	387	CZE
	U0701	subsp. *dioica*	LR	0.544	4	1.4	2.0	6	N49.25972	E13.94703	387	CZE
	U0702	subsp. *dioica*	LR	0.544	4	1.4	2.0	6	N49.25972	E13.94703	387	CZE
	U0703	subsp. *dioica*	LR	0.544	4	1.4	2.0	6	N49.25972	E13.94703	387	CZE
	U0704	subsp. *dioica*	LR	0.544	4	1.4	2.0	6	N49.25972	E13.94703	387	CZE
	U0705	subsp. *dioica*	LR	0.544	4	1.4	2.0	6	N49.25972	E13.94703	387	CZE
UP0251	U0706	subsp. *dioica*	LR	0.563	4	1.4	2.2	6	N49.21244	E13.50589	498	CZE
	U0707	subsp. *dioica*	LR	0.563	4	1.4	2.2	6	N49.21244	E13.50589	498	CZE
	U0708	subsp. *dioica*	LR	0.563	4	1.4	2.2	6	N49.21244	E13.50589	498	CZE
	U0709	subsp. *dioica*	LR	0.563	4	1.4	2.2	6	N49.21244	E13.50589	498	CZE
	U0710	subsp. *dioica*	LR	0.563	4	1.4	2.2	6	N49.21244	E13.50589	498	CZE
	U0711	subsp. *dioica*	LR	0.563	4	1.4	2.2	6	N49.21244	E13.50589	498	CZE
UP0252	U0712	subsp. *dioica*	LR	0.576	4	1.7	2.4	3	N50.19222	E14.67117	171	CZE
	U0713	subsp. *dioica*	LR	0.576	4	1.7	2.4	3	N50.19222	E14.67117	171	CZE
	U0714	subsp. *dioica*	LR	0.576	4	1.7	2.4	3	N50.19222	E14.67117	171	CZE
UP0253	U0715	subsp. *dioica*	LR	0.565	4	1.6	2.2	6	N49.81252	E14.32170	285	CZE
	U0716	subsp. *dioica*	LR	0.565	4	1.6	2.2	6	N49.81252	E14.32170	285	CZE
	U0717	subsp. *dioica*	LR	0.565	4	1.6	2.2	6	N49.81252	E14.32170	285	CZE

ID number of population	ID number of analysis	Taxon	Collector	Relative fluorescence intensi	DNA-ploidy level	CV of standard	CV of sample	N. of individuals in analysis	Latitude (WGS-84)	Longitude (WGS-84)	Altitude (m a.s.l.)	Country (ISO 3166-1)
	U0718	subsp. *dioica*	LR	0.565	4	1.6	2.2	6	N49.81252	E14.32170	285	CZE
	U0719	subsp. *dioica*	LR	0.565	4	1.6	2.2	6	N49.81252	E14.32170	285	CZE
	U0720	subsp. *dioica*	LR	0.565	4	1.6	2.2	6	N49.81252	E14.32170	285	CZE
UP0254	U0725	subsp. *dioica*	LR	0.567	4	1.2	1.9	3	N50.32378	E14.47419	164	CZE
	U0726	subsp. *dioica*	LR	0.567	4	1.2	1.9	3	N50.32378	E14.47419	164	CZE
	U0727	subsp. *dioica*	LR	0.567	4	1.2	1.9	3	N50.32378	E14.47419	164	CZE
UP0255	U0728	subsp. *dioica*	LR	0.543	4	2.9	2.1	1	N49.28494	E14.69825	398	CZE
UP0256	U0729	subsp. *dioica*	LR	0.564	4	1.2	1.9	2	N49.23392	E14.71339	402	CZE
	U0730	subsp. *dioica*	LR	0.564	4	1.2	1.9	2	N49.23392	E14.71339	402	CZE
UP0257	U0731	subsp. *dioica*	LR	0.561	4	1.4	2.1	4	N50.42769	E14.14625	165	CZE
	U0732	subsp. *dioica*	LR	0.561	4	1.4	2.1	4	N50.42769	E14.14625	165	CZE
	U0733	subsp. *dioica*	LR	0.561	4	1.4	2.1	4	N50.42769	E14.14625	165	CZE
	U0734	subsp. *dioica*	LR	0.561	4	1.4	2.1	4	N50.42769	E14.14625	165	CZE
UP0258	U0739	subsp. *dioica*	LR	0.567	4	1.3	2.2	3	N50.44908	E14.16089	164	CZE
	U0740	subsp. *dioica*	LR	0.567	4	1.3	2.2	3	N50.44908	E14.16089	164	CZE
	U0741	subsp. *dioica*	LR	0.567	4	1.3	2.2	3	N50.44908	E14.16089	164	CZE
UP0259	U0742	subsp. *dioica*	LR	0.583	4	1.1	1.9	1	N50.44933	E14.16033	165	CZE
UP0260	U0743	subsp. *dioica*	LR	0.558	4	1.4	2.6	5	N50.25561	E14.54397	181	CZE
	U0744	subsp. *dioica*	LR	0.558	4	1.4	2.6	5	N50.25561	E14.54397	181	CZE
	U0745	subsp. *dioica*	LR	0.558	4	1.4	2.6	5	N50.25561	E14.54397	181	CZE
	U0746	subsp. *dioica*	LR	0.558	4	1.4	2.6	5	N50.25561	E14.54397	181	CZE
	U0747	subsp. *dioica*	LR	0.558	4	1.4	2.6	5	N50.25561	E14.54397	181	CZE
UP0261	U0748	subsp. *dioica*	LR	0.581	4	1.3	1.8	3	N49.32194	E14.70639	409	CZE

ID number of population	ID number of analysis	Taxon	Collector	Relative fluorescence intensi	DNA-ploidy level	CV of standard	CV of sample	N. of individuals in analysis	Latitude (WGS-84)	Longitude (WGS-84)	Altitude (m a.s.l.)	Country (ISO 3166-1)
	U0749	subsp. *dioica*	LR	0.581	4	1.3	1.8	3	N49.32194	E14.70639	409	CZE
	U0750	subsp. *dioica*	LR	0.581	4	1.3	1.8	3	N49.32194	E14.70639	409	CZE
UP0262	U0751	subsp. *dioica*	LR	0.562	4	1.4	2.3	1	N48.80530	E16.64211	364	CZE
UP0263	U0752	subsp. *dioica*	LR	0.562	4	1.4	2.0	2	N49.03861	E14.80639	426	CZE
	U0753	subsp. *dioica*	LR	0.562	4	1.4	2.0	2	N49.03861	E14.80639	426	CZE
UP0264	U0754	subsp. *dioica*	LR	0.563	4	1.1	1.9	2	N49.23344	E14.71922	404	CZE
	U0755	subsp. *dioica*	LR	0.563	4	1.1	1.9	2	N49.23344	E14.71922	404	CZE
UP0265	U0756	subsp. *dioica*	LR	0.562	4	1.7	1.8	3	N49.28400	E14.70047	399	CZE
	U0757	subsp. *dioica*	LR	0.562	4	1.7	1.8	3	N49.28400	E14.70047	399	CZE
	U0758	subsp. *dioica*	LR	0.562	4	1.7	1.8	3	N49.28400	E14.70047	399	CZE
UP0266	U0759	subsp. *dioica*	LR	0.564	4	1.9	2.1	3	N49.03794	E14.80583	432	CZE
	U0760	subsp. *dioica*	LR	0.564	4	1.9	2.1	3	N49.03794	E14.80583	432	CZE
	U0761	subsp. *dioica*	LR	0.564	4	1.9	2.1	3	N49.03794	E14.80583	432	CZE
UP0267	U0762	subsp. *dioica*	LR	0.569	4	1.0	2.1	4	N50.45183	E14.15956	160	CZE
	U0763	subsp. *dioica*	LR	0.569	4	1.0	2.1	4	N50.45183	E14.15956	160	CZE
	U0764	subsp. *dioica*	LR	0.569	4	1.0	2.1	4	N50.45183	E14.15956	160	CZE
	U0765	subsp. *dioica*	LR	0.569	4	1.0	2.1	4	N50.45183	E14.15956	160	CZE
UP0268	U0766	subsp. *dioica*	LR	0.571	4	1.2	2.1	3	N49.26003	E13.94394	389	CZE
	U0767	subsp. *dioica*	LR	0.571	4	1.2	2.1	3	N49.26003	E13.94394	389	CZE
	U0768	subsp. *dioica*	LR	0.571	4	1.2	2.1	3	N49.26003	E13.94394	389	CZE
UP0269	U0769	subsp. *dioica*	LR	0.567	4	1.1	2.4	4	N49.26011	E13.94556	385	CZE
	U0770	subsp. *dioica*	LR	0.567	4	1.1	2.4	4	N49.26011	E13.94556	385	CZE
	U0771	subsp. *dioica*	LR	0.567	4	1.1	2.4	4	N49.26011	E13.94556	385	CZE

ID number of population	ID number of analysis	Taxon	Collector	Relative fluorescence intensi	DNA-ploidy level	CV of standard	CV of sample	N. of individuals in analysis	Latitude (WGS-84)	Longitude (WGS-84)	Altitude (m a.s.l.)	Country (ISO 3166-1)
	U0772	subsp. *dioica*	LR	0.567	4	1.1	2.4	4	N49.26011	E13.94556	385	CZE
UP0270	U0773	subsp. *dioica*	LR	0.574	4	1.5	2.6	3	N50.39744	E14.08536	170	CZE
	U0774	subsp. *dioica*	LR	0.574	4	1.5	2.6	3	N50.39744	E14.08536	170	CZE
	U0775	subsp. *dioica*	LR	0.574	4	1.5	2.6	3	N50.39744	E14.08536	170	CZE
UP0271	U0776	subsp. *dioica*	LR	0.567	4	1.8	2.2	1	N50.42703	E14.14703	162	CZE
	U0777	subsp. *subinermis*	LR	0.293	2	1.2	3.1	1	N50.42703	E14.14703	162	CZE
UP0272	U0778	subsp. *dioica*	LR	0.576	4	1.7	2.3	3	N49.28417	E14.70069	399	CZE
	U0779	subsp. *dioica*	LR	0.576	4	1.7	2.3	3	N49.28417	E14.70069	399	CZE
	U0780	subsp. *dioica*	LR	0.576	4	1.7	2.3	3	N49.28417	E14.70069	399	CZE
UP0273	U0781	subsp. *dioica*	LR	0.578	4	1.7	2.6	3	N49.32139	E14.70381	403	CZE
	U0782	subsp. *dioica*	LR	0.578	4	1.7	2.6	3	N49.32139	E14.70381	403	CZE
	U0783	subsp. *dioica*	LR	0.578	4	1.7	2.6	3	N49.32139	E14.70381	403	CZE
UP0274	U0784	subsp. *dioica*	LR	0.573	4	1.8	2.1	2	N49.28564	E14.69819	400	CZE
	U0785	subsp. *dioica*	LR	0.573	4	1.8	2.1	2	N49.28564	E14.69819	400	CZE
UP0275	U0786	subsp. *dioica*	LR	0.596	4	1.2	2.6	1	N49.03333	E17.44667	175	CZE
	U0787	subsp. *dioica*	LR	0.606	4	1.5	2.7	1	N49.03333	E17.44667	175	CZE
	U0788	subsp. *dioica*	LR	0.619	4	1.5	3.4	1	N49.03333	E17.44667	175	CZE
	U0789	subsp. *dioica*	LR	0.590	4	1.2	2.3	1	N49.03333	E17.44667	175	CZE
UP0276	U0790	subsp. *dioica*	LR	0.569	4	1.2	2.2	8	N50.61189	E13.87884	224	CZE
	U0791	subsp. *dioica*	LR	0.569	4	1.2	2.2	8	N50.61189	E13.87884	224	CZE
	U0792	subsp. *dioica*	LR	0.569	4	1.2	2.2	8	N50.61189	E13.87884	224	CZE
	U0793	subsp. *dioica*	LR	0.569	4	1.2	2.2	8	N50.61189	E13.87884	224	CZE
	U0794	subsp. *dioica*	LR	0.569	4	1.2	2.2	8	N50.61189	E13.87884	224	CZE

ID number of population	ID number of analysis	Taxon	Collector	Relative fluorescence intensi	DNA-ploidy level	CV of standard	CV of sample	N. of individuals in analysis	Latitude (WGS-84)	Longitude (WGS-84)	Altitude (m a.s.l.)	Country (ISO 3166-1)
	U0795	subsp. *dioica*	LR	0.569	4	1.2	2.2	8	N50.61189	E13.87884	224	CZE
	U0796	subsp. *dioica*	LR	0.569	4	1.2	2.2	8	N50.61189	E13.87884	224	CZE
	U0797	subsp. *dioica*	LR	0.569	4	1.2	2.2	8	N50.61189	E13.87884	224	CZE
UP0277	U0798	subsp. *dioica*	LR	0.565	4	1.7	2.6	3	N50.19172	E14.67044	173	CZE
	U0799	subsp. *dioica*	LR	0.565	4	1.7	2.6	3	N50.19172	E14.67044	173	CZE
	U0800	subsp. *dioica*	LR	0.565	4	1.7	2.6	3	N50.19172	E14.67044	173	CZE
UP0278	U0801	subsp. *dioica*	LR	0.578	4	1.3	1.9	1	N50.19261	E14.66986	170	CZE
	U0802	subsp. *dioica*	LR	0.589	4	1.1	1.9	1	N50.19261	E14.66986	170	CZE
	U0803	subsp. *dioica*	LR	0.571	4	1.3	2.1	1	N50.19261	E14.66986	170	CZE
	U0804	subsp. *dioica*	LR	0.582	4	1.4	3.3	1	N50.19261	E14.66986	170	CZE
	U3638	subsp. *dioica*	LR	0.560	4	1.6	2.2	1	N50.19261	E14.66986	170	CZE
UP0279	U0805	subsp. *dioica*	LR	0.573	4	1.4	2.1	2	N50.25914	E14.54375	167	CZE
	U0806	subsp. *dioica*	LR	0.573	4	1.4	2.1	2	N50.25914	E14.54375	167	CZE
UP0280	U0807	subsp. *dioica*	LR	0.552	4	1.4	1.8	1	N50.32214	E14.46925	163	CZE
	U0808	subsp. *dioica*	LR	0.568	4	1.6	2.7	1	N50.32214	E14.46925	163	CZE
UP0281	U0809	subsp. *dioica*	LR	0.569	4	1.7	2.1	2	N50.32278	E14.47306	163	CZE
	U0810	subsp. *dioica*	LR	0.569	4	1.7	2.1	2	N50.32278	E14.47306	163	CZE
UP0282	U0815	subsp. *dioica*	LR	0.574	4	1.7	2.0	3	N50.25797	E14.54425	167	CZE
	U0816	subsp. *dioica*	LR	0.574	4	1.7	2.0	3	N50.25797	E14.54425	167	CZE
	U0817	subsp. *dioica*	LR	0.574	4	1.7	2.0	3	N50.25797	E14.54425	167	CZE
UP0283	U0818	subsp. *dioica*	LR	0.570	4	1.7	2.7	2	N50.25656	E14.54411	177	CZE
	U0819	subsp. *dioica*	LR	0.570	4	1.7	2.7	2	N50.25656	E14.54411	177	CZE
UP0284	U0820	subsp. *dioica*	LR	0.559	4	1.5	2.7	5	N50.44975	E14.16000	166	CZE

ID number of population	ID number of analysis	Taxon	Collector	Relative fluorescence intensi	DNA-ploidy level	CV of standard	CV of sample	N. of individuals in analysis	Latitude (WGS-84)	Longitude (WGS-84)	Altitude (m a.s.l.)	Country (ISO 3166-1)
	U0821	subsp. *dioica*	LR	0.559	4	1.5	2.7	5	N50.44975	E14.16000	166	CZE
	U0822	subsp. *dioica*	LR	0.559	4	1.5	2.7	5	N50.44975	E14.16000	166	CZE
	U0823	subsp. *dioica*	LR	0.559	4	1.5	2.7	5	N50.44975	E14.16000	166	CZE
	U0824	subsp. *dioica*	LR	0.559	4	1.5	2.7	5	N50.44975	E14.16000	166	CZE
UP0285	U0825	subsp. *dioica*	LR	0.568	4	1.3	1.9	2	N49.26083	E13.94356	387	CZE
	U0826	subsp. *dioica*	LR	0.568	4	1.3	1.9	2	N49.26083	E13.94356	387	CZE
UP0286	U0827	subsp. *dioica*	LR	0.565	4	1.3	2.7	1	N49.26069	E13.94258	388	CZE
	U0828	subsp. *dioica*	LR	0.565	4	1.3	2.7	1	N49.26069	E13.94258	388	CZE
	U0829	subsp. *dioica*	LR	0.565	4	1.3	2.7	1	N49.26069	E13.94258	388	CZE
	U0830	subsp. *dioica*	LR	0.565	4	1.3	2.7	1	N49.26069	E13.94258	388	CZE
UP0287	U0831	subsp. *dioica*	LR	0.560	4	1.6	2.3	1	N50.42717	E14.14592	164	CZE
	U0832	subsp. *subinermis*	LR	0.291	2	2.2	3.4	1	N50.42717	E14.14592	164	CZE
UP0288	U0833	subsp. *dioica*	LR	0.552	4	1.6	2.7	4	N50.18044	E14.78839	190	CZE
	U0834	subsp. *dioica*	LR	0.552	4	1.6	2.7	4	N50.18044	E14.78839	190	CZE
	U0835	subsp. *dioica*	LR	0.552	4	1.6	2.7	4	N50.18044	E14.78839	190	CZE
	U0836	subsp. *dioica*	LR	0.552	4	1.6	2.7	4	N50.18044	E14.78839	190	CZE
UP0289	U0837	subsp. *dioica*	LR	0.568	4	1.3	2.2	5	N49.25442	E13.55300	462	CZE
	U0838	subsp. *dioica*	LR	0.568	4	1.3	2.2	5	N49.25442	E13.55300	462	CZE
	U0839	subsp. *dioica*	LR	0.568	4	1.3	2.2	5	N49.25442	E13.55300	462	CZE
	U0840	subsp. *dioica*	LR	0.568	4	1.3	2.2	5	N49.25442	E13.55300	462	CZE
	U0841	subsp. *dioica*	LR	0.568	4	1.3	2.2	5	N49.25442	E13.55300	462	CZE
UP0290	U0842	subsp. *dioica*	LR	0.570	4	1.7	2.3	3	N49.32131	E14.70600	404	CZE
	U0843	subsp. *dioica*	LR	0.570	4	1.7	2.3	3	N49.32131	E14.70600	404	CZE

ID number of population	ID number of analysis	Taxon	Collector	Relative fluorescence intensi	DNA-ploidy level	CV of standard	CV of sample	N. of individuals in analysis	Latitude (WGS-84)	Longitude (WGS-84)	Altitude (m a.s.l.)	Country (ISO 3166-1)
	U0844	subsp. *dioica*	LR	0.570	4	1.7	2.3	3	N49.32131	E14.70600	404	CZE
UP0291	U0845	subsp. *dioica*	LR	0.568	4	1.9	2.4	1	N50.44781	E14.16028	157	CZE
UP0292	U0846	subsp. *dioica*	LR	0.558	4	1.4	2.9	3	N49.21211	E13.50508	498	CZE
	U0847	subsp. *dioica*	LR	0.558	4	1.4	2.9	3	N49.21211	E13.50508	498	CZE
	U0848	subsp. *dioica*	LR	0.558	4	1.4	2.9	3	N49.21211	E13.50508	498	CZE
UP0293	U0849	subsp. *dioica*	HJ	0.574	4	1.8	2.7	6	N50.62975	E13.85302	192	CZE
	U0850	subsp. *dioica*	HJ	0.574	4	1.8	2.7	6	N50.62975	E13.85302	192	CZE
	U0851	subsp. *dioica*	HJ	0.574	4	1.8	2.7	6	N50.62975	E13.85302	192	CZE
	U0852	subsp. *dioica*	HJ	0.574	4	1.8	2.7	6	N50.62975	E13.85302	192	CZE
	U0853	subsp. *dioica*	HJ	0.574	4	1.8	2.7	6	N50.62975	E13.85302	192	CZE
	U0854	subsp. *dioica*	HJ	0.574	4	1.8	2.7	6	N50.62975	E13.85302	192	CZE
	U0877	subsp. *dioica*	HJ	0.576	4	1.4	2.3	5	N50.62975	E13.85302	192	CZE
	U0878	subsp. *dioica*	HJ	0.576	4	1.4	2.3	5	N50.62975	E13.85302	192	CZE
	U0879	subsp. *dioica*	HJ	0.576	4	1.4	2.3	5	N50.62975	E13.85302	192	CZE
	U0880	subsp. *dioica*	HJ	0.576	4	1.4	2.3	5	N50.62975	E13.85302	192	CZE
	U0881	subsp. *dioica*	HJ	0.576	4	1.4	2.3	5	N50.62975	E13.85302	192	CZE
	U0882	subsp. *dioica*	HJ	0.563	4	1.8	1.6	5	N50.62975	E13.85302	192	CZE
	U0883	subsp. *dioica*	HJ	0.563	4	1.8	1.6	5	N50.62975	E13.85302	192	CZE
	U0884	subsp. *dioica*	HJ	0.563	4	1.8	1.6	5	N50.62975	E13.85302	192	CZE
	U0885	subsp. *dioica*	HJ	0.563	4	1.8	1.6	5	N50.62975	E13.85302	192	CZE
	U0886	subsp. *dioica*	HJ	0.563	4	1.8	1.6	5	N50.62975	E13.85302	192	CZE
	U0977	subsp. *dioica*	HJ	0.574	4	1.8	2.7	5	N50.62975	E13.85302	192	CZE
	U0978	subsp. *dioica*	HJ	0.574	4	1.8	2.7	5	N50.62975	E13.85302	192	CZE

ID number of population	ID number of analysis	Taxon	Collector	Relative fluorescence intensi	DNA-ploidy level	CV of standard	CV of sample	N. of individuals in analysis	Latitude (WGS-84)	Longitude (WGS-84)	Altitude (m a.s.l.)	Country (ISO 3166-1)
	U0979	subsp. *dioica*	HJ	0.574	4	1.8	2.7	5	N50.62975	E13.85302	192	CZE
	U0980	subsp. *dioica*	HJ	0.574	4	1.8	2.7	5	N50.62975	E13.85302	192	CZE
	U0981	subsp. *dioica*	HJ	0.574	4	1.8	2.7	5	N50.62975	E13.85302	192	CZE
UP0294	U0855	subsp. *dioica*	HJ	0.568	4	1.4	1.2	5	N50.50097	E14.04775	157	CZE
	U0856	subsp. *dioica*	HJ	0.568	4	1.4	1.2	5	N50.50097	E14.04775	157	CZE
	U0857	subsp. *dioica*	HJ	0.568	4	1.4	1.2	5	N50.50097	E14.04775	157	CZE
	U0858	subsp. *dioica*	HJ	0.568	4	1.4	1.2	5	N50.50097	E14.04775	157	CZE
	U0859	subsp. *dioica*	HJ	0.568	4	1.4	1.2	5	N50.50097	E14.04775	157	CZE
	U0864	subsp. *dioica*	HJ	0.582	4	1.7	2.9	5	N50.50097	E14.04775	157	CZE
	U0865	subsp. *dioica*	HJ	0.582	4	1.7	2.9	5	N50.50097	E14.04775	157	CZE
	U0866	subsp. *dioica*	HJ	0.582	4	1.7	2.9	5	N50.50097	E14.04775	157	CZE
	U0867	subsp. *dioica*	HJ	0.582	4	1.7	2.9	5	N50.50097	E14.04775	157	CZE
	U0868	subsp. *dioica*	HJ	0.582	4	1.7	2.9	5	N50.50097	E14.04775	157	CZE
UP0295	U0860	subsp. *dioica*	LR	0.573	4	1.7	2.2	4	N50.19272	E14.67150	171	CZE
	U0861	subsp. *dioica*	LR	0.573	4	1.7	2.2	4	N50.19272	E14.67150	171	CZE
	U0862	subsp. *dioica*	LR	0.573	4	1.7	2.2	4	N50.19272	E14.67150	171	CZE
	U0863	subsp. *dioica*	LR	0.573	4	1.7	2.2	4	N50.19272	E14.67150	171	CZE
UP0296	U0869	subsp. *dioica*	LR	0.567	4	1.7	2.1	3	N50.87577	E14.23508	133	DEU
	U0870	subsp. *dioica*	LR	0.567	4	1.7	2.1	3	N50.87577	E14.23508	133	DEU
	U0871	subsp. *dioica*	LR	0.567	4	1.7	2.1	3	N50.87577	E14.23508	133	DEU
	U0872	subsp. *dioica*	LR	0.580	4	1.3	2.1	3	N50.87577	E14.23508	133	DEU
	U0873	subsp. *dioica*	LR	0.580	4	1.3	2.1	3	N50.87577	E14.23508	133	DEU
	U0874	subsp. *dioica*	LR	0.580	4	1.3	2.1	3	N50.87577	E14.23508	133	DEU

ID number of population	ID number of analysis	Taxon	Collector	Relative fluorescence intensi	DNA-ploidy level	CV of standard	CV of sample	N. of individuals in analysis	Latitude (WGS-84)	Longitude (WGS-84)	Altitude (m a.s.l.)	Country (ISO 3166-1)
UP0297	U0875	subsp. *dioica*	RB	0.563	4	1.7	2.2	2	N49.93755	E14.30574	201	CZE
	U0876	subsp. *dioica*	RB	0.563	4	1.7	2.2	2	N49.93755	E14.30574	201	CZE
UP0298	U0887	subsp. *dioica*	JR	0.558	4	1.2	1.9	1	N50.11364	E15.16389	198	CZE
UP0299	U0888	subsp. *dioica*	JR	0.560	4	1.4	1.8	1	N50.10243	E15.17509	199	CZE
UP0300	U0889	subsp. *dioica*	JR	0.554	4	1.7	2.2	1	N50.10589	E15.18225	195	CZE
UP0301	U0890	subsp. *dioica*	JR	0.563	4	1.5	1.7	1	N50.09537	E15.17349	194	CZE
UP0302	U0891	subsp. *dioica*	LR	0.560	4	1.4	2.2	4	N50.44778	E14.16078	158	CZE
	U0892	subsp. *dioica*	LR	0.560	4	1.4	2.2	4	N50.44778	E14.16078	158	CZE
	U0893	subsp. *dioica*	LR	0.560	4	1.4	2.2	4	N50.44778	E14.16078	158	CZE
	U0894	subsp. *dioica*	LR	0.560	4	1.4	2.2	4	N50.44778	E14.16078	158	CZE
UP0303	U0895	subsp. *dioica*	LR	0.578	4	1.1	1.8	2	N50.19189	E14.67094	172	CZE
	U0896	subsp. *dioica*	LR	0.578	4	1.1	1.8	2	N50.19189	E14.67094	172	CZE
UP0304	U0897	subsp. *dioica*	LR	0.567	4	1.5	1.7	1	N49.81213	E14.32397	287	CZE
	U0898	subsp. *dioica*	LR	0.578	4	1.8	1.9	1	N49.81213	E14.32397	287	CZE
	U0899	subsp. *dioica*	LR	0.570	4	1.5	2.0	1	N49.81213	E14.32397	287	CZE
	U0900	subsp. *dioica*	LR	0.571	4	1.5	2.3	1	N49.81213	E14.32397	287	CZE
UP0305	U0901	subsp. *dioica*	LR	0.556	4	1.3	1.8	2	N50.44950	E14.16275	161	CZE
	U0902	subsp. *dioica*	LR	0.556	4	1.3	1.8	2	N50.44950	E14.16275	161	CZE
UP0306	U0903	subsp. *dioica*	LR	0.564	4	1.9	2.4	1	N50.45200	E14.16481	169	CZE
UP0307	U0904	subsp. *dioica*	LR	0.573	4	1.5	2.4	4	N49.28392	E14.69950	400	CZE
	U0905	subsp. *dioica*	LR	0.573	4	1.5	2.4	4	N49.28392	E14.69950	400	CZE
	U0906	subsp. *dioica*	LR	0.573	4	1.5	2.4	4	N49.28392	E14.69950	400	CZE
	U0907	subsp. *dioica*	LR	0.573	4	1.5	2.4	4	N49.28392	E14.69950	400	CZE

ID number of population	ID number of analysis	Taxon	Collector	Relative fluorescence intensi	DNA-ploidy level	CV of standard	CV of sample	N. of individuals in analysis	Latitude (WGS-84)	Longitude (WGS-84)	Altitude (m a.s.l.)	Country (ISO 3166-1)
UP0308	U0914	subsp. *dioica*	LR	0.554	4	1.4	1.9	7	N49.21125	E13.50250	489	CZE
	U0915	subsp. *dioica*	LR	0.554	4	1.4	1.9	7	N49.21125	E13.50250	489	CZE
	U0916	subsp. *dioica*	LR	0.554	4	1.4	1.9	7	N49.21125	E13.50250	489	CZE
	U0917	subsp. *dioica*	LR	0.554	4	1.4	1.9	7	N49.21125	E13.50250	489	CZE
	U0918	subsp. *dioica*	LR	0.554	4	1.4	1.9	7	N49.21125	E13.50250	489	CZE
	U0919	subsp. *dioica*	LR	0.554	4	1.4	1.9	7	N49.21125	E13.50250	489	CZE
	U0920	subsp. *dioica*	LR	0.554	4	1.4	1.9	7	N49.21125	E13.50250	489	CZE
UP0309	U0921	subsp. *dioica*	LR	0.573	4	1.2	1.9	3	N50.42561	E14.14122	166	CZE
	U0922	subsp. *dioica*	LR	0.573	4	1.2	1.9	3	N50.42561	E14.14122	166	CZE
	U0923	subsp. *dioica*	LR	0.573	4	1.2	1.9	3	N50.42561	E14.14122	166	CZE
UP0310	U0924	subsp. *dioica*	LR	0.562	4	1.3	2.0	2	N50.44917	E14.16214	162	CZE
	U0925	subsp. *dioica*	LR	0.562	4	1.3	2.0	2	N50.44917	E14.16214	162	CZE
UP0311	U0926	subsp. *dioica*	LR	0.565	4	1.6	2.3	3	N50.32417	E14.47469	162	CZE
	U0927	subsp. *dioica*	LR	0.565	4	1.6	2.3	3	N50.32417	E14.47469	162	CZE
	U0928	subsp. *dioica*	LR	0.565	4	1.6	2.3	3	N50.32417	E14.47469	162	CZE
UP0312	U0929	subsp. *dioica*	LR	0.564	4	1.7	2.6	5	N49.81673	E14.33176	281	CZE
	U0930	subsp. *dioica*	LR	0.564	4	1.7	2.6	5	N49.81673	E14.33176	281	CZE
	U0931	subsp. *dioica*	LR	0.564	4	1.7	2.6	5	N49.81673	E14.33176	281	CZE
	U0932	subsp. *dioica*	LR	0.564	4	1.7	2.6	5	N49.81673	E14.33176	281	CZE
	U0933	subsp. *dioica*	LR	0.564	4	1.7	2.6	5	N49.81673	E14.33176	281	CZE
UP0313	U0934	subsp. *dioica*	LR	0.565	4	1.5	2.4	3	N50.44903	E14.16117	163	CZE
	U0935	subsp. *dioica*	LR	0.565	4	1.5	2.4	3	N50.44903	E14.16117	163	CZE
	U0936	subsp. *dioica*	LR	0.565	4	1.5	2.4	3	N50.44903	E14.16117	163	CZE

ID number of population	ID number of analysis	Taxon	Collector	Relative fluorescence intensi	DNA-ploidy level	CV of standard	CV of sample	N. of individuals in analysis	Latitude (WGS-84)	Longitude (WGS-84)	Altitude (m a.s.l.)	Country (ISO 3166-1)
UP0314	U0937	subsp. *dioica*	LR	0.576	4	1.2	2.0	3	N50.44781	E14.16047	157	CZE
	U0938	subsp. *dioica*	LR	0.576	4	1.2	2.0	3	N50.44781	E14.16047	157	CZE
	U0939	subsp. *dioica*	LR	0.576	4	1.2	2.0	3	N50.44781	E14.16047	157	CZE
UP0315	U0940	subsp. *dioica*	LR	0.564	4	1.4	1.9	3	N50.45169	E14.16017	161	CZE
	U0941	subsp. *dioica*	LR	0.564	4	1.4	1.9	3	N50.45169	E14.16017	161	CZE
	U0942	subsp. *dioica*	LR	0.564	4	1.4	1.9	3	N50.45169	E14.16017	161	CZE
UP0316	U0943	subsp. *dioica*	LR	0.575	4	1.6	1.9	3	N50.45094	E14.16150	160	CZE
	U0944	subsp. *dioica*	LR	0.575	4	1.6	1.9	3	N50.45094	E14.16150	160	CZE
	U0945	subsp. *dioica*	LR	0.575	4	1.6	1.9	3	N50.45094	E14.16150	160	CZE
UP0317	U0946	subsp. *dioica*	LR	0.563	4	1.4	2.7	3	N50.45022	E14.16006	167	CZE
	U0947	subsp. *dioica*	LR	0.563	4	1.4	2.7	3	N50.45022	E14.16006	167	CZE
	U0948	subsp. *dioica*	LR	0.563	4	1.4	2.7	3	N50.45022	E14.16006	167	CZE
UP0318	U0949	subsp. *dioica*	LR	0.569	4	1.3	1.9	3	N49.21219	E13.50722	500	CZE
	U0950	subsp. *dioica*	LR	0.569	4	1.3	1.9	3	N49.21219	E13.50722	500	CZE
	U0951	subsp. *dioica*	LR	0.569	4	1.3	1.9	3	N49.21219	E13.50722	500	CZE
UP0319	U0952	subsp. *dioica*	LR	0.569	4	1.2	1.8	2	N49.32156	E14.70328	402	CZE
	U0953	subsp. *dioica*	LR	0.569	4	1.2	1.8	2	N49.32156	E14.70328	402	CZE
UP0320	U0954	subsp. *dioica*	LR	0.570	4	1.5	1.9	3	N49.03808	E14.80625	429	CZE
	U0955	subsp. *dioica*	LR	0.570	4	1.5	1.9	3	N49.03808	E14.80625	429	CZE
	U0956	subsp. *dioica*	LR	0.570	4	1.5	1.9	3	N49.03808	E14.80625	429	CZE
UP0321	U0957	subsp. *dioica*	LR	0.561	4	1.3	2.4	5	N49.82140	E14.33470	269	CZE
	U0958	subsp. *dioica*	LR	0.561	4	1.3	2.4	5	N49.82140	E14.33470	269	CZE
	U0959	subsp. *dioica*	LR	0.561	4	1.3	2.4	5	N49.82140	E14.33470	269	CZE

ID number of population	ID number of analysis	Taxon	Collector	Relative fluorescence intensi	DNA-ploidy level	CV of standard	CV of sample	N. of individuals in analysis	Latitude (WGS-84)	Longitude (WGS-84)	Altitude (m a.s.l.)	Country (ISO 3166-1)
	U0960	subsp. *dioica*	LR	0.561	4	1.3	2.4	5	N49.82140	E14.33470	269	CZE
	U0961	subsp. *dioica*	LR	0.561	4	1.3	2.4	5	N49.82140	E14.33470	269	CZE
UP0322	U0962	subsp. *dioica*	LR	0.562	4	1.6	2.4	3	N50.25522	E14.54422	181	CZE
	U0963	subsp. *dioica*	LR	0.562	4	1.6	2.4	3	N50.25522	E14.54422	181	CZE
	U0964	subsp. *dioica*	LR	0.562	4	1.6	2.4	3	N50.25522	E14.54422	181	CZE
UP0323	U0965	subsp. *dioica*	LR	0.563	4	1.3	2.9	1	N50.42658	E14.14703	160	CZE
UP0324	U0969	subsp. *dioica*	LR	0.561	4	1.7	2.2	4	N49.21208	E13.50733	502	CZE
	U0970	subsp. *dioica*	LR	0.561	4	1.7	2.2	4	N49.21208	E13.50733	502	CZE
	U0971	subsp. *dioica*	LR	0.561	4	1.7	2.2	4	N49.21208	E13.50733	502	CZE
	U0972	subsp. *dioica*	LR	0.561	4	1.7	2.2	4	N49.21208	E13.50733	502	CZE
UP0325	U0973	subsp. *dioica*	IR	0.586	4	1.6	1.4	1	N48.90803	E15.82213	397	CZE
	U0974	subsp. *dioica*	IR	0.565	4	1.2	1.8	3	N48.90803	E15.82213	397	CZE
	U0975	subsp. *dioica*	IR	0.565	4	1.2	1.8	3	N48.90803	E15.82213	397	CZE
	U0976	subsp. *dioica*	IR	0.565	4	1.2	1.8	3	N48.90803	E15.82213	397	CZE
UP0326	U0982	subsp. *dioica*	LR	0.575	4	1.2	2.2	2	N50.44767	E14.16128	158	CZE
	U0983	subsp. *dioica*	LR	0.575	4	1.2	2.2	2	N50.44767	E14.16128	158	CZE
UP0327	U0990	subsp. *dioica*	LR	0.570	4	1.5	1.2	1	N50.39678	E14.08764	168	CZE
	U0991	subsp. *dioica*	LR	0.572	4	2.6	2.9	1	N50.39678	E14.08764	168	CZE
	U0992	subsp. *subinermis*	LR	0.289	2	1.5	2.8	1	N50.39678	E14.08764	168	CZE
UP0328	U0993	subsp. *dioica*	LR	0.565	4	1.8	2.4	4	N50.19267	E14.67136	171	CZE
	U0994	subsp. *dioica*	LR	0.565	4	1.8	2.4	4	N50.19267	E14.67136	171	CZE
	U0995	subsp. *dioica*	LR	0.565	4	1.8	2.4	4	N50.19267	E14.67136	171	CZE
	U0996	subsp. *dioica*	LR	0.565	4	1.8	2.4	4	N50.19267	E14.67136	171	CZE

ID number of population	ID number of analysis	Taxon	Collector	Relative fluorescence intensi	DNA-ploidy level	CV of standard	CV of sample	N. of individuals in analysis	Latitude (WGS-84)	Longitude (WGS-84)	Altitude (m a.s.l.)	Country (ISO 3166-1)
UP0329	U0997	subsp. *dioica*	TU	0.558	4	1.4	2.0	2	N50.13325	E14.39417	178	CZE
	U0998	subsp. *dioica*	TU	0.558	4	1.4	2.0	2	N50.13325	E14.39417	178	CZE
UP0330	U1001	subsp. *dioica*	LR	0.596	4	1.2	2.7	1	N49.43979	E14.19153	354	CZE
UP0331	U1002	subsp. *dioica*	LR	0.595	4	1.3	2.1	1	N49.43874	E14.19307	354	CZE
UP0332	U1003	subsp. *dioica*	LR	0.604	4	1.3	4.6	1	N49.43776	E14.19257	354	CZE
UP0333	U1006	subsp. *dioica*	LR	0.591	4	0.9	4.1	1	N49.43946	E14.19011	354	CZE
UP0334	U1009	subsp. *dioica*	FK	0.567	4	0.8	1.1	1	N52.47448	E13.26699	38	DEU
UP0335	U1014	subsp. *dioica*	LR	0.587	4	0.9	1.1	2	N45.07807	E13.64188	17	HRV
	U1015	subsp. *dioica*	LR	0.580	4	1.1	1.3	2	N45.07807	E13.64188	17	HRV
UP0336	U1017	subsp. *dioica*	LR	0.576	4	0.7	1.0	5	N45.07626	E13.64170	20	HRV
UP0337	U1018	subsp. *dioica*	FK	0.593	4	1.4	1.8	1	N41.83395	E43.32967	600	GEO
	U2896	subsp. *dioica*	FK	0.571	4	1.0	2.3	1	N41.83395	E43.32967	934	GEO
UP0338	U1019	subsp. *dioica*	RB	0.562	4	1.3	1.6	3	N48.88340	E17.07230	180	CZE
	U1020	subsp. *dioica*	RB	0.562	4	1.3	1.6	3	N48.88340	E17.07230	180	CZE
	U1021	subsp. *dioica*	RB	0.562	4	1.3	1.6	3	N48.88340	E17.07230	180	CZE
UP0339	U1022	subsp. *dioica*	RB	0.565	4	1.2	2.0	1	N48.88697	E17.06596	172	CZE
	U1023	subsp. *dioica*	RB	0.573	4	1.5	1.6	1	N48.88697	E17.06596	172	CZE
	U1024	subsp. *dioica*	RB	0.578	4	1.3	1.7	1	N48.88697	E17.06596	172	CZE
	U1025	subsp. *dioica*	RB	0.558	4	1.4	2.1	1	N48.88697	E17.06596	172	CZE
UP0340	U1026	subsp. *subinermis*	RB	0.289	2	1.4	1.9	1	N48.88807	E17.07454	175	CZE
	U1027	subsp. *subinermis*	RB	0.291	2	1.4	1.9	1	N48.88807	E17.07454	175	CZE
UP0341	U1028	subsp. *dioica*	RB	0.559	4	1.3	2.2	4	N48.88909	E17.07427	179	CZE
	U1029	subsp. *dioica*	RB	0.559	4	1.3	2.2	4	N48.88909	E17.07427	179	CZE

ID number of population	ID number of analysis	Taxon	Collector	Relative fluorescence intensi	DNA-ploidy level	CV of standard	CV of sample	N. of individuals in analysis	Latitude (WGS-84)	Longitude (WGS-84)	Altitude (m a.s.l.)	Country (ISO 3166-1)
	U1030	subsp. *dioica*	RB	0.559	4	1.3	2.2	4	N48.88909	E17.07427	179	CZE
	U1031	subsp. *dioica*	RB	0.559	4	1.3	2.2	4	N48.88909	E17.07427	179	CZE
UP0342	U1032	subsp. *dioica*	RB	0.558	4	1.2	1.8	1	N48.88961	E17.07668	180	CZE
	U1033	subsp. *dioica*	RB	0.564	4	1.2	1.7	1	N48.88961	E17.07668	180	CZE
	U1034	subsp. *dioica*	RB	0.569	4	1.6	2.2	1	N48.88961	E17.07668	180	CZE
	U1035	subsp. *dioica*	RB	0.563	4	0.9	1.8	1	N48.88961	E17.07668	180	CZE
UP0343	U1042	subsp. *dioica*	LR	0.567	4	1.3	2.3	3	N50.17750	E14.92461	185	CZE
	U1043	subsp. *dioica*	LR	0.567	4	1.3	2.3	3	N50.17750	E14.92461	185	CZE
	U1044	subsp. *dioica*	LR	0.567	4	1.3	2.3	3	N50.17750	E14.92461	185	CZE
UP0344	U1045	subsp. *dioica*	LR	0.567	4	1.3	2.0	4	N50.17489	E14.92442	188	CZE
	U1046	subsp. *dioica*	LR	0.567	4	1.3	2.0	4	N50.17489	E14.92442	188	CZE
	U1047	subsp. *dioica*	LR	0.567	4	1.3	2.0	4	N50.17489	E14.92442	188	CZE
	U1048	subsp. *dioica*	LR	0.567	4	1.3	2.0	4	N50.17489	E14.92442	188	CZE
UP0345	U1049	subsp. *dioica*	LR	0.552	4	1.5	2.1	5	N50.17431	E14.92386	187	CZE
	U1050	subsp. *dioica*	LR	0.552	4	1.5	2.1	5	N50.17431	E14.92386	187	CZE
	U1051	subsp. *dioica*	LR	0.552	4	1.5	2.1	5	N50.17431	E14.92386	187	CZE
	U1052	subsp. *dioica*	LR	0.552	4	1.5	2.1	5	N50.17431	E14.92386	187	CZE
	U1053	subsp. *dioica*	LR	0.552	4	1.5	2.1	5	N50.17431	E14.92386	187	CZE
UP0346	U1054	subsp. *dioica*	LR	0.564	4	1.8	2.4	5	N50.17261	E14.92244	186	CZE
	U1055	subsp. *dioica*	LR	0.564	4	1.8	2.4	5	N50.17261	E14.92244	186	CZE
	U1056	subsp. *dioica*	LR	0.564	4	1.8	2.4	5	N50.17261	E14.92244	186	CZE
	U1057	subsp. *dioica*	LR	0.564	4	1.8	2.4	5	N50.17261	E14.92244	186	CZE
	U1058	subsp. *dioica*	LR	0.564	4	1.8	2.4	5	N50.17261	E14.92244	186	CZE

ID number of population	ID number of analysis	Taxon	Collector	Relative fluorescence intensi	DNA-ploidy level	CV of standard	CV of sample	N. of individuals in analysis	Latitude (WGS-84)	Longitude (WGS-84)	Altitude (m a.s.l.)	Country (ISO 3166-1)
UP0347	U1059	subsp. *dioica*	LR	0.571	4	1.9	2.6	5	N50.17172	E14.92242	188	CZE
	U1060	subsp. *dioica*	LR	0.571	4	1.9	2.6	5	N50.17172	E14.92242	188	CZE
	U1061	subsp. *dioica*	LR	0.571	4	1.9	2.6	5	N50.17172	E14.92242	188	CZE
	U1062	subsp. *dioica*	LR	0.571	4	1.9	2.6	5	N50.17172	E14.92242	188	CZE
	U1063	subsp. *dioica*	LR	0.571	4	1.9	2.6	5	N50.17172	E14.92242	188	CZE
UP0348	U1064	subsp. *dioica*	LR	0.562	4	1.2	2.1	3	N50.17131	E14.92033	184	CZE
	U1065	subsp. *dioica*	LR	0.562	4	1.2	2.1	3	N50.17131	E14.92033	184	CZE
	U1066	subsp. *dioica*	LR	0.562	4	1.2	2.1	3	N50.17131	E14.92033	184	CZE
UP0349	U1067	subsp. *dioica*	LR	0.559	4	1.6	2.2	3	N50.17044	E14.91961	186	CZE
	U1068	subsp. *dioica*	LR	0.559	4	1.6	2.2	3	N50.17044	E14.91961	186	CZE
	U1069	subsp. *dioica*	LR	0.559	4	1.6	2.2	3	N50.17044	E14.91961	186	CZE
UP0350	U1070	subsp. *dioica*	LR	0.573	4	1.3	2.6	2	N50.16842	E14.92111	187	CZE
	U1071	subsp. *dioica*	LR	0.573	4	1.3	2.6	2	N50.16842	E14.92111	187	CZE
UP0351	U1072	subsp. *dioica*	LR	0.569	4	1.4	2.2	3	N50.17006	E14.91981	185	CZE
	U1073	subsp. *dioica*	LR	0.569	4	1.4	2.2	3	N50.17006	E14.91981	185	CZE
	U1074	subsp. *dioica*	LR	0.569	4	1.4	2.2	3	N50.17006	E14.91981	185	CZE
UP0352	U1075	subsp. *dioica*	LR	0.563	4	1.2	2.3	3	N50.11425	E15.17731	196	CZE
	U1076	subsp. *dioica*	LR	0.563	4	1.2	2.3	3	N50.11425	E15.17731	196	CZE
	U1077	subsp. *dioica*	LR	0.563	4	1.2	2.3	3	N50.11425	E15.17731	196	CZE
UP0353	U1078	subsp. *dioica*	LR	0.550	4	1.3	2.6	3	N50.11203	E15.17381	202	CZE
	U1079	subsp. *dioica*	LR	0.550	4	1.3	2.6	3	N50.11203	E15.17381	202	CZE
	U1080	subsp. *dioica*	LR	0.550	4	1.3	2.6	3	N50.11203	E15.17381	202	CZE
UP0354	U1081	subsp. *dioica*	LR	0.555	4	1.6	2.3	5	N50.11197	E15.17275	203	CZE

ID number of population	ID number of analysis	Taxon	Collector	Relative fluorescence intensi	DNA-ploidy level	CV of standard	CV of sample	N. of individuals in analysis	Latitude (WGS-84)	Longitude (WGS-84)	Altitude (m a.s.l.)	Country (ISO 3166-1)
	U1082	subsp. *dioica*	LR	0.555	4	1.6	2.3	5	N50.11197	E15.17275	203	CZE
	U1083	subsp. *dioica*	LR	0.555	4	1.6	2.3	5	N50.11197	E15.17275	203	CZE
	U1084	subsp. *dioica*	LR	0.555	4	1.6	2.3	5	N50.11197	E15.17275	203	CZE
	U1085	subsp. *dioica*	LR	0.555	4	1.6	2.3	5	N50.11197	E15.17275	203	CZE
UP0355	U1086	subsp. *dioica*	LR	0.560	4	1.5	2.7	5	N50.11200	E15.17222	204	CZE
	U1087	subsp. *dioica*	LR	0.560	4	1.5	2.7	5	N50.11200	E15.17222	204	CZE
	U1088	subsp. *dioica*	LR	0.560	4	1.5	2.7	5	N50.11200	E15.17222	204	CZE
	U1089	subsp. *dioica*	LR	0.560	4	1.5	2.7	5	N50.11200	E15.17222	204	CZE
	U1090	subsp. *dioica*	LR	0.560	4	1.5	2.7	5	N50.11200	E15.17222	204	CZE
UP0356	U1091	subsp. *dioica*	LR	0.560	4	1.4	2.4	3	N50.11267	E15.16667	200	CZE
	U1092	subsp. *dioica*	LR	0.560	4	1.4	2.4	3	N50.11267	E15.16667	200	CZE
	U1093	subsp. *dioica*	LR	0.560	4	1.4	2.4	3	N50.11267	E15.16667	200	CZE
UP0357	U1094	subsp. *dioica*	LR	0.555	4	1.7	3.4	6	N50.11394	E15.16161	196	CZE
	U1095	subsp. *dioica*	LR	0.555	4	1.7	3.4	6	N50.11394	E15.16161	196	CZE
	U1096	subsp. *dioica*	LR	0.555	4	1.7	3.4	6	N50.11394	E15.16161	196	CZE
	U1097	subsp. *dioica*	LR	0.555	4	1.7	3.4	6	N50.11394	E15.16161	196	CZE
	U1098	subsp. *dioica*	LR	0.555	4	1.7	3.4	6	N50.11394	E15.16161	196	CZE
	U1099	subsp. *dioica*	LR	0.555	4	1.7	3.4	6	N50.11394	E15.16161	196	CZE
UP0358	U1100	subsp. *dioica*	LR	0.562	4	1.3	1.9	3	N50.11094	E15.16533	198	CZE
	U1101	subsp. *dioica*	LR	0.562	4	1.3	1.9	3	N50.11094	E15.16533	198	CZE
	U1102	subsp. *dioica*	LR	0.562	4	1.3	1.9	3	N50.11094	E15.16533	198	CZE
UP0359	U1103	subsp. *dioica*	LR	0.570	4	1.5	1.9	5	N50.10986	E15.16278	198	CZE
	U1104	subsp. *dioica*	LR	0.570	4	1.5	1.9	5	N50.10986	E15.16278	198	CZE

ID number of population	ID number of analysis	Taxon	Collector	Relative fluorescence intensi	DNA-ploidy level	CV of standard	CV of sample	N. of individuals in analysis	Latitude (WGS-84)	Longitude (WGS-84)	Altitude (m a.s.l.)	Country (ISO 3166-1)
	U1105	subsp. *dioica*	LR	0.570	4	1.5	1.9	5	N50.10986	E15.16278	198	CZE
	U1106	subsp. *dioica*	LR	0.570	4	1.5	1.9	5	N50.10986	E15.16278	198	CZE
	U1107	subsp. *dioica*	LR	0.570	4	1.5	1.9	5	N50.10986	E15.16278	198	CZE
UP0360	U1108	subsp. *dioica*	LR	0.568	4	1.3	2.3	3	N50.11133	E15.16658	199	CZE
	U1109	subsp. *dioica*	LR	0.568	4	1.3	2.3	3	N50.11133	E15.16658	199	CZE
	U1110	subsp. *dioica*	LR	0.568	4	1.3	2.3	3	N50.11133	E15.16658	199	CZE
UP0361	U1111	subsp. *dioica*	LR	0.559	4	1.2	2.6	5	N50.11086	E15.17119	201	CZE
	U1112	subsp. *dioica*	LR	0.559	4	1.2	2.6	5	N50.11086	E15.17119	201	CZE
	U1113	subsp. *dioica*	LR	0.559	4	1.2	2.6	5	N50.11086	E15.17119	201	CZE
	U1114	subsp. *dioica*	LR	0.559	4	1.2	2.6	5	N50.11086	E15.17119	201	CZE
	U1115	subsp. *dioica*	LR	0.559	4	1.2	2.6	5	N50.11086	E15.17119	201	CZE
UP0362	U1116	subsp. *dioica*	LR	0.566	4	1.2	1.9	2	N50.11047	E15.17267	200	CZE
	U1117	subsp. *dioica*	LR	0.566	4	1.2	1.9	2	N50.11047	E15.17267	200	CZE
UP0363	U1118	subsp. *dioica*	LR	0.576	4	1.5	2.2	3	N50.07158	E15.16981	202	CZE
	U1119	subsp. *dioica*	LR	0.576	4	1.5	2.2	3	N50.07158	E15.16981	202	CZE
	U1120	subsp. *dioica*	LR	0.576	4	1.5	2.2	3	N50.07158	E15.16981	202	CZE
UP0364	U1121	subsp. *dioica*	LR	0.556	4	1.6	2.2	4	N50.07078	E15.16931	201	CZE
	U1122	subsp. *dioica*	LR	0.556	4	1.6	2.2	4	N50.07078	E15.16931	201	CZE
	U1123	subsp. *dioica*	LR	0.556	4	1.6	2.2	4	N50.07078	E15.16931	201	CZE
	U1124	subsp. *dioica*	LR	0.556	4	1.6	2.2	4	N50.07078	E15.16931	201	CZE
UP0365	U1125	subsp. *dioica*	LR	0.538	4	1.8	2.3	3	N50.07069	E15.16914	202	CZE
	U1126	subsp. *dioica*	LR	0.538	4	1.8	2.3	3	N50.07069	E15.16914	202	CZE
	U1127	subsp. *dioica*	LR	0.538	4	1.8	2.3	3	N50.07069	E15.16914	202	CZE

ID number of population	ID number of analysis	Taxon	Collector	Relative fluorescence intensi	DNA-ploidy level	CV of standard	CV of sample	N. of individuals in analysis	Latitude (WGS-84)	Longitude (WGS-84)	Altitude (m a.s.l.)	Country (ISO 3166-1)
UP0366	U1128	subsp. *dioica*	LR	0.566	4	1.4	2.3	4	N50.07044	E15.16886	202	CZE
	U1129	subsp. *dioica*	LR	0.566	4	1.4	2.3	4	N50.07044	E15.16886	202	CZE
	U1130	subsp. *dioica*	LR	0.566	4	1.4	2.3	4	N50.07044	E15.16886	202	CZE
	U1131	subsp. *dioica*	LR	0.566	4	1.4	2.3	4	N50.07044	E15.16886	202	CZE
UP0367	U1132	subsp. *dioica*	LR	0.565	4	1.3	1.8	3	N50.07033	E15.17000	204	CZE
	U1133	subsp. *dioica*	LR	0.565	4	1.3	1.8	3	N50.07033	E15.17000	204	CZE
	U1134	subsp. *dioica*	LR	0.565	4	1.3	1.8	3	N50.07033	E15.17000	204	CZE
UP0368	U1135	subsp. *dioica*	LR	0.578	4	1.3	1.9	3	N50.07142	E15.17064	202	CZE
	U1136	subsp. *dioica*	LR	0.578	4	1.3	1.9	3	N50.07142	E15.17064	202	CZE
	U1137	subsp. *dioica*	LR	0.578	4	1.3	1.9	3	N50.07142	E15.17064	202	CZE
UP0369	U1138	subsp. *dioica*	LR	0.563	4	1.4	2.5	5	N50.02236	E15.32689	200	CZE
	U1139	subsp. *dioica*	LR	0.563	4	1.4	2.5	5	N50.02236	E15.32689	200	CZE
	U1140	subsp. *dioica*	LR	0.563	4	1.4	2.5	5	N50.02236	E15.32689	200	CZE
	U1141	subsp. *dioica*	LR	0.563	4	1.4	2.5	5	N50.02236	E15.32689	200	CZE
	U1142	subsp. *dioica*	LR	0.563	4	1.4	2.5	5	N50.02236	E15.32689	200	CZE
UP0370	U1143	subsp. *dioica*	LR	0.561	4	2.1	2.0	6	N50.02250	E15.32658	201	CZE
	U1144	subsp. *dioica*	LR	0.561	4	2.1	2.0	6	N50.02250	E15.32658	201	CZE
	U1145	subsp. *dioica*	LR	0.561	4	2.1	2.0	6	N50.02250	E15.32658	201	CZE
	U1146	subsp. *dioica*	LR	0.561	4	2.1	2.0	6	N50.02250	E15.32658	201	CZE
	U1147	subsp. *dioica*	LR	0.561	4	2.1	2.0	6	N50.02250	E15.32658	201	CZE
	U1148	subsp. *dioica*	LR	0.561	4	2.1	2.0	6	N50.02250	E15.32658	201	CZE
UP0371	U1149	subsp. *dioica*	LR	0.564	4	1.3	2.4	3	N50.03561	E15.51017	209	CZE
	U1150	subsp. *dioica*	LR	0.564	4	1.3	2.4	3	N50.03561	E15.51017	209	CZE

ID number of population	ID number of analysis	Taxon	Collector	Relative fluorescence intensi	DNA-ploidy level	CV of standard	CV of sample	N. of individuals in analysis	Latitude (WGS-84)	Longitude (WGS-84)	Altitude (m a.s.l.)	Country (ISO 3166-1)
	U1151	subsp. *dioica*	LR	0.564	4	1.3	2.4	3	N50.03561	E15.51017	209	CZE
UP0372	U1152	subsp. *dioica*	LR	0.560	4	1.3	2.5	3	N50.03622	E15.50931	207	CZE
	U1153	subsp. *dioica*	LR	0.560	4	1.3	2.5	3	N50.03622	E15.50931	207	CZE
	U1154	subsp. *dioica*	LR	0.560	4	1.3	2.5	3	N50.03622	E15.50931	207	CZE
UP0373	U1155	subsp. *dioica*	LR	0.551	4	1.3	2.5	3	N50.03639	E15.50922	208	CZE
	U1156	subsp. *dioica*	LR	0.551	4	1.3	2.5	3	N50.03639	E15.50922	208	CZE
	U1157	subsp. *dioica*	LR	0.551	4	1.3	2.5	3	N50.03639	E15.50922	208	CZE
UP0374	U1158	subsp. *dioica*	LR	0.557	4	1.6	2.9	3	N50.03692	E15.50883	207	CZE
	U1159	subsp. *dioica*	LR	0.557	4	1.6	2.9	3	N50.03692	E15.50883	207	CZE
	U1160	subsp. *dioica*	LR	0.557	4	1.6	2.9	3	N50.03692	E15.50883	207	CZE
UP0375	U1161	subsp. *dioica*	LR	0.567	4	1.6	1.8	2	N50.03692	E15.50878	207	CZE
	U1162	subsp. *dioica*	LR	0.567	4	1.6	1.8	2	N50.03692	E15.50878	207	CZE
	U1163	subsp. *dioica*	LR	0.603	4	1.3	2.4	1	N50.03692	E15.50878	207	CZE
	U1164	subsp. *dioica*	LR	0.555	4	1.2	2.4	1	N50.03692	E15.50878	207	CZE
	U3630	subsp. *dioica*	LR	0.554	4	1.8	2.2	2	N50.03692	E15.50878	207	CZE
UP0376	U1165	subsp. *dioica*	LR	0.576	4	1.1	3.5	1	N44.86184	E15.60296	663	HRV
	U1166	subsp. *dioica*	LR	0.591	4	1.0	3.4	1	N44.86184	E15.60296	663	HRV
	U1350	subsp. *dioica*	LR	0.584	4	1.1	2.5	1	N44.86184	E15.60296	663	HRV
	U1351	subsp. *dioica*	LR	0.613	4	1.3	3.0	1	N44.86184	E15.60296	663	HRV
	U1352	subsp. *dioica*	LR	0.577	4	1.0	1.6	1	N44.86184	E15.60296	663	HRV
UP0377	U1167	subsp. *subinermis*	LR	0.304	2	1.1	3.7	1	N45.74481	E16.17747	99	HRV
	U1355	subsp. *subinermis*	LR	0.299	2	1.1	3.5	1	N45.74481	E16.17747	99	HRV
	U1356	subsp. *subinermis*	LR	0.300	2	1.3	4.4	1	N45.74481	E16.17747	99	HRV

ID number of population	ID number of analysis	Taxon	Collector	Relative fluorescence intensi	DNA-ploidy level	CV of standard	CV of sample	N. of individuals in analysis	Latitude (WGS-84)	Longitude (WGS-84)	Altitude (m a.s.l.)	Country (ISO 3166-1)
	U1357	subsp. *subinermis*	LR	0.302	2	1.1	3.2	1	N45.74481	E16.17747	99	HRV
UP0378	U1168	subsp. *subinermis*	LR	0.315	2	0.9	2.4	1	N45.36678	E16.75156	93	HRV
UP0379	U1170	subsp. *subinermis*	LR	0.302	2	1.2	2.5	1	N45.71819	E16.21206	97	HRV
	U1171	subsp. *subinermis*	LR	0.309	2	1.4	2.9	1	N45.71819	E16.21206	97	HRV
UP0380	U1172	subsp. *dioica*	LR	0.563	4	0.9	3.9	1	N45.68881	E16.24772	98	HRV
	U1359	subsp. *dioica*	LR	0.572	4	1.1	1.7	1	N45.68881	E16.24772	98	HRV
	U1360	subsp. *dioica*	LR	0.583	4	1.2	3.3	1	N45.68881	E16.24772	98	HRV
	U1361	subsp. *dioica*	LR	0.574	4	1.9	2.6	1	N45.68881	E16.24772	98	HRV
UP0381	U1173	subsp. *dioica*	LR	0.599	4	1.1	2.3	1	N45.66028	E16.27800	97	HRV
UP0382	U1174	subsp. *dioica*	LR	0.584	4	1.1	2.1	1	N45.63306	E16.22733	98	HRV
	U1363	subsp. *dioica*	LR	0.570	4	1.3	3.4	1	N45.63306	E16.22733	98	HRV
UP0383	U1175	subsp. *subinermis*	LR	0.309	2	1.2	3.7	1	N45.38817	E16.67422	94	HRV
	U1376	subsp. *subinermis*	LR	0.303	2	1.5	3.3	1	N45.38817	E16.67422	94	HRV
UP0384	U1176	subsp. *subinermis*	LR	0.300	2	1.2	3.2	1	N45.36631	E16.69739	98	HRV
	U1377	subsp. *subinermis*	LR	0.299	2	1.2	2.9	1	N45.36631	E16.69739	98	HRV
	U1378	subsp. *subinermis*	LR	0.305	2	1.1	2.4	1	N45.36631	E16.69739	98	HRV
	U1379	subsp. *subinermis*	LR	0.300	2	0.8	4.5	1	N45.36631	E16.69739	98	HRV
	U1380	subsp. *subinermis*	LR	0.304	2	1.2	2.8	1	N45.36631	E16.69739	98	HRV
UP0385	U1177	subsp. *dioica*	LR	0.576	4	1.1	3.4	1	N45.31458	E16.79586	91	HRV
	U1178	subsp. *dioica*	LR	0.570	4	1.3	2.6	1	N45.31458	E16.79586	91	HRV
	U1381	subsp. *dioica*	LR	0.559	4	1.1	3.2	1	N45.31458	E16.79586	91	HRV
	U1382	subsp. *dioica*	LR	0.576	4	1.2	2.4	1	N45.31458	E16.79586	91	HRV
	U3301	subsp. *dioica*	LR	0.582	4	1.3	2.4	1	N45.31458	E16.79586	91	HRV

ID number of population	ID number of analysis	Taxon	Collector	Relative fluorescence intensi	DNA-ploidy level	CV of standard	CV of sample	N. of individuals in analysis	Latitude (WGS-84)	Longitude (WGS-84)	Altitude (m a.s.l.)	Country (ISO 3166-1)
UP0386	U1179	subsp. *dioica*	LR	0.565	4	1.0	2.2	1	N45.31489	E16.79603	93	HRV
	U1383	subsp. *dioica*	LR	0.558	4	1.3	2.3	1	N45.31489	E16.79603	93	HRV
UP0387	U1180	subsp. *dioica*	LR	0.570	4	1.1	3.8	1	N45.33261	E16.87097	108	HRV
	U1181	subsp. *dioica*	LR	0.578	4	0.9	2.3	1	N45.33261	E16.87097	108	HRV
	U1182	subsp. *dioica*	LR	0.578	4	0.8	2.7	1	N45.33261	E16.87097	108	HRV
	U3302	subsp. *dioica*	LR	0.577	4	1.4	2.6	1	N45.33261	E16.87097	108	HRV
UP0388	U1183	subsp. *dioica*	LR	0.599	4	1.4	2.9	1	N45.33269	E16.87342	106	HRV
UP0389	U1184	subsp. *subinermis*	LR	0.296	2	1.1	3.2	1	N45.27072	E16.94061	91	HRV
	U1185	subsp. *subinermis*	LR	0.302	2	1.3	3.7	1	N45.27072	E16.94061	91	HRV
	U1186	subsp. *subinermis*	LR	0.293	2	1.0	3.3	1	N45.27072	E16.94061	91	HRV
UP0390	U1187	subsp. *subinermis*	LR	0.300	2	0.8	3.3	1	N45.24886	E16.94675	90	BIH
	U1385	subsp. *subinermis*	LR	0.298	2	1.1	2.7	1	N45.24886	E16.94675	90	BIH
	U1386	subsp. *subinermis*	LR	0.300	2	1.2	3.7	1	N45.24886	E16.94675	90	BIH
UP0391	U1188	subsp. *dioica*	LR	0.570	4	1.8	2.5	1	N45.11825	E15.58606	243	HRV
UP0392	U1189	subsp. *dioica*	RB	0.572	4	0.7	1.1	5	N45.71882	E13.72987	232	ITA
	U1190	subsp. *dioica*	RB	0.572	4	0.7	1.1	5	N45.71882	E13.72987	232	ITA
	U1191	subsp. *dioica*	RB	0.572	4	0.7	1.1	5	N45.71882	E13.72987	232	ITA
	U1192	subsp. *dioica*	RB	0.572	4	0.7	1.1	5	N45.71882	E13.72987	232	ITA
	U1193	subsp. *dioica*	RB	0.572	4	0.7	1.1	5	N45.71882	E13.72987	232	ITA
UP0393	U1194	subsp. *pubescens*	RB	0.298	2	0.7	1.3	1	N45.87569	E13.43600	17	ITA
	U1195	subsp. *pubescens*	RB	0.292	2	0.7	1.3	1	N45.87569	E13.43600	17	ITA
	U1196	subsp. *pubescens*	RB	0.298	2	0.7	1.1	1	N45.87569	E13.43600	17	ITA
	U1197	subsp. *pubescens*	RB	0.295	2	0.7	1.7	1	N45.87569	E13.43600	17	ITA

ID number of population	ID number of analysis	Taxon	Collector	Relative fluorescence intensi	DNA-ploidy level	CV of standard	CV of sample	N. of individuals in analysis	Latitude (WGS-84)	Longitude (WGS-84)	Altitude (m a.s.l.)	Country (ISO 3166-1)
	U1198	subsp. *pubescens*	RB	0.301	2	0.8	1.5	1	N45.87569	E13.43600	17	ITA
UP0394	U1199	subsp. *pubescens*	LR	0.295	2	1.5	1.1	1	N46.11852	E13.21936	137	ITA
	U1200	subsp. *pubescens*	LR	0.300	2	0.8	1.5	1	N46.11852	E13.21936	137	ITA
	U1201	subsp. *pubescens*	LR	0.296	2	0.7	1.4	1	N46.11852	E13.21936	137	ITA
	U1202	subsp. *pubescens*	LR	0.297	2	0.7	1.3	1	N46.11852	E13.21936	137	ITA
UP0395	U1203	subsp. *pubescens*	LR	0.301	2	0.8	1.6	1	N46.37548	E13.05119	284	ITA
	U1204	subsp. *pubescens*	LR	0.300	2	0.7	1.5	1	N46.37548	E13.05119	284	ITA
	U1205	subsp. *pubescens*	LR	0.295	2	0.8	1.3	1	N46.37548	E13.05119	284	ITA
	U1206	subsp. *pubescens*	LR	0.295	2	0.9	1.5	1	N46.37548	E13.05119	284	ITA
	U1207	subsp. *pubescens*	LR	0.295	2	0.7	1.5	1	N46.37548	E13.05119	284	ITA
UP0396	U1208	subsp. *pubescens*	LR	0.292	2	0.8	1.5	1	N46.39472	E13.22091	324	ITA
	U1209	subsp. *pubescens*	LR	0.302	2	0.6	1.3	1	N46.39472	E13.22091	324	ITA
	U1210	subsp. *pubescens*	LR	0.298	2	0.8	1.3	1	N46.39472	E13.22091	324	ITA
UP0397	U1211	subsp. *dioica*	LR	0.559	4	0.8	1.0	4	N46.54111	E13.67201	613	AUT
	U1212	subsp. *dioica*	LR	0.559	4	0.8	1.0	4	N46.54111	E13.67201	613	AUT
	U1213	subsp. *dioica*	LR	0.559	4	0.8	1.0	4	N46.54111	E13.67201	613	AUT
	U1214	subsp. *dioica*	LR	0.559	4	0.8	1.0	4	N46.54111	E13.67201	613	AUT
UP0398	U1215	subsp. *dioica*	HC	0.586	4	0.7	1.7	1	N50.74795	E0.18942	27	GBR
	U1216	subsp. *dioica*	HC	0.592	4	0.7	1.6	1	N50.74795	E0.18942	27	GBR
	U1217	subsp. *dioica*	HC	0.577	4	0.8	1.7	1	N50.74795	E0.18942	27	GBR
	U1218	subsp. *dioica*	HC	0.578	4	0.8	1.2	1	N50.74795	E0.18942	27	GBR
	U1219	subsp. *dioica*	HC	0.578	4	0.6	1.2	1	N50.74795	E0.18942	27	GBR
UP0399	U1220	subsp. *dioica*	HC	0.622	4	0.9	2.4	1	N50.76942	E0.17448	76	GBR

ID number of population	ID number of analysis	Taxon	Collector	Relative fluorescence intensi	DNA-ploidy level	CV of standard	CV of sample	N. of individuals in analysis	Latitude (WGS-84)	Longitude (WGS-84)	Altitude (m a.s.l.)	Country (ISO 3166-1)
	U1221	subsp. *dioica*	HC	0.586	4	1.2	1.7	1	N50.76942	E0.17448	76	GBR
	U1222	subsp. *dioica*	HC	0.604	4	1.8	2.1	1	N50.76942	E0.17448	76	GBR
	U1223	subsp. *dioica*	HC	0.592	4	0.6	1.8	1	N50.76942	E0.17448	76	GBR
	U1224	subsp. *dioica*	HC	0.580	4	1.5	2.2	1	N50.76942	E0.17448	76	GBR
UP0400	U1225	subsp. *dioica*	HC	0.578	4	1.0	1.8	1	N51.33255	E0.05405	169	GBR
	U1226	subsp. *dioica*	HC	0.584	4	0.8	1.9	1	N51.33255	E0.05405	169	GBR
	U1227	subsp. *dioica*	HC	0.605	4	1.2	2.2	1	N51.33255	E0.05405	169	GBR
	U1228	subsp. *dioica*	HC	0.582	4	1.3	2.3	1	N51.33255	E0.05405	169	GBR
	U1229	subsp. *dioica*	HC	0.582	4	0.9	1.8	1	N51.33255	E0.05405	169	GBR
UP0401	U1231	subsp. *dioica*	HC	0.592	4	0.9	1.3	1	N51.45587	E0.01853	20	GBR
	U1232	subsp. *dioica*	HC	0.606	4	0.9	1.6	1	N51.45587	E0.01853	20	GBR
	U1233	subsp. *dioica*	HC	0.633	4	1.0	1.6	1	N51.45587	E0.01853	20	GBR
	U1234	subsp. *dioica*	HC	0.611	4	0.7	1.6	1	N51.45587	E0.01853	20	GBR
UP0402	U1235	subsp. *subinermis*	BS, KSE	0.305	2	1.0	2.5	1	N61.48951	E23.79006	80	FIN
UP0403	U1236	subsp. *subinermis*	BS, KSE	0.311	2	0.9	3.2	1	N61.49465	E23.79238	119	FIN
UP0404	U1237	subsp. *sondenii*	BS, KSE	0.302	2	0.8	1.7	1	N61.48636	E23.68982	94	FIN
UP0405	U1238	subsp. *sondenii*	BS, KSE	0.308	2	0.8	2.7	1	N61.48420	E23.69830	84	FIN
UP0406	U1239	subsp. *sondenii*	BS, KSE	0.300	2	0.8	2.7	1	N61.46638	E23.73464	83	FIN
UP0407	U1240	subsp. *sondenii*	BS, KSE	0.294	2	1.0	2.3	1	N61.47259	E23.74331	83	FIN
UP0408	U1241	subsp. *subinermis*	BS, KSE	0.304	2	0.7	2.3	1	N61.51436	E23.77772	102	FIN
UP0409	U1242	subsp. *subinermis*	BS, KSE	0.312	2	0.7	3.2	1	N60.84215	E24.59997	98	FIN
UP0410	U1243	subsp. *subinermis*	BS, KSE	0.301	2	1.0	2.3	1	N61.30560	E23.96916	106	FIN
UP0411	U1244	subsp. *sondenii*	BS, KSE	0.301	2	1.0	2.8	1	N68.16568	E14.66502	1	NOR

ID number of population	ID number of analysis	Taxon	Collector	Relative fluorescence intensi	DNA-ploidy level	CV of standard	CV of sample	N. of individuals in analysis	Latitude (WGS-84)	Longitude (WGS-84)	Altitude (m a.s.l.)	Country (ISO 3166-1)
UP0412	U1245	subsp. *sondenii*	BS, KSE	0.302	2	1.0	2.6	1	N68.16739	E14.66380	1	NOR
UP0413	U1246	subsp. *sondenii*	BS, KSE	0.308	2	0.9	2.2	1	N68.03581	E13.34848	9	NOR
UP0414	U1247	subsp. *sondenii*	BS, KSE	0.299	2	1.0	2.9	1	N68.03381	E13.34800	15	NOR
UP0415	U1248	subsp. *sondenii*	BS, KSE	0.316	2	1.0	3.7	1	N68.14789	E14.19803	1	NOR
UP0416	U1249	subsp. *sondenii*	BS, KSE	0.308	2	0.8	2.4	1	N68.15561	E14.21187	1	NOR
UP0417	U1250	subsp. *dioica*	BS, KSE	0.612	4	0.8	2.0	1	N69.01995	E15.12213	1	NOR
UP0418	U1251	subsp. *sondenii*	BS, KSE	0.302	2	0.7	1.9	1	N69.11327	E15.98409	1	NOR
UP0419	U1252	subsp. *sondenii*	BS, KSE	0.303	2	0.9	2.7	1	N67.27269	E14.37982	13	NOR
UP0420	U1253	subsp. *sondenii*	BS, KSE	0.304	2	1.1	3.5	1	N67.27018	E14.33708	1	NOR
UP0421	U1254	subsp. *sondenii*	BS, KSE	0.306	2	0.7	2.4	1	N61.47048	E23.88040	99	FIN
UP0422	U1255	subsp. *sondenii*	BS, KSE	0.306	2	1.9	2.2	1	N61.47315	E23.89520	97	FIN
UP0423	U1256	subsp. *sondenii*	BS, KSE	0.303	2	0.9	2.1	1	N61.50715	E23.61435	129	FIN
UP0424	U1257	subsp. *sondenii*	BS, KSE	0.304	2	0.7	2.3	1	N61.51227	E23.63980	105	FIN
UP0425	U1258	subsp. *subinermis*	BS, KSE	0.302	2	0.9	2.6	1	N61.78543	E25.47773	91	FIN
UP0426	U1259	subsp. *subinermis*	BS, KSE	0.305	2	0.8	2.6	1	N61.79176	E25.47760	131	FIN
UP0427	U1260	subsp. *sondenii*	BS, KSE	0.308	2	0.8	2.8	1	N62.83056	E27.68569	81	FIN
UP0428	U1261	subsp. *sondenii*	BS, KSE	0.308	2	0.8	2.4	1	N62.82746	E27.69895	83	FIN
UP0429	U1268	subsp. *dioica*	LR	0.572	4	0.9	2.4	1	N39.44106	W5.31467	525	ESP
	U1269	subsp. *dioica*	LR	0.602	4	1.7	3.7	1	N39.44106	W5.31467	525	ESP
	U1270	subsp. *dioica*	LR	0.589	4	1.0	1.3	1	N39.44106	W5.31467	525	ESP
	U1271	subsp. *dioica*	LR	0.580	4	1.7	2.0	1	N39.44106	W5.31467	525	ESP
	U1272	subsp. *dioica*	LR	0.580	4	0.7	2.8	1	N39.44106	W5.31467	525	ESP
	U1273	subsp. *dioica*	LR	0.614	4	1.0	3.6	1	N39.44106	W5.31467	525	ESP

ID number of population	ID number of analysis	Taxon	Collector	Relative fluorescence intensi	DNA-ploidy level	CV of standard	CV of sample	N. of individuals in analysis	Latitude (WGS-84)	Longitude (WGS-84)	Altitude (m a.s.l.)	Country (ISO 3166-1)
	U1274	subsp. *dioica*	LR	0.585	4	1.2	3.3	1	N39.44106	W5.31467	525	ESP
UP0430	U1275	subsp. *dioica*	LR	0.593	4	1.4	3.7	1	N40.11544	W5.83850	1221	ESP
	U1277	subsp. *dioica*	LR	0.595	4	1.1	2.6	1	N40.11544	W5.83850	1221	ESP
	U1279	subsp. *dioica*	LR	0.589	4	1.4	2.8	1	N40.11544	W5.83850	1221	ESP
UP0431	U1280	subsp. *dioica*	LR	0.600	4	1.3	4.1	1	N39.64611	W5.81836	354	ESP
	U1281	subsp. *dioica*	LR	0.564	4	1.1	2.3	1	N39.64611	W5.81836	354	ESP
	U1282	subsp. *dioica*	LR	0.603	4	1.6	2.4	1	N39.64611	W5.81836	354	ESP
	U1283	subsp. *dioica*	LR	0.572	4	1.0	2.9	1	N39.64611	W5.81836	354	ESP
	U1284	subsp. *dioica*	LR	0.613	4	1.2	3.2	1	N39.64611	W5.81836	354	ESP
	U1285	subsp. *dioica*	LR	0.583	4	0.9	1.3	1	N39.64611	W5.81836	354	ESP
UP0432	U1296	subsp. *dioica*	LR	0.591	4	1.4	3.2	1	N40.56883	W6.09161	962	ESP
	U1297	subsp. *dioica*	LR	0.596	4	1.4	3.4	1	N40.56883	W6.09161	962	ESP
	U1299	subsp. *dioica* pentaploid	LR	0.771	5	0.8	3.2	1	N40.56883	W6.09161	962	ESP
	U1300	subsp. *dioica*	LR	0.597	4	1.6	2.9	1	N40.56883	W6.09161	962	ESP
	U1301	subsp. *dioica*	LR	0.623	4	1.0	3.7	1	N40.56883	W6.09161	962	ESP
	U1302	subsp. *dioica*	LR	0.589	4	1.2	3.3	1	N40.56883	W6.09161	962	ESP
	U1303	subsp. *dioica*	LR	0.612	4	1.2	3.9	1	N40.56883	W6.09161	962	ESP
	U1304	subsp. *dioica*	LR	0.606	4	0.9	4.3	1	N40.56883	W6.09161	962	ESP
UP0433	U1311	subsp. *dioica*	HC	0.616	4	1.4	2.7	1	N45.72545	E10.84377	2168	ITA
	U1312	subsp. *dioica*	HC	0.604	4	1.3	2.3	1	N45.72545	E10.84377	2168	ITA
	U1313	subsp. *dioica*	HC	0.596	4	1.1	1.7	1	N45.72545	E10.84377	2168	ITA
UP0434	U1314	subsp. *dioica*	HC	0.596	4	1.2	1.8	1	N45.64280	E10.60287	174	ITA
	U1315	subsp. *dioica*	HC	0.610	4	1.1	3.0	1	N45.64280	E10.60287	174	ITA

ID number of population	ID number of analysis	Taxon	Collector	Relative fluorescence intensi	DNA-ploidy level	CV of standard	CV of sample	N. of individuals in analysis	Latitude (WGS-84)	Longitude (WGS-84)	Altitude (m a.s.l.)	Country (ISO 3166-1)
	U1316	subsp. *dioica*	HC	0.607	4	1.0	2.6	1	N45.64280	E10.60287	174	ITA
UP0435	U1317	subsp. *dioica*	FK	0.594	4	1.1	2.9	1	N69.65605	E18.93484	100	NOR
	U1318	subsp. *dioica*	FK	0.592	4	0.9	2.3	1	N69.65605	E18.93484	100	NOR
	U1319	subsp. *sondenii*	FK	0.306	2	1.2	3.3	1	N69.65605	E18.93484	100	NOR
	U1320	subsp. *sondenii*	FK	0.304	2	1.4	3.4	1	N69.65605	E18.93484	100	NOR
UP0436	U1321	subsp. *sondenii*	FK	0.298	2	1.0	2.4	1	N69.67887	E18.89820	1	NOR
	U1322	subsp. *sondenii*	FK	0.325	2	1.2	2.1	1	N69.67887	E18.89820	1	NOR
UP0437	U1328	subsp. *dioica*	LR	0.558	4	1.1	1.9	6	N49.25981	E13.94494	387	CZE
	U1329	subsp. *dioica*	LR	0.558	4	1.1	1.9	6	N49.25981	E13.94494	387	CZE
	U1330	subsp. *dioica*	LR	0.558	4	1.1	1.9	6	N49.25981	E13.94494	387	CZE
	U1331	subsp. *dioica*	LR	0.558	4	1.1	1.9	6	N49.25981	E13.94494	387	CZE
	U1332	subsp. *dioica*	LR	0.558	4	1.1	1.9	6	N49.25981	E13.94494	387	CZE
	U1333	subsp. *dioica*	LR	0.558	4	1.1	1.9	6	N49.25981	E13.94494	387	CZE
UP0438	U1334	subsp. *dioica*	LR	0.607	4	0.9	3.2	1	N48.65858	E16.96608	171	CZE
	U1335	subsp. *dioica*	LR	0.565	4	0.9	2.6	1	N48.65858	E16.96608	171	CZE
UP0439	U1343	subsp. *subinermis*	DR	0.307	2	1.6	2.5	1	N45.38767	E20.20983	84	SRB
	U1344	subsp. *subinermis*	DR	0.310	2	0.8	2.0	1	N45.38767	E20.20983	85	SRB
UP0440	U1345	subsp. *dioica*	DR	0.608	4	1.2	2.1	1	N45.38464	E19.89236	75	SRB
	U1346	subsp. *dioica*	DR	0.601	4	1.2	1.5	1	N45.38464	E19.89236	75	SRB
UP0441	U1347	subsp. *dioica*	DR	0.582	4	1.1	2.8	1	N45.64764	E19.90456	80	SRB
	U1348	subsp. *dioica*	DR	0.581	4	1.4	1.9	1	N45.64764	E19.90456	80	SRB
UP0442	U1353	subsp. *dioica*	LR	0.569	4	1.6	2.7	1	N44.67164	E15.61206	376	HRV
	U1354	subsp. *dioica*	LR	0.600	4	1.6	3.3	1	N44.67164	E15.61206	376	HRV

ID number of population	ID number of analysis	Taxon	Collector	Relative fluorescence intensi	DNA-ploidy level	CV of standard	CV of sample	N. of individuals in analysis	Latitude (WGS-84)	Longitude (WGS-84)	Altitude (m a.s.l.)	Country (ISO 3166-1)
UP0443	U1358	subsp. *dioica*	LR	0.597	4	1.6	2.6	1	N45.74489	E16.17436	101	HRV
UP0444	U1362	subsp. *subinermis*	LR	0.301	2	1.6	3.3	1	N44.88727	E15.60123	603	HRV
	U1364	subsp. *dioica*	LR	0.578	4	1.5	3.4	1	N44.88727	E15.60123	603	HRV
UP0445	U1365	subsp. *dioica*	LR	0.570	4	0.9	2.3	1	N45.63756	E16.28308	99	HRV
	U3300	subsp. *dioica*	LR	0.569	4	1.2	2.6	1	N45.63756	E16.28308	99	HRV
UP0446	U1366	subsp. *dioica*	LR	0.575	4	1.4	1.9	1	N45.44219	E16.47836	96	HRV
	U1367	subsp. *dioica*	LR	0.564	4	1.1	2.5	1	N45.44219	E16.47836	96	HRV
UP0447	U1368	subsp. *subinermis*	LR	0.301	2	1.5	3.9	1	N45.41522	E16.57194	91	HRV
	U1369	subsp. *subinermis*	LR	0.302	2	1.1	2.6	1	N45.41522	E16.57194	91	HRV
	U1370	subsp. *subinermis*	LR	0.308	2	1.3	3.0	1	N45.41522	E16.57194	91	HRV
UP0448	U1371	subsp. *dioica*	LR	0.563	4	1.0	3.5	1	N45.42283	E16.60911	94	HRV
	U1372	subsp. *subinermis*	LR	0.296	2	0.9	3.1	1	N45.42283	E16.60911	94	HRV
	U1373	subsp. *subinermis*	LR	0.293	2	1.9	3.2	1	N45.42283	E16.60911	94	HRV
	U1374	subsp. *dioica*	LR	0.560	4	1.3	2.4	1	N45.42283	E16.60911	94	HRV
UP0449	U1387	subsp. *dioica*	FK	0.575	4	1.3	1.5	4	N48.80190	E12.98233	315	DEU
UP0450	U1406	subsp. *dioica*	LR	0.587	4	1.2	2.9	1	N56.99045	E23.87425	12	LVA
	U1407	subsp. *dioica*	LR	0.566	4	1.9	3.3	1	N56.99045	E23.87425	12	LVA
	U1408	subsp. *dioica*	LR	0.567	4	1.1	1.6	1	N56.99045	E23.87425	12	LVA
UP0451	U1409	subsp. *dioica*	LR	0.607	4	1.6	3.9	1	N56.97942	E23.87247	4	LVA
	U1410	subsp. *dioica*	LR	0.568	4	1.0	1.8	1	N56.97942	E23.87247	4	LVA
	U1411	subsp. *dioica*	LR	0.581	4	1.6	2.3	1	N56.97942	E23.87247	4	LVA
	U1412	subsp. *dioica*	LR	0.635	4	1.7	4.3	1	N56.97942	E23.87247	4	LVA
UP0452	U1413	subsp. *dioica*	LR	0.578	4	1.1	2.3	1	N57.06205	E24.04180	5	LVA

ID number of population	ID number of analysis	Taxon	Collector	Relative fluorescence intensi	DNA-ploidy level	CV of standard	CV of sample	N. of individuals in analysis	Latitude (WGS-84)	Longitude (WGS-84)	Altitude (m a.s.l.)	Country (ISO 3166-1)
	U1414	subsp. *dioica*	LR	0.567	4	2.1	2.9	1	N57.06205	E24.04180	5	LVA
	U1415	subsp. *dioica*	LR	0.580	4	1.0	1.2	1	N57.06205	E24.04180	5	LVA
	U1416	subsp. *dioica*	LR	0.575	4	1.0	3.7	1	N57.06205	E24.04180	5	LVA
UP0453	U1420	subsp. *dioica*	LR	0.566	4	1.2	2.1	1	N57.31392	E25.26653	88	LVA
	U1421	subsp. *dioica*	LR	0.615	4	1.9	3.9	1	N57.31392	E25.26653	88	LVA
	U1422	subsp. *dioica*	LR	0.605	4	1.5	2.6	1	N57.31392	E25.26653	88	LVA
UP0454	U1426	subsp. *dioica*	LR	0.577	4	1.3	3.4	1	N56.48862	E23.37308	71	LVA
	U1427	subsp. *dioica*	LR	0.579	4	1.1	3.2	1	N56.48862	E23.37308	71	LVA
	U1428	subsp. *dioica*	LR	0.579	4	1.6	2.3	1	N56.48862	E23.37308	71	LVA
	U1429	subsp. *dioica*	LR	0.591	4	1.4	3.1	1	N56.48862	E23.37308	71	LVA
UP0455	U1430	subsp. *dioica*	LR	0.577	4	2.1	3.3	1	N56.41448	E24.02425	21	LVA
	U1431	subsp. *dioica*	LR	0.599	4	1.8	4.4	1	N56.41448	E24.02425	21	LVA
UP0456	U1442	subsp. *dioica*	LR	0.607	4	1.7	4.8	1	N57.39730	E21.56268	3	LVA
	U1443	subsp. *dioica*	LR	0.573	4	0.9	1.8	1	N57.39730	E21.56268	3	LVA
	U1444	subsp. *dioica*	LR	0.592	4	1.9	4.3	1	N57.39730	E21.56268	3	LVA
UP0457	U1528	subsp. *dioica*	JPR	0.566	4	1.2	2.6	1	N42.32672	W3.01102	811	ESP
UP0458	U1529	subsp. *subinermis*	JR	0.298	2	1.2	1.8	1	N48.44764	E22.13367	103	SVK
	U1530	subsp. *subinermis*	JR	0.298	2	1.6	1.8	1	N48.44764	E22.13367	103	SVK
	U1531	subsp. *subinermis*	JR	0.294	2	1.5	2.2	1	N48.44764	E22.13367	103	SVK
	U1532	subsp. *subinermis*	JR	0.299	2	1.2	2.2	1	N48.44764	E22.13367	103	SVK
	U1533	subsp. *subinermis*	JR	0.297	2	1.0	2.0	1	N48.44764	E22.13367	103	SVK
	U1534	subsp. *subinermis*	JR	0.298	2	1.0	1.9	1	N48.44764	E22.13367	103	SVK
	U1535	subsp. *subinermis*	JR	0.300	2	1.2	2.1	1	N48.44764	E22.13367	103	SVK

ID number of population	ID number of analysis	Taxon	Collector	Relative fluorescence intensi	DNA-ploidy level	CV of standard	CV of sample	N. of individuals in analysis	Latitude (WGS-84)	Longitude (WGS-84)	Altitude (m a.s.l.)	Country (ISO 3166-1)
	U1536	subsp. *subinermis*	JR	0.300	2	1.1	2.3	1	N48.44764	E22.13367	103	SVK
UP0459	U1537	subsp. *dioica*	JV, PV	0.621	4	1.2	2.9	1	N47.26552	E13.14493	2122	AUT
	U1538	subsp. *dioica*	JV, PV	0.615	4	1.8	3.9	1	N47.26552	E13.14493	2122	AUT
	U1539	subsp. *dioica*	JV, PV	0.584	4	1.7	2.2	1	N47.26552	E13.14493	2122	AUT
	U1540	subsp. *dioica*	JV, PV	0.627	4	2.0	3.0	1	N47.26552	E13.14493	2122	AUT
UP0460	U1541	subsp. *dioica*	AK, KS	0.585	4	1.9	4.2	1	N46.48827	E23.36598	901	ROU
	U1542	subsp. *dioica*	AK, KS	0.584	4	1.3	2.7	1	N46.48827	E23.36598	901	ROU
	U1544	subsp. *dioica*	AK, KS	0.584	4	1.3	3.4	1	N46.48827	E23.36598	901	ROU
UP0461	U1545	subsp. *dioica*	JV, PV	0.584	4	1.6	2.6	1	N45.45233	E25.88427	1463	ROU
	U1547	subsp. *dioica*	JV, PV	0.572	4	1.6	2.4	1	N45.45233	E25.88427	1463	ROU
	U1548	subsp. *dioica*	JV, PV	0.597	4	1.7	2.9	1	N45.45233	E25.88427	1463	ROU
	U1549	subsp. *dioica*	JV, PV	0.570	4	1.1	2.1	1	N45.45233	E25.88427	1463	ROU
UP0462	U1552	subsp. *dioica*	FK	0.580	4	1.6	3.5	1	N54.97653	E12.52559	104	DNK
	U1553	subsp. *dioica*	FK	0.579	4	1.2	2.4	1	N54.97653	E12.52559	104	DNK
	U1554	subsp. *dioica*	FK	0.557	4	1.6	2.7	1	N54.97653	E12.52559	104	DNK
	U1555	subsp. *dioica*	FK	0.622	4	0.9	2.3	1	N54.97653	E12.52559	104	DNK
	U1556	subsp. *dioica*	FK	0.611	4	2.1	2.8	1	N54.97653	E12.52559	104	DNK
	U1557	subsp. *dioica*	FK	0.572	4	1.3	2.6	1	N54.97653	E12.52559	104	DNK
UP0463	U1574	subsp. *dioica*	EZ, FK	0.572	4	1.2	2.4	1	N36.98810	W4.92534	422	ESP
	U1575	subsp. *dioica*	EZ, FK	0.583	4	1.0	2.3	1	N36.98810	W4.92534	422	ESP
	U1576	subsp. *dioica*	EZ, FK	0.613	4	0.8	2.0	1	N36.98810	W4.92534	422	ESP
	U1577	subsp. *dioica*	EZ, FK	0.588	4	1.0	2.5	1	N36.98810	W4.92534	422	ESP
	U1578	subsp. *dioica*	EZ, FK	0.597	4	1.5	3.6	1	N36.98810	W4.92534	422	ESP

ID number of population	ID number of analysis	Taxon	Collector	Relative fluorescence intensi	DNA-ploidy level	CV of standard	CV of sample	N. of individuals in analysis	Latitude (WGS-84)	Longitude (WGS-84)	Altitude (m a.s.l.)	Country (ISO 3166-1)
UP0464	U1582	subsp. *dioica*	KK	0.586	4	1.5	2.8	5	N47.54571	E24.92977	1372	ROU
	U3656	subsp. *dioica*	KK	0.550	4	1.0	1.6	5	N47.54571	E24.92977	1372	ROU
UP0465	U1583	subsp. *dioica*	LR	0.578	4	0.8	1.1	1	N45.08942	E13.88261	311	HRV
	U1584	subsp. *dioica*	LR	0.589	4	0.7	0.9	1	N45.08942	E13.88261	311	HRV
	U1585	subsp. *dioica*	LR	0.566	4	0.8	0.9	1	N45.08942	E13.88261	311	HRV
	U1586	subsp. *dioica*	LR	0.580	4	0.9	1.1	1	N45.08942	E13.88261	311	HRV
	U1587	subsp. *dioica*	LR	0.584	4	0.7	1.0	1	N45.08942	E13.88261	311	HRV
	U1588	subsp. *dioica*	LR	0.604	4	0.7	1.7	1	N45.08942	E13.88261	311	HRV
	U1589	subsp. *dioica*	LR	0.573	4	0.7	0.9	1	N45.08942	E13.88261	311	HRV
	U1590	subsp. *dioica*	LR	0.580	4	1.1	1.2	1	N45.08942	E13.88261	311	HRV
	U1591	subsp. *dioica*	LR	0.577	4	0.8	1.0	1	N45.08942	E13.88261	311	HRV
UP0466	U1592	subsp. *subinermis*	LR	0.318	2	0.9	1.4	1	N48.94444	E16.59044	183	CZE
	U1595	subsp. *subinermis*	LR	0.311	2	0.8	1.7	1	N48.94444	E16.59044	183	CZE
	U3561	subsp. *dioica*	LR	0.550	4	1.2	1.6	5	N48.94444	E16.59044	183	CZE
	U3570	*U. kioviensis*	FK	0.334	2	1.2	2.4	1	N48.94444	E16.59044	183	CZE
	U3571	*U. kioviensis*	FK	0.345	2	1.5	3.3	1	N48.94444	E16.59044	183	CZE
UP0467	U1597	*U. atrovirens*	LR	0.305	2	0.9	2.3	1	N42.13596	E8.60742	69	FRA
UP0468	U1598	subsp. *dioica*	KH	0.574	4	1.2	1.2	2	N45.67561	E7.31292	2008	ITA
UP0469	U1599	subsp. *subinermis*	LR	0.296	2	1.5	2.3	1	N41.74439	E8.87122	72	FRA
UP0470	U1600	subsp. *subinermis*	LR	0.300	2	1.5	2.2	1	N42.30683	E9.15045	435	FRA
UP0471	U1601	subsp. *subinermis*	LR	0.311	2	0.8	2.4	1	N42.38130	E9.15270	337	FRA
	U1602	subsp. *subinermis*	LR	0.301	2	1.8	2.4	1	N42.38130	E9.15270	337	FRA
	U1603	subsp. *subinermis*	LR	0.308	2	1.0	2.9	1	N42.38130	E9.15270	337	FRA

ID number of population	ID number of analysis	Taxon	Collector	Relative fluorescence intensi	DNA-ploidy level	CV of standard	CV of sample	N. of individuals in analysis	Latitude (WGS-84)	Longitude (WGS-84)	Altitude (m a.s.l.)	Country (ISO 3166-1)
	U1604	subsp. *subinermis*	LR	0.295	2	2.6	1.7	1	N42.38130	E9.15270	337	FRA
	U1606	subsp. *subinermis*	LR	0.297	2	0.8	2.0	1	N42.38130	E9.15270	337	FRA
UP0472	U1607	subsp. *dioica*	KH	0.571	4	1.2	1.6	10	N45.07250	E2.71194	1240	FRA
UP0473	U1608	subsp. *dioica*	KH	0.565	4	1.4	1.6	2	N45.18192	E0.72219	92	FRA
UP0474	U1609	subsp. *dioica*	KH	0.571	4	1.4	1.6	3	N47.23167	E6.03194	327	FRA
UP0475	U1610	subsp. *dioica*	KH	0.574	4	1.4	1.6	4	N47.49472	E7.31747	561	FRA
UP0476	U1611	subsp. *dioica*	KH	0.578	4	1.6	1.4	3	N44.75194	E5.59806	1287	FRA
UP0477	U1612	subsp. *dioica*	KH	0.580	4	1.2	1.4	3	N46.61381	E5.85228	567	FRA
UP0478	U1613	subsp. *dioica*	KH	0.570	4	1.7	1.5	3	N44.11408	E7.28572	1486	FRA
UP0479	U1614	subsp. *dioica*	KH	0.580	4	1.3	1.4	2	N46.31075	E6.78419	1230	FRA
UP0480	U1615	subsp. *dioica*	FK	0.569	4	1.2	1.4	5	N40.95158	E20.84589	1474	MKD
UP0481	U1616	subsp. *dioica*	LR	0.595	4	1.0	3.4	1	N49.34185	E14.11678	427	CZE
	U1624	subsp. *dioica*	LR	0.568	4	1.1	4.0	1	N49.34185	E14.11678	427	CZE
	U3555	subsp. *dioica*	LR	0.569	4	1.2	1.8	1	N49.34185	E14.11678	426	CZE
	U3556	subsp. *dioica*	LR	0.559	4	0.7	1.6	1	N49.34185	E14.11678	426	CZE
UP0482	U1617	subsp. *dioica*	LR	0.571	4	0.9	1.9	1	N49.51612	E14.02878	440	CZE
	U1618	subsp. *dioica*	LR	0.569	4	1.0	1.5	1	N49.51612	E14.02878	440	CZE
UP0483	U1619	subsp. *dioica*	LR	0.563	4	0.7	1.6	1	N49.51605	E14.03003	441	CZE
	U1620	subsp. *dioica*	LR	0.567	4	1.2	1.8	1	N49.51605	E14.03003	441	CZE
UP0484	U1623	subsp. *dioica*	LR	0.577	4	0.8	1.8	1	N49.35007	E14.14567	354	CZE
	U1627	subsp. *dioica*	LR	0.575	4	1.2	2.2	1	N49.35007	E14.14567	354	CZE
UP0485	U1625	subsp. *dioica*	LR	0.562	4	0.8	1.6	1	N49.34187	E14.11592	420	CZE
	U3557	subsp. *dioica*	LR	0.565	4	0.6	1.8	1	N49.34187	E14.11592	420	CZE

ID number of population	ID number of analysis	Taxon	Collector	Relative fluorescence intensi	DNA-ploidy level	CV of standard	CV of sample	N. of individuals in analysis	Latitude (WGS-84)	Longitude (WGS-84)	Altitude (m a.s.l.)	Country (ISO 3166-1)
UP0486	U1626	subsp. *dioica*	LR	0.569	4	0.7	1.5	1	N49.51625	E14.02915	441	CZE
UP0487	U1630	subsp. *subinermis*	LR	0.301	2	0.7	1.7	1	N49.35520	E14.14777	356	CZE
	U1631	subsp. *subinermis*	LR	0.304	2	0.7	2.4	1	N49.35520	E14.14777	356	CZE
UP0488	U1633	subsp. *dioica*	FK	0.560	4	0.9	1.4	1	N50.59298	E5.44383	116	BEL
UP0489	U1634	subsp. *dioica*	FK	0.579	4	1.2	1.7	1	N50.42564	E7.27298	351	DEU
UP0490	U1635	subsp. *dioica*	FK	0.571	4	0.9	1.5	5	N50.62814	E8.73034	304	DEU
UP0491	U1636	subsp. *dioica*	FK	0.556	4	1.3	1.6	5	N50.47523	E10.01138	799	DEU
UP0492	U1637	subsp. *dioica*	FK	0.558	4	1.2	1.8	5	N50.24111	E7.56283	120	DEU
UP0493	U1638	subsp. *subinermis*	JR	0.304	2	1.3	2.6	1	N48.47194	E21.80556	96	SVK
	U1639	subsp. *subinermis*	JR	0.299	2	1.7	4.2	1	N48.47194	E21.80556	96	SVK
	U1640	subsp. *dioica* triploid	JR	0.429	3	1.2	2.6	1	N48.47194	E21.80556	96	SVK
	U1641	subsp. *subinermis*	JR	0.290	2	0.9	4.8	1	N48.47194	E21.80556	96	SVK
UP0494	U1643	subsp. *dioica*	LR	0.586	4	0.9	1.9	1	N46.81356	E18.93364	95	HUN
UP0495	U1644	subsp. *dioica*	FK	0.596	4	1.3	3.4	1	N45.58717	E13.72294	54	SVN
UP0496	U1645	subsp. *dioica*	FK	0.595	4	1.5	2.7	1	N47.43408	E13.46494	992	AUT
UP0497	U1650	subsp. *dioica*	FK	0.578	4	0.9	2.4	1	N53.18212	W4.26211	48	GBR
UP0498	U1651	subsp. *dioica*	FK	0.594	4	1.2	2.8	1	N53.23350	W4.11818	3	GBR
UP0499	U1652	subsp. *dioica*	FK	0.573	4	0.8	2.4	1	N56.14456	W4.03230	10	GBR
UP0500	U1653	subsp. *dioica*	LR	0.571	4	1.0	2.8	1	N53.48848	W2.26895	31	GBR
UP0501	U1654	subsp. *dioica*	FK	0.573	4	1.1	2.4	1	N56.03860	E12.62174	10	DNK
UP0502	U1655	subsp. *dioica*	FK	0.582	4	1.2	4.4	1	N60.04434	E10.78176	420	NOR
	U3399	subsp. *dioica*	FK	0.581	4	1.2	1.5	1	N60.04434	E10.78176	420	NOR
UP0503	U1657	subsp. *dioica*	LR	0.593	4	1.7	3.7	1	N61.14747	E8.63622	466	NOR

ID number of population	ID number of analysis	Taxon	Collector	Relative fluorescence intensi	DNA-ploidy level	CV of standard	CV of sample	N. of individuals in analysis	Latitude (WGS-84)	Longitude (WGS-84)	Altitude (m a.s.l.)	Country (ISO 3166-1)
UP0504	U1659	subsp. *dioica*	DH	0.632	4	0.9	4.3	1	N49.32846	E14.20895	475	CZE
UP0505	U1663	subsp. *dioica*	FK	0.593	4	0.8	4.9	1	N42.68226	E22.05947	331	SRB
UP0506	U1664	subsp. *dioica*	FK	0.598	4	1.3	2.9	1	N41.29126	E21.16714	655	MKD
UP0507	U1667	subsp. *dioica*	KSR	0.568	4	1.0	1.3	3	N52.26017	E4.44542	9	NLD
	U1668	subsp. *dioica*	KSR	0.568	4	1.0	1.3	3	N52.26017	E4.44542	9	NLD
	U1669	subsp. *dioica*	KSR	0.568	4	1.0	1.3	3	N52.26017	E4.44542	9	NLD
UP0508	U1687	subsp. *dioica*	LR	0.578	4	1.1	1.4	4	N59.96352	E10.66717	326	NOR
	U1688	subsp. *dioica*	LR	0.578	4	1.1	1.4	4	N59.96352	E10.66717	326	NOR
	U1689	subsp. *dioica*	LR	0.578	4	1.1	1.4	4	N59.96352	E10.66717	326	NOR
	U1690	subsp. *dioica*	LR	0.578	4	1.1	1.4	4	N59.96352	E10.66717	326	NOR
UP0509	U1691	subsp. *dioica*	LR	0.572	4	1.3	1.5	4	N59.90117	E10.76604	44	NOR
	U1692	subsp. *dioica*	LR	0.572	4	1.3	1.5	4	N59.90117	E10.76604	44	NOR
	U1693	subsp. *dioica*	LR	0.572	4	1.3	1.5	4	N59.90117	E10.76604	44	NOR
	U1694	subsp. *dioica*	LR	0.572	4	1.3	1.5	4	N59.90117	E10.76604	44	NOR
UP0510	U1695	subsp. *dioica*	LR	0.571	4	1.4	1.6	6	N59.91865	E10.72833	31	NOR
	U1696	subsp. *dioica*	LR	0.571	4	1.4	1.6	6	N59.91865	E10.72833	31	NOR
	U1697	subsp. *dioica*	LR	0.571	4	1.4	1.6	6	N59.91865	E10.72833	31	NOR
	U1698	subsp. *dioica*	LR	0.571	4	1.4	1.6	6	N59.91865	E10.72833	31	NOR
	U1699	subsp. *dioica*	LR	0.571	4	1.4	1.6	6	N59.91865	E10.72833	31	NOR
	U1700	subsp. *dioica*	LR	0.571	4	1.4	1.6	6	N59.91865	E10.72833	31	NOR
UP0511	U1701	subsp. *dioica*	LR	0.568	4	1.4	1.7	5	N59.89830	E10.72941	9	NOR
	U1702	subsp. *dioica*	LR	0.568	4	1.4	1.7	5	N59.89830	E10.72941	9	NOR
	U1703	subsp. *dioica*	LR	0.568	4	1.4	1.7	5	N59.89830	E10.72941	9	NOR

ID number of population	ID number of analysis	Taxon	Collector	Relative fluorescence intensi	DNA-ploidy level	CV of standard	CV of sample	N. of individuals in analysis	Latitude (WGS-84)	Longitude (WGS-84)	Altitude (m a.s.l.)	Country (ISO 3166-1)
	U1704	subsp. *dioica*	LR	0.568	4	1.4	1.7	5	N59.89830	E10.72941	9	NOR
	U1705	subsp. *dioica*	LR	0.568	4	1.4	1.7	5	N59.89830	E10.72941	9	NOR
UP0512	U1706	subsp. *dioica*	LR	0.578	4	1.2	1.8	4	N59.89426	E10.72516	2	NOR
	U1707	subsp. *dioica*	LR	0.578	4	1.2	1.8	4	N59.89426	E10.72516	2	NOR
	U1708	subsp. *dioica*	LR	0.578	4	1.2	1.8	4	N59.89426	E10.72516	2	NOR
	U1709	subsp. *dioica*	LR	0.578	4	1.2	1.8	4	N59.89426	E10.72516	2	NOR
UP0513	U1715	subsp. *dioica*	IR	0.604	4	1.1	3.2	1	N43.63938	E11.16857	118	ITA
UP0514	U1716	subsp. *dioica*	IR	0.596	4	0.8	1.3	1	N43.69119	E11.20720	107	ITA
UP0515	U1725	subsp. *dioica*	JP	0.578	4	0.9	1.2	1	N35.85223	E52.07535	2370	IRN
UP0516	U1726	subsp. *dioica*	JP	0.578	4	0.8	1.3	1	N36.44307	E51.06507	1821	IRN
UP0517	U1727	subsp. *dioica*	JP	0.576	4	0.8	1.5	1	N36.47182	E51.09903	1569	IRN
UP0518	U1728	subsp. *dioica*	JP	0.566	4	0.9	1.3	1	N37.59830	E48.68333	2072	IRN
UP0519	U1742	subsp. *dioica*	JC, TU	0.565	4	1.1	2.5	1	N40.78980	E39.61565	451	TUR
	U1743	subsp. *dioica*	JC, TU	0.574	4	1.9	1.4	1	N40.78980	E39.61565	451	TUR
	U1744	subsp. *dioica*	JC, TU	0.575	4	0.9	2.6	1	N40.78980	E39.61565	451	TUR
	U1745	subsp. *dioica*	JC, TU	0.566	4	0.9	1.8	1	N40.78980	E39.61565	451	TUR
	U1746	subsp. *dioica*	JC, TU	0.562	4	1.1	1.5	1	N40.78980	E39.61565	451	TUR
UP0520	U1747	subsp. *dioica*	JC, TU	0.559	4	0.7	1.6	1	N40.69418	E39.45818	1380	TUR
	U1748	subsp. *dioica*	JC, TU	0.574	4	1.1	1.8	1	N40.69418	E39.45818	1380	TUR
	U1749	subsp. *dioica*	JC, TU	0.556	4	1.0	1.5	1	N40.69418	E39.45818	1380	TUR
	U1750	subsp. *dioica*	JC, TU	0.560	4	1.1	1.6	1	N40.69418	E39.45818	1380	TUR
UP0521	U1752	subsp. *dioica*	JC, TU	0.580	4	1.3	2.8	1	N40.64180	E39.40345	2060	TUR
	U1753	subsp. *dioica*	JC, TU	0.583	4	1.5	2.4	1	N40.64180	E39.40345	2060	TUR

ID number of population	ID number of analysis	Taxon	Collector	Relative fluorescence intensi	DNA-ploidy level	CV of standard	CV of sample	N. of individuals in analysis	Latitude (WGS-84)	Longitude (WGS-84)	Altitude (m a.s.l.)	Country (ISO 3166-1)
	U1754	subsp. *dioica*	JC, TU	0.576	4	1.0	1.9	1	N40.64180	E39.40345	2060	TUR
	U1755	subsp. *dioica*	JC, TU	0.575	4	0.8	2.1	1	N40.64180	E39.40345	2060	TUR
	U1756	subsp. *dioica*	JC, TU	0.568	4	0.9	1.7	1	N40.64180	E39.40345	2060	TUR
	U1757	subsp. *dioica*	JC, TU	0.587	4	0.8	1.9	1	N40.64180	E39.40345	2060	TUR
UP0522	U1758	subsp. *dioica*	JC, TU	0.578	4	0.8	1.9	1	N40.23224	E39.44778	1710	TUR
	U1759	subsp. *dioica*	JC, TU	0.579	4	0.9	2.3	1	N40.23224	E39.44778	1710	TUR
UP0523	U1761	subsp. *dioica*	JC, TU	0.601	4	0.9	2.6	1	N40.07921	E38.77463	1165	TUR
	U1762	subsp. *dioica*	JC, TU	0.595	4	0.7	2.5	1	N40.07921	E38.77463	1165	TUR
	U1763	subsp. *dioica*	JC, TU	0.579	4	0.8	2.0	1	N40.07921	E38.77463	1165	TUR
UP0524	U1765	subsp. *dioica*	JC, TU	0.594	4	0.8	2.9	1	N39.85510	E38.39826	2080	TUR
UP0525	U1767	subsp. *kurdistanica*	JC, TU	0.316	2	1.3	1.8	1	N38.52032	E35.52553	2196	TUR
	U1768	subsp. *kurdistanica*	JC, TU	0.309	2	0.7	2.3	1	N38.52032	E35.52553	2196	TUR
	U1769	subsp. *kurdistanica*	JC, TU	0.319	2	0.9	3.8	1	N38.52032	E35.52553	2196	TUR
	U1770	subsp. *kurdistanica*	JC, TU	0.322	2	1.2	3.5	1	N38.52032	E35.52553	2196	TUR
	U1771	subsp. *kurdistanica*	JC, TU	0.311	2	0.8	3.0	1	N38.52032	E35.52553	2196	TUR
UP0526	U1772	subsp. *kurdistanica*	JC, TU	0.300	2	1.2	2.4	1	N38.55548	E35.50371	2251	TUR
	U1773	subsp. *kurdistanica*	JC, TU	0.305	2	0.8	2.5	1	N38.55548	E35.50371	2251	TUR
	U1774	subsp. *kurdistanica*	JC, TU	0.304	2	1.1	2.2	1	N38.55548	E35.50371	2251	TUR
	U1775	subsp. *kurdistanica*	JC, TU	0.302	2	1.0	2.4	1	N38.55548	E35.50371	2251	TUR
	U1776	subsp. *kurdistanica*	JC, TU	0.304	2	1.6	2.3	1	N38.55548	E35.50371	2251	TUR
UP0527	U1777	subsp. *kurdistanica*	JC, TU	0.300	2	1.2	2.5	1	N37.35832	E34.69034	1765	TUR
	U1778	subsp. *kurdistanica*	JC, TU	0.302	2	1.1	2.2	1	N37.35832	E34.69034	1765	TUR
	U1779	subsp. *kurdistanica*	JC, TU	0.298	2	1.3	2.3	1	N37.35832	E34.69034	1765	TUR

ID number of population	ID number of analysis	Taxon	Collector	Relative fluorescence intensi	DNA-ploidy level	CV of standard	CV of sample	N. of individuals in analysis	Latitude (WGS-84)	Longitude (WGS-84)	Altitude (m a.s.l.)	Country (ISO 3166-1)
	U1781	subsp. *kurdistanica*	JC, TU	0.298	2	0.7	2.2	1	N37.35832	E34.69034	1765	TUR
	U1782	subsp. *kurdistanica*	JC, TU	0.302	2	1.1	2.5	1	N37.35832	E34.69034	1765	TUR
UP0528	U1783	subsp. *subinermis*	JC, TU	0.321	2	1.0	3.4	1	N37.51418	E34.62849	1208	TUR
	U1784	subsp. *subinermis*	JC, TU	0.323	2	1.2	4.1	1	N37.51418	E34.62849	1208	TUR
	U1785	subsp. *subinermis*	JC, TU	0.317	2	0.8	2.9	1	N37.51418	E34.62849	1208	TUR
UP0529	U1786	subsp. *subinermis*	JC, TU	0.321	2	1.0	4.7	1	N38.71923	E37.35649	1250	TUR
	U1788	subsp. *subinermis*	JC, TU	0.316	2	0.7	3.4	1	N38.71923	E37.35649	1250	TUR
	U1789	subsp. *subinermis*	JC, TU	0.324	2	0.9	2.9	1	N38.71923	E37.35649	1250	TUR
UP0530	U1790	subsp. *dioica*	JC, TU	0.580	4	0.9	1.5	1	N39.01303	E40.70639	1195	TUR
	U1791	subsp. *dioica*	JC, TU	0.587	4	1.1	2.1	1	N39.01303	E40.70639	1195	TUR
	U1792	subsp. *dioica*	JC, TU	0.600	4	1.1	4.4	1	N39.01303	E40.70639	1195	TUR
	U1793	subsp. *dioica*	JC, TU	0.588	4	0.7	1.7	1	N39.01303	E40.70639	1195	TUR
UP0531	U1794	subsp. *dioica*	JC, TU	0.591	4	0.7	2.4	1	N39.65896	E41.04833	2142	TUR
	U1795	subsp. *dioica*	JC, TU	0.579	4	0.9	1.9	1	N39.65896	E41.04833	2142	TUR
	U1796	subsp. *dioica*	JC, TU	0.598	4	1.0	1.8	1	N39.65896	E41.04833	2142	TUR
UP0532	U1797	subsp. *dioica*	JC, TU	0.576	4	0.8	3.6	1	N40.11127	E40.98937	1938	TUR
UP0533	U1801	subsp. *dioica*	JC, TU	0.571	4	1.3	2.2	1	N40.17477	E40.97288	2322	TUR
	U1802	subsp. *dioica*	JC, TU	0.596	4	1.3	2.8	1	N40.17477	E40.97288	2322	TUR
UP0534	U1803	subsp. *dioica*	JC, TU	0.605	4	1.5	3.0	1	N40.34862	E40.78948	2380	TUR
UP0535	U1804	subsp. *dioica*	JC, TU	0.567	4	0.8	2.4	1	N40.62250	E40.78063	2740	TUR
	U1805	subsp. *dioica*	JC, TU	0.609	4	0.9	2.2	1	N40.62250	E40.78063	2740	TUR
	U1806	subsp. *dioica*	JC, TU	0.574	4	0.8	1.2	1	N40.62250	E40.78063	2740	TUR
	U1807	subsp. *dioica*	JC, TU	0.616	4	1.0	4.0	1	N40.62250	E40.78063	2740	TUR

ID number of population	ID number of analysis	Taxon	Collector	Relative fluorescence intensi	DNA-ploidy level	CV of standard	CV of sample	N. of individuals in analysis	Latitude (WGS-84)	Longitude (WGS-84)	Altitude (m a.s.l.)	Country (ISO 3166-1)
UP0536	U1809	subsp. *dioica*	JC, TU	0.563	4	0.9	2.2	1	N40.70466	E40.65491	1492	TUR
UP0537	U1811	subsp. *dioica*	JC, TU	0.556	4	1.1	2.8	1	N40.75945	E40.59265	800	TUR
	U1812	subsp. *dioica*	JC, TU	0.570	4	1.0	2.2	1	N40.75945	E40.59265	800	TUR
	U1813	subsp. *dioica*	JC, TU	0.579	4	0.8	2.9	1	N40.75945	E40.59265	800	TUR
UP0538	U1814	subsp. *dioica*	JC, TU	0.578	4	0.9	2.0	1	N40.98679	E40.33489	8	TUR
	U1815	subsp. *dioica*	JC, TU	0.582	4	0.9	1.8	1	N40.98679	E40.33489	8	TUR
	U1816	subsp. *dioica*	JC, TU	0.596	4	0.9	2.9	1	N40.98679	E40.33489	8	TUR
UP0539	U1818	subsp. *dioica*	LR, SS	0.557	4	0.9	1.5	1	N64.07651	E20.88490	29	SWE
	U1819	subsp. *dioica*	LR, SS	0.557	4	0.9	1.5	1	N64.07651	E20.88490	29	SWE
	U1820	subsp. *dioica*	LR, SS	0.557	4	0.9	1.5	1	N64.07651	E20.88490	29	SWE
	U1821	subsp. *dioica*	LR, SS	0.557	4	0.9	1.5	1	N64.07651	E20.88490	69	SWE
UP0540	U1823	subsp. *dioica*	LR, SS	0.559	4	0.8	2.6	1	N55.67270	E13.33271	69	SWE
	U1824	subsp. *dioica*	LR, SS	0.592	4	0.8	2.4	1	N55.67270	E13.33271	69	SWE
	U1825	subsp. *dioica*	LR, SS	0.563	4	0.9	2.4	1	N55.67270	E13.33271	69	SWE
	U1826	subsp. *dioica*	LR, SS	0.576	4	0.9	3.0	1	N55.67270	E13.33271	69	SWE
UP0541	U1828	subsp. *dioica*	LR, SS	0.571	4	1.2	1.6	1	N55.67540	E13.33463	66	SWE
	U1829	subsp. *dioica*	LR, SS	0.571	4	1.2	1.6	1	N55.67540	E13.33463	66	SWE
	U1830	subsp. *dioica*	LR, SS	0.571	4	1.2	1.6	1	N55.67540	E13.33463	66	SWE
	U1831	subsp. *dioica*	LR, SS	0.571	4	1.2	1.6	1	N55.67540	E13.33463	66	SWE
UP0542	U1832	subsp. *dioica*	LR, SS	0.590	4	1.6	1.3	1	N55.93731	E13.39284	63	SWE
	U1833	subsp. *dioica*	LR, SS	0.590	4	1.6	1.3	1	N55.93731	E13.39284	63	SWE
	U1834	subsp. *dioica*	LR, SS	0.590	4	1.6	1.3	1	N55.93731	E13.39284	63	SWE
	U1835	subsp. *dioica*	LR, SS	0.590	4	1.6	1.3	1	N55.93731	E13.39284	63	SWE

ID number of population	ID number of analysis	Taxon	Collector	Relative fluorescence intensi	DNA-ploidy level	CV of standard	CV of sample	N. of individuals in analysis	Latitude (WGS-84)	Longitude (WGS-84)	Altitude (m a.s.l.)	Country (ISO 3166-1)
UP0543	U1837	subsp. *dioica*	LR, SS	0.572	4	1.2	1.7	1	N56.03463	E13.34168	40	SWE
	U1838	subsp. *dioica*	LR, SS	0.572	4	1.2	1.7	1	N56.03463	E13.34168	40	SWE
	U1839	subsp. *dioica*	LR, SS	0.572	4	1.2	1.7	1	N56.03463	E13.34168	40	SWE
	U1840	subsp. *dioica*	LR, SS	0.572	4	1.2	1.7	1	N56.03463	E13.34168	40	SWE
	U1841	subsp. *dioica*	LR, SS	0.572	4	1.2	1.7	1	N56.03463	E13.34168	40	SWE
	U1842	subsp. *dioica*	LR, SS	0.572	4	1.2	1.7	1	N56.03463	E13.34168	40	SWE
UP0544	U1845	subsp. *dioica*	LR, SS	0.569	4	1.7	1.9	4	N56.88250	E14.56061	164	SWE
	U1846	subsp. *dioica*	LR, SS	0.569	4	1.7	1.9	4	N56.88250	E14.56061	164	SWE
	U1847	subsp. *dioica*	LR, SS	0.569	4	1.7	1.9	4	N56.88250	E14.56061	164	SWE
	U1848	subsp. *dioica*	LR, SS	0.569	4	1.7	1.9	4	N56.88250	E14.56061	164	SWE
UP0545	U1849	subsp. *dioica*	LR, SS	0.580	4	1.9	1.6	1	N57.10716	E14.16966	153	SWE
	U1850	subsp. *dioica*	LR, SS	0.580	4	1.9	1.6	1	N57.10716	E14.16966	153	SWE
	U1851	subsp. *dioica*	LR, SS	0.580	4	1.9	1.6	1	N57.10716	E14.16966	153	SWE
	U1852	subsp. *dioica*	LR, SS	0.580	4	1.9	1.6	1	N57.10716	E14.16966	153	SWE
UP0546	U1854	subsp. *dioica*	LR, SS	0.564	4	0.9	2.4	1	N57.28708	E13.93266	174	SWE
	U1856	subsp. *dioica*	LR, SS	0.564	4	0.9	2.4	1	N57.28708	E13.93266	174	SWE
	U1857	subsp. *dioica*	LR, SS	0.564	4	0.9	2.4	1	N57.28708	E13.93266	174	SWE
	U1858	subsp. *dioica*	LR, SS	0.564	4	0.9	2.4	1	N57.28708	E13.93266	174	SWE
UP0547	U1860	subsp. *dioica*	LR, SS	0.608	4	0.8	2.3	1	N57.35338	E13.93296	166	SWE
	U1861	subsp. *dioica*	LR, SS	0.588	4	0.9	1.9	1	N57.35338	E13.93296	166	SWE
	U1862	subsp. *dioica*	LR, SS	0.610	4	0.9	2.1	1	N57.35338	E13.93296	166	SWE
	U1863	subsp. *dioica*	LR, SS	0.583	4	0.9	1.5	1	N57.35338	E13.93296	166	SWE
UP0548	U1865	subsp. *dioica*	LR, SS	0.559	4	1.5	1.7	4	N58.36045	E14.35256	138	SWE

ID number of population	ID number of analysis	Taxon	Collector	Relative fluorescence intensi	DNA-ploidy level	CV of standard	CV of sample	N. of individuals in analysis	Latitude (WGS-84)	Longitude (WGS-84)	Altitude (m a.s.l.)	Country (ISO 3166-1)
	U1866	subsp. *dioica*	LR, SS	0.559	4	1.5	1.7	4	N58.36045	E14.35256	138	SWE
	U1867	subsp. *dioica*	LR, SS	0.559	4	1.5	1.7	4	N58.36045	E14.35256	138	SWE
	U1868	subsp. *dioica*	LR, SS	0.559	4	1.5	1.7	4	N58.36045	E14.35256	138	SWE
UP0549	U1870	subsp. *dioica*	LR, SS	0.582	4	1.1	1.8	4	N58.67096	E14.61750	114	SWE
	U1871	subsp. *dioica*	LR, SS	0.582	4	1.1	1.8	4	N58.67096	E14.61750	114	SWE
	U1872	subsp. *dioica*	LR, SS	0.582	4	1.1	1.8	4	N58.67096	E14.61750	114	SWE
	U1873	subsp. *dioica*	LR, SS	0.582	4	1.1	1.8	4	N58.67096	E14.61750	114	SWE
UP0550	U1876	subsp. *dioica*	LR, SS	0.591	4	1.0	2.4	1	N58.71581	E14.59296	183	SWE
UP0551	U1877	subsp. *dioica*	LR, SS	0.584	4	0.8	1.9	1	N59.02181	E14.59265	106	SWE
	U1878	subsp. *dioica*	LR, SS	0.584	4	0.8	1.9	1	N59.02181	E14.59265	106	SWE
	U1879	subsp. *dioica*	LR, SS	0.584	4	0.8	1.9	1	N59.02181	E14.59265	106	SWE
	U1880	subsp. *dioica*	LR, SS	0.584	4	0.8	1.9	1	N59.02181	E14.59265	106	SWE
UP0552	U1882	subsp. *dioica*	LR, SS	0.577	4	0.8	1.9	1	N59.54798	E14.28916	130	SWE
	U1884	subsp. *dioica*	LR, SS	0.567	4	1.0	3.1	1	N59.54798	E14.28916	130	SWE
UP0553	U1885	subsp. *dioica*	LR, SS	0.581	4	1.2	2.7	1	N60.51391	E14.23883	254	SWE
	U1886	subsp. *dioica*	LR, SS	0.585	4	1.3	2.3	4	N60.51391	E14.23883	254	SWE
	U1887	subsp. *dioica*	LR, SS	0.585	4	1.3	2.3	4	N60.51391	E14.23883	254	SWE
	U1888	subsp. *dioica*	LR, SS	0.585	4	1.3	2.3	4	N60.51391	E14.23883	254	SWE
	U1889	subsp. *dioica*	LR, SS	0.585	4	1.3	2.3	4	N60.51391	E14.23883	254	SWE
UP0554	U1890	subsp. *dioica*	LR, SS	0.603	4	0.9	2.6	1	N60.82551	E14.12665	283	SWE
	U1891	subsp. *dioica*	LR, SS	0.574	4	0.8	1.5	3	N60.82551	E14.12665	283	SWE
	U1892	subsp. *dioica*	LR, SS	0.574	4	0.8	1.5	3	N60.82551	E14.12665	283	SWE
	U1893	subsp. *dioica*	LR, SS	0.574	4	0.8	1.5	3	N60.82551	E14.12665	283	SWE

ID number of population	ID number of analysis	Taxon	Collector	Relative fluorescence intensi	DNA-ploidy level	CV of standard	CV of sample	N. of individuals in analysis	Latitude (WGS-84)	Longitude (WGS-84)	Altitude (m a.s.l.)	Country (ISO 3166-1)
UP0555	U1896	subsp. *dioica*	LR, SS	0.593	4	0.9	2.4	1	N60.96453	E13.89091	284	SWE
	U1897	subsp. *dioica*	LR, SS	0.565	4	0.9	1.5	4	N60.96453	E13.89091	284	SWE
	U1898	subsp. *dioica*	LR, SS	0.565	4	0.9	1.5	4	N60.96453	E13.89091	284	SWE
	U1899	subsp. *dioica*	LR, SS	0.565	4	0.9	1.5	4	N60.96453	E13.89091	284	SWE
	U1900	subsp. *dioica*	LR, SS	0.596	4	0.7	2.0	1	N60.96453	E13.89091	284	SWE
	U1901	subsp. *dioica*	LR, SS	0.565	4	0.9	1.5	4	N60.96453	E13.89091	284	SWE
UP0556	U1903	subsp. *dioica*	LR, SS	0.573	4	1.4	1.6	1	N61.28001	E13.76493	267	SWE
	U1904	subsp. *dioica*	LR, SS	0.573	4	1.4	1.6	1	N61.28001	E13.76493	267	SWE
	U1905	subsp. *dioica*	LR, SS	0.573	4	1.4	1.6	1	N61.28001	E13.76493	267	SWE
	U1906	subsp. *dioica*	LR, SS	0.573	4	1.4	1.6	1	N61.28001	E13.76493	267	SWE
UP0557	U1907	subsp. *dioica*	LR, SS	0.598	4	1.9	3.8	1	N61.69381	E13.14653	442	SWE
	U1908	subsp. *dioica*	LR, SS	0.567	4	1.4	1.2	3	N61.69381	E13.14653	442	SWE
	U1909	subsp. *dioica*	LR, SS	0.567	4	1.4	1.2	3	N61.69381	E13.14653	442	SWE
	U1910	subsp. *dioica*	LR, SS	0.567	4	1.4	1.2	3	N61.69381	E13.14653	442	SWE
UP0558	U1913	subsp. *dioica*	LR, SS	0.572	4	0.9	1.4	1	N61.84621	E14.05050	467	SWE
	U1914	subsp. *dioica*	LR, SS	0.572	4	0.9	1.4	1	N61.84621	E14.05050	467	SWE
	U1915	subsp. *dioica*	LR, SS	0.572	4	0.9	1.4	1	N61.84621	E14.05050	467	SWE
	U1916	subsp. *dioica*	LR, SS	0.572	4	0.9	1.4	1	N61.84621	E14.05050	467	SWE
UP0559	U1918	subsp. *dioica*	LR, SS	0.568	4	1.3	1.6	4	N62.04211	E14.67931	350	SWE
	U1919	subsp. *dioica*	LR, SS	0.568	4	1.3	1.6	4	N62.04211	E14.67931	350	SWE
	U1920	subsp. *dioica*	LR, SS	0.568	4	1.3	1.6	4	N62.04211	E14.67931	350	SWE
	U1921	subsp. *dioica*	LR, SS	0.568	4	1.3	1.6	4	N62.04211	E14.67931	350	SWE
UP0560	U1923	subsp. *dioica*	LR, SS	0.572	4	0.9	1.6	1	N62.17363	E14.94276	270	SWE

ID number of population	ID number of analysis	Taxon	Collector	Relative fluorescence intensi	DNA-ploidy level	CV of standard	CV of sample	N. of individuals in analysis	Latitude (WGS-84)	Longitude (WGS-84)	Altitude (m a.s.l.)	Country (ISO 3166-1)
	U1924	subsp. *dioica*	LR, SS	0.572	4	0.9	1.6	1	N62.17363	E14.94276	270	SWE
	U1925	subsp. *dioica*	LR, SS	0.572	4	0.9	1.6	1	N62.17363	E14.94276	270	SWE
	U1926	subsp. *dioica*	LR, SS	0.572	4	0.9	1.6	1	N62.17363	E14.94276	270	SWE
UP0561	U1928	subsp. *dioica*	LR, SS	0.585	4	1.6	1.2	1	N62.78120	E14.43401	295	SWE
	U1929	subsp. *dioica*	LR, SS	0.585	4	1.6	1.2	1	N62.78120	E14.43401	295	SWE
	U1930	subsp. *dioica*	LR, SS	0.585	4	1.6	1.2	1	N62.78120	E14.43401	295	SWE
	U1931	subsp. *dioica*	LR, SS	0.585	4	1.6	1.2	1	N62.78120	E14.43401	295	SWE
	U1932	subsp. *dioica*	LR, SS	0.585	4	1.6	1.2	1	N62.78120	E14.43401	295	SWE
UP0562	U1933	subsp. *dioica*	LR, SS	0.576	4	1.0	1.8	5	N63.20056	E13.95024	363	SWE
	U1934	subsp. *dioica*	LR, SS	0.576	4	1.0	1.8	5	N63.20056	E13.95024	363	SWE
	U1935	subsp. *dioica*	LR, SS	0.576	4	1.0	1.8	5	N63.20056	E13.95024	363	SWE
	U1937	subsp. *dioica*	LR, SS	0.576	4	1.0	1.8	5	N63.20056	E13.95024	363	SWE
	U1938	subsp. *dioica*	LR, SS	0.576	4	1.0	1.8	5	N63.20056	E13.95024	363	SWE
UP0563	U1939	subsp. *dioica*	LR, SS	0.571	4	1.2	1.6	5	N63.22173	E13.92875	362	SWE
	U1940	subsp. *dioica*	LR, SS	0.571	4	1.2	1.6	5	N63.22173	E13.92875	362	SWE
	U1941	subsp. *dioica*	LR, SS	0.571	4	1.2	1.6	5	N63.22173	E13.92875	362	SWE
	U1942	subsp. *dioica*	LR, SS	0.571	4	1.2	1.6	5	N63.22173	E13.92875	362	SWE
	U1943	subsp. *dioica*	LR, SS	0.571	4	1.2	1.6	5	N63.22173	E13.92875	362	SWE
	U1945	subsp. *dioica*	LR, SS	0.598	4	1.0	2.9	1	N63.22173	E13.92875	362	SWE
	U1946	subsp. *dioica*	LR, SS	0.583	4	1.2	3.5	1	N63.22173	E13.92875	362	SWE
	U1947	subsp. *dioica*	LR, SS	0.614	4	1.0	3.5	1	N63.22173	E13.92875	362	SWE
	U1948	subsp. *dioica*	LR, SS	0.594	4	1.9	2.3	1	N63.22173	E13.92875	362	SWE
UP0564	U1950	subsp. *dioica*	LR, SS	0.576	4	0.7	1.2	4	N63.19455	E15.13136	309	SWE

ID number of population	ID number of analysis	Taxon	Collector	Relative fluorescence intensi	DNA-ploidy level	CV of standard	CV of sample	N. of individuals in analysis	Latitude (WGS-84)	Longitude (WGS-84)	Altitude (m a.s.l.)	Country (ISO 3166-1)
	U1951	subsp. *dioica*	LR, SS	0.576	4	0.7	1.2	4	N63.19455	E15.13136	309	SWE
	U1952	subsp. *dioica*	LR, SS	0.576	4	0.7	1.2	4	N63.19455	E15.13136	309	SWE
	U1953	subsp. *dioica*	LR, SS	0.576	4	0.7	1.2	4	N63.19455	E15.13136	309	SWE
UP0565	U1954	subsp. *dioica*	LR, SS	0.593	4	1.1	3.7	1	N62.95636	E16.67799	111	SWE
	U1956	subsp. *dioica*	LR, SS	0.598	4	1.2	3.1	1	N62.95636	E16.67799	111	SWE
	U1957	subsp. *dioica*	LR, SS	0.617	4	1.0	3.1	1	N62.95636	E16.67799	111	SWE
UP0566	U1959	subsp. *dioica*	LR, SS	0.582	4	0.7	2.2	1	N63.14806	E17.76411	23	SWE
	U1960	subsp. *dioica*	LR, SS	0.584	4	0.9	3.3	1	N63.14806	E17.76411	23	SWE
	U1962	subsp. *dioica*	LR, SS	0.585	4	1.4	2.2	1	N63.14806	E17.76411	23	SWE
	U1963	subsp. *dioica*	LR, SS	0.584	4	0.9	1.6	1	N63.14806	E17.76411	23	SWE
UP0567	U1964	subsp. *dioica*	LR, SS	0.608	4	0.7	2.5	1	N63.28958	E18.64225	1	SWE
	U1965	subsp. *dioica*	LR, SS	0.575	4	0.8	1.4	1	N63.28958	E18.64225	1	SWE
	U1966	subsp. *dioica*	LR, SS	0.575	4	0.8	1.4	1	N63.28958	E18.64225	1	SWE
	U1967	subsp. *dioica*	LR, SS	0.575	4	0.8	1.4	1	N63.28958	E18.64225	1	SWE
	U1968	subsp. *dioica*	LR, SS	0.575	4	0.8	1.4	1	N63.28958	E18.64225	1	SWE
UP0568	U1969	subsp. *dioica*	LR, SS	0.578	4	0.9	1.1	3	N64.06273	E20.86291	16	SWE
	U1970	subsp. *dioica*	LR, SS	0.578	4	0.9	1.1	3	N64.06273	E20.86291	16	SWE
	U1972	subsp. *dioica*	LR, SS	0.622	4	0.8	3.5	1	N64.06273	E20.86291	16	SWE
	U1973	subsp. *dioica*	LR, SS	0.578	4	0.9	1.1	3	N64.06273	E20.86291	16	SWE
UP0569	U1974	subsp. *dioica*	LR, SS	0.578	4	1.5	1.7	1	N64.53873	E21.25659	64	SWE
	U1975	subsp. *dioica*	LR, SS	0.578	4	1.5	1.7	1	N64.53873	E21.25659	64	SWE
	U1976	subsp. *dioica*	LR, SS	0.578	4	1.5	1.7	1	N64.53873	E21.25659	64	SWE
	U1977	subsp. *dioica*	LR, SS	0.578	4	1.5	1.7	1	N64.53873	E21.25659	64	SWE

ID number of population	ID number of analysis	Taxon	Collector	Relative fluorescence intensi	DNA-ploidy level	CV of standard	CV of sample	N. of individuals in analysis	Latitude (WGS-84)	Longitude (WGS-84)	Altitude (m a.s.l.)	Country (ISO 3166-1)
UP0570	U1980	subsp. *dioica*	LR, SS	0.563	4	1.2	1.7	1	N65.14340	E21.50853	24	SWE
	U1981	subsp. *dioica*	LR, SS	0.563	4	1.2	1.7	1	N65.14340	E21.50853	24	SWE
	U1982	subsp. *dioica*	LR, SS	0.563	4	1.2	1.7	1	N65.14340	E21.50853	24	SWE
	U1983	subsp. *dioica*	LR, SS	0.563	4	1.2	1.7	1	N65.14340	E21.50853	24	SWE
UP0571	U1985	subsp. *dioica*	LR, SS	0.572	4	0.9	1.5	1	N65.37790	E21.29759	24	SWE
	U1986	subsp. *dioica*	LR, SS	0.572	4	0.9	1.5	1	N65.37790	E21.29759	24	SWE
	U1987	subsp. *dioica*	LR, SS	0.572	4	0.9	1.5	1	N65.37790	E21.29759	24	SWE
	U1988	subsp. *dioica*	LR, SS	0.572	4	0.9	1.5	1	N65.37790	E21.29759	24	SWE
UP0572	U1989	subsp. *dioica*	LR, SS	0.572	4	1.1	1.4	1	N65.35451	E21.58293	10	SWE
	U1990	subsp. *dioica*	LR, SS	0.572	4	1.1	1.4	1	N65.35451	E21.58293	10	SWE
	U1991	subsp. *dioica*	LR, SS	0.572	4	1.1	1.4	1	N65.35451	E21.58293	10	SWE
	U1992	subsp. *dioica*	LR, SS	0.572	4	1.1	1.4	1	N65.35451	E21.58293	10	SWE
	U1993	subsp. *dioica*	LR, SS	0.572	4	1.1	1.4	1	N65.35451	E21.58293	10	SWE
UP0573	U1994	subsp. *dioica*	LR, SS	0.567	4	1.9	1.7	1	N65.88580	E22.95945	55	SWE
	U1995	subsp. *dioica*	LR, SS	0.567	4	1.9	1.7	1	N65.88580	E22.95945	55	SWE
	U1997	subsp. *dioica*	LR, SS	0.567	4	1.9	1.7	1	N65.88580	E22.95945	55	SWE
	U1998	subsp. *dioica*	LR, SS	0.567	4	1.9	1.7	1	N65.88580	E22.95945	55	SWE
UP0574	U1999	subsp. *dioica*	LR, SS	0.574	4	0.9	1.3	1	N65.71911	E23.07973	63	SWE
	U2000	subsp. *dioica*	LR, SS	0.574	4	0.9	1.3	1	N65.71911	E23.07973	63	SWE
	U2001	subsp. *dioica*	LR, SS	0.574	4	0.9	1.3	1	N65.71911	E23.07973	63	SWE
	U2002	subsp. *dioica*	LR, SS	0.574	4	0.9	1.3	1	N65.71911	E23.07973	63	SWE
	U2003	subsp. *dioica*	LR, SS	0.574	4	0.9	1.3	1	N65.71911	E23.07973	63	SWE
UP0575	U2005	subsp. *dioica*	LR, SS	0.571	4	0.9	1.8	3	N66.14780	E23.92623	44	FIN

ID number of population	ID number of analysis	Taxon	Collector	Relative fluorescence intensi	DNA-ploidy level	CV of standard	CV of sample	N. of individuals in analysis	Latitude (WGS-84)	Longitude (WGS-84)	Altitude (m a.s.l.)	Country (ISO 3166-1)
	U2006	subsp. *dioica*	LR, SS	0.571	4	0.9	1.8	3	N66.14780	E23.92623	44	FIN
	U2007	subsp. *dioica*	LR, SS	0.571	4	0.9	1.8	3	N66.14780	E23.92623	44	FIN
	U2008	subsp. *dioica*	LR, SS	0.575	4	1.3	1.6	4	N66.14780	E23.92623	44	FIN
	U2009	subsp. *dioica*	LR, SS	0.575	4	1.3	1.6	4	N66.14780	E23.92623	44	FIN
	U2010	subsp. *dioica*	LR, SS	0.575	4	1.3	1.6	4	N66.14780	E23.92623	44	FIN
	U2011	subsp. *dioica*	LR, SS	0.575	4	1.3	1.6	4	N66.14780	E23.92623	44	FIN
	U2013	subsp. *dioica*	LR, SS	0.573	4	1.2	1.6	4	N66.14780	E23.92623	44	FIN
	U2014	subsp. *dioica*	LR, SS	0.573	4	1.2	1.6	4	N66.14780	E23.92623	44	FIN
	U2015	subsp. *dioica*	LR, SS	0.573	4	1.2	1.6	4	N66.14780	E23.92623	44	FIN
	U2016	subsp. *dioica*	LR, SS	0.573	4	1.2	1.6	4	N66.14780	E23.92623	44	FIN
UP0576	U2018	subsp. *dioica*	LR, SS	0.587	4	1.9	2.5	1	N66.21545	E23.71080	45	SWE
	U2019	subsp. *dioica*	LR, SS	0.587	4	1.9	2.5	1	N66.21545	E23.71080	45	SWE
	U2021	subsp. *dioica*	LR, SS	0.587	4	1.9	2.5	1	N66.21545	E23.71080	45	SWE
	U2022	subsp. *dioica*	LR, SS	0.587	4	1.9	2.5	1	N66.21545	E23.71080	45	SWE
	U2023	subsp. *dioica*	LR, SS	0.587	4	1.9	2.5	1	N66.21545	E23.71080	45	SWE
	U2024	subsp. *dioica*	LR, SS	0.587	4	1.9	2.5	1	N66.21545	E23.71080	45	SWE
UP0577	U2025	subsp. *dioica*	LR, SS	0.598	4	0.9	3.4	1	N66.56066	E23.83698	104	SWE
	U2026	subsp. *dioica*	LR, SS	0.615	4	0.9	3.3	1	N66.56066	E23.83698	104	SWE
	U2028	subsp. *dioica*	LR, SS	0.600	4	1.2	2.1	1	N66.56066	E23.83698	104	SWE
UP0578	U2030	subsp. *dioica*	LR, SS	0.588	4	0.8	1.5	1	N66.45353	E22.39183	88	SWE
	U2031	subsp. *dioica*	LR, SS	0.601	4	0.7	3.2	1	N66.45353	E22.39183	88	SWE
	U2032	subsp. *dioica*	LR, SS	0.591	4	0.8	2.4	1	N66.45353	E22.39183	88	SWE
	U2033	subsp. *dioica*	LR, SS	0.571	4	1.0	2.4	1	N66.45353	E22.39183	88	SWE

ID number of population	ID number of analysis	Taxon	Collector	Relative fluorescence intensi	DNA-ploidy level	CV of standard	CV of sample	N. of individuals in analysis	Latitude (WGS-84)	Longitude (WGS-84)	Altitude (m a.s.l.)	Country (ISO 3166-1)
	U2034	subsp. *dioica*	LR, SS	0.624	4	1.3	2.7	1	N66.45353	E22.39183	88	SWE
UP0579	U2036	subsp. *dioica*	LR, SS	0.595	4	1.0	2.3	1	N68.43923	E22.46148	313	SWE
	U2037	subsp. *dioica*	LR, SS	0.606	4	1.0	2.9	1	N68.43923	E22.46148	313	SWE
	U2038	subsp. *dioica*	LR, SS	0.602	4	0.9	2.2	1	N68.43923	E22.46148	313	SWE
	U2039	subsp. *dioica*	LR, SS	0.599	4	0.9	1.9	1	N68.43923	E22.46148	313	SWE
UP0580	U2041	subsp. *sondenii*	LR, SS	0.306	2	1.0	2.6	1	N68.48526	E22.29748	328	FIN
	U2043	subsp. *sondenii*	LR, SS	0.308	2	0.9	3.4	1	N68.48526	E22.29748	328	FIN
	U2044	subsp. *sondenii*	LR, SS	0.312	2	0.8	3.5	1	N68.48526	E22.29748	328	FIN
UP0581	U2045	subsp. *dioica*	LR, SS	0.590	4	1.2	3.9	1	N69.92744	E23.27426	13	NOR
	U2046	subsp. *dioica*	LR, SS	0.593	4	0.9	2.6	1	N69.92744	E23.27426	13	NOR
	U2047	subsp. *dioica*	LR, SS	0.584	4	1.0	2.2	1	N69.92744	E23.27426	13	NOR
	U2048	subsp. *dioica*	LR, SS	0.598	4	0.9	1.7	1	N69.92744	E23.27426	13	NOR
	U2049	subsp. *dioica*	LR, SS	0.631	4	0.9	2.6	1	N69.92744	E23.27426	13	NOR
	U2050	subsp. *dioica*	LR, SS	0.608	4	0.8	1.3	1	N69.92744	E23.27426	13	NOR
	U2051	subsp. *dioica*	LR, SS	0.593	4	1.2	1.5	1	N69.92744	E23.27426	13	NOR
	U2052	subsp. *dioica*	LR, SS	0.606	4	1.1	2.7	1	N69.92744	E23.27426	13	NOR
	U2053	subsp. *dioica*	LR, SS	0.581	4	1.0	2.4	1	N69.92744	E23.27426	13	NOR
	U2054	subsp. *dioica*	LR, SS	0.584	4	1.1	1.9	1	N69.92744	E23.27426	13	NOR
UP0582	U2055	subsp. *sondenii*	LR, SS	0.322	2	0.8	3.5	1	N70.51665	E25.07363	13	NOR
	U2056	subsp. *sondenii*	LR, SS	0.294	2	1.2	2.1	1	N70.51665	E25.07363	13	NOR
	U2058	subsp. *sondenii*	LR, SS	0.301	2	1.3	2.6	1	N70.51665	E25.07363	13	NOR
	U2059	subsp. *sondenii*	LR, SS	0.308	2	0.8	3.0	1	N70.51665	E25.07363	13	NOR
	U2060	subsp. *sondenii*	LR, SS	0.306	2	1.0	2.5	1	N70.51665	E25.07363	13	NOR

ID number of population	ID number of analysis	Taxon	Collector	Relative fluorescence intensi	DNA-ploidy level	CV of standard	CV of sample	N. of individuals in analysis	Latitude (WGS-84)	Longitude (WGS-84)	Altitude (m a.s.l.)	Country (ISO 3166-1)
UP0583	U2061	subsp. *dioica*	LR, SS	0.603	4	1.0	2.4	1	N70.03428	E24.97239	47	NOR
	U2062	subsp. *sondenii*	LR, SS	0.326	2	1.6	3.6	1	N70.03428	E24.97239	47	NOR
	U2063	subsp. *sondenii*	LR, SS	0.315	2	0.8	2.6	1	N70.03428	E24.97239	47	NOR
	U2064	subsp. *dioica*	LR, SS	0.616	4	0.9	2.3	1	N70.03428	E24.97239	47	NOR
	U2065	subsp. *dioica*	LR, SS	0.603	4	1.3	2.8	1	N70.03428	E24.97239	47	NOR
	U2066	subsp. *dioica*	LR, SS	0.584	4	1.0	1.4	1	N70.03428	E24.97239	47	NOR
	U2067	subsp. *dioica*	LR, SS	0.595	4	0.9	1.8	1	N70.03428	E24.97239	47	NOR
	U2068	subsp. *dioica*	LR, SS	0.579	4	0.7	2.8	1	N70.03428	E24.97239	47	NOR
	U2069	subsp. *dioica*	LR, SS	0.620	4	0.9	2.1	1	N70.03428	E24.97239	47	NOR
	U2070	subsp. *dioica*	LR, SS	0.592	4	1.1	3.3	1	N70.03428	E24.97239	47	NOR
UP0584	U2072	subsp. *sondenii*	LR, SS	0.297	2	1.3	1.9	1	N67.83978	E26.72058	182	FIN
	U2073	subsp. *sondenii*	LR, SS	0.299	2	0.9	2.8	1	N67.83978	E26.72058	182	FIN
	U2074	subsp. *sondenii*	LR, SS	0.311	2	1.0	3.7	1	N67.83978	E26.72058	182	FIN
	U2075	subsp. *sondenii*	LR, SS	0.311	2	1.5	3.3	1	N67.83978	E26.72058	182	FIN
	U2076	subsp. *sondenii*	LR, SS	0.302	2	1.3	2.3	1	N67.83978	E26.72058	182	FIN
UP0585	U2078	subsp. *dioica*	LR, SS	0.587	4	1.1	1.7	4	N66.53263	E25.71350	70	FIN
	U2079	subsp. *dioica*	LR, SS	0.587	4	1.1	1.7	4	N66.53263	E25.71350	70	FIN
	U2080	subsp. *dioica*	LR, SS	0.587	4	1.1	1.7	4	N66.53263	E25.71350	70	FIN
	U2081	subsp. *dioica*	LR, SS	0.587	4	1.1	1.7	4	N66.53263	E25.71350	70	FIN
UP0586	U2083	subsp. *dioica*	LR, SS	0.570	4	0.9	1.6	3	N65.99875	E26.21096	141	FIN
	U2084	subsp. *dioica*	LR, SS	0.570	4	0.9	1.6	3	N65.99875	E26.21096	141	FIN
	U2085	subsp. *dioica*	LR, SS	0.570	4	0.9	1.6	3	N65.99875	E26.21096	141	FIN
UP0587	U2087	subsp. *dioica*	LR, SS	0.554	4	1.4	2.4	5	N65.32458	E25.37945	13	FIN

ID number of population	ID number of analysis	Taxon	Collector	Relative fluorescence intensi	DNA-ploidy level	CV of standard	CV of sample	N. of individuals in analysis	Latitude (WGS-84)	Longitude (WGS-84)	Altitude (m a.s.l.)	Country (ISO 3166-1)
	U2088	subsp. *dioica*	LR, SS	0.554	4	1.4	2.4	5	N65.32458	E25.37945	13	FIN
	U2089	subsp. *dioica*	LR, SS	0.554	4	1.4	2.4	5	N65.32458	E25.37945	13	FIN
	U2090	subsp. *dioica*	LR, SS	0.554	4	1.4	2.4	5	N65.32458	E25.37945	13	FIN
	U2091	subsp. *dioica*	LR, SS	0.554	4	1.4	2.4	5	N65.32458	E25.37945	13	FIN
UP0588	U2092	subsp. *dioica*	LR, SS	0.573	4	1.0	1.4	4	N64.84421	E25.17436	7	FIN
	U2093	subsp. *dioica*	LR, SS	0.604	4	0.9	3.4	1	N64.84421	E25.17436	7	FIN
	U2094	subsp. *dioica*	LR, SS	0.573	4	1.0	1.4	4	N64.84421	E25.17436	7	FIN
	U2095	subsp. *dioica*	LR, SS	0.573	4	1.0	1.4	4	N64.84421	E25.17436	7	FIN
	U2096	subsp. *dioica*	LR, SS	0.573	4	1.0	1.4	4	N64.84421	E25.17436	7	FIN
UP0589	U2098	subsp. *dioica*	LR, SS	0.586	4	1.1	1.6	3	N64.85576	E24.72666	91	FIN
	U2099	subsp. *dioica*	LR, SS	0.589	4	1.0	2.6	1	N64.85576	E24.72666	91	FIN
	U2100	subsp. *dioica*	LR, SS	0.586	4	1.1	1.6	3	N64.85576	E24.72666	91	FIN
	U2101	subsp. *dioica*	LR, SS	0.608	4	0.9	2.4	1	N64.85576	E24.72666	91	FIN
UP0590	U2102	subsp. *dioica*	LR, SS	0.611	4	1.0	2.7	1	N64.46155	E24.23703	53	FIN
	U2103	subsp. *dioica*	LR, SS	0.587	4	1.0	2.0	1	N64.46155	E24.23703	53	FIN
	U2104	subsp. *dioica*	LR, SS	0.573	4	0.8	1.7	1	N64.46155	E24.23703	53	FIN
	U2105	subsp. *dioica*	LR, SS	0.585	4	0.9	2.4	1	N64.46155	E24.23703	53	FIN
UP0591	U2108	subsp. *dioica*	LR, SS	0.589	4	1.0	2.0	1	N64.28918	E23.91998	48	FIN
	U2109	subsp. *subinermis*	LR, SS	0.302	2	1.3	2.5	1	N64.28918	E23.91998	48	FIN
	U2110	subsp. *dioica*	LR, SS	0.580	4	0.9	1.3	1	N64.28918	E23.91998	48	FIN
	U2111	subsp. *dioica*	LR, SS	0.583	4	1.2	1.7	1	N64.28918	E23.91998	48	FIN
	U2112	subsp. *dioica*	LR, SS	0.571	4	1.1	1.2	1	N64.28918	E23.91998	48	FIN
	U2113	subsp. *dioica*	LR, SS	0.572	4	1.7	1.5	1	N64.28918	E23.91998	48	FIN

ID number of population	ID number of analysis	Taxon	Collector	Relative fluorescence intensi	DNA-ploidy level	CV of standard	CV of sample	N. of individuals in analysis	Latitude (WGS-84)	Longitude (WGS-84)	Altitude (m a.s.l.)	Country (ISO 3166-1)
	U2114	subsp. *dioica*	LR, SS	0.571	4	1.3	1.5	1	N64.28918	E23.91998	48	FIN
	U2115	subsp. *dioica*	LR, SS	0.577	4	1.5	1.5	1	N64.28918	E23.91998	48	FIN
UP0592	U2117	subsp. *subinermis*	LR, SS	0.301	2	0.8	1.8	1	N63.90906	E25.00998	84	FIN
	U2118	subsp. *subinermis*	LR, SS	0.297	2	1.6	1.8	1	N63.90906	E25.00998	84	FIN
	U2119	subsp. *subinermis*	LR, SS	0.299	2	0.7	1.6	1	N63.90906	E25.00998	84	FIN
	U2120	subsp. *subinermis*	LR, SS	0.299	2	0.9	1.8	1	N63.90906	E25.00998	84	FIN
UP0593	U2122	subsp. *subinermis*	LR, SS	0.297	2	0.8	1.7	2	N63.59490	E27.16149	93	FIN
	U2123	subsp. *subinermis*	LR, SS	0.297	2	0.8	1.7	2	N63.59490	E27.16149	93	FIN
	U2124	subsp. *subinermis*	LR, SS	0.297	2	1.7	1.7	2	N63.59490	E27.16149	93	FIN
	U2125	subsp. *subinermis*	LR, SS	0.297	2	1.7	1.7	2	N63.59490	E27.16149	93	FIN
UP0594	U2126	subsp. *subinermis*	LR, SS	0.297	2	1.3	1.8	2	N62.16090	E28.15618	91	FIN
	U2127	subsp. *subinermis*	LR, SS	0.318	2	0.9	4.0	1	N62.16090	E28.15618	91	FIN
	U2128	subsp. *subinermis*	LR, SS	0.297	2	1.3	1.8	2	N62.16090	E28.15618	91	FIN
	U2130	subsp. *subinermis*	LR, SS	0.300	2	1.9	2.7	2	N62.16090	E28.15618	91	FIN
	U2131	subsp. *subinermis*	LR, SS	0.300	2	1.9	2.7	2	N62.16090	E28.15618	91	FIN
UP0595	U2133	subsp. *subinermis*	LR, SS	0.306	2	0.9	3.7	2	N61.84828	E29.17859	129	FIN
	U2134	subsp. *subinermis*	LR, SS	0.306	2	0.9	3.7	2	N61.84828	E29.17859	129	FIN
UP0596	U2136	subsp. *subinermis*	LR, SS	0.293	2	1.1	2.7	1	N61.48451	E29.48244	88	FIN
	U2137	subsp. *subinermis*	LR, SS	0.294	2	0.9	2.4	1	N61.48451	E29.48244	88	FIN
	U2138	subsp. *subinermis*	LR, SS	0.299	2	0.8	2.4	1	N61.48451	E29.48244	88	FIN
	U2139	subsp. *subinermis*	LR, SS	0.297	2	0.9	1.9	1	N61.48451	E29.48244	88	FIN
UP0597	U2140	subsp. *subinermis*	LR, SS	0.306	2	0.8	2.6	1	N61.86335	E29.28756	74	FIN
	U2141	subsp. *subinermis*	LR, SS	0.302	2	1.3	2.0	2	N61.86335	E29.28756	74	FIN

ID number of population	ID number of analysis	Taxon	Collector	Relative fluorescence intensi	DNA-ploidy level	CV of standard	CV of sample	N. of individuals in analysis	Latitude (WGS-84)	Longitude (WGS-84)	Altitude (m a.s.l.)	Country (ISO 3166-1)
	U2142	subsp. *subinermis*	LR, SS	0.302	2	1.3	2.0	2	N61.86335	E29.28756	74	FIN
	U2143	subsp. *subinermis*	LR, SS	0.296	2	0.8	2.1	2	N61.86335	E29.28756	74	FIN
	U2144	subsp. *subinermis*	LR, SS	0.296	2	0.8	2.1	2	N61.86335	E29.28756	74	FIN
UP0598	U2145	subsp. *subinermis*	LR, SS	0.299	2	1.0	2.6	1	N62.06351	E27.56121	127	FIN
	U2147	subsp. *subinermis*	LR, SS	0.298	2	1.0	2.2	1	N62.06351	E27.56121	127	FIN
UP0599	U2148	subsp. *subinermis*	LR, SS	0.302	2	1.1	3.2	1	N62.39703	E26.42971	99	FIN
	U2149	subsp. *subinermis*	LR, SS	0.308	2	1.3	3.4	1	N62.39703	E26.42971	99	FIN
	U2150	subsp. *subinermis*	LR, SS	0.295	2	0.9	2.3	1	N62.39703	E26.42971	99	FIN
UP0600	U2152	subsp. *subinermis*	LR, SS	0.302	2	0.8	2.1	1	N62.90926	E24.82408	150	FIN
	U2153	subsp. *subinermis*	LR, SS	0.297	2	0.8	1.7	1	N62.90926	E24.82408	150	FIN
UP0601	U2155	subsp. *dioica*	LR, SS	0.574	4	0.9	2.1	3	N63.03061	E23.83238	127	FIN
	U2156	subsp. *dioica*	LR, SS	0.574	4	0.9	2.1	3	N63.03061	E23.83238	127	FIN
	U2157	subsp. *dioica*	LR, SS	0.574	4	0.9	2.1	3	N63.03061	E23.83238	127	FIN
	U2158	subsp. *dioica*	LR, SS	0.566	4	1.0	1.1	1	N63.03061	E23.83238	127	FIN
UP0602	U2160	subsp. *dioica*	LR, SS	0.575	4	1.2	1.4	4	N62.95746	E22.54081	37	FIN
	U2161	subsp. *dioica*	LR, SS	0.575	4	1.2	1.4	4	N62.95746	E22.54081	37	FIN
	U2162	subsp. *dioica*	LR, SS	0.575	4	1.2	1.4	4	N62.95746	E22.54081	37	FIN
	U2163	subsp. *dioica*	LR, SS	0.575	4	1.2	1.4	4	N62.95746	E22.54081	37	FIN
UP0603	U2165	subsp. *dioica*	LR, SS	0.589	4	1.5	1.6	1	N62.97150	E21.49270	4	FIN
	U2166	subsp. *dioica*	LR, SS	0.589	4	1.5	1.6	1	N62.97150	E21.49270	4	FIN
	U2167	subsp. *dioica*	LR, SS	0.589	4	1.5	1.6	1	N62.97150	E21.49270	4	FIN
UP0604	U2169	subsp. *dioica*	LR, SS	0.572	4	1.1	1.3	5	N62.72737	E21.17329	1	FIN
	U2170	subsp. *dioica*	LR, SS	0.572	4	1.1	1.3	5	N62.72737	E21.17329	1	FIN

ID number of population	ID number of analysis	Taxon	Collector	Relative fluorescence intensi	DNA-ploidy level	CV of standard	CV of sample	N. of individuals in analysis	Latitude (WGS-84)	Longitude (WGS-84)	Altitude (m a.s.l.)	Country (ISO 3166-1)
	U2171	subsp. *dioica*	LR, SS	0.572	4	1.1	1.3	5	N62.72737	E21.17329	1	FIN
	U2172	subsp. *dioica*	LR, SS	0.572	4	1.1	1.3	5	N62.72737	E21.17329	1	FIN
	U2173	subsp. *dioica*	LR, SS	0.572	4	1.1	1.3	5	N62.72737	E21.17329	1	FIN
UP0605	U2175	subsp. *dioica*	LR, SS	0.582	4	1.3	1.5	3	N62.26381	E21.36276	6	FIN
	U2176	subsp. *dioica*	LR, SS	0.582	4	1.3	1.5	3	N62.26381	E21.36276	6	FIN
	U2177	subsp. *dioica*	LR, SS	0.582	4	1.3	1.5	3	N62.26381	E21.36276	6	FIN
UP0606	U2179	subsp. *dioica*	LR, SS	0.582	4	0.9	1.3	1	N61.56547	E21.68465	1	FIN
	U2180	subsp. *dioica*	LR, SS	0.588	4	0.8	1.3	1	N61.56547	E21.68465	1	FIN
	U2181	subsp. *dioica*	LR, SS	0.574	4	0.7	1.4	1	N61.56547	E21.68465	1	FIN
	U2182	subsp. *dioica* pentaploid	LR, SS	0.715	5	0.8	1.2	1	N61.56547	E21.68465	1	FIN
	U2183	subsp. *dioica*	LR, SS	0.572	4	0.8	1.3	1	N61.56547	E21.68465	1	FIN
UP0607	U2185	subsp. *dioica*	LR, SS	0.575	4	1.5	1.3	1	N61.12825	E21.49078	20	FIN
	U2186	subsp. *dioica*	LR, SS	0.575	4	1.5	1.3	1	N61.12825	E21.49078	20	FIN
	U2187	subsp. *dioica*	LR, SS	0.575	4	1.5	1.3	1	N61.12825	E21.49078	20	FIN
	U2188	subsp. *dioica*	LR, SS	0.575	4	1.5	1.3	1	N61.12825	E21.49078	20	FIN
UP0608	U2189	subsp. *dioica*	LR, SS	0.568	4	0.9	1.3	3	N60.23938	E24.65983	19	FIN
	U2191	subsp. *dioica*	LR, SS	0.568	4	0.9	1.3	3	N60.23938	E24.65983	19	FIN
	U2192	subsp. *dioica*	LR, SS	0.568	4	0.9	1.3	3	N60.23938	E24.65983	19	FIN
UP0609	U2194	subsp. *dioica*	LR, SS	0.569	4	1.2	1.9	3	N59.58903	E25.70749	26	EST
	U2195	subsp. *dioica*	LR, SS	0.569	4	1.2	1.9	3	N59.58903	E25.70749	26	EST
	U2196	subsp. *dioica*	LR, SS	0.569	4	1.2	1.9	3	N59.58903	E25.70749	26	EST
UP0610	U2198	subsp. *dioica*	LR, SS	0.568	4	1.2	1.6	4	N59.56408	E25.86721	19	EST
	U2199	subsp. *dioica*	LR, SS	0.568	4	1.2	1.6	4	N59.56408	E25.86721	19	EST

ID number of population	ID number of analysis	Taxon	Collector	Relative fluorescence intensi	DNA-ploidy level	CV of standard	CV of sample	N. of individuals in analysis	Latitude (WGS-84)	Longitude (WGS-84)	Altitude (m a.s.l.)	Country (ISO 3166-1)
	U2200	subsp. *dioica*	LR, SS	0.568	4	1.2	1.6	4	N59.56408	E25.86721	19	EST
	U2201	subsp. *dioica*	LR, SS	0.568	4	1.2	1.6	4	N59.56408	E25.86721	19	EST
UP0611	U2202	subsp. *dioica*	LR, SS	0.567	4	0.8	1.4	3	N59.23143	E24.71170	55	EST
	U2203	subsp. *dioica*	LR, SS	0.567	4	0.8	1.4	3	N59.23143	E24.71170	55	EST
	U2204	subsp. *dioica*	LR, SS	0.567	4	0.8	1.4	3	N59.23143	E24.71170	55	EST
UP0612	U2207	subsp. *dioica*	LR, SS	0.580	4	1.8	1.6	3	N59.38068	E27.55390	1	EST
	U2208	subsp. *dioica*	LR, SS	0.580	4	1.8	1.6	3	N59.38068	E27.55390	1	EST
	U2209	subsp. *dioica*	LR, SS	0.580	4	1.8	1.6	3	N59.38068	E27.55390	1	EST
UP0613	U2211	subsp. *dioica*	LR, SS	0.564	4	1.7	1.7	4	N58.98710	E27.16925	40	EST
	U2212	subsp. *dioica*	LR, SS	0.564	4	1.7	1.7	4	N58.98710	E27.16925	40	EST
	U2213	subsp. *dioica*	LR, SS	0.564	4	1.7	1.7	4	N58.98710	E27.16925	40	EST
	U2214	subsp. *dioica*	LR, SS	0.564	4	1.7	1.7	4	N58.98710	E27.16925	40	EST
UP0614	U2215	subsp. *dioica*	LR, SS	0.579	4	1.0	1.3	3	N58.94098	E27.04663	31	EST
	U2216	subsp. *dioica*	LR, SS	0.579	4	1.0	1.3	3	N58.94098	E27.04663	31	EST
	U2217	subsp. *dioica*	LR, SS	0.579	4	1.0	1.3	3	N58.94098	E27.04663	31	EST
UP0615	U2219	subsp. *dioica*	LR, SS	0.577	4	1.3	1.5	1	N58.01028	E26.13123	118	EST
	U2221	subsp. *dioica*	LR, SS	0.577	4	1.3	1.5	1	N58.01028	E26.13123	118	EST
	U2222	subsp. *dioica*	LR, SS	0.577	4	1.3	1.5	1	N58.01028	E26.13123	118	EST
	U2223	subsp. *dioica*	LR, SS	0.577	4	1.3	1.5	1	N58.01028	E26.13123	118	EST
UP0616	U2225	subsp. *dioica*	LR, SS	0.569	4	1.3	1.5	4	N57.98581	E26.04486	71	EST
	U2226	subsp. *dioica*	LR, SS	0.569	4	1.3	1.5	4	N57.98581	E26.04486	71	EST
	U2227	subsp. *dioica*	LR, SS	0.569	4	1.3	1.5	4	N57.98581	E26.04486	71	EST
	U2228	subsp. *dioica*	LR, SS	0.569	4	1.3	1.5	4	N57.98581	E26.04486	71	EST

ID number of population	ID number of analysis	Taxon	Collector	Relative fluorescence intensi	DNA-ploidy level	CV of standard	CV of sample	N. of individuals in analysis	Latitude (WGS-84)	Longitude (WGS-84)	Altitude (m a.s.l.)	Country (ISO 3166-1)
UP0617	U2236	subsp. *dioica*	LR, SS	0.563	4	1.1	1.3	2	N56.01236	E23.40613	94	LTU
	U2237	subsp. *dioica*	LR, SS	0.563	4	1.1	1.3	2	N56.01236	E23.40613	94	LTU
UP0618	U2239	subsp. *dioica*	LR, SS	0.566	4	1.3	2.3	3	N55.82765	E23.10500	143	LTU
	U2240	subsp. *dioica*	LR, SS	0.566	4	1.3	2.3	3	N55.82765	E23.10500	143	LTU
	U2241	subsp. *dioica*	LR, SS	0.566	4	1.3	2.3	3	N55.82765	E23.10500	143	LTU
UP0619	U2243	subsp. *dioica*	LR, SS	0.566	4	1.3	1.5	5	N54.80492	E24.26690	53	LTU
	U2244	subsp. *dioica*	LR, SS	0.566	4	1.3	1.5	5	N54.80492	E24.26690	53	LTU
	U2245	subsp. *dioica*	LR, SS	0.566	4	1.3	1.5	5	N54.80492	E24.26690	53	LTU
	U2246	subsp. *dioica*	LR, SS	0.566	4	1.3	1.5	5	N54.80492	E24.26690	53	LTU
	U2247	subsp. *dioica*	LR, SS	0.566	4	1.3	1.5	5	N54.80492	E24.26690	53	LTU
UP0620	U2248	subsp. *dioica*	LR, SS	0.573	4	0.8	1.9	1	N54.64675	E24.93551	156	LTU
	U2250	subsp. *dioica*	LR, SS	0.579	4	1.1	1.2	2	N54.64675	E24.93551	156	LTU
	U2251	subsp. *dioica*	LR, SS	0.579	4	1.1	1.2	2	N54.64675	E24.93551	156	LTU
UP0621	U2264	subsp. *dioica*	PV, TU	0.567	4	0.8	1.1	1	N51.10167	E17.07150	132	POL
	U2265	subsp. *dioica*	PV, TU	0.572	4	0.8	1.2	1	N51.10167	E17.07150	132	POL
	U2266	subsp. *dioica*	PV, TU	0.571	4	0.9	1.2	1	N51.10167	E17.07150	132	POL
UP0622	U2268	subsp. *dioica*	PV, TU	0.580	4	1.6	1.6	5	N51.18257	E16.91867	114	POL
	U2269	subsp. *dioica*	PV, TU	0.580	4	1.6	1.6	5	N51.18257	E16.91867	114	POL
	U2270	subsp. *dioica*	PV, TU	0.580	4	1.6	1.6	5	N51.18257	E16.91867	114	POL
	U2271	subsp. *dioica*	PV, TU	0.580	4	1.6	1.6	5	N51.18257	E16.91867	114	POL
UP0623	U2272	subsp. *dioica*	PV, TU	0.566	4	1.2	1.8	3	N52.02578	E15.57197	50	POL
	U2273	subsp. *dioica*	PV, TU	0.566	4	1.2	1.8	3	N52.02578	E15.57197	50	POL
	U2274	subsp. *dioica*	PV, TU	0.566	4	1.2	1.8	3	N52.02578	E15.57197	50	POL

ID number of population	ID number of analysis	Taxon	Collector	Relative fluorescence intensi	DNA-ploidy level	CV of standard	CV of sample	N. of individuals in analysis	Latitude (WGS-84)	Longitude (WGS-84)	Altitude (m a.s.l.)	Country (ISO 3166-1)
UP0624	U2275	subsp. *dioica*	PV, TU	0.549	4	1.8	1.5	1	N52.58655	E14.61229	11	POL
	U2276	subsp. *dioica*	PV, TU	0.549	4	1.8	1.5	1	N52.58655	E14.61229	11	POL
	U2277	subsp. *dioica*	PV, TU	0.549	4	1.8	1.5	1	N52.58655	E14.61229	11	POL
UP0625	U2278	subsp. *dioica*	PV, TU	0.559	4	1.2	1.6	3	N52.60095	E14.60101	10	POL
	U2279	subsp. *dioica*	PV, TU	0.559	4	1.2	1.6	3	N52.60095	E14.60101	10	POL
	U2280	subsp. *dioica*	PV, TU	0.559	4	1.2	1.6	3	N52.60095	E14.60101	10	POL
UP0626	U2281	subsp. *dioica*	PV, TU	0.563	4	1.2	1.4	1	N52.48602	E12.83499	29	DEU
	U2282	subsp. *subinermis*	PV, TU	0.299	2	1.7	1.8	1	N52.48602	E12.83499	29	DEU
UP0627	U2284	subsp. *dioica*	PV, TU	0.557	4	0.9	1.4	1	N52.48055	E12.80989	29	DEU
	U2285	subsp. *subinermis*	PV, TU	0.294	2	1.4	2.3	1	N52.48055	E12.80989	29	DEU
UP0628	U2296	subsp. *subinermis*	ZK	0.297	2	0.9	2.2	1	N48.73739	E16.87706	155	CZE
	U2296	subsp. *subinermis*	ZK	0.301	2	0.7	2.7	1	N48.73739	E16.87706	155	CZE
	U2297	subsp. *subinermis*	ZK	0.296	2	0.9	3.9	1	N48.73739	E16.87706	155	CZE
	U2297	subsp. *subinermis*	ZK	0.300	2	1.1	3.2	1	N48.73739	E16.87706	155	CZE
UP0629	U2305	subsp. *dioica*	FK	0.602	4	1.0	2.1	1	N36.66114	E51.32025	190	IRN
	U2306	subsp. *dioica*	FK	0.624	4	0.9	2.3	1	N36.66114	E51.32025	190	IRN
	U2307	subsp. *dioica*	FK	0.613	4	0.6	2.3	1	N36.66114	E51.32025	190	IRN
UP0630	U2351	subsp. *dioica* aneuploid	LR	0.492	–	1.1	1.5	1	N47.62380	E12.97902	615	DEU
	U2352	subsp. *dioica*	LR	0.565	4	1.3	4.6	1	N47.62380	E12.97902	615	DEU
	U2353	subsp. *dioica*	LR	0.566	4	1.2	1.7	1	N47.62380	E12.97902	615	DEU
	U2354	subsp. *dioica*	LR	0.562	4	1.4	2.0	1	N47.62380	E12.97902	615	DEU
UP0631	U2356	subsp. *dioica*	LR	0.572	4	1.3	1.5	1	N46.68460	E13.67080	607	AUT
	U2357	subsp. *dioica*	LR	0.571	4	1.4	3.7	1	N46.68460	E13.67080	607	AUT

ID number of population	ID number of analysis	Taxon	Collector	Relative fluorescence intensi	DNA-ploidy level	CV of standard	CV of sample	N. of individuals in analysis	Latitude (WGS-84)	Longitude (WGS-84)	Altitude (m a.s.l.)	Country (ISO 3166-1)
UP0632	U2359	subsp. *pubescens*	LR	0.307	2	1.1	3.9	1	N46.39920	E13.16783	302	ITA
	U2360	subsp. *pubescens*	LR	0.305	2	1.0	3.0	1	N46.39920	E13.16783	302	ITA
	U2361	subsp. *pubescens*	LR	0.307	2	1.0	3.1	1	N46.39920	E13.16783	302	ITA
	U2362	subsp. *pubescens*	LR	0.297	2	1.1	2.1	1	N46.39920	E13.16783	302	ITA
	U2363	subsp. *pubescens*	LR	0.305	2	0.9	3.0	1	N46.39920	E13.16783	302	ITA
UP0633	U2364	subsp. *pubescens*	LR	0.299	2	1.1	3.5	1	N45.52650	E12.26158	12	ITA
	U2365	subsp. *pubescens*	LR	0.302	2	1.1	3.1	1	N45.52650	E12.26158	12	ITA
	U2366	subsp. *pubescens*	LR	0.298	2	1.0	3.5	1	N45.52650	E12.26158	12	ITA
UP0634	U2367	subsp. *pubescens*	LR	0.310	2	1.1	3.3	1	N44.94770	E12.26107	1	ITA
	U2368	subsp. *pubescens*	LR	0.306	2	0.9	3.1	1	N44.94770	E12.26107	1	ITA
	U2369	subsp. *pubescens*	LR	0.304	2	1.4	3.1	1	N44.94770	E12.26107	1	ITA
	U2370	subsp. *pubescens*	LR	0.309	2	1.5	3.6	1	N44.94770	E12.26107	1	ITA
	U2371	subsp. *dioica*	LR	0.611	4	1.2	1.3	1	N44.94770	E12.26107	1	ITA
UP0635	U2372	subsp. *pubescens*	LR	0.297	2	0.9	2.1	1	N44.95785	E12.33095	1	ITA
	U2373	subsp. *pubescens*	LR	0.300	2	1.1	1.9	1	N44.95785	E12.33095	1	ITA
	U2374	subsp. *pubescens*	LR	0.297	2	1.1	1.9	1	N44.95785	E12.33095	1	ITA
	U2375	subsp. *pubescens*	LR	0.300	2	1.1	2.2	1	N44.95785	E12.33095	1	ITA
UP0636	U2376	subsp. *pubescens*	LR	0.303	2	1.0	3.6	1	N45.50597	E11.93240	24	ITA
	U2377	subsp. *pubescens*	LR	0.306	2	1.2	3.8	1	N45.50597	E11.93240	24	ITA
	U2378	subsp. *pubescens*	LR	0.299	2	1.4	1.2	1	N45.50597	E11.93240	24	ITA
	U2379	subsp. *pubescens*	LR	0.303	2	1.1	2.1	1	N45.50597	E11.93240	24	ITA
	U2380	subsp. *pubescens*	LR	0.302	2	1.3	2.2	1	N45.50597	E11.93240	24	ITA
UP0637	U2381	subsp. *pubescens*	LR	0.302	2	1.0	2.3	1	N45.77563	E11.72663	114	ITA

ID number of population	ID number of analysis	Taxon	Collector	Relative fluorescence intensi	DNA-ploidy level	CV of standard	CV of sample	N. of individuals in analysis	Latitude (WGS-84)	Longitude (WGS-84)	Altitude (m a.s.l.)	Country (ISO 3166-1)
	U2382	subsp. *pubescens*	LR	0.297	2	1.3	2.3	1	N45.77563	E11.72663	114	ITA
	U2383	subsp. *pubescens*	LR	0.304	2	1.2	2.8	1	N45.77563	E11.72663	114	ITA
UP0638	U2384	subsp. *pubescens*	LR	0.298	2	1.2	4.9	1	N45.99642	E11.66612	224	ITA
	U2385	subsp. *pubescens*	LR	0.304	2	1.1	2.1	1	N45.99642	E11.66612	224	ITA
	U2386	subsp. *pubescens*	LR	0.299	2	1.1	2.2	1	N45.99642	E11.66612	224	ITA
	U2387	subsp. *pubescens*	LR	0.298	2	0.8	1.8	1	N45.99642	E11.66612	224	ITA
	U2388	subsp. *pubescens*	LR	0.299	2	1.4	2.3	1	N45.99642	E11.66612	224	ITA
UP0639	U2389	subsp. *pubescens*	LR	0.311	2	1.5	3.3	1	N46.00285	E11.29060	451	ITA
	U2391	subsp. *pubescens*	LR	0.320	2	1.2	4.7	1	N46.00285	E11.29060	451	ITA
	U2392	subsp. *pubescens*	LR	0.303	2	1.3	3.7	1	N46.00285	E11.29060	451	ITA
	U2393	subsp. *pubescens*	LR	0.301	2	1.0	2.0	1	N46.00285	E11.29060	451	ITA
UP0640	U2395	subsp. *pubescens*	LR	0.317	2	1.1	4.8	1	N45.90287	E11.02720	184	ITA
	U2396	subsp. *pubescens*	LR	0.299	2	1.1	4.3	1	N45.90287	E11.02720	184	ITA
	U2397	subsp. *pubescens*	LR	0.300	2	0.8	2.2	1	N45.90287	E11.02720	184	ITA
	U2398	subsp. *pubescens*	LR	0.300	2	1.3	5.0	1	N45.90287	E11.02720	184	ITA
UP0641	U2399	subsp. *pubescens*	LR	0.302	2	1.1	2.5	1	N45.58527	E10.45353	174	ITA
	U2401	subsp. *pubescens*	LR	0.301	2	1.1	2.2	1	N45.58527	E10.45353	174	ITA
UP0642	U2402	subsp. *pubescens*	LR	0.310	2	1.1	3.4	1	N45.60553	E10.19905	213	ITA
	U2403	subsp. *pubescens*	LR	0.307	2	1.2	3.1	1	N45.60553	E10.19905	213	ITA
	U2404	subsp. *pubescens*	LR	0.313	2	1.3	4.0	1	N45.60553	E10.19905	213	ITA
	U2405	subsp. *pubescens*	LR	0.301	2	1.1	2.4	1	N45.60553	E10.19905	213	ITA
	U2406	subsp. *pubescens*	LR	0.311	2	1.1	3.4	1	N45.60553	E10.19905	213	ITA
UP0643	U2407	subsp. *pubescens*	LR	0.304	2	1.7	2.3	1	N45.69125	E10.11113	332	ITA

ID number of population	ID number of analysis	Taxon	Collector	Relative fluorescence intensi	DNA-ploidy level	CV of standard	CV of sample	N. of individuals in analysis	Latitude (WGS-84)	Longitude (WGS-84)	Altitude (m a.s.l.)	Country (ISO 3166-1)
	U2408	subsp. *pubescens*	LR	0.303	2	1.0	1.4	1	N45.69125	E10.11113	332	ITA
UP0644	U2409	subsp. *pubescens*	LR	0.316	2	1.5	2.2	1	N45.75367	E10.11938	616	ITA
	U2410	subsp. *pubescens*	LR	0.324	2	0.8	4.0	1	N45.75367	E10.11938	616	ITA
	U2411	subsp. *pubescens*	LR	0.313	2	1.0	3.8	1	N45.75367	E10.11938	616	ITA
	U2412	subsp. *pubescens*	LR	0.295	2	1.0	1.2	1	N45.75367	E10.11938	616	ITA
UP0645	U2413	subsp. *pubescens*	LR	0.298	2	1.4	2.5	1	N45.92070	E10.23918	292	ITA
	U2414	subsp. *pubescens*	LR	0.302	2	1.1	1.8	1	N45.92070	E10.23918	292	ITA
	U2415	subsp. *pubescens*	LR	0.298	2	1.6	2.7	1	N45.92070	E10.23918	292	ITA
UP0646	U2416	subsp. *pubescens*	LR	0.299	2	0.9	1.8	1	N46.10165	E10.31545	556	ITA
	U2417	subsp. *pubescens*	LR	0.300	2	0.9	2.5	1	N46.10165	E10.31545	556	ITA
	U2418	subsp. *pubescens*	LR	0.325	2	1.2	4.8	1	N46.10165	E10.31545	556	ITA
	U2419	subsp. *pubescens*	LR	0.299	2	1.2	1.8	1	N46.10165	E10.31545	556	ITA
UP0647	U2420	subsp. *dioica*	LR	0.601	4	1.3	3.4	1	N46.24527	E10.44912	1225	ITA
	U2421	subsp. *dioica*	LR	0.572	4	1.3	1.3	1	N46.24527	E10.44912	1225	ITA
	U2422	subsp. *dioica*	LR	0.571	4	1.1	1.7	1	N46.24527	E10.44912	1225	ITA
	U2423	subsp. *dioica*	LR	0.619	4	1.3	2.3	1	N46.24527	E10.44912	1225	ITA
	U2424	subsp. *dioica*	LR	0.604	4	0.9	2.6	1	N46.24527	E10.44912	1225	ITA
UP0648	U2425	subsp. *dioica*	LR	0.580	4	1.1	1.5	1	N46.25853	E10.52680	1608	ITA
	U2426	subsp. *dioica*	LR	0.594	4	1.6	3.3	1	N46.25853	E10.52680	1608	ITA
	U2427	subsp. *dioica*	LR	0.601	4	1.1	2.8	1	N46.25853	E10.52680	1608	ITA
	U2428	subsp. *dioica*	LR	0.594	4	1.3	3.5	1	N46.25853	E10.52680	1608	ITA
UP0649	U2429	subsp. *dioica*	LR	0.568	4	1.0	1.4	1	N46.31120	E10.77390	951	ITA
	U2430	subsp. *dioica*	LR	0.584	4	1.1	1.4	1	N46.31120	E10.77390	951	ITA

ID number of population	ID number of analysis	Taxon	Collector	Relative fluorescence intensi	DNA-ploidy level	CV of standard	CV of sample	N. of individuals in analysis	Latitude (WGS-84)	Longitude (WGS-84)	Altitude (m a.s.l.)	Country (ISO 3166-1)
	U2431	subsp. *dioica*	LR	0.587	4	1.2	1.4	1	N46.31120	E10.77390	951	ITA
UP0650	U2435	subsp. *dioica*	LR	0.585	4	1.1	2.8	1	N46.48940	E11.03627	1404	ITA
UP0651	U2436	subsp. *pubescens*	LR	0.299	2	1.0	1.2	1	N46.70330	E11.18230	505	ITA
	U2437	subsp. *pubescens*	LR	0.323	2	0.9	3.2	1	N46.70330	E11.18230	505	ITA
	U2438	subsp. *pubescens*	LR	0.317	2	1.3	4.9	1	N46.70330	E11.18230	505	ITA
UP0652	U2439	subsp. *dioica*	LR	0.569	4	1.5	1.6	1	N46.61300	E10.58228	935	ITA
	U2440	subsp. *dioica*	LR	0.588	4	1.2	2.5	1	N46.61300	E10.58228	935	ITA
	U2441	subsp. *pubescens*	LR	0.300	2	0.9	2.2	1	N46.61300	E10.58228	935	ITA
	U2442	subsp. *dioica*	LR	0.626	4	1.2	2.4	1	N46.61300	E10.58228	935	ITA
	U2443	subsp. *pubescens*	LR	0.308	2	0.7	1.9	1	N46.61300	E10.58228	935	ITA
UP0653	U2445	subsp. *dioica*	LR	0.560	4	1.4	1.4	1	N46.47508	E10.36713	1275	ITA
	U2446	subsp. *dioica*	LR	0.564	4	1.1	1.6	1	N46.47508	E10.36713	1275	ITA
	U2447	subsp. *dioica*	LR	0.578	4	1.0	1.2	1	N46.47508	E10.36713	1275	ITA
	U2448	subsp. *dioica*	LR	0.580	4	1.4	2.0	1	N46.47508	E10.36713	1275	ITA
	U2449	subsp. *dioica*	LR	0.570	4	1.2	1.5	1	N46.47508	E10.36713	1275	ITA
UP0654	U2450	subsp. *pubescens*	LR	0.311	2	0.8	2.2	1	N46.23650	E10.22507	530	ITA
	U2451	subsp. *pubescens*	LR	0.308	2	1.9	1.9	1	N46.23650	E10.22507	530	ITA
	U2452	subsp. *pubescens*	LR	0.304	2	0.9	2.0	1	N46.23650	E10.22507	530	ITA
UP0655	U2453	subsp. *pubescens*	LR	0.304	2	1.4	2.5	1	N46.16257	E9.78898	269	ITA
	U2454	subsp. *pubescens*	LR	0.311	2	1.2	2.8	1	N46.16257	E9.78898	269	ITA
	U2455	subsp. *pubescens*	LR	0.304	2	0.8	1.9	1	N46.16257	E9.78898	269	ITA
	U2456	subsp. *pubescens*	LR	0.296	2	0.8	2.6	1	N46.16257	E9.78898	269	ITA
UP0656	U2457	subsp. *pubescens*	LR	0.301	2	1.2	2.3	1	N45.86217	E9.42163	444	ITA

ID number of population	ID number of analysis	Taxon	Collector	Relative fluorescence intensi	DNA-ploidy level	CV of standard	CV of sample	N. of individuals in analysis	Latitude (WGS-84)	Longitude (WGS-84)	Altitude (m a.s.l.)	Country (ISO 3166-1)
	U2459	subsp. *pubescens*	LR	0.303	2	1.9	2.3	1	N45.86217	E9.42163	444	ITA
	U2460	subsp. *pubescens*	LR	0.297	2	1.0	4.2	1	N45.86217	E9.42163	444	ITA
	U2461	subsp. *pubescens*	LR	0.306	2	0.8	2.8	1	N45.86217	E9.42163	444	ITA
UP0657	U2462	subsp. *pubescens*	LR	0.304	2	0.9	2.1	1	N45.80548	E9.25597	265	ITA
	U2463	subsp. *pubescens*	LR	0.321	2	1.1	3.8	1	N45.80548	E9.25597	265	ITA
	U2464	subsp. *pubescens*	LR	0.308	2	1.2	2.7	1	N45.80548	E9.25597	265	ITA
	U2465	subsp. *pubescens*	LR	0.299	2	1.0	2.1	1	N45.80548	E9.25597	265	ITA
	U2467	subsp. *pubescens*	LR	0.294	2	0.9	2.2	1	N45.80548	E9.25597	265	ITA
UP0658	U2468	subsp. *pubescens*	LR	0.306	2	0.8	3.4	1	N45.78667	E8.92007	411	ITA
	U2469	subsp. *dioica*	LR	0.609	4	0.8	1.4	1	N45.78667	E8.92007	411	ITA
	U2470	subsp. *pubescens*	LR	0.299	2	0.9	1.7	1	N45.78667	E8.92007	411	ITA
	U2471	subsp. *dioica*	LR	0.601	4	1.1	1.8	1	N45.78667	E8.92007	411	ITA
UP0659	U2472	subsp. *pubescens*	LR	0.309	2	1.0	2.4	1	N45.87413	E8.64148	239	ITA
	U2473	subsp. *pubescens*	LR	0.304	2	0.9	2.0	1	N45.87413	E8.64148	239	ITA
	U2474	subsp. *pubescens*	LR	0.307	2	1.6	1.9	1	N45.87413	E8.64148	239	ITA
	U2475	subsp. *pubescens*	LR	0.310	2	0.8	2.5	1	N45.87413	E8.64148	239	ITA
UP0660	U2476	subsp. *pubescens*	LR	0.302	2	0.8	1.8	1	N45.87413	E8.62487	197	ITA
	U2477	subsp. *pubescens*	LR	0.301	2	0.8	1.7	1	N45.87413	E8.62487	197	ITA
	U2478	subsp. *pubescens*	LR	0.299	2	0.8	1.7	1	N45.87413	E8.62487	197	ITA
	U2479	subsp. *pubescens*	LR	0.304	2	1.1	2.4	1	N45.87413	E8.62487	197	ITA
	U2480	subsp. *pubescens*	LR	0.304	2	1.2	2.8	1	N45.87413	E8.62487	197	ITA
UP0661	U2481	subsp. *pubescens*	LR	0.300	2	1.9	2.0	1	N45.44000	E8.29033	168	ITA
	U2482	subsp. *pubescens*	LR	0.301	2	1.7	2.1	1	N45.44000	E8.29033	168	ITA

ID number of population	ID number of analysis	Taxon	Collector	Relative fluorescence intensi	DNA-ploidy level	CV of standard	CV of sample	N. of individuals in analysis	Latitude (WGS-84)	Longitude (WGS-84)	Altitude (m a.s.l.)	Country (ISO 3166-1)
	U2483	subsp. *pubescens*	LR	0.304	2	0.9	2.9	1	N45.44000	E8.29033	168	ITA
	U2484	subsp. *pubescens*	LR	0.300	2	1.0	2.1	1	N45.44000	E8.29033	168	ITA
UP0662	U2485	subsp. *dioica*	LR	0.565	4	1.1	1.4	1	N45.56478	E7.81420	282	ITA
	U2486	subsp. *dioica*	LR	0.567	4	1.0	1.6	1	N45.56478	E7.81420	282	ITA
	U2487	subsp. *dioica*	LR	0.568	4	1.1	1.5	1	N45.56478	E7.81420	282	ITA
UP0663	U2488	subsp. *dioica*	LR	0.574	4	0.9	1.1	1	N45.90240	E6.85825	1187	FRA
	U2489	subsp. *dioica*	LR	0.578	4	0.9	1.4	1	N45.90240	E6.85825	1187	FRA
	U2490	subsp. *dioica*	LR	0.577	4	1.0	2.2	1	N45.90240	E6.85825	1187	FRA
	U2491	subsp. *dioica*	LR	0.576	4	0.9	1.4	1	N45.90240	E6.85825	1187	FRA
UP0664	U2492	subsp. *dioica*	LR	0.578	4	0.8	1.4	1	N45.81215	E6.96012	1266	ITA
	U2494	subsp. *dioica*	LR	0.579	4	1.6	1.3	1	N45.81215	E6.96012	1266	ITA
	U2495	subsp. *dioica*	LR	0.582	4	0.9	1.4	1	N45.81215	E6.96012	1266	ITA
UP0665	U2496	subsp. *pubescens*	LR	0.303	2	1.7	2.0	1	N45.70443	E7.67285	349	ITA
	U2497	subsp. *pubescens*	LR	0.310	2	0.9	1.7	1	N45.70443	E7.67285	349	ITA
	U2498	subsp. *pubescens*	LR	0.313	2	1.2	3.0	1	N45.70443	E7.67285	349	ITA
	U2499	subsp. *pubescens*	LR	0.309	2	0.9	2.3	1	N45.70443	E7.67285	349	ITA
UP0666	U2500	subsp. *pubescens*	LR	0.301	2	1.0	1.8	1	N45.21292	E7.78245	194	ITA
	U2501	subsp. *pubescens*	LR	0.299	2	0.9	1.8	1	N45.21292	E7.78245	194	ITA
	U2502	subsp. *pubescens*	LR	0.303	2	1.4	1.6	1	N45.21292	E7.78245	194	ITA
	U2503	subsp. *pubescens*	LR	0.303	2	1.3	2.5	1	N45.21292	E7.78245	194	ITA
	U2504	subsp. *pubescens*	LR	0.302	2	1.2	2.7	1	N45.21292	E7.78245	194	ITA
UP0667	U2505	subsp. *pubescens*	LR	0.305	2	1.1	2.4	1	N44.90648	E7.69230	223	ITA
	U2506	subsp. *pubescens*	LR	0.302	2	1.0	2.5	1	N44.90648	E7.69230	223	ITA

ID number of population	ID number of analysis	Taxon	Collector	Relative fluorescence intensi	DNA-ploidy level	CV of standard	CV of sample	N. of individuals in analysis	Latitude (WGS-84)	Longitude (WGS-84)	Altitude (m a.s.l.)	Country (ISO 3166-1)
	U2507	subsp. *pubescens*	LR	0.300	2	0.7	2.8	1	N44.90648	E7.69230	223	ITA
	U2508	subsp. *pubescens*	LR	0.302	2	1.2	1.8	1	N44.90648	E7.69230	223	ITA
	U2509	subsp. *pubescens*	LR	0.303	2	1.1	2.9	1	N44.90648	E7.69230	223	ITA
UP0668	U2510	subsp. *pubescens*	LR	0.306	2	1.3	3.5	1	N44.47222	E7.89325	275	ITA
	U2511	subsp. *pubescens*	LR	0.305	2	1.1	2.8	1	N44.47222	E7.89325	275	ITA
	U2512	subsp. *pubescens*	LR	0.301	2	1.0	2.1	1	N44.47222	E7.89325	275	ITA
	U2513	subsp. *pubescens*	LR	0.307	2	0.8	2.8	1	N44.47222	E7.89325	275	ITA
	U2514	subsp. *pubescens*	LR	0.301	2	1.2	2.1	1	N44.47222	E7.89325	275	ITA
UP0669	U2515	subsp. *pubescens*	LR	0.302	2	1.8	2.4	1	N44.37202	E8.07663	465	ITA
	U2516	subsp. *pubescens*	LR	0.306	2	0.9	2.1	1	N44.37202	E8.07663	465	ITA
	U2517	subsp. *pubescens*	LR	0.309	2	1.1	3.1	1	N44.37202	E8.07663	465	ITA
	U2518	subsp. *pubescens*	LR	0.307	2	1.5	2.4	1	N44.37202	E8.07663	465	ITA
UP0670	U2519	subsp. *pubescens*	LR	0.304	2	1.1	2.6	1	N44.34772	E8.20962	451	ITA
	U2520	subsp. *pubescens*	LR	0.304	2	1.2	2.3	1	N44.34772	E8.20962	451	ITA
	U2521	subsp. *pubescens*	LR	0.306	2	1.4	2.4	1	N44.34772	E8.20962	451	ITA
	U2522	subsp. *pubescens*	LR	0.307	2	1.2	2.4	1	N44.34772	E8.20962	451	ITA
	U2523	subsp. *pubescens*	LR	0.310	2	1.4	2.9	1	N44.34772	E8.20962	451	ITA
UP0671	U2524	subsp. *pubescens*	LR	0.299	2	0.9	1.9	1	N44.37552	E8.50473	83	ITA
	U2525	subsp. *pubescens*	LR	0.300	2	1.3	2.6	1	N44.37552	E8.50473	83	ITA
	U2526	subsp. *pubescens*	LR	0.300	2	1.2	3.6	1	N44.37552	E8.50473	83	ITA
	U2527	subsp. *pubescens*	LR	0.303	2	1.0	2.9	1	N44.37552	E8.50473	83	ITA
UP0672	U2528	subsp. *pubescens*	LR	0.303	2	1.1	3.3	1	N44.48730	E8.49667	387	ITA
	U2529	subsp. *pubescens*	LR	0.300	2	1.1	2.4	1	N44.48730	E8.49667	387	ITA

ID number of population	ID number of analysis	Taxon	Collector	Relative fluorescence intensi	DNA-ploidy level	CV of standard	CV of sample	N. of individuals in analysis	Latitude (WGS-84)	Longitude (WGS-84)	Altitude (m a.s.l.)	Country (ISO 3166-1)
	U2530	subsp. *pubescens*	LR	0.300	2	1.6	2.9	1	N44.48730	E8.49667	387	ITA
	U2531	subsp. *pubescens*	LR	0.299	2	1.3	2.7	1	N44.48730	E8.49667	387	ITA
	U2532	subsp. *pubescens*	LR	0.317	2	1.3	3.7	1	N44.48730	E8.49667	387	ITA
UP0673	U2533	subsp. *pubescens*	LR	0.311	2	1.0	2.7	1	N44.73902	E8.51898	122	ITA
	U2534	subsp. *pubescens*	LR	0.303	2	1.3	2.4	1	N44.73902	E8.51898	122	ITA
	U2535	subsp. *pubescens*	LR	0.303	2	1.5	2.2	1	N44.73902	E8.51898	122	ITA
UP0674	U2536	subsp. *dioica*	LR	0.592	4	1.0	1.7	1	N45.04970	E8.62982	86	ITA
	U2537	subsp. *pubescens*	LR	0.304	2	1.4	2.5	1	N45.04970	E8.62982	86	ITA
	U2538	subsp. *pubescens*	LR	0.300	2	1.3	2.6	1	N45.04970	E8.62982	86	ITA
	U2539	subsp. *pubescens*	LR	0.313	2	1.1	3.3	1	N45.04970	E8.62982	86	ITA
UP0675	U2540	subsp. *pubescens*	LR	0.299	2	1.1	2.7	1	N45.24735	E8.75097	101	ITA
	U2541	subsp. *pubescens*	LR	0.301	2	1.1	3.6	1	N45.24735	E8.75097	101	ITA
	U2542	subsp. *pubescens*	LR	0.300	2	1.2	2.1	1	N45.24735	E8.75097	101	ITA
	U2543	subsp. *pubescens*	LR	0.300	2	1.4	2.8	1	N45.24735	E8.75097	101	ITA
UP0676	U2544	subsp. *pubescens*	LR	0.298	2	1.3	2.4	1	N45.16738	E9.07907	65	ITA
	U2545	subsp. *pubescens*	LR	0.303	2	1.2	2.4	1	N45.16738	E9.07907	65	ITA
	U2546	subsp. *pubescens*	LR	0.301	2	1.2	2.2	1	N45.16738	E9.07907	65	ITA
	U2547	subsp. *pubescens*	LR	0.300	2	1.4	2.2	1	N45.16738	E9.07907	65	ITA
UP0677	U2548	subsp. *pubescens*	LR	0.303	2	1.4	2.2	1	N44.96310	E9.60420	106	ITA
	U2549	subsp. *pubescens*	LR	0.304	2	1.2	3.0	1	N44.96310	E9.60420	106	ITA
UP0678	U2550	subsp. *pubescens*	LR	0.312	2	1.1	2.8	1	N44.88212	E9.61557	342	ITA
	U2551	subsp. *pubescens*	LR	0.307	2	1.4	2.8	1	N44.88212	E9.61557	342	ITA
	U2552	subsp. *pubescens*	LR	0.307	2	1.4	2.8	1	N44.88212	E9.61557	342	ITA

ID number of population	ID number of analysis	Taxon	Collector	Relative fluorescence intensi	DNA-ploidy level	CV of standard	CV of sample	N. of individuals in analysis	Latitude (WGS-84)	Longitude (WGS-84)	Altitude (m a.s.l.)	Country (ISO 3166-1)
	U2553	subsp. *pubescens*	LR	0.303	2	1.0	2.2	1	N44.88212	E9.61557	342	ITA
UP0679	U2554	subsp. *pubescens*	LR	0.302	2	1.2	2.7	1	N44.66752	E9.61743	758	ITA
	U2555	subsp. *pubescens*	LR	0.309	2	1.3	2.5	1	N44.66752	E9.61743	758	ITA
	U2556	subsp. *pubescens*	LR	0.306	2	1.2	2.5	1	N44.66752	E9.61743	758	ITA
	U2557	subsp. *pubescens*	LR	0.307	2	0.9	2.2	1	N44.66752	E9.61743	758	ITA
	U2559	subsp. *pubescens*	LR	0.304	2	1.4	2.8	1	N44.66752	E9.61743	758	ITA
	U2560	subsp. *pubescens*	LR	0.299	2	1.7	3.4	1	N44.66752	E9.61743	758	ITA
	U2561	subsp. *pubescens*	LR	0.303	2	1.0	3.6	1	N44.66752	E9.61743	758	ITA
	U2562	subsp. *pubescens*	LR	0.304	2	1.5	2.1	1	N44.66752	E9.61743	758	ITA
UP0680	U2563	subsp. *pubescens*	LR	0.306	2	1.4	2.5	1	N44.65102	E9.81080	349	ITA
UP0681	U2564	subsp. *pubescens*	LR	0.300	2	1.5	2.7	1	N44.72987	E10.16108	111	ITA
	U2565	subsp. *pubescens*	LR	0.301	2	1.2	1.2	1	N44.72987	E10.16108	111	ITA
	U2566	subsp. *pubescens*	LR	0.303	2	1.9	2.7	1	N44.72987	E10.16108	111	ITA
	U2567	subsp. *pubescens*	LR	0.301	2	1.3	2.6	1	N44.72987	E10.16108	111	ITA
	U2568	subsp. *pubescens*	LR	0.303	2	1.4	1.8	1	N44.72987	E10.16108	111	ITA
UP0682	U2569	subsp. *pubescens*	LR	0.309	2	1.4	3.0	1	N44.93050	E10.36508	42	ITA
	U2570	subsp. *pubescens*	LR	0.305	2	1.3	2.7	1	N44.93050	E10.36508	42	ITA
	U2571	subsp. *pubescens*	LR	0.308	2	1.1	2.5	1	N44.93050	E10.36508	42	ITA
UP0683	U2572	subsp. *pubescens*	LR	0.307	2	1.3	2.3	1	N44.90670	E10.51723	14	ITA
	U2573	subsp. *pubescens*	LR	0.311	2	0.9	2.5	1	N44.90670	E10.51723	14	ITA
	U2574	subsp. *pubescens*	LR	0.324	2	1.2	3.8	1	N44.90670	E10.51723	14	ITA
	U2575	subsp. *pubescens*	LR	0.317	2	1.3	2.5	1	N44.90670	E10.51723	14	ITA
	U2576	subsp. *pubescens*	LR	0.307	2	1.1	2.6	1	N44.90670	E10.51723	14	ITA

ID number of population	ID number of analysis	Taxon	Collector	Relative fluorescence intensi	DNA-ploidy level	CV of standard	CV of sample	N. of individuals in analysis	Latitude (WGS-84)	Longitude (WGS-84)	Altitude (m a.s.l.)	Country (ISO 3166-1)
	U2577	subsp. *pubescens*	LR	0.302	2	1.4	2.5	1	N44.90670	E10.51723	14	ITA
UP0684	U2578	subsp. *pubescens*	LR	0.305	2	0.7	2.3	1	N45.06637	E10.84375	10	ITA
	U2579	subsp. *pubescens*	LR	0.299	2	1.2	2.6	1	N45.06637	E10.84375	10	ITA
	U2580	subsp. *pubescens*	LR	0.301	2	1.2	2.8	1	N45.06637	E10.84375	10	ITA
	U2581	subsp. *pubescens*	LR	0.307	2	0.7	2.5	1	N45.06637	E10.84375	10	ITA
UP0685	U2582	subsp. *dioica*	LR	0.577	4	1.0	1.2	1	N45.93151	E6.91746	1916	FRA
	U2583	subsp. *dioica*	LR	0.574	4	1.3	1.4	1	N45.93151	E6.91746	1916	FRA
	U2584	subsp. *dioica*	LR	0.561	4	1.2	1.4	1	N45.93151	E6.91746	1916	FRA
UP0686	U2585	subsp. *dioica*	LR	0.575	4	1.3	3.4	1	N48.22452	E17.21377	106	SVK
	U2586	subsp. *dioica*	LR	0.569	4	1.1	1.8	1	N48.22452	E17.21377	106	SVK
	U2587	subsp. *dioica*	LR	0.565	4	1.3	1.4	1	N48.22452	E17.21377	106	SVK
UP0687	U2588	subsp. *dioica*	LR	0.570	4	1.2	1.8	1	N48.22525	E17.21138	140	SVK
	U2589	subsp. *subinermis*	LR	0.298	2	1.5	1.6	1	N48.22525	E17.21138	140	SVK
	U2590	subsp. *dioica*	LR	0.575	4	1.3	1.5	1	N48.22525	E17.21138	140	SVK
	U2591	subsp. *subinermis*	LR	0.299	2	1.5	2.4	1	N48.22525	E17.21138	140	SVK
UP0688	U2592	subsp. *dioica*	LR	0.567	4	0.9	1.3	1	N48.24330	E17.25355	134	SVK
	U2593	subsp. *dioica*	LR	0.568	4	1.0	1.3	1	N48.24330	E17.25355	134	SVK
	U2594	subsp. *dioica*	LR	0.578	4	0.5	1.7	1	N48.24330	E17.25355	134	SVK
	U2595	subsp. *dioica*	LR	0.572	4	0.9	1.6	1	N48.24330	E17.25355	134	SVK
	U2596	subsp. *dioica*	LR	0.595	4	1.2	4.6	1	N48.24330	E17.25355	134	SVK
	U2597	subsp. *dioica*	LR	0.566	4	1.0	1.4	1	N48.24330	E17.25355	134	SVK
	U2598	subsp. *dioica*	LR	0.559	4	1.4	1.1	1	N48.24330	E17.25355	134	SVK
	U2599	*U. kioviensis*	LR	0.338	2	0.8	2.4	1	N48.24330	E17.25355	134	SVK

ID number of population	ID number of analysis	Taxon	Collector	Relative fluorescence intensi	DNA-ploidy level	CV of standard	CV of sample	N. of individuals in analysis	Latitude (WGS-84)	Longitude (WGS-84)	Altitude (m a.s.l.)	Country (ISO 3166-1)
	U2600	*U. kioviensis*	LR	0.338	2	1.1	1.7	1	N48.24330	E17.25355	134	SVK
	U2601	*U. kioviensis*	LR	0.338	2	1.1	2.3	1	N48.24330	E17.25355	134	SVK
UP0689	U2602	subsp. *dioica*	LR	0.570	4	1.1	1.6	1	N48.24555	E17.24822	135	SVK
	U2603	subsp. *dioica*	LR	0.577	4	0.9	1.6	1	N48.24555	E17.24822	135	SVK
	U2604	subsp. *dioica*	LR	0.567	4	1.2	1.3	1	N48.24555	E17.24822	135	SVK
	U2605	subsp. *dioica*	LR	0.571	4	0.9	1.4	1	N48.24555	E17.24822	135	SVK
	U2606	subsp. *dioica*	LR	0.577	4	0.9	1.5	1	N48.24555	E17.24822	135	SVK
UP0690	U2607	subsp. *dioica*	LR	0.564	4	1.2	1.8	1	N48.03842	E17.19805	134	SVK
	U2608	subsp. *dioica*	LR	0.575	4	0.9	1.5	1	N48.03842	E17.19805	134	SVK
UP0691	U2609	subsp. *dioica*	LR	0.574	4	1.3	1.5	1	N48.03718	E17.19877	140	SVK
	U2610	subsp. *dioica*	LR	0.573	4	1.4	1.4	1	N48.03718	E17.19877	140	SVK
	U2611	subsp. *dioica*	LR	0.573	4	1.0	1.5	1	N48.03718	E17.19877	140	SVK
UP0692	U2612	subsp. *dioica*	LR	0.572	4	0.9	1.4	1	N48.04298	E17.17520	139	SVK
	U2613	subsp. *dioica*	LR	0.568	4	0.8	1.4	1	N48.04298	E17.17520	139	SVK
	U2614	subsp. *dioica*	LR	0.571	4	0.9	1.5	1	N48.04298	E17.17520	139	SVK
UP0693	U2615	subsp. *dioica*	LR	0.561	4	1.0	1.6	1	N48.04942	E17.16158	139	SVK
UP0694	U2616	subsp. *dioica*	LR	0.566	4	0.8	1.2	1	N47.77967	E18.13973	102	SVK
	U2617	subsp. *subinermis*	LR	0.295	2	1.3	2.5	1	N47.77967	E18.13973	102	SVK
	U2618	subsp. *dioica*	LR	0.557	4	1.0	1.5	1	N47.77967	E18.13973	102	SVK
	U2619	subsp. *subinermis*	LR	0.300	2	1.7	1.8	1	N47.77967	E18.13973	102	SVK
	U2620	subsp. *subinermis*	LR	0.294	2	0.9	1.4	1	N47.77967	E18.13973	102	SVK
UP0695	U2621	subsp. *dioica*	LR	0.566	4	0.9	1.6	1	N47.80682	E18.09690	113	SVK
	U2622	subsp. *dioica*	LR	0.575	4	1.4	1.6	1	N47.80682	E18.09690	113	SVK

ID number of population	ID number of analysis	Taxon	Collector	Relative fluorescence intensi	DNA-ploidy level	CV of standard	CV of sample	N. of individuals in analysis	Latitude (WGS-84)	Longitude (WGS-84)	Altitude (m a.s.l.)	Country (ISO 3166-1)
	U2623	subsp. *dioica*	LR	0.570	4	1.3	1.6	1	N47.80682	E18.09690	113	SVK
	U2624	subsp. *dioica*	LR	0.572	4	1.2	1.5	1	N47.80682	E18.09690	113	SVK
	U2625	subsp. *subinermis*	LR	0.297	2	1.1	2.5	1	N47.80682	E18.09690	113	SVK
UP0696	U2626	subsp. *subinermis*	LR	0.296	2	0.9	2.5	1	N47.79298	E18.12825	110	SVK
	U2627	subsp. *subinermis*	LR	0.296	2	1.3	2.1	1	N47.79298	E18.12825	110	SVK
	U2628	subsp. *subinermis*	LR	0.303	2	1.2	2.2	1	N47.79298	E18.12825	110	SVK
	U2629	subsp. *subinermis*	LR	0.299	2	1.3	2.6	1	N47.79298	E18.12825	110	SVK
UP0697	U2631	subsp. *dioica*	LR	0.584	4	0.8	1.6	1	N47.84493	E17.50455	107	HUN
	U2632	subsp. *dioica*	LR	0.577	4	1.0	1.4	1	N47.84493	E17.50455	107	HUN
	U2633	subsp. *dioica*	LR	0.565	4	1.3	1.3	1	N47.84493	E17.50455	107	HUN
	U2634	subsp. *dioica*	LR	0.571	4	1.1	1.5	1	N47.84493	E17.50455	107	HUN
	U2635	subsp. *dioica*	LR	0.571	4	1.8	1.5	1	N47.84493	E17.50455	107	HUN
UP0698	U2636	subsp. *dioica*	LR	0.583	4	1.0	1.5	1	N47.96305	E17.34648	140	HUN
	U2637	subsp. *dioica*	LR	0.565	4	1.0	1.2	1	N47.96305	E17.34648	140	HUN
	U2638	subsp. *dioica*	LR	0.565	4	0.9	1.7	1	N47.96305	E17.34648	140	HUN
	U2639	subsp. *dioica*	LR	0.558	4	1.5	1.5	1	N47.96305	E17.34648	140	HUN
	U2640	subsp. *dioica*	LR	0.579	4	1.2	1.5	1	N47.96305	E17.34648	140	HUN
	U2641	subsp. *dioica*	LR	0.580	4	1.6	1.7	1	N47.96305	E17.34648	140	HUN
UP0699	U2642	subsp. *dioica*	LR	0.591	4	0.9	1.5	1	N47.97180	E17.36667	128	SVK
	U2643	subsp. *dioica*	LR	0.581	4	1.5	4.2	1	N47.97180	E17.36667	128	SVK
	U2644	subsp. *dioica*	LR	0.579	4	1.3	1.7	1	N47.97180	E17.36667	128	SVK
	U2645	subsp. *dioica*	LR	0.579	4	1.1	1.3	1	N47.97180	E17.36667	128	SVK
	U2646	subsp. *dioica*	LR	0.574	4	1.0	1.2	1	N47.97180	E17.36667	128	SVK

ID number of population	ID number of analysis	Taxon	Collector	Relative fluorescence intensi	DNA-ploidy level	CV of standard	CV of sample	N. of individuals in analysis	Latitude (WGS-84)	Longitude (WGS-84)	Altitude (m a.s.l.)	Country (ISO 3166-1)
UP0700	U2647	subsp. *dioica*	LR	0.569	4	1.0	1.4	1	N47.97500	E17.36198	129	SVK
	U2648	subsp. *dioica*	LR	0.576	4	0.9	1.4	1	N47.97500	E17.36198	129	SVK
	U2649	subsp. *dioica*	LR	0.581	4	0.9	1.3	1	N47.97500	E17.36198	129	SVK
	U2650	subsp. *dioica*	LR	0.579	4	1.4	1.3	1	N47.97500	E17.36198	129	SVK
	U2651	subsp. *dioica*	LR	0.569	4	1.2	1.5	1	N47.97500	E17.36198	129	SVK
UP0701	U2652	subsp. *dioica*	LR	0.571	4	1.7	1.7	1	N47.97800	E17.35777	117	SVK
	U2653	subsp. *dioica*	LR	0.567	4	1.3	4.2	1	N47.97800	E17.35777	117	SVK
	U2654	subsp. *dioica*	LR	0.584	4	1.3	1.8	1	N47.97800	E17.35777	117	SVK
	U2655	subsp. *dioica*	LR	0.575	4	1.1	1.4	1	N47.97800	E17.35777	117	SVK
	U2656	subsp. *dioica*	LR	0.564	4	0.8	1.2	1	N47.97800	E17.35777	117	SVK
	U2657	subsp. *dioica*	LR	0.568	4	1.0	1.6	1	N47.97800	E17.35777	117	SVK
UP0702	U2658	subsp. *dioica*	LR	0.569	4	1.1	1.9	1	N47.90598	E17.45193	125	SVK
	U2659	subsp. *dioica*	LR	0.567	4	0.8	1.3	1	N47.90598	E17.45193	125	SVK
	U2660	subsp. *dioica*	LR	0.568	4	1.5	1.2	1	N47.90598	E17.45193	125	SVK
	U2661	subsp. *dioica*	LR	0.573	4	0.9	1.5	1	N47.90598	E17.45193	125	SVK
	U2662	subsp. *dioica*	LR	0.573	4	1.0	1.3	1	N47.90598	E17.45193	125	SVK
	U2663	subsp. *dioica*	LR	0.566	4	1.7	1.3	1	N47.90598	E17.45193	98	SVK
	U2664	subsp. *dioica*	LR	0.571	4	0.9	1.3	1	N47.90598	E17.45193	98	SVK
UP0703	U2665	subsp. *dioica*	PV, RB, TU	0.577	4	1.5	1.8	1	N37.75986	E46.51035	2910	IRN
	U2666	subsp. *dioica*	PV, RB, TU	0.575	4	1.1	2.4	1	N37.75986	E46.51035	2910	IRN
	U2667	subsp. *dioica*	PV, RB, TU	0.572	4	1.3	3.2	1	N37.75986	E46.51035	2910	IRN
	U2669	subsp. *dioica*	PV, RB, TU	0.567	4	0.9	2.3	1	N37.75986	E46.51035	2910	IRN
	U2670	subsp. *dioica*	PV, RB, TU	0.570	4	1.3	3.0	1	N37.75986	E46.51035	2910	IRN

ID number of population	ID number of analysis	Taxon	Collector	Relative fluorescence intensi	DNA-ploidy level	CV of standard	CV of sample	N. of individuals in analysis	Latitude (WGS-84)	Longitude (WGS-84)	Altitude (m a.s.l.)	Country (ISO 3166-1)
	U2671	subsp. *dioica*	PV, RB, TU	0.581	4	1.0	3.3	1	N37.75986	E46.51035	2910	IRN
UP0704	U2672	subsp. *dioica*	PV, RB, TU	0.583	4	1.5	3.4	1	N38.10666	E48.13635	1472	IRN
	U2673	subsp. *dioica*	PV, RB, TU	0.569	4	1.3	2.9	1	N38.10666	E48.13635	1472	IRN
	U2674	subsp. *dioica*	PV, RB, TU	0.602	4	1.1	3.6	1	N38.10666	E48.13635	1472	IRN
	U2675	subsp. *dioica*	PV, RB, TU	0.594	4	1.3	3.8	1	N38.10666	E48.13635	1472	IRN
	U2676	subsp. *dioica*	PV, RB, TU	0.592	4	1.0	2.2	1	N38.10666	E48.13635	1472	IRN
UP0705	U2678	subsp. *dioica*	PV, RB, TU	0.583	4	1.2	3.4	1	N38.21084	E47.87413	3090	IRN
	U2679	subsp. *dioica*	PV, RB, TU	0.570	4	1.2	3.3	1	N38.21084	E47.87413	3090	IRN
	U2681	subsp. *dioica*	PV, RB, TU	0.581	4	1.4	2.4	1	N38.21084	E47.87413	3090	IRN
	U2682	subsp. *dioica*	PV, RB, TU	0.574	4	1.6	2.6	1	N38.21084	E47.87413	3090	IRN
UP0706	U2683	subsp. *dioica*	PV, RB, TU	0.589	4	1.2	1.9	1	N38.11178	E47.94624	1952	IRN
UP0707	U2685	subsp. *dioica*	PV, RB, TU	0.595	4	1.1	3.5	1	N38.33353	E47.96654	2662	IRN
	U2687	subsp. *dioica*	PV, RB, TU	0.590	4	1.4	4.0	1	N38.33353	E47.96654	2662	IRN
	U2688	subsp. *dioica*	PV, RB, TU	0.583	4	1.3	3.0	1	N38.33353	E47.96654	2662	IRN
UP0708	U2692	subsp. *dioica*	PV, RB, TU	0.581	4	1.4	3.4	1	N38.32907	E47.96724	2548	IRN
UP0709	U2693	subsp. *dioica*	PV, RB, TU	0.586	4	1.5	3.1	1	N37.56881	E48.60270	1935	IRN
	U2694	subsp. *dioica*	PV, RB, TU	0.578	4	1.3	3.3	1	N37.56881	E48.60270	1935	IRN
	U2695	subsp. *dioica*	PV, RB, TU	0.581	4	1.1	2.3	1	N37.56881	E48.60270	1935	IRN
	U2696	subsp. *dioica*	PV, RB, TU	0.592	4	1.1	4.3	1	N37.56881	E48.60270	1935	IRN
UP0710	U2697	subsp. *dioica*	PV, RB, TU	0.583	4	1.5	2.9	1	N37.57083	E48.67630	2273	IRN
	U2698	subsp. *dioica*	PV, RB, TU	0.606	4	1.2	3.4	1	N37.57083	E48.67630	2273	IRN
	U2699	subsp. *dioica*	PV, RB, TU	0.576	4	1.6	3.8	1	N37.57083	E48.67630	2273	IRN
	U2700	subsp. *dioica*	PV, RB, TU	0.566	4	1.4	3.5	1	N37.57083	E48.67630	2273	IRN

ID number of population	ID number of analysis	Taxon	Collector	Relative fluorescence intensi	DNA-ploidy level	CV of standard	CV of sample	N. of individuals in analysis	Latitude (WGS-84)	Longitude (WGS-84)	Altitude (m a.s.l.)	Country (ISO 3166-1)
UP0711	U2701	subsp. *dioica*	PV, RB, TU	0.577	4	1.2	2.3	1	N37.16932	E48.92923	2110	IRN
	U2702	subsp. *dioica*	PV, RB, TU	0.592	4	1.1	4.3	1	N37.16932	E48.92923	2110	IRN
	U2703	subsp. *dioica*	PV, RB, TU	0.600	4	1.1	4.7	1	N37.16932	E48.92923	2110	IRN
	U2704	subsp. *dioica*	PV, RB, TU	0.579	4	1.8	2.8	1	N37.16932	E48.92923	2110	IRN
	U2705	subsp. *dioica*	PV, RB, TU	0.580	4	1.7	2.9	1	N37.16932	E48.92923	2110	IRN
UP0712	U2707	subsp. *dioica*	PV, RB, TU	0.592	4	1.7	3.6	1	N37.17198	E48.97948	1450	IRN
UP0713	U2708	subsp. *dioica*	PV, RB, TU	0.599	4	1.4	4.4	1	N37.19984	E49.21139	125	IRN
UP0714	U2711	subsp. *dioica*	PV, RB, TU	0.575	4	1.3	3.4	1	N36.85701	E50.48173	1726	IRN
	U2712	subsp. *dioica*	PV, RB, TU	0.590	4	1.9	3.5	1	N36.85701	E50.48173	1726	IRN
UP0715	U2713	subsp. *dioica*	PV, RB, TU	0.600	4	1.4	4.1	1	N36.35899	E52.35977	246	IRN
	U2714	subsp. *dioica*	PV, RB, TU	0.604	4	1.6	3.1	1	N36.35899	E52.35977	246	IRN
UP0716	U2717	subsp. *dioica*	PV, RB, TU	0.598	4	1.3	3.5	1	N36.28833	E52.88775	249	IRN
	U2718	subsp. *dioica*	PV, RB, TU	0.601	4	1.7	3.5	1	N36.28833	E52.88775	249	IRN
	U2719	subsp. *dioica*	PV, RB, TU	0.578	4	1.5	3.1	1	N36.28833	E52.88775	249	IRN
UP0717	U2720	subsp. *dioica*	PV, RB, TU	0.584	4	1.0	2.9	1	N36.06619	E53.15799	1502	IRN
	U2721	subsp. *dioica*	PV, RB, TU	0.604	4	1.4	3.3	1	N36.06619	E53.15799	1502	IRN
UP0718	U2723	subsp. *dioica*	PV, RB, TU	0.590	4	1.6	4.3	1	N35.80444	E52.29733	2878	IRN
	U2724	subsp. *dioica*	PV, RB, TU	0.580	4	1.9	4.9	1	N35.80444	E52.29733	2878	IRN
	U2725	subsp. *dioica*	PV, RB, TU	0.574	4	1.3	3.0	1	N35.80444	E52.29733	2878	IRN
	U2726	subsp. *dioica*	PV, RB, TU	0.584	4	1.3	2.9	1	N35.80444	E52.29733	2878	IRN
	U2727	subsp. *dioica*	PV, RB, TU	0.591	4	1.3	3.5	1	N35.80444	E52.29733	2878	IRN
UP0719	U2728	subsp. *dioica*	PV, RB, TU	0.577	4	1.8	2.7	1	N35.80882	E52.28233	2968	IRN
	U2729	subsp. *dioica*	PV, RB, TU	0.588	4	1.2	2.4	1	N35.80882	E52.28233	2968	IRN

ID number of population	ID number of analysis	Taxon	Collector	Relative fluorescence intensi	DNA-ploidy level	CV of standard	CV of sample	N. of individuals in analysis	Latitude (WGS-84)	Longitude (WGS-84)	Altitude (m a.s.l.)	Country (ISO 3166-1)
	U2730	subsp. *dioica*	PV, RB, TU	0.598	4	1.3	2.6	1	N35.80882	E52.28233	2968	IRN
	U2731	subsp. *dioica*	PV, RB, TU	0.596	4	1.2	3.5	1	N35.80882	E52.28233	2968	IRN
	U2732	subsp. *dioica*	PV, RB, TU	0.624	4	2.2	2.9	1	N35.80882	E52.28233	2968	IRN
	U2733	subsp. *dioica*	PV, RB, TU	0.583	4	1.1	1.9	1	N35.80882	E52.28233	2968	IRN
UP0720	U2734	subsp. *dioica*	PV, RB, TU	0.595	4	1.4	3.7	1	N36.20252	E51.92603	1925	IRN
	U2735	subsp. *dioica*	PV, RB, TU	0.586	4	1.2	4.7	1	N36.20252	E51.92603	1925	IRN
UP0721	U2736	subsp. *dioica*	PV, RB, TU	0.613	4	1.6	4.3	1	N36.23832	E51.43928	3140	IRN
	U2737	subsp. *dioica*	PV, RB, TU	0.593	4	1.4	3.4	1	N36.23832	E51.43928	3140	IRN
	U2738	subsp. *dioica*	PV, RB, TU	0.609	4	1.1	4.6	1	N36.23832	E51.43928	3140	IRN
	U2739	subsp. *dioica*	PV, RB, TU	0.585	4	1.4	2.9	1	N36.23832	E51.43928	3140	IRN
	U2740	subsp. *dioica*	PV, RB, TU	0.570	4	1.5	3.3	1	N36.23832	E51.43928	3140	IRN
	U2741	subsp. *dioica*	PV, RB, TU	0.574	4	1.7	2.6	1	N36.23832	E51.43928	3140	IRN
UP0722	U2742	subsp. *dioica*	PV, RB, TU	0.614	4	1.9	3.2	1	N36.17365	E51.31466	2565	IRN
	U2743	subsp. *dioica*	PV, RB, TU	0.578	4	1.1	2.5	1	N36.17365	E51.31466	2565	IRN
	U2744	subsp. *dioica*	PV, RB, TU	0.613	4	1.7	4.0	1	N36.17365	E51.31466	2565	IRN
UP0723	U2745	subsp. *dioica*	PV, RB, TU	0.596	4	1.3	1.3	1	N36.05601	E51.40791	2604	IRN
UP0724	U2748	subsp. *dioica*	RB, TU	0.566	4	1.3	2.0	1	N41.53789	E22.13830	128	MKD
	U2749	subsp. *dioica*	RB, TU	0.571	4	1.1	1.8	1	N41.53789	E22.13830	128	MKD
	U2750	subsp. *dioica*	RB, TU	0.570	4	1.5	1.8	1	N41.53789	E22.13830	128	MKD
UP0725	U2751	subsp. *dioica*	RB, TU	0.598	4	1.2	1.9	1	N41.19679	E22.77564	146	GRC
	U2752	subsp. *dioica*	RB, TU	0.597	4	1.5	1.9	1	N41.19679	E22.77564	146	GRC
	U2753	subsp. *dioica*	RB, TU	0.589	4	0.9	1.6	1	N41.19679	E22.77564	146	GRC
UP0726	U2754	subsp. *dioica*	RB, TU	0.571	4	1.2	1.9	1	N41.44564	E23.27741	86	BGR

ID number of population	ID number of analysis	Taxon	Collector	Relative fluorescence intensi	DNA-ploidy level	CV of standard	CV of sample	N. of individuals in analysis	Latitude (WGS-84)	Longitude (WGS-84)	Altitude (m a.s.l.)	Country (ISO 3166-1)
	U2767	subsp. *subinermis*	RB, TU	0.297	2	0.9	2.5	1	N41.44564	E23.27741	86	BGR
	U2768	subsp. *subinermis*	RB, TU	0.302	2	0.9	2.8	1	N41.44564	E23.27741	86	BGR
	U2873	subsp. *dioica*	RB, TU	0.585	4	1.1	2.6	1	N41.44564	E23.27741	86	BGR
	U2874	subsp. *subinermis*	RB, TU	0.309	2	0.8	3.4	1	N41.44564	E23.27741	86	BGR
UP0727	U2755	subsp. *dioica*	RB, TU	0.568	4	0.9	1.4	2	N41.52702	E23.40403	454	BGR
	U2756	subsp. *dioica*	RB, TU	0.568	4	0.9	1.4	2	N41.52702	E23.40403	454	BGR
	U2761	subsp. *dioica*	RB, TU	0.577	4	1.2	1.7	1	N41.52702	E23.40403	454	BGR
UP0728	U2757	subsp. *dioica*	RB, TU	0.564	4	1.2	1.5	1	N42.13247	E23.33359	1236	BGR
	U2758	subsp. *dioica*	RB, TU	0.561	4	1.0	1.5	1	N42.13247	E23.33359	1236	BGR
UP0729	U2759	subsp. *dioica*	RB, TU	0.567	4	1.2	1.9	1	N44.69285	E20.58211	116	SRB
	U2766	subsp. *dioica*	RB, TU	0.567	4	1.2	1.9	1	N44.69285	E20.58211	116	SRB
UP0730	U2760	subsp. *dioica*	RB, TU	0.583	4	1.2	1.2	2	N41.40521	E22.26003	133	MKD
	U2762	subsp. *dioica*	RB, TU	0.561	4	1.2	2.2	1	N41.40521	E22.26003	133	MKD
	U2769	subsp. *dioica*	RB, TU	0.583	4	1.2	1.2	2	N41.40521	E22.26003	133	MKD
UP0731	U2763	subsp. *subinermis*	RB, TU	0.297	2	0.9	1.8	1	N42.42413	E21.73910	399	SRB
UP0732	U2764	subsp. *dioica*	RB, TU	0.570	4	1.2	1.8	3	N43.33701	E22.08378	300	SRB
UP0733	U2765	subsp. *dioica*	RB, TU	0.571	4	1.4	1.7	3	N44.69344	E20.01966	72	SRB
UP0734	U2770	subsp. *dioica*	JH, ZK	0.576	4	1.2	1.2	1	N42.79472	E13.07389	551	ITA
	U2771	subsp. *dioica*	JH, ZK	0.594	4	0.9	1.2	1	N42.79472	E13.07389	551	ITA
	U2772	subsp. *dioica*	JH, ZK	0.570	4	0.8	1.2	1	N42.79472	E13.07389	551	ITA
	U2773	subsp. *dioica*	JH, ZK	0.571	4	0.8	1.2	1	N42.79472	E13.07389	551	ITA
	U2789	subsp. *dioica*	JH, ZK	0.584	4	0.9	1.2	1	N42.79472	E13.07389	551	ITA
UP0735	U2779	subsp. *pubescens*	JH, ZK	0.321	2	0.6	1.7	1	N39.35428	E9.55250	8	ITA

ID number of population	ID number of analysis	Taxon	Collector	Relative fluorescence intensi	DNA-ploidy level	CV of standard	CV of sample	N. of individuals in analysis	Latitude (WGS-84)	Longitude (WGS-84)	Altitude (m a.s.l.)	Country (ISO 3166-1)
	U2780	subsp. *pubescens*	JH, ZK	0.319	2	0.8	2.0	1	N39.35428	E9.55250	8	ITA
	U2783	subsp. *pubescens*	JH, ZK	0.319	2	0.8	1.4	1	N39.35428	E9.55250	8	ITA
UP0736	U2790	subsp. *dioica*	JH, ZK	0.588	4	0.7	1.2	1	N41.20500	E13.79417	4	ITA
	U2791	subsp. *dioica*	JH, ZK	0.593	4	0.8	1.6	1	N41.20500	E13.79417	4	ITA
	U2792	subsp. *dioica*	JH, ZK	0.583	4	0.9	1.3	1	N41.20500	E13.79417	4	ITA
	U2793	subsp. *dioica*	JH, ZK	0.590	4	0.9	1.0	1	N41.20500	E13.79417	4	ITA
	U2794	subsp. *dioica*	JH, ZK	0.601	4	0.8	1.2	1	N41.20500	E13.79417	4	ITA
UP0737	U2798	subsp. *dioica*	CP	0.614	4	0.8	2.9	1	N48.21922	E16.22628	280	AUT
	U2799	subsp. *dioica*	CP	0.614	4	0.8	2.0	1	N48.21922	E16.22628	280	AUT
	U2800	subsp. *dioica*	CP	0.606	4	1.0	2.2	1	N48.21922	E16.22628	280	AUT
UP0738	U2801	subsp. *dioica*	CP	0.594	4	0.8	1.2	1	N48.39322	E15.68928	208	AUT
	U2802	subsp. *dioica*	CP	0.610	4	0.8	2.3	1	N48.39322	E15.68928	208	AUT
	U2803	subsp. *dioica*	CP	0.593	4	1.1	1.9	1	N48.39322	E15.68928	208	AUT
	U2804	subsp. *dioica*	CP	0.585	4	1.0	1.6	1	N48.39322	E15.68928	208	AUT
	U2805	subsp. *dioica*	CP	0.598	4	1.0	1.4	1	N48.39322	E15.68928	208	AUT
	U2806	subsp. *dioica*	CP	0.588	4	0.8	2.0	1	N48.39322	E15.68928	208	AUT
	U2807	subsp. *dioica*	CP	0.586	4	1.8	2.1	1	N48.39322	E15.68928	208	AUT
UP0739	U2808	subsp. *dioica*	CP	0.597	4	1.2	3.7	1	N48.38997	E15.69161	199	AUT
	U2809	subsp. *dioica*	CP	0.579	4	1.1	2.2	1	N48.38997	E15.69161	199	AUT
	U2810	subsp. *dioica*	CP	0.568	4	0.9	1.7	1	N48.38997	E15.69161	199	AUT
	U2812	subsp. *dioica*	CP	0.585	4	0.9	1.9	1	N48.38997	E15.69161	199	AUT
	U2813	subsp. *dioica*	CP	0.579	4	1.1	2.6	1	N48.38997	E15.69161	199	AUT
	U2814	subsp. *dioica*	CP	0.580	4	0.9	2.5	1	N48.38997	E15.69161	199	AUT

ID number of population	ID number of analysis	Taxon	Collector	Relative fluorescence intensi	DNA-ploidy level	CV of standard	CV of sample	N. of individuals in analysis	Latitude (WGS-84)	Longitude (WGS-84)	Altitude (m a.s.l.)	Country (ISO 3166-1)
	U2816	subsp. *dioica*	CP	0.565	4	0.9	1.9	1	N48.38997	E15.69161	199	AUT
	U2817	subsp. *dioica*	CP	0.588	4	0.9	2.7	1	N48.38997	E15.69161	199	AUT
	U2818	subsp. *dioica*	CP	0.588	4	0.7	2.0	1	N48.38997	E15.69161	199	AUT
UP0740	U2819	subsp. *dioica*	FK	0.559	4	0.7	1.4	1	N48.38924	E15.68373	200	AUT
UP0741	U2820	subsp. *dioica*	JP	0.574	4	1.2	1.4	1	N52.09506	W2.33837	313	GBR
UP0742	U2821	subsp. *dioica*	JP	0.573	4	1.1	1.5	1	N52.11168	W2.34012	313	GBR
UP0743	U2822	subsp. *dioica*	JP	0.564	4	0.9	1.4	1	N52.11189	W2.33459	313	GBR
UP0744	U2824	subsp. *dioica*	OK	0.579	4	1.0	1.4	1	N45.52917	E25.37028	725	ROU
UP0745	U2825	subsp. *dioica*	OK	0.564	4	1.4	1.4	1	N45.17806	E23.78583	522	ROU
	U2826	subsp. *dioica*	OK	0.561	4	1.8	1.4	1	N45.17806	E23.78583	522	ROU
UP0746	U2827	subsp. *dioica*	GF	0.581	4	1.3	1.9	5	N52.62146	E1.23149	10	GBR
UP0747	U2830	subsp. *dioica*	RB, TU	0.586	4	1.0	1.8	1	N47.64449	E17.59997	116	HUN
UP0748	U2832	subsp. *dioica*	RB, TU	0.587	4	1.1	1.7	1	N45.85720	E18.43004	171	HUN
	U2833	subsp. *dioica*	RB, TU	0.559	4	1.2	1.9	1	N45.85720	E18.43004	171	HUN
UP0749	U2834	subsp. *dioica*	RB, TU	0.568	4	1.2	1.8	2	N46.21278	E18.19086	229	HUN
UP0750	U2835	subsp. *dioica*	RB, TU	0.585	4	1.3	1.7	4	N46.35242	E17.69505	148	HUN
UP0751	U2836	subsp. *subinermis*	RB, TU	0.303	2	1.1	2.4	1	N46.70164	E17.25790	110	HUN
	U2837	subsp. *subinermis*	RB, TU	0.306	2	1.1	3.0	1	N46.70164	E17.25790	110	HUN
	U2838	subsp. *dioica*	RB, TU	0.587	4	1.0	2.0	1	N46.70164	E17.25790	110	HUN
	U2839	subsp. *dioica*	RB, TU	0.573	4	1.1	1.4	1	N46.70164	E17.25790	110	HUN
	U2840	subsp. *dioica*	RB, TU	0.581	4	1.2	1.8	1	N46.70164	E17.25790	110	HUN
UP0752	U2841	subsp. *dioica*	RB, TU	0.571	4	1.1	1.5	2	N46.68832	E16.72637	197	HUN
UP0753	U2842	subsp. *dioica*	RB, TU	0.575	4	0.9	1.6	1	N46.73830	E16.63762	230	HUN

ID number of population	ID number of analysis	Taxon	Collector	Relative fluorescence intensi	DNA-ploidy level	CV of standard	CV of sample	N. of individuals in analysis	Latitude (WGS-84)	Longitude (WGS-84)	Altitude (m a.s.l.)	Country (ISO 3166-1)
UP0754	U2843	subsp. *dioica*	RB, TU	0.566	4	1.1	1.8	1	N47.00717	E16.61203	191	HUN
	U2844	subsp. *dioica*	RB, TU	0.577	4	1.1	1.9	1	N47.00717	E16.61203	191	HUN
	U2845	subsp. *dioica*	RB, TU	0.574	4	1.1	1.4	1	N47.00717	E16.61203	191	HUN
	U2846	subsp. *dioica*	RB, TU	0.578	4	1.0	1.7	1	N47.00717	E16.61203	191	HUN
UP0755	U2847	subsp. *dioica*	RB, TU	0.567	4	1.1	1.8	2	N47.23156	E17.11906	214	HUN
UP0756	U2848	subsp. *subinermis*	RB, TU	0.306	2	1.3	3.2	1	N47.73218	E18.31424	114	HUN
	U2849	subsp. *dioica*	RB, TU	0.581	4	1.3	1.7	1	N47.73218	E18.31424	114	HUN
UP0757	U2850	subsp. *dioica*	RB, TU	0.584	4	1.0	1.3	1	N47.76276	E18.69994	105	HUN
UP0758	U2851	subsp. *dioica*	RB, TU	0.564	4	1.0	1.4	2	N47.66878	E18.95017	221	HUN
UP0759	U2852	subsp. *dioica*	RB, TU	0.576	4	1.2	1.7	5	N47.40110	E19.02083	114	HUN
UP0760	U2853	subsp. *dioica*	RB, TU	0.569	4	1.0	2.0	2	N47.10633	E19.36230	113	HUN
UP0761	U2854	subsp. *dioica*	RB, TU	0.573	4	1.2	1.7	1	N46.81113	E18.93695	98	HUN
	U2855	subsp. *dioica*	RB, TU	0.579	4	1.3	1.7	1	N46.81113	E18.93695	98	HUN
	U2856	subsp. *dioica*	RB, TU	0.574	4	1.3	1.3	1	N46.81113	E18.93695	98	HUN
	U2857	subsp. *dioica*	RB, TU	0.569	4	1.3	1.9	1	N46.81113	E18.93695	98	HUN
	U2858	subsp. *dioica*	RB, TU	0.565	4	1.0	1.9	1	N46.81113	E18.93695	98	HUN
	U2859	subsp. *dioica*	RB, TU	0.569	4	1.4	2.0	1	N46.81113	E18.93695	98	HUN
	U2860	subsp. *dioica*	RB, TU	0.585	4	1.0	1.7	1	N46.81113	E18.93695	98	HUN
	U2861	subsp. *dioica*	RB, TU	0.594	4	1.3	1.3	1	N46.81113	E18.93695	98	HUN
	U2862	subsp. *dioica* pentaploid	RB, TU	0.726	5	1.0	1.1	1	N46.81113	E18.93695	98	HUN
UP0762	U2863	subsp. *dioica*	RB, TU	0.578	4	1.3	1.9	2	N46.41502	E18.79731	88	HUN
UP0763	U2864	subsp. *dioica*	RB, TU	0.569	4	1.2	1.5	1	N46.17097	E18.32557	408	HUN
	U2865	subsp. *dioica*	RB, TU	0.580	4	1.2	1.9	1	N46.17097	E18.32557	408	HUN

ID number of population	ID number of analysis	Taxon	Collector	Relative fluorescence intensi	DNA-ploidy level	CV of standard	CV of sample	N. of individuals in analysis	Latitude (WGS-84)	Longitude (WGS-84)	Altitude (m a.s.l.)	Country (ISO 3166-1)
UP0764	U2866	subsp. *subinermis*	RB, TU	0.304	2	1.3	3.0	1	N48.17976	E16.97722	152	AUT
	U2867	subsp. *subinermis*	RB, TU	0.301	2	1.2	3.1	1	N48.17976	E16.97722	152	AUT
	U2868	subsp. *dioica*	RB, TU	0.575	4	1.4	2.1	1	N48.17976	E16.97722	152	AUT
	U2869	subsp. *subinermis*	RB, TU	0.302	2	1.3	2.7	1	N48.17976	E16.97722	152	AUT
UP0765	U2871	subsp. *dioica*	GF	0.585	4	0.6	1.6	4	N54.37762	E19.42514	10	POL
UP0766	U2872	subsp. *dioica*	GF	0.571	4	0.8	1.5	1	N51.67240	E15.71750	114	POL
UP0767	U2877	subsp. *dioica*	LR	0.565	4	1.2	1.7	1	N52.37696	E4.88763	13	NLD
	U2878	subsp. *dioica*	LR	0.565	4	1.2	1.7	1	N52.37696	E4.88763	13	NLD
UP0768	U2879	subsp. *dioica*	LR	0.568	4	0.9	1.4	1	N52.37532	E4.91007	1	NLD
	U2880	subsp. *dioica*	LR	0.568	4	0.9	1.4	1	N52.37532	E4.91007	1	NLD
	U2881	subsp. *dioica*	LR	0.568	4	0.9	1.4	1	N52.37532	E4.91007	1	NLD
UP0769	U2882	subsp. *dioica*	LR	0.569	4	1.0	1.4	1	N52.11840	E4.29253	23	NLD
	U2883	subsp. *dioica*	LR	0.569	4	1.0	1.4	1	N52.11840	E4.29253	23	NLD
	U2884	subsp. *dioica*	LR	0.569	4	1.0	1.4	1	N52.11840	E4.29253	23	NLD
UP0770	U2885	subsp. *dioica* pentaploid	GF	0.711	5	0.7	0.8	1	N50.59951	E14.03729	224	CZE
	U3185	subsp. *dioica*	GF	0.563	4	0.7	1.1	1	N50.59951	E14.03729	224	CZE
UP0771	U2905	subsp. *dioica*	FK	0.580	4	0.7	2.3	1	N41.66000	E43.65279	2000	GEO
UP0772	U2917	subsp. *subinermis*	TU	0.301	2	0.5	1.6	1	N42.80928	E9.48922	7	FRA
	U2918	subsp. *subinermis*	TU	0.313	2	1.3	3.9	1	N42.80928	E9.48922	7	FRA
UP0773	U2919	subsp. *subinermis*	TU	0.296	2	0.8	1.5	1	N42.48634	E9.28053	150	FRA
UP0774	U2920	*U. atrovirens*	TU	0.306	2	0.8	2.7	1	N42.30477	E9.15027	460	FRA
	U2921	*U. atrovirens*	TU	0.310	2	0.8	1.7	1	N42.30477	E9.15027	460	FRA
UP0775	U2922	subsp. *subinermis*	TU	0.316	2	1.0	3.3	1	N42.30138	E9.15204	410	FRA

ID number of population	ID number of analysis	Taxon	Collector	Relative fluorescence intensi	DNA-ploidy level	CV of standard	CV of sample	N. of individuals in analysis	Latitude (WGS-84)	Longitude (WGS-84)	Altitude (m a.s.l.)	Country (ISO 3166-1)
	U2923	subsp. *subinermis*	TU	0.320	2	1.2	3.2	1	N42.30138	E9.15204	410	FRA
UP0776	U2924	*U. atrovirens*	TU	0.311	2	0.8	2.8	1	N42.37303	E9.14494	440	FRA
	U2925	*U. atrovirens*	TU	0.311	2	0.8	1.7	1	N42.37303	E9.14494	440	FRA
UP0777	U2927	*U. atrovirens*	TU	0.311	2	0.7	2.3	1	N42.24014	E8.84380	790	FRA
	U2928	*U. atrovirens*	TU	0.316	2	0.7	1.5	1	N42.24014	E8.84380	790	FRA
UP0778	U2930	subsp. *subinermis*	LR	0.309	2	1.2	3.0	1	N48.77615	E12.93960	331	DEU
	U2931	subsp. *subinermis*	LR	0.297	2	0.8	1.9	1	N48.77615	E12.93960	331	DEU
	U2932	subsp. *dioica*	LR	0.593	4	1.4	2.0	1	N48.77615	E12.93960	331	DEU
	U2933	subsp. *subinermis*	LR	0.306	2	1.4	2.5	1	N48.77615	E12.93960	331	DEU
	U2934	subsp. *subinermis*	LR	0.301	2	1.0	2.4	1	N48.77615	E12.93960	331	DEU
	U2935	subsp. *subinermis*	LR	0.290	2	1.2	4.4	1	N48.77615	E12.93960	331	DEU
	U2936	subsp. *subinermis*	LR	0.302	2	0.9	3.1	1	N48.77615	E12.93960	331	DEU
	U2937	subsp. *subinermis*	LR	0.297	2	1.4	2.9	1	N48.77615	E12.93960	331	DEU
	U2938	subsp. *subinermis*	LR	0.309	2	1.2	2.8	1	N48.77615	E12.93960	331	DEU
UP0779	U2939	subsp. *dioica*	LR	0.571	4	1.1	1.4	1	N48.98442	E12.44242	331	DEU
	U2940	subsp. *subinermis*	LR	0.300	2	1.4	2.8	1	N48.98442	E12.44242	331	DEU
	U2941	subsp. *dioica*	LR	0.567	4	0.8	1.1	1	N48.98442	E12.44242	331	DEU
	U2942	subsp. *dioica*	LR	0.580	4	0.8	1.6	1	N48.98442	E12.44242	331	DEU
	U2943	subsp. *dioica*	LR	0.579	4	1.0	1.1	1	N48.98442	E12.44242	331	DEU
UP0780	U2944	subsp. *dioica*	LR	0.581	4	1.2	1.6	1	N49.05863	E11.95813	335	DEU
	U2945	subsp. *dioica*	LR	0.569	4	1.0	1.6	1	N49.05863	E11.95813	335	DEU
	U2946	subsp. *dioica*	LR	0.573	4	1.2	1.4	1	N49.05863	E11.95813	335	DEU
UP0781	U2947	subsp. *dioica*	LR	0.577	4	1.1	1.9	1	N49.39000	E10.84357	281	DEU

ID number of population	ID number of analysis	Taxon	Collector	Relative fluorescence intensi	DNA-ploidy level	CV of standard	CV of sample	N. of individuals in analysis	Latitude (WGS-84)	Longitude (WGS-84)	Altitude (m a.s.l.)	Country (ISO 3166-1)
	U2948	subsp. *dioica*	LR	0.574	4	1.2	1.9	1	N49.39000	E10.84357	281	DEU
	U2949	subsp. *dioica*	LR	0.569	4	1.0	1.7	1	N49.39000	E10.84357	281	DEU
	U2950	subsp. *dioica*	LR	0.573	4	0.9	1.4	1	N49.39000	E10.84357	281	DEU
	U2951	subsp. *dioica*	LR	0.565	4	1.2	1.9	1	N49.39000	E10.84357	281	DEU
UP0782	U2952	subsp. *dioica*	LR	0.561	4	0.8	1.7	1	N49.97610	E10.17870	209	DEU
	U2953	subsp. *dioica*	LR	0.567	4	1.2	1.6	1	N49.97610	E10.17870	209	DEU
	U2954	subsp. *dioica*	LR	0.570	4	1.1	1.7	1	N49.97610	E10.17870	209	DEU
	U2955	subsp. *dioica*	LR	0.574	4	1.0	1.4	1	N49.97610	E10.17870	209	DEU
	U2956	subsp. *subinermis*	LR	0.305	2	1.4	2.9	1	N49.97610	E10.17870	209	DEU
	U2978	subsp. *subinermis*	LR	0.295	2	0.9	2.4	1	N49.97610	E10.17870	209	DEU
UP0783	U2957	subsp. *dioica*	LR	0.562	4	1.1	1.4	1	N49.76927	E9.34690	121	DEU
	U2958	subsp. *dioica*	LR	0.560	4	1.2	1.4	1	N49.76927	E9.34690	121	DEU
	U2959	subsp. *dioica*	LR	0.558	4	1.2	1.4	1	N49.76927	E9.34690	121	DEU
	U2960	subsp. *dioica*	LR	0.574	4	1.2	1.7	1	N49.76927	E9.34690	121	DEU
UP0784	U2961	subsp. *dioica*	LR	0.581	4	1.1	1.7	1	N49.81522	E8.45752	94	DEU
	U2962	subsp. *dioica*	LR	0.553	4	1.2	1.4	1	N49.81522	E8.45752	94	DEU
	U2963	subsp. *dioica*	LR	0.572	4	1.4	1.8	1	N49.81522	E8.45752	94	DEU
	U2964	subsp. *subinermis*	LR	0.299	2	1.3	2.6	1	N49.81522	E8.45752	94	DEU
	U2965	subsp. *dioica*	LR	0.573	4	1.0	1.4	1	N49.81522	E8.45752	94	DEU
	U2966	subsp. *dioica*	LR	0.569	4	1.3	1.7	1	N49.81522	E8.45752	94	DEU
	U2967	subsp. *dioica*	LR	0.575	4	1.2	1.6	1	N49.81522	E8.45752	94	DEU
UP0785	U2968	subsp. *dioica*	LR	0.563	4	0.9	1.4	1	N51.06280	E6.89218	29	DEU
	U2969	subsp. *dioica*	LR	0.568	4	1.0	1.3	1	N51.06280	E6.89218	29	DEU

ID number of population	ID number of analysis	Taxon	Collector	Relative fluorescence intensi	DNA-ploidy level	CV of standard	CV of sample	N. of individuals in analysis	Latitude (WGS-84)	Longitude (WGS-84)	Altitude (m a.s.l.)	Country (ISO 3166-1)
	U2970	subsp. *dioica*	LR	0.566	4	1.0	0.8	1	N51.06280	E6.89218	29	DEU
	U2971	subsp. *dioica*	LR	0.564	4	1.1	1.6	1	N51.06280	E6.89218	29	DEU
	U2972	subsp. *dioica*	LR	0.570	4	1.0	1.4	1	N51.06280	E6.89218	29	DEU
	U2973	subsp. *dioica*	LR	0.566	4	1.2	1.7	1	N51.06280	E6.89218	29	DEU
	U2974	subsp. *dioica*	LR	0.561	4	1.0	1.6	1	N51.06280	E6.89218	29	DEU
	U2975	subsp. *dioica*	LR	0.558	4	1.2	2.1	1	N51.06280	E6.89218	29	DEU
	U2976	subsp. *dioica*	LR	0.561	4	1.1	1.7	1	N51.06280	E6.89218	29	DEU
	U2977	subsp. *dioica*	LR	0.564	4	1.2	2.0	1	N51.06280	E6.89218	29	DEU
UP0786	U2979	subsp. *subinermis*	TU	0.304	2	0.6	2.1	1	N48.84767	E16.72642	160	CZE
	U3595	subsp. *subinermis*	LR	0.294	2	1.7	2.5	1	N48.84767	E16.72642	168	CZE
	U3596	subsp. *subinermis*	LR	0.292	2	1.8	2.2	1	N48.84767	E16.72642	168	CZE
	U3597	subsp. *subinermis*	LR	0.295	2	1.8	2.2	1	N48.84767	E16.72642	168	CZE
	U3682	subsp. *subinermis*	TU	0.297	2	0.6	2.2	1	N48.84767	E16.72642	160	CZE
	U3683	subsp. *subinermis*	TU	0.304	2	0.7	1.8	1	N48.84767	E16.72642	160	CZE
	U3684	subsp. *subinermis*	TU	0.294	2	0.5	2.1	1	N48.84767	E16.72642	160	CZE
	U3685	subsp. *subinermis*	TU	0.297	2	0.8	1.9	1	N48.84767	E16.72642	160	CZE
	U3686	subsp. *subinermis*	TU	0.301	2	0.8	1.8	1	N48.84767	E16.72642	160	CZE
	U3687	subsp. *subinermis*	TU	0.302	2	0.8	2.3	1	N48.84767	E16.72642	160	CZE
	U3688	subsp. *subinermis*	TU	0.297	2	0.8	2.1	1	N48.84767	E16.72642	160	CZE
	U3689	subsp. *subinermis*	TU	0.299	2	0.8	1.7	1	N48.84767	E16.72642	160	CZE
	U3690	subsp. *subinermis*	TU	0.299	2	0.8	2.0	1	N48.84767	E16.72642	160	CZE
	U3691	subsp. *subinermis*	TU	0.299	2	0.9	2.0	1	N48.84767	E16.72642	160	CZE
	U3692	subsp. *subinermis*	TU	0.297	2	0.6	2.1	1	N48.84767	E16.72642	160	CZE

ID number of population	ID number of analysis	Taxon	Collector	Relative fluorescence intensi	DNA-ploidy level	CV of standard	CV of sample	N. of individuals in analysis	Latitude (WGS-84)	Longitude (WGS-84)	Altitude (m a.s.l.)	Country (ISO 3166-1)
	U3693	subsp. *subinermis*	TU	0.308	2	1.1	2.9	1	N48.84767	E16.72642	160	CZE
	U3694	subsp. *subinermis*	TU	0.299	2	0.8	1.4	1	N48.84767	E16.72642	160	CZE
	U3695	subsp. *subinermis*	TU	0.299	2	0.7	2.1	1	N48.84767	E16.72642	160	CZE
	U3696	subsp. *subinermis*	TU	0.303	2	0.9	1.9	1	N48.84767	E16.72642	160	CZE
	U3697	subsp. *subinermis*	TU	0.303	2	1.0	1.8	1	N48.84767	E16.72642	160	CZE
	U3698	subsp. *subinermis*	TU	0.298	2	0.8	2.1	1	N48.84767	E16.72642	160	CZE
UP0787	U2981	subsp. *dioica*	CP	0.559	4	0.9	1.1	1	N41.26139	E20.53528	1820	MKD
	U2982	subsp. *dioica*	CP	0.586	4	0.7	2.4	1	N41.26139	E20.53528	1820	MKD
	U2983	subsp. *dioica*	CP	0.573	4	0.7	1.5	1	N41.26139	E20.53528	1820	MKD
UP0788	U2984	subsp. *dioica*	CP	0.580	4	0.7	2.4	1	N47.50005	E24.79859	1342	ROU
	U2985	subsp. *dioica*	CP	0.581	4	0.7	1.8	1	N47.50005	E24.79859	1342	ROU
	U2986	subsp. *dioica*	CP	0.585	4	0.7	2.3	1	N47.50005	E24.79859	1342	ROU
	U2987	subsp. *dioica*	CP	0.586	4	0.7	2.3	1	N47.50005	E24.79859	1342	ROU
	U2988	subsp. *dioica*	CP	0.582	4	0.7	1.3	1	N47.50005	E24.79859	1342	ROU
UP0789	U2989	subsp. *dioica*	CP	0.573	4	0.7	1.5	1	N47.45533	E12.37121	810	AUT
	U2990	subsp. *dioica*	CP	0.570	4	0.8	1.6	1	N47.45533	E12.37121	810	AUT
	U2991	subsp. *dioica*	CP	0.575	4	0.9	1.6	1	N47.45533	E12.37121	810	AUT
	U2992	subsp. *dioica*	CP	0.566	4	0.8	1.1	1	N47.45533	E12.37121	810	AUT
	U2993	subsp. *dioica*	CP	0.571	4	0.8	1.7	1	N47.45533	E12.37121	810	AUT
	U2994	subsp. *dioica*	CP	0.576	4	1.1	2.0	1	N47.45533	E12.37121	810	AUT
UP0790	U2995	subsp. *dioica*	CP	0.576	4	0.9	1.6	1	N48.09917	E24.47000	770	UKR
	U2996	subsp. *dioica*	CP	0.584	4	0.6	1.6	1	N48.09917	E24.47000	770	UKR
	U2997	subsp. *dioica*	CP	0.572	4	0.7	1.9	1	N48.09917	E24.47000	770	UKR

ID number of population	ID number of analysis	Taxon	Collector	Relative fluorescence intensi	DNA-ploidy level	CV of standard	CV of sample	N. of individuals in analysis	Latitude (WGS-84)	Longitude (WGS-84)	Altitude (m a.s.l.)	Country (ISO 3166-1)
	U2998	subsp. *dioica*	CP	0.586	4	0.8	1.5	1	N48.09917	E24.47000	770	UKR
UP0791	U2999	subsp. *dioica*	CP	0.581	4	0.9	1.5	1	N47.70171	E15.73974	1730	AUT
	U3000	subsp. *dioica*	CP	0.573	4	0.9	1.6	1	N47.70171	E15.73974	1730	AUT
	U3001	subsp. *dioica*	CP	0.583	4	0.8	1.6	1	N47.70171	E15.73974	1730	AUT
	U3002	subsp. *dioica*	CP	0.600	4	0.7	1.8	1	N47.70171	E15.73974	1730	AUT
	U3003	subsp. *dioica*	CP	0.578	4	0.8	1.8	1	N47.70171	E15.73974	1730	AUT
	U3004	subsp. *dioica*	CP	0.575	4	0.8	1.3	1	N47.70171	E15.73974	1730	AUT
	U3005	subsp. *dioica*	CP	0.575	4	0.9	1.3	1	N47.70171	E15.73974	1730	AUT
	U3006	subsp. *dioica*	CP	0.572	4	1.0	1.8	1	N47.70171	E15.73974	1730	AUT
	U3007	subsp. *dioica*	CP	0.573	4	0.7	1.5	1	N47.70171	E15.73974	1730	AUT
	U3008	subsp. *dioica*	CP	0.585	4	0.9	1.6	1	N47.70171	E15.73974	1730	AUT
UP0792	U3023	*U. kioviensis*	TU	0.343	2	0.6	1.8	1	N48.94404	E16.59074	160	CZE
	U3024	*U. kioviensis*	TU	0.343	2	1.3	2.2	1	N48.94404	E16.59074	160	CZE
	U3025	*U. kioviensis*	TU	0.320	2	1.1	2.1	1	N48.94404	E16.59074	160	CZE
	U3026	*U. kioviensis*	TU	0.321	2	1.1	2.1	1	N48.94404	E16.59074	160	CZE
	U3027	*U. kioviensis*	TU	0.339	2	0.6	1.8	1	N48.94404	E16.59074	160	CZE
	U3028	*U. kioviensis*	TU	0.342	2	0.8	1.7	1	N48.94404	E16.59074	160	CZE
	U3029	*U. kioviensis*	TU	0.337	2	1.0	2.3	1	N48.94404	E16.59074	160	CZE
	U3030	*U. kioviensis*	TU	0.339	2	0.7	1.7	1	N48.94404	E16.59074	160	CZE
	U3031	*U. kioviensis*	TU	0.337	2	0.8	2.0	1	N48.94404	E16.59074	160	CZE
	U3032	*U. kioviensis*	TU	0.322	2	1.0	1.7	1	N48.94404	E16.59074	160	CZE
	U3033	*U. kioviensis*	TU	0.343	2	0.7	2.0	1	N48.94404	E16.59074	160	CZE
	U3034	*U. kioviensis*	TU	0.336	2	0.9	2.0	1	N48.94404	E16.59074	160	CZE

ID number of population	ID number of analysis	Taxon	Collector	Relative fluorescence intensi	DNA-ploidy level	CV of standard	CV of sample	N. of individuals in analysis	Latitude (WGS-84)	Longitude (WGS-84)	Altitude (m a.s.l.)	Country (ISO 3166-1)
	U3724	subsp. *dioica*	TU	0.567	4	1.0	1.3	1	N48.94404	E16.59074	160	CZE
	U3725	subsp. *subinermis*	TU	0.322	2	0.9	1.7	1	N48.94404	E16.59074	160	CZE
	U3726	subsp. *subinermis*	TU	0.302	2	0.8	2.2	1	N48.94404	E16.59074	160	CZE
	U3727	subsp. *subinermis*	TU	0.299	2	0.7	2.2	1	N48.94404	E16.59074	160	CZE
	U3728	subsp. *subinermis*	TU	0.293	2	0.7	1.9	1	N48.94404	E16.59074	160	CZE
	U3729	subsp. *subinermis*	TU	0.302	2	0.5	2.3	1	N48.94404	E16.59074	160	CZE
	U3730	subsp. *dioica*	TU	0.557	4	0.6	1.2	1	N48.94404	E16.59074	160	CZE
	U3731	subsp. *subinermis*	TU	0.300	2	0.6	1.7	1	N48.94404	E16.59074	160	CZE
	U3732	subsp. *dioica*	TU	0.566	4	0.7	1.2	1	N48.94404	E16.59074	160	CZE
	U3733	subsp. *dioica*	TU	0.568	4	1.0	1.3	1	N48.94404	E16.59074	160	CZE
	U3734	subsp. *dioica*	TU	0.565	4	1.0	1.6	1	N48.94404	E16.59074	160	CZE
	U3735	subsp. *dioica*	TU	0.574	4	1.0	1.3	1	N48.94404	E16.59074	160	CZE
	U3736	subsp. *subinermis*	TU	0.324	2	0.6	2.0	1	N48.94404	E16.59074	160	CZE
	U3737	subsp. *subinermis*	TU	0.327	2	0.7	2.2	1	N48.94404	E16.59074	160	CZE
	U3738	subsp. *dioica*	TU	0.573	4	1.0	1.0	1	N48.94404	E16.59074	160	CZE
	U3739	subsp. *dioica*	TU	0.576	4	0.7	1.0	1	N48.94404	E16.59074	160	CZE
	U3740	subsp. *subinermis*	TU	0.298	2	0.7	2.0	1	N48.94404	E16.59074	160	CZE
UP0793	U3047	subsp. *dioica*	LR	0.560	4	1.4	1.7	5	N49.68920	E15.10983	432	CZE
	U3078	subsp. *dioica*	LR	0.560	4	1.2	1.2	1	N49.68920	E15.10983	432	CZE
	U3079	subsp. *dioica*	LR	0.564	4	0.9	1.0	1	N49.68920	E15.10983	432	CZE
	U3080	subsp. *dioica*	LR	0.544	4	0.5	0.9	1	N49.68920	E15.10983	432	CZE
	U3081	subsp. *dioica*	LR	0.569	4	0.9	1.6	1	N49.68920	E15.10983	432	CZE
UP0794	U3048	subsp. *dioica*	LR	0.560	4	1.7	1.9	3	N49.51457	E15.41653	616	CZE

ID number of population	ID number of analysis	Taxon	Collector	Relative fluorescence intensi	DNA-ploidy level	CV of standard	CV of sample	N. of individuals in analysis	Latitude (WGS-84)	Longitude (WGS-84)	Altitude (m a.s.l.)	Country (ISO 3166-1)
UP0795	U3049	subsp. *dioica*	LR	0.552	4	2.2	2.3	3	N49.14113	E16.20512	298	CZE
UP0796	U3050	subsp. *dioica*	RB	0.562	4	2.7	2.9	5	N48.82570	E16.09330	268	CZE
UP0797	U3051	subsp. *dioica*	RB	0.559	4	1.4	2.3	5	N48.84175	E16.12119	213	CZE
UP0798	U3052	subsp. *dioica*	RB	0.562	4	2.0	2.2	5	N49.13652	E16.97326	253	CZE
UP0799	U3053	subsp. *dioica*	RB	0.564	4	1.6	2.5	5	N49.15578	E16.92359	276	CZE
UP0800	U3054	subsp. *dioica*	RB	0.554	4	1.6	2.0	5	N49.19459	E16.92602	292	CZE
UP0801	U3055	subsp. *dioica*	RB	0.569	4	1.7	1.8	5	N49.17022	E16.88927	356	CZE
UP0802	U3056	subsp. *dioica*	RB	0.568	4	1.8	2.3	5	N49.71465	E16.25503	570	CZE
UP0803	U3057	subsp. *dioica*	RB	0.560	4	1.5	1.8	5	N49.04737	E16.32021	307	CZE
UP0804	U3058	subsp. *dioica*	RB	0.571	4	1.7	1.7	3	N49.17138	E16.40997	298	CZE
	U3141	subsp. *dioica*	RB	0.567	4	1.3	1.2	1	N49.17138	E16.40997	298	CZE
	U3142	subsp. *dioica*	RB	0.564	4	0.9	1.3	1	N49.17138	E16.40997	298	CZE
	U3143	subsp. *dioica*	RB	0.562	4	0.9	1.2	1	N49.17138	E16.40997	298	CZE
	U3144	subsp. *dioica*	RB	0.567	4	0.9	1.3	1	N49.17138	E16.40997	298	CZE
UP0805	U3059	subsp. *dioica*	RB	0.562	4	0.9	1.2	4	N49.57450	E17.27853	211	CZE
UP0806	U3060	subsp. *dioica*	RB	0.561	4	1.7	1.6	4	N49.82717	E16.10019	475	CZE
UP0807	U3061	subsp. *dioica*	PK	0.576	4	1.7	1.6	5	N49.94096	E14.20423	369	CZE
	U3062	subsp. *dioica*	PK	0.571	4	1.1	1.9	5	N49.94096	E14.20423	369	CZE
	U3063	subsp. *dioica*	PK	0.565	4	1.0	1.5	5	N49.94096	E14.20423	369	CZE
	U3064	subsp. *dioica*	PK	0.567	4	0.9	1.4	5	N49.94096	E14.20423	369	CZE
UP0808	U3065	subsp. *dioica*	PK	0.571	4	1.3	1.6	5	N50.01982	E15.30019	204	CZE
	U3066	subsp. *dioica*	PK	0.564	4	1.2	1.6	5	N50.01982	E15.30019	204	CZE
	U3067	subsp. *dioica*	PK	0.563	4	1.3	1.5	5	N50.01982	E15.30019	204	CZE

ID number of population	ID number of analysis	Taxon	Collector	Relative fluorescence intensi	DNA-ploidy level	CV of standard	CV of sample	N. of individuals in analysis	Latitude (WGS-84)	Longitude (WGS-84)	Altitude (m a.s.l.)	Country (ISO 3166-1)
UP0809	U3068	subsp. *dioica*	PK	0.573	4	1.3	1.6	7	N50.14887	E15.05279	186	CZE
	U3069	subsp. *dioica*	PK	0.570	4	1.9	1.7	7	N50.14887	E15.05279	186	CZE
	U3070	subsp. *dioica*	PK	0.574	4	1.0	1.7	7	N50.14887	E15.05279	186	CZE
	U3070	subsp. *dioica*	PK	0.570	4	1.2	2.0	7	N50.14887	E15.05279	186	CZE
	U3071	subsp. *dioica*	PK	0.568	4	1.2	1.4	7	N50.14887	E15.05279	186	CZE
UP0810	U3072	subsp. *dioica*	PK	0.569	4	1.0	1.7	6	N50.11408	E15.17422	197	CZE
	U3073	subsp. *dioica*	PK	0.567	4	1.1	1.7	5	N50.11408	E15.17422	197	CZE
	U3074	subsp. *dioica*	PK	0.569	4	1.3	1.7	5	N50.11408	E15.17422	197	CZE
UP0811	U3075	subsp. *dioica*	PK	0.560	4	1.3	1.6	6	N49.42504	E14.60711	459	CZE
	U3076	subsp. *dioica*	PK	0.566	4	1.3	1.5	6	N49.42504	E14.60711	459	CZE
	U3077	subsp. *dioica*	PK	0.566	4	1.5	1.5	6	N49.42504	E14.60711	459	CZE
UP0812	U3082	subsp. *dioica*	PK	0.561	4	0.8	1.1	1	N49.25892	E13.87251	404	CZE
	U3083	subsp. *dioica*	PK	0.563	4	0.6	1.2	1	N49.25892	E13.87251	404	CZE
	U3084	subsp. *dioica*	PK	0.570	4	0.9	0.9	1	N49.25892	E13.87251	404	CZE
	U3085	subsp. *dioica*	PK	0.566	4	0.7	1.2	1	N49.25892	E13.87251	404	CZE
	U3086	subsp. *dioica*	PK	0.565	4	0.8	1.0	1	N49.25892	E13.87251	404	CZE
	U3087	subsp. *dioica*	PK	0.572	4	0.8	1.3	1	N49.25892	E13.87251	404	CZE
	U3088	subsp. *dioica*	PK	0.572	4	0.7	0.9	1	N49.25892	E13.87251	404	CZE
	U3089	subsp. *dioica*	PK	0.558	4	0.9	1.0	1	N49.25892	E13.87251	404	CZE
	U3090	subsp. *dioica*	PK	0.575	4	0.8	1.0	1	N49.25892	E13.87251	404	CZE
	U3091	subsp. *dioica*	PK	0.562	4	0.8	0.9	1	N49.25892	E13.87251	404	CZE
	U3092	subsp. *dioica*	PK	0.575	4	0.8	1.3	1	N49.25892	E13.87251	404	CZE
	U3093	subsp. *dioica*	PK	0.557	4	0.9	1.4	1	N49.25892	E13.87251	404	CZE

ID number of population	ID number of analysis	Taxon	Collector	Relative fluorescence intensi	DNA-ploidy level	CV of standard	CV of sample	N. of individuals in analysis	Latitude (WGS-84)	Longitude (WGS-84)	Altitude (m a.s.l.)	Country (ISO 3166-1)
	U3094	subsp. *dioica*	PK	0.581	4	0.9	1.0	1	N49.25892	E13.87251	404	CZE
	U3095	subsp. *dioica*	PK	0.562	4	0.9	1.3	1	N49.25892	E13.87251	404	CZE
	U3096	subsp. *dioica*	PK	0.566	4	0.8	1.3	1	N49.25892	E13.87251	404	CZE
UP0813	U3097	subsp. *dioica*	PK	0.567	4	1.0	1.9	6	N49.26610	E13.83792	407	CZE
	U3098	subsp. *dioica*	PK	0.563	4	0.7	1.6	6	N49.26610	E13.83792	407	CZE
UP0814	U3099	subsp. *dioica*	LR	0.569	4	1.0	1.3	5	N48.65250	E14.23163	906	CZE
UP0815	U3100	subsp. *dioica*	LR	0.578	4	0.7	1.4	4	N48.79795	E14.30685	498	CZE
UP0816	U3101	subsp. *dioica*	PK	0.563	4	0.9	1.1	6	N49.27280	E13.61885	446	CZE
UP0817	U3103	subsp. *dioica*	LR	0.579	4	1.4	1.6	5	N50.19858	E15.96122	237	CZE
UP0818	U3104	subsp. *dioica*	LR	0.571	4	1.3	1.9	5	N50.01925	E16.33586	446	CZE
UP0819	U3105	subsp. *dioica*	LR	0.567	4	1.2	1.7	5	N49.99964	E16.25142	312	CZE
UP0820	U3106	subsp. *dioica*	RB	0.573	4	1.0	1.3	2	N49.45531	E15.59381	523	CZE
UP0821	U3107	subsp. *dioica*	LR	0.566	4	1.0	1.5	5	N50.08644	E16.11394	258	CZE
UP0822	U3108	subsp. *dioica*	LR	0.570	4	1.7	1.7	5	N50.08944	E16.11253	259	CZE
UP0823	U3109	subsp. *dioica*	LR	0.577	4	1.5	1.7	5	N50.11583	E15.80078	222	CZE
UP0824	U3110	subsp. *dioica*	PV, TU	0.569	4	1.2	1.2	1	N52.56075	E14.67413	13	POL
	U3111	*U. kioviensis*	PV, TU	0.342	2	1.1	1.8	1	N52.56075	E14.67413	13	POL
UP0825	U3112	subsp. *dioica*	JB	0.565	4	1.3	1.6	1	N48.90424	E14.29547	509	CZE
	U3113	subsp. *dioica*	JB	0.633	4	0.6	1.4	1	N48.90424	E14.29547	509	CZE
	U3114	subsp. *dioica*	JB	0.555	4	0.9	1.5	1	N48.90424	E14.29547	509	CZE
	U3115	subsp. *dioica*	JB	0.573	4	1.0	1.6	1	N48.90424	E14.29547	509	CZE
	U3116	subsp. *dioica*	JB	0.572	4	1.0	1.7	1	N48.90424	E14.29547	509	CZE
UP0826	U3117	subsp. *dioica*	FK	0.566	4	0.8	1.2	5	N44.17975	E19.87119	478	SRB

ID number of population	ID number of analysis	Taxon	Collector	Relative fluorescence intensi	DNA-ploidy level	CV of standard	CV of sample	N. of individuals in analysis	Latitude (WGS-84)	Longitude (WGS-84)	Altitude (m a.s.l.)	Country (ISO 3166-1)
UP0827	U3118	subsp. *dioica*	FK	0.553	4	0.8	1.2	8	N45.41035	E22.77623	714	ROU
UP0828	U3119	subsp. *dioica*	LR	0.567	4	1.0	1.5	5	N50.19314	E15.97994	239	CZE
	U3135	subsp. *dioica*	LR	0.570	4	1.2	1.8	5	N50.19314	E15.97994	239	CZE
UP0829	U3120	subsp. *dioica*	JA	0.569	4	1.2	1.7	5	N50.23862	E12.69942	485	CZE
	U3677	subsp. *dioica*	JA	0.568	4	1.6	1.8	5	N50.23862	E12.69942	485	CZE
UP0830	U3121	subsp. *dioica*	RB	0.563	4	1.2	1.6	5	N50.56285	E13.97431	541	CZE
UP0831	U3122	subsp. *dioica*	RB	0.576	4	1.0	1.8	4	N48.86118	E16.64640	343	CZE
UP0832	U3123	subsp. *dioica*	RB	0.570	4	1.1	1.8	5	N48.85559	E16.64462	340	CZE
UP0833	U3124	subsp. *dioica*	RB	0.569	4	1.0	1.7	5	N48.82614	E16.64158	353	CZE
UP0834	U3125	subsp. *dioica*	LR	0.584	4	0.9	1.2	5	N46.91975	E13.57945	906	AUT
UP0835	U3126	subsp. *dioica*	LR	0.577	4	0.9	1.2	5	N46.43998	E14.02674	581	SVN
UP0836	U3127	subsp. *dioica*	LR	0.580	4	0.7	1.1	5	N45.08062	E13.64661	29	HRV
UP0837	U3128	subsp. *dioica*	LR	0.581	4	1.3	1.0	5	N46.57303	E13.83565	531	AUT
UP0838	U3129	subsp. *dioica*	RB	0.566	4	0.7	1.3	2	N50.15679	E19.93557	349	POL
UP0839	U3130	subsp. *dioica*	RB	0.570	4	0.7	1.0	2	N50.06128	E19.94756	211	POL
UP0840	U3131	subsp. *dioica*	RB	0.577	4	0.8	1.1	4	N51.10492	E17.03820	122	POL
UP0841	U3132	subsp. *dioica*	RB	0.579	4	0.7	1.2	5	N50.34983	E14.49229	171	CZE
UP0842	U3133	subsp. *dioica*	RB	0.567	4	0.5	1.2	6	N50.34702	E14.49882	174	CZE
UP0843	U3134	subsp. *dioica*	RB	0.570	4	0.8	0.9	5	N50.33936	E14.53998	189	CZE
UP0844	U3136	subsp. *dioica*	RB	0.558	4	0.6	1.1	5	N50.52807	E15.09166	246	CZE
UP0845	U3137	subsp. *dioica*	RB	0.563	4	0.9	1.1	6	N50.59446	E14.88859	388	CZE
UP0846	U3138	subsp. *dioica*	RB	0.573	4	1.4	1.4	5	N49.01844	E14.77760	432	CZE
UP0847	U3139	subsp. *dioica*	RB	0.561	4	1.0	1.3	5	N50.33404	E13.08063	440	CZE

ID number of population	ID number of analysis	Taxon	Collector	Relative fluorescence intensi	DNA-ploidy level	CV of standard	CV of sample	N. of individuals in analysis	Latitude (WGS-84)	Longitude (WGS-84)	Altitude (m a.s.l.)	Country (ISO 3166-1)
UP0848	U3140	subsp. *dioica*	RB	0.558	4	1.8	2.3	5	N49.32543	E15.32009	605	CZE
UP0849	U3145	subsp. *dioica*	RB	0.566	4	0.7	1.3	4	N48.81258	E15.98163	258	CZE
UP0850	U3146	subsp. *dioica*	RB	0.568	4	1.0	1.4	5	N49.87268	E14.89729	290	CZE
UP0851	U3147	subsp. *dioica*	RB	0.570	4	0.9	1.4	1	N48.61134	E20.87384	344	SVK
	U3148	subsp. *dioica*	RB	0.563	4	1.0	1.3	4	N48.61134	E20.87384	344	SVK
UP0852	U3149	subsp. *dioica*	RB	0.573	4	0.9	1.4	3	N48.48742	E20.55065	227	HUN
UP0853	U3150	subsp. *dioica*	RB	0.572	4	0.8	1.3	3	N50.11890	E25.73972	365	UKR
UP0854	U3151	subsp. *dioica*	RB	0.557	4	0.9	1.5	2	N49.22437	E24.70292	313	UKR
UP0855	U3152	subsp. *dioica*	RB	0.572	4	1.0	2.0	3	N48.16205	E24.55347	1142	UKR
UP0856	U3153	subsp. *dioica*	RB	0.564	4	1.3	1.6	5	N48.63821	E26.78208	129	UKR
UP0857	U3154	subsp. *dioica*	RB	0.564	4	1.2	1.3	2	N48.13392	E27.16782	132	MDA
UP0858	U3155	subsp. *dioica*	RB	0.574	4	1.4	2.1	1	N47.80081	E27.28225	140	MDA
	U3156	subsp. *dioica*	RB	0.559	4	0.8	1.3	1	N47.80081	E27.28225	140	MDA
	U3157	subsp. *dioica*	RB	0.583	4	0.9	1.9	1	N47.80081	E27.28225	140	MDA
UP0859	U3158	subsp. *dioica*	RB	0.573	4	0.8	1.4	4	N47.61506	E27.40441	53	MDA
UP0860	U3159	subsp. *dioica*	RB	0.573	4	1.1	1.5	5	N47.62170	E27.39394	55	MDA
UP0861	U3160	subsp. *dioica*	RB	0.566	4	1.0	1.4	2	N47.35573	E27.37043	42	ROU
UP0862	U3161	subsp. *dioica*	JR	0.560	4	0.8	0.9	1	N49.13645	E16.23324	269	CZE
UP0863	U3162	subsp. *dioica*	LR	0.584	4	1.7	1.5	5	N50.15028	E16.06439	248	CZE
	U3175	subsp. *dioica*	LR	0.575	4	1.2	1.6	5	N50.15028	E16.06439	248	CZE
UP0864	U3163	subsp. *dioica*	RB	0.575	4	1.0	1.4	2	N46.68166	E25.39290	809	ROU
UP0865	U3164	subsp. *dioica*	RB	0.564	4	1.6	1.7	5	N46.38212	E23.58089	481	ROU
UP0866	U3165	subsp. *dioica*	TU	0.571	4	0.7	1.1	1	N48.64958	E20.44265	528	SVK

ID number of population	ID number of analysis	Taxon	Collector	Relative fluorescence intensi	DNA-ploidy level	CV of standard	CV of sample	N. of individuals in analysis	Latitude (WGS-84)	Longitude (WGS-84)	Altitude (m a.s.l.)	Country (ISO 3166-1)
UP0867	U3166	subsp. *dioica*	TU	0.575	4	0.9	1.4	3	N48.87204	E16.65778	409	CZE
UP0868	U3167	subsp. *dioica*	TU	0.558	4	1.2	1.1	1	N49.17553	E20.26463	1065	SVK
UP0869	U3168	subsp. *dioica*	TU	0.571	4	1.0	1.5	5	N49.84083	E14.81361	461	CZE
UP0870	U3169	subsp. *dioica* triploid	GF	0.438	3	0.9	1.3	1	N49.72099	E15.71472	514	CZE
	U3170	subsp. *dioica*	GF	0.571	4	0.8	1.6	1	N49.72099	E15.71472	514	CZE
	U3171	subsp. *dioica*	GF	0.571	4	0.8	1.1	1	N49.72099	E15.71472	514	CZE
	U3172	subsp. *dioica*	GF	0.566	4	0.8	1.2	1	N49.72099	E15.71472	514	CZE
	U3173	subsp. *dioica* triploid	GF	0.445	3	0.9	1.5	1	N49.72099	E15.71472	514	CZE
	U3174	subsp. *dioica* triploid	GF	0.459	3	0.8	1.4	1	N49.72099	E15.71472	514	CZE
UP0871	U3176	subsp. *dioica*	TU	0.565	4	0.7	1.5	4	N50.80572	E14.54061	499	CZE
UP0872	U3177	subsp. *dioica*	TU	0.559	4	1.2	1.4	5	N50.28585	E14.34354	167	CZE
UP0873	U3178	subsp. *dioica*	TU	0.572	4	1.2	1.3	5	N50.29267	E14.34354	171	CZE
UP0874	U3179	subsp. *dioica*	TU	0.568	4	1.3	1.4	5	N50.29935	E14.33206	166	CZE
UP0875	U3180	subsp. *dioica*	TU	0.567	4	1.5	1.5	5	N50.31619	E14.43294	160	CZE
	U3181	subsp. *dioica*	TU	0.572	4	1.7	1.5	5	N50.31619	E14.43294	160	CZE
UP0876	U3182	subsp. *dioica*	TU	0.549	4	1.4	2.4	5	N50.31776	E14.44203	161	CZE
UP0877	U3183	subsp. *dioica*	TU	0.560	4	1.4	1.7	5	N50.32178	E14.43966	163	CZE
	U3184	subsp. *dioica*	TU	0.563	4	1.2	1.7	5	N50.32178	E14.43966	163	CZE
UP0878	U3186	subsp. *dioica*	LR	0.568	4	1.0	1.5	5	N50.10339	E16.28047	293	CZE
UP0879	U3187	subsp. *dioica*	RB	0.543	4	1.7	1.7	3	N48.99055	E19.28263	574	SVK
UP0880	U3188	subsp. *dioica*	LR	0.565	4	1.0	1.4	5	N50.09067	E16.36800	386	CZE
	U3194	subsp. *dioica*	LR	0.560	4	1.2	1.4	5	N50.09067	E16.36800	386	CZE
	U3195	subsp. *dioica*	LR	0.570	4	1.4	1.6	5	N50.09067	E16.36800	386	CZE

ID number of population	ID number of analysis	Taxon	Collector	Relative fluorescence intensi	DNA-ploidy level	CV of standard	CV of sample	N. of individuals in analysis	Latitude (WGS-84)	Longitude (WGS-84)	Altitude (m a.s.l.)	Country (ISO 3166-1)
UP0881	U3189	subsp. *dioica*	RB	0.564	4	1.1	2.1	2	N49.04667	E19.28872	734	SVK
UP0882	U3190	subsp. *dioica*	RB	0.550	4	1.2	1.0	1	N48.98867	E19.30307	806	SVK
	U3191	subsp. *dioica*	RB	0.557	4	1.2	1.5	1	N48.98867	E19.30307	806	SVK
	U3192	subsp. *dioica*	RB	0.560	4	1.3	1.9	1	N48.98867	E19.30307	806	SVK
	U3193	subsp. *dioica*	RB	0.559	4	1.3	1.5	1	N48.98867	E19.30307	806	SVK
	U3205	subsp. *dioica*	SP	0.569	4	1.5	1.8	1	N48.98867	E19.30307	806	SVK
UP0883	U3196	subsp. *dioica*	TU	0.551	4	1.1	1.7	1	N48.37417	E16.23819	170	AUT
UP0884	U3198	subsp. *dioica*	TU	0.548	4	1.4	1.8	1	N47.49108	E16.27989	432	AUT
UP0885	U3200	subsp. *dioica*	TF	0.563	4	1.7	1.4	1	N49.07515	E12.10112	331	DEU
UP0886	U3201	subsp. *dioica*	AR	0.611	4	0.8	1.5	1	N51.12881	E17.05693	116	POL
UP0887	U3202	subsp. *dioica*	FK	0.577	4	1.0	1.4	1	N47.33497	E15.37219	494	AUT
UP0888	U3203	subsp. *dioica* aneuploid	FK	0.498	–	1.1	1.7	1	N47.14472	E15.87933	323	AUT
UP0889	U3204	subsp. *dioica*	FK	0.556	4	1.1	1.6	1	N49.95108	E13.75200	274	CZE
UP0890	U3206	subsp. *dioica*	RB	0.569	4	1.3	1.4	5	N47.77383	E14.17643	504	AUT
UP0891	U3207	subsp. *subinermis*	JR	0.294	2	1.2	2.3	5	N50.91993	E14.13936	120	DEU
	U3208	subsp. *subinermis*	JR	0.294	2	1.7	2.2	5	N50.91993	E14.13936	120	DEU
	U3209	subsp. *subinermis*	JR	0.296	2	1.2	1.8	5	N50.91993	E14.13936	120	DEU
UP0892	U3210	subsp. *subinermis*	FK	0.294	2	0.8	1.6	1	N46.19805	E18.85195	96	HRV
	U3211	subsp. *subinermis*	FK	0.300	2	0.8	1.5	1	N46.19805	E18.85195	96	HRV
	U3212	subsp. *subinermis*	FK	0.303	2	0.8	1.6	1	N46.19805	E18.85195	96	HRV
	U3213	subsp. *subinermis*	FK	0.299	2	0.7	1.5	1	N46.19805	E18.85195	96	HRV
	U3214	subsp. *subinermis*	FK	0.302	2	0.7	1.7	1	N46.19805	E18.85195	96	HRV
	U3215	subsp. *subinermis*	FK	0.300	2	0.8	1.5	1	N46.19805	E18.85195	96	HRV

ID number of population	ID number of analysis	Taxon	Collector	Relative fluorescence intensi	DNA-ploidy level	CV of standard	CV of sample	N. of individuals in analysis	Latitude (WGS-84)	Longitude (WGS-84)	Altitude (m a.s.l.)	Country (ISO 3166-1)
	U3216	subsp. *subinermis*	FK	0.298	2	0.7	1.5	1	N46.19805	E18.85195	96	HRV
UP0893	U3217	subsp. *dioica*	FK	0.561	4	1.2	1.7	5	N49.12150	E19.86643	854	SVK
UP0894	U3218	subsp. *dioica*	ML	0.560	4	0.7	1.3	3	N50.07736	E17.28493	900	CZE
UP0895	U3219	subsp. *dioica*	RB	0.563	4	1.1	1.5	6	N48.95079	E15.98788	338	CZE
UP0896	U3220	subsp. *dioica*	TU	0.563	4	1.2	1.3	5	N50.41453	E12.89964	960	CZE
UP0897	U3221	subsp. *dioica*	TU	0.563	4	1.2	1.7	5	N49.78692	E14.07747	621	CZE
UP0898	U3222	subsp. *dioica*	FK	0.569	4	1.0	1.4	5	N48.75449	E16.89148	157	CZE
UP0899	U3223	subsp. *dioica*	FK	0.579	4	1.4	1.4	5	N48.74169	E16.89697	157	CZE
UP0900	U3224	subsp. *dioica*	TU	0.579	4	0.9	1.4	5	N50.35739	E13.73881	187	CZE
UP0901	U3225	subsp. *dioica*	RB	0.550	4	0.8	1.4	5	N49.62909	E16.35100	604	CZE
UP0902	U3226	subsp. *dioica*	TU	0.556	4	1.1	1.3	3	N50.71825	E15.37809	713	CZE
UP0903	U3227	subsp. *dioica*	TU	0.559	4	0.9	1.3	4	N50.45511	E14.25619	170	CZE
UP0904	U3228	subsp. *dioica*	RB	0.563	4	1.3	2.1	5	N50.55802	E13.97288	437	CZE
UP0905	U3229	subsp. *dioica*	FK	0.563	4	1.1	1.6	5	N50.07910	E14.50827	231	CZE
UP0906	U3230	subsp. *dioica*	LR	0.578	4	1.4	1.3	2	N49.26048	E13.94343	389	CZE
UP0907	U3231	subsp. *dioica*	LR	0.578	4	1.1	1.6	2	N49.00606	E14.44152	380	CZE
UP0908	U3232	subsp. *dioica*	LR	0.581	4	1.4	1.4	2	N49.00696	E14.43152	383	CZE
UP0909	U3233	subsp. *dioica*	LR	0.566	4	1.7	1.2	1	N49.00358	E14.42556	388	CZE
UP0910	U3234	subsp. *dioica*	LR	0.583	4	1.2	1.7	2	N48.99767	E14.43062	387	CZE
UP0911	U3235	subsp. *dioica*	LR	0.562	4	1.6	1.7	2	N49.00071	E14.44023	383	CZE
UP0912	U3236	subsp. *dioica*	LR	0.571	4	1.3	1.5	2	N49.26085	E13.86634	406	CZE
UP0913	U3237	subsp. *dioica*	LR	0.558	4	1.5	1.8	3	N49.38078	E14.14253	440	CZE
UP0914	U3238	subsp. *dioica*	LR	0.576	4	1.4	1.7	2	N49.04866	E14.37496	383	CZE

ID number of population	ID number of analysis	Taxon	Collector	Relative fluorescence intensi	DNA-ploidy level	CV of standard	CV of sample	N. of individuals in analysis	Latitude (WGS-84)	Longitude (WGS-84)	Altitude (m a.s.l.)	Country (ISO 3166-1)
UP0915	U3239	subsp. *dioica*	LR	0.571	4	1.2	1.5	2	N49.07183	E14.34664	383	CZE
UP0916	U3240	subsp. *dioica*	LR	0.573	4	1.2	1.6	1	N49.37681	E14.15171	424	CZE
UP0917	U3241	subsp. *dioica*	LR	0.568	4	1.2	1.7	2	N49.37122	E14.15395	423	CZE
UP0918	U3242	subsp. *dioica*	LR	0.564	4	1.7	1.4	2	N49.37150	E14.14549	367	CZE
UP0919	U3243	subsp. *dioica*	RB	0.582	4	1.0	1.8	5	N48.97444	E14.46445	385	CZE
UP0920	U3244	subsp. *dioica*	GF	0.561	4	1.3	1.5	1	N50.64035	E14.98829	284	CZE
	U3245	subsp. *dioica*	GF	0.559	4	1.4	1.4	1	N50.64035	E14.98829	284	CZE
	U3246	subsp. *dioica*	GF	0.549	4	1.6	1.6	1	N50.64035	E14.98829	284	CZE
	U3247	subsp. *dioica*	GF	0.557	4	1.3	1.6	1	N50.64035	E14.98829	284	CZE
	U3248	subsp. *dioica*	GF	0.568	4	1.2	1.4	1	N50.64035	E14.98829	284	CZE
UP0921	U3249	subsp. *dioica*	RB	0.570	4	1.3	1.8	4	N49.89998	E14.39756	237	CZE
UP0922	U3250	subsp. *dioica*	TR	0.570	4	0.9	1.4	5	N49.88264	E14.39628	205	CZE
UP0923	U3251	subsp. *dioica*	RB	0.587	4	1.3	1.6	3	N48.87609	E16.66214	426	CZE
UP0924	U3252	subsp. *dioica*	PV	0.548	4	0.9	1.5	5	N49.97730	E14.75192	460	CZE
UP0925	U3253	subsp. *dioica*	ML	0.569	4	1.4	1.6	5	N49.46213	E17.47643	218	CZE
UP0926	U3254	subsp. *dioica*	LR	0.563	4	0.8	1.2	5	N49.34676	E14.51356	384	CZE
UP0927	U3255	subsp. *dioica*	RB	0.578	4	1.1	1.3	5	N48.88580	E16.64789	190	CZE
UP0928	U3256	subsp. *dioica*	VC	0.557	4	1.2	1.4	1	N49.70330	E14.03969	550	CZE
UP0929	U3257	subsp. *dioica*	MDU	0.570	4	0.8	1.3	5	N50.26531	E16.05317	269	CZE
	U3258	subsp. *dioica*	MDU	0.572	4	0.8	1.0	5	N50.26531	E16.05317	269	CZE
	U3259	subsp. *dioica*	MDU	0.573	4	0.6	1.3	5	N50.26531	E16.05317	269	CZE
	U3260	subsp. *dioica*	MDU	0.565	4	1.0	1.3	6	N50.26531	E16.05317	269	CZE
	U3261	subsp. *dioica*	MDU	0.576	4	0.7	2.9	6	N50.26531	E16.05317	269	CZE

ID number of population	ID number of analysis	Taxon	Collector	Relative fluorescence intensi	DNA-ploidy level	CV of standard	CV of sample	N. of individuals in analysis	Latitude (WGS-84)	Longitude (WGS-84)	Altitude (m a.s.l.)	Country (ISO 3166-1)
UP0930	U3262	subsp. *dioica*	TF	0.565	4	1.4	1.4	1	N55.95204	W3.16316	40	GBR
UP0931	U3263	subsp. *dioica*	TF	0.573	4	1.0	1.7	5	N48.85816	E17.67861	947	SVK
UP0932	U3264	subsp. *dioica*	TF	0.576	4	1.8	1.2	5	N48.82013	E21.96971	185	SVK
UP0933	U3265	subsp. *dioica*	TF	0.572	4	1.2	1.4	5	N48.57732	E18.32049	216	SVK
UP0934	U3266	subsp. *dioica*	TF	0.571	4	1.0	1.4	5	N49.03207	E21.96094	191	SVK
UP0935	U3267	subsp. *dioica*	TF	0.576	4	1.2	1.9	5	N48.36513	E18.89477	441	SVK
UP0936	U3268	subsp. *dioica*	TF	0.567	4	1.4	1.7	5	N48.89003	E21.93593	236	SVK
UP0937	U3269	subsp. *dioica*	RB	0.550	4	1.9	3.5	5	N48.81071	E16.00906	289	CZE
UP0938	U3270	subsp. *dioica*	FK	0.569	4	1.1	1.6	5	N46.78772	E17.19184	112	HUN
UP0939	U3271	subsp. *subinermis*	RB	0.301	2	1.1	2.5	1	N48.88786	E17.07357	174	CZE
	U3272	subsp. *subinermis*	RB	0.299	2	1.2	2.3	1	N48.88786	E17.07357	174	CZE
	U3273	subsp. *subinermis*	RB	0.299	2	1.7	2.3	1	N48.88786	E17.07357	174	CZE
UP0940	U3274	subsp. *dioica*	TU	0.567	4	0.9	1.6	5	N48.60150	E16.93653	176	AUT
UP0941	U3275	subsp. *subinermis*	JR	0.292	2	1.1	2.2	1	N50.66874	E14.15891	136	CZE
	U3276	subsp. *subinermis*	JR	0.293	2	1.4	2.4	1	N50.66874	E14.15891	136	CZE
	U3277	subsp. *subinermis*	JR	0.294	2	1.0	2.4	1	N50.66874	E14.15891	136	CZE
	U3278	subsp. *subinermis*	JR	0.291	2	1.1	2.2	1	N50.66874	E14.15891	136	CZE
	U3279	subsp. *subinermis*	JR	0.293	2	1.3	2.5	1	N50.66874	E14.15891	136	CZE
	U3280	subsp. *subinermis*	JR	0.292	2	1.1	2.4	1	N50.66874	E14.15891	136	CZE
	U3281	subsp. *subinermis*	JR	0.291	2	1.3	1.2	1	N50.66874	E14.15891	136	CZE
	U3282	subsp. *dioica*	JR	0.556	4	1.4	1.6	1	N50.66874	E14.15891	136	CZE
	U3283	subsp. *dioica*	JR	0.562	4	1.4	1.5	1	N50.66874	E14.15891	136	CZE
	U3284	subsp. *dioica*	JR	0.563	4	1.2	1.3	1	N50.66874	E14.15891	136	CZE

ID number of population	ID number of analysis	Taxon	Collector	Relative fluorescence intensi	DNA-ploidy level	CV of standard	CV of sample	N. of individuals in analysis	Latitude (WGS-84)	Longitude (WGS-84)	Altitude (m a.s.l.)	Country (ISO 3166-1)
	U3285	subsp. *subinermis*	JR	0.293	2	1.8	2.0	1	N50.66874	E14.15891	136	CZE
	U3286	subsp. *dioica*	JR	0.578	4	1.2	1.6	1	N50.66874	E14.15891	136	CZE
	U3287	subsp. *subinermis*	JR	0.289	2	1.7	2.4	1	N50.66874	E14.15891	136	CZE
	U3288	subsp. *subinermis*	JR	0.293	2	1.2	1.6	1	N50.66874	E14.15891	136	CZE
	U3289	subsp. *dioica*	JR	0.561	4	1.1	1.8	1	N50.66874	E14.15891	136	CZE
	U3396	subsp. *subinermis*	JR	0.288	2	1.4	2.3	5	N50.66874	E14.15891	136	CZE
	U3397	subsp. *subinermis*	JR	0.291	2	1.3	2.6	5	N50.66874	E14.15891	136	CZE
	U3398	subsp. *subinermis*	JR	0.290	2	1.1	1.9	5	N50.66874	E14.15891	136	CZE
UP0942	U3290	subsp. *dioica*	TF	0.597	4	1.4	2.2	5	N48.47498	E17.86353	228	SVK
UP0943	U3291	subsp. *subinermis*	TU	0.298	2	0.9	1.6	5	N48.34689	E16.88764	157	AUT
	U3292	subsp. *subinermis*	TU	0.303	2	1.4	1.8	5	N48.34689	E16.88764	157	AUT
UP0944	U3293	subsp. *dioica*	MDU	0.559	4	0.7	1.4	8	N50.36028	E15.62312	294	CZE
	U3294	subsp. *dioica*	MDU	0.567	4	0.6	1.6	8	N50.36028	E15.62312	294	CZE
	U3295	subsp. *dioica*	MDU	0.570	4	0.7	1.0	8	N50.36028	E15.62312	294	CZE
UP0945	U3296	subsp. *dioica*	TU	0.557	4	1.3	1.6	2	N49.37670	E17.72349	425	CZE
	U3297	subsp. *dioica*	TU	0.558	4	1.1	1.8	3	N49.37670	E17.72349	440	CZE
UP0946	U3298	subsp. *dioica*	LR	0.568	4	1.8	2.0	5	N50.10058	E15.66736	223	CZE
UP0947	U3299	subsp. *dioica*	TF	0.575	4	1.4	1.7	5	N49.15480	E20.08446	1535	SVK
UP0948	U3303	subsp. *dioica*	JP	0.582	4	0.8	1.1	1	N36.67442	E54.46397	1325	IRN
UP0949	U3307	subsp. *dioica*	JB	0.563	4	0.9	1.3	3	N49.65922	E17.36294	378	CZE
UP0950	U3308	subsp. *dioica*	JB	0.572	4	0.8	0.9	3	N49.64206	E17.36981	429	CZE
UP0951	U3309	subsp. *dioica*	JB	0.566	4	0.7	1.3	4	N49.69091	E17.34414	396	CZE
UP0952	U3310	subsp. *dioica*	JB	0.566	4	0.9	1.2	5	N50.06786	E14.41941	198	CZE

ID number of population	ID number of analysis	Taxon	Collector	Relative fluorescence intensi	DNA-ploidy level	CV of standard	CV of sample	N. of individuals in analysis	Latitude (WGS-84)	Longitude (WGS-84)	Altitude (m a.s.l.)	Country (ISO 3166-1)
UP0953	U3311	subsp. *dioica*	JB	0.566	4	1.4	1.3	5	N50.04413	E14.35345	269	CZE
UP0954	U3312	subsp. *dioica*	JR	0.559	4	0.9	0.9	4	N49.10071	E16.21351	254	CZE
UP0955	U3313	subsp. *dioica*	VR	0.564	4	2.0	2.8	1	N50.23044	E14.75086	176	CZE
UP0956	U3314	subsp. *dioica*	FK	0.559	4	1.1	1.3	5	N49.05976	E20.93043	656	SVK
UP0957	U3315	subsp. *dioica*	LR	0.574	4	0.8	0.9	5	N48.72969	E14.48100	559	CZE
UP0958	U3316	subsp. *dioica*	LR	0.573	4	0.9	1.2	5	N49.94004	E14.18800	288	CZE
UP0959	U3317	subsp. *dioica*	MD	0.584	4	1.0	2.0	1	N48.76503	E21.24214	229	SVK
UP0960	U3318	subsp. *dioica*	PT	0.572	4	1.1	1.5	5	N50.16823	E14.90592	177	CZE
UP0961	U3319	*U. kioviensis*	FK	0.336	2	0.8	1.5	1	N48.67931	E16.94617	173	CZE
UP0962	U3320	subsp. *dioica*	RB	0.561	4	1.1	1.8	5	N50.56554	E13.97277	597	CZE
UP0963	U3321	subsp. *dioica*	TU	0.564	4	1.0	1.4	3	N50.42578	E14.46278	183	CZE
UP0964	U3322	subsp. *dioica*	TF	0.559	4	1.8	2.4	5	N48.47394	E17.80484	141	SVK
UP0965	U3323	subsp. *dioica*	RB	0.564	4	1.2	1.6	2	N50.11530	E14.40732	180	CZE
UP0966	U3324	subsp. *dioica*	VC	0.558	4	1.1	1.4	1	N49.73486	E14.09411	417	CZE
UP0967	U3325	subsp. *dioica*	FK	0.564	4	1.5	1.7	5	N50.68489	E14.83344	390	CZE
UP0968	U3326	subsp. *dioica*	TU	0.561	4	1.1	1.8	3	N50.65175	E15.35836	451	CZE
UP0969	U3327	subsp. *dioica*	TU	0.561	4	1.1	1.7	1	N50.71247	E15.42056	483	CZE
UP0970	U3328	subsp. *dioica*	VK	0.567	4	0.8	1.2	1	N50.85019	E14.65150	618	DEU
UP0971	U3329	subsp. *dioica*	VK	0.570	4	0.8	1.2	1	N50.58542	E16.11436	671	CZE
UP0972	U3330	subsp. *dioica*	VK	0.567	4	0.8	1.1	1	N50.65799	E15.59464	611	CZE
UP0973	U3331	subsp. *dioica*	VK	0.561	4	0.9	1.3	1	N50.69891	E15.59464	766	CZE
UP0974	U3332	subsp. *subinermis*	LR	0.299	2	1.0	2.3	5	N48.69703	E16.95969	154	CZE
UP0975	U3333	subsp. *dioica*	PT	0.571	4	0.8	1.6	5	N50.10632	E15.19024	201	CZE

ID number of population	ID number of analysis	Taxon	Collector	Relative fluorescence intensi	DNA-ploidy level	CV of standard	CV of sample	N. of individuals in analysis	Latitude (WGS-84)	Longitude (WGS-84)	Altitude (m a.s.l.)	Country (ISO 3166-1)
UP0976	U3334	subsp. *dioica*	LM	0.558	4	1.2	1.7	5	N49.70647	E17.09705	232	CZE
UP0977	U3335	subsp. *dioica*	ML	0.558	4	0.7	1.4	7	N49.70834	E17.09481	230	CZE
UP0978	U3336	subsp. *dioica*	LR	0.567	4	0.7	1.2	4	N53.39834	W2.97262	38	GBR
UP0979	U3337	subsp. *dioica*	LM	0.565	4	0.9	1.1	1	N49.20809	E19.04139	763	SVK
UP0980	U3338	subsp. *dioica*	LR	0.561	4	0.9	2.3	5	N49.54238	E13.31842	356	CZE
UP0981	U3339	subsp. *dioica*	LR	0.562	4	1.3	1.5	5	N49.54525	E13.31770	356	CZE
UP0982	U3340	subsp. *dioica*	LR	0.550	4	1.6	2.9	5	N49.54452	E13.31932	355	CZE
UP0983	U3341	subsp. *dioica*	LR	0.570	4	1.7	2.1	5	N49.54418	E13.31867	356	CZE
UP0984	U3342	subsp. *dioica*	LR	0.553	4	1.5	1.7	5	N49.54433	E13.31732	356	CZE
UP0985	U3343	subsp. *dioica*	LR	0.559	4	1.4	1.8	5	N49.54362	E13.31695	356	CZE
UP0986	U3344	subsp. *dioica*	LR	0.572	4	1.0	1.7	5	N49.54310	E13.31847	357	CZE
UP0987	U3345	subsp. *dioica*	LR	0.567	4	1.2	1.6	5	N49.54335	E13.31875	356	CZE
UP0988	U3346	subsp. *dioica*	LR	0.577	4	1.2	1.6	5	N49.54575	E13.31997	359	CZE
	U3347	subsp. *dioica*	LR	0.568	4	1.3	1.6	5	N49.54575	E13.31997	359	CZE
	U3348	subsp. *dioica*	LR	0.598	4	1.2	1.8	5	N49.54575	E13.31997	359	CZE
	U3349	subsp. *dioica*	LR	0.563	4	1.3	1.5	5	N49.54575	E13.31997	359	CZE
	U3350	subsp. *dioica*	LR	0.556	4	0.9	1.8	5	N49.54575	E13.31997	359	CZE
	U3351	subsp. *dioica*	LR	0.574	4	1.1	1.5	5	N49.54575	E13.31997	359	CZE
	U3352	subsp. *dioica*	LR	0.573	4	1.0	1.6	5	N49.54575	E13.31997	359	CZE
	U3353	subsp. *dioica*	LR	0.584	4	1.2	2.3	5	N49.54575	E13.31997	359	CZE
	U3354	subsp. *dioica*	LR	0.576	4	1.2	1.5	5	N49.54575	E13.31997	359	CZE
	U3355	subsp. *dioica*	LR	0.572	4	1.4	1.7	5	N49.54575	E13.31997	359	CZE
	U3356	subsp. *dioica*	LR	0.580	4	0.8	1.8	5	N49.54575	E13.31997	359	CZE

ID number of population	ID number of analysis	Taxon	Collector	Relative fluorescence intensi	DNA-ploidy level	CV of standard	CV of sample	N. of individuals in analysis	Latitude (WGS-84)	Longitude (WGS-84)	Altitude (m a.s.l.)	Country (ISO 3166-1)
	U3357	subsp. *dioica*	LR	0.580	4	1.7	1.6	5	N49.54575	E13.31997	359	CZE
	U3358	subsp. *dioica*	LR	0.575	4	1.4	1.7	5	N49.54575	E13.31997	359	CZE
	U3359	subsp. *dioica*	LR	0.574	4	1.4	1.8	5	N49.54575	E13.31997	359	CZE
UP0989	U3360	subsp. *dioica*	PK	0.576	4	1.3	1.4	5	N50.38570	E13.92320	181	CZE
	U3361	subsp. *dioica*	PK	0.573	4	1.1	1.5	5	N50.38570	E13.92320	181	CZE
	U3362	subsp. *dioica*	PK	0.580	4	1.2	1.6	5	N50.38570	E13.92320	181	CZE
	U3363	subsp. *dioica*	PK	0.573	4	1.2	1.6	5	N50.38570	E13.92320	181	CZE
	U3364	subsp. *dioica*	PK	0.575	4	1.3	1.9	5	N50.38570	E13.92320	181	CZE
UP0990	U3365	subsp. *dioica*	PK	0.573	4	1.2	1.7	4	N50.41556	E14.13972	162	CZE
	U3366	subsp. *dioica*	PK	0.572	4	1.2	1.9	5	N50.41556	E14.13972	162	CZE
	U3367	subsp. *dioica*	PK	0.566	4	1.1	1.6	5	N50.41556	E14.13972	162	CZE
	U3368	subsp. *dioica*	PK	0.582	4	1.1	1.9	5	N50.41556	E14.13972	162	CZE
UP0991	U3369	subsp. *dioica*	TF	0.566	4	1.2	1.6	5	N49.07276	E18.58640	481	SVK
UP0992	U3370	subsp. *dioica*	LR	0.567	4	0.7	1.0	2	N53.48355	W2.23586	51	GBR
UP0993	U3371	subsp. *dioica*	GF	0.568	4	0.7	1.1	5	N50.55199	E16.34890	385	CZE
	U3372	subsp. *dioica*	GF	0.573	4	0.7	1.2	4	N50.55199	E16.34890	385	CZE
UP0994	U3376	subsp. *dioica*	TU	0.570	4	1.2	2.1	5	N50.43512	E13.75956	435	CZE
UP0995	U3377	subsp. *dioica*	TU	0.566	4	1.4	1.4	7	N50.55644	E13.92886	749	CZE
UP0996	U3378	subsp. *dioica*	LR	0.569	4	1.0	1.6	5	N50.12020	E16.50322	459	CZE
UP0997	U3379	subsp. *dioica*	RB	0.556	4	1.4	1.6	1	N49.10410	E16.18660	263	CZE
	U3380	subsp. *dioica*	RB	0.547	4	1.5	1.8	1	N49.10410	E16.18660	263	CZE
	U3381	subsp. *dioica*	RB	0.564	4	1.6	1.4	1	N49.10410	E16.18660	263	CZE
	U3382	subsp. *dioica*	RB	0.564	4	1.7	1.5	1	N49.10410	E16.18660	263	CZE

ID number of population	ID number of analysis	Taxon	Collector	Relative fluorescence intensi	DNA-ploidy level	CV of standard	CV of sample	N. of individuals in analysis	Latitude (WGS-84)	Longitude (WGS-84)	Altitude (m a.s.l.)	Country (ISO 3166-1)
UP0998	U3383	subsp. *dioica*	RB	0.571	4	0.9	1.6	5	N47.34762	E13.30571	943	AUT
UP0999	U3384	subsp. *dioica*	LR	0.556	4	0.7	1.3	7	N49.01778	E16.64293	183	CZE
UP1000	U3385	subsp. *dioica*	LR	0.564	4	0.7	1.2	5	N48.78045	E16.70784	174	CZE
UP1001	U3386	subsp. *dioica*	LR	0.566	4	1.0	1.1	6	N48.81729	E16.77653	170	CZE
UP1002	U3387	subsp. *dioica*	LR	0.559	4	0.9	1.2	4	N48.81977	E16.78977	170	CZE
UP1003	U3388	subsp. *dioica*	LR	0.562	4	0.9	1.7	5	N48.81700	E16.78469	169	CZE
UP1004	U3389	subsp. *dioica*	LR	0.564	4	0.7	1.1	9	N48.76555	E16.85404	163	CZE
UP1005	U3390	subsp. *dioica*	LR	0.564	4	1.1	1.3	8	N48.76760	E16.86153	162	CZE
UP1006	U3391	subsp. *dioica*	LR	0.567	4	0.9	1.3	4	N48.80408	E17.09122	165	CZE
UP1007	U3392	subsp. *dioica*	LR	0.564	4	0.7	1.4	7	N48.93260	E17.28843	172	CZE
UP1008	U3393	subsp. *dioica*	LR	0.577	4	0.9	1.6	8	N48.89038	E15.91409	408	CZE
UP1009	U3394	subsp. *dioica*	LR	0.563	4	0.6	1.3	5	N49.08611	E16.72972	194	CZE
UP1010	U3395	subsp. *dioica*	LR	0.565	4	0.9	1.2	9	N48.84469	E16.68961	216	CZE
UP1011	U3400	subsp. *dioica*	FK	0.564	4	1.1	1.6	14	N52.61583	E1.24379	8	GBR
UP1012	U3401	subsp. *dioica*	FK	0.564	4	1.0	1.4	5	N50.48294	E15.50471	470	CZE
UP1013	U3402	subsp. *dioica*	TU	0.565	4	0.9	1.9	5	N50.72511	E15.21677	624	CZE
UP1014	U3403	subsp. *dioica*	LR	0.575	4	1.2	1.6	5	N49.62328	E17.90933	265	CZE
UP1015	U3404	subsp. *dioica*	LR	0.570	4	1.2	1.6	5	N49.66356	E17.99678	242	CZE
UP1016	U3405	subsp. *dioica*	LR	0.562	4	1.9	1.6	5	N49.67956	E18.02869	248	CZE
UP1017	U3406	subsp. *dioica*	LR	0.565	4	1.0	1.5	5	N49.67633	E18.02906	245	CZE
UP1018	U3407	subsp. *dioica*	LR	0.571	4	1.1	2.0	5	N49.71700	E18.12050	230	CZE
UP1019	U3408	subsp. *dioica*	LR	0.562	4	1.5	1.8	5	N49.71689	E18.12103	231	CZE
UP1020	U3409	subsp. *dioica*	LR	0.564	4	1.3	1.7	5	N49.71675	E18.12119	232	CZE

ID number of population	ID number of analysis	Taxon	Collector	Relative fluorescence intensi	DNA-ploidy level	CV of standard	CV of sample	N. of individuals in analysis	Latitude (WGS-84)	Longitude (WGS-84)	Altitude (m a.s.l.)	Country (ISO 3166-1)
UP1021	U3410	subsp. *dioica*	LR	0.567	4	1.0	1.5	5	N49.62336	E17.90931	265	CZE
UP1022	U3411	subsp. *dioica*	LR	0.567	4	1.4	1.7	5	N49.62331	E17.90956	266	CZE
UP1023	U3412	subsp. *dioica*	LR	0.577	4	1.0	1.5	5	N49.62422	E17.94311	258	CZE
UP1024	U3413	subsp. *dioica*	LR	0.570	4	1.3	1.5	5	N49.62442	E17.94272	256	CZE
UP1025	U3414	subsp. *dioica*	LR	0.573	4	1.3	1.7	5	N49.62450	E17.94217	257	CZE
UP1026	U3415	subsp. *dioica*	LR	0.570	4	1.4	1.8	5	N49.62425	E17.94108	255	CZE
UP1027	U3416	subsp. *dioica*	LR	0.571	4	1.0	1.6	5	N49.64017	E17.95661	253	CZE
UP1028	U3417	subsp. *dioica*	LR	0.566	4	1.5	1.9	5	N49.64036	E17.95769	258	CZE
UP1029	U3418	subsp. *dioica*	TU	0.560	4	1.3	1.7	5	N49.66181	E17.19853	225	CZE
	U3419	subsp. *dioica*	TU	0.565	4	1.3	1.8	5	N49.66181	E17.19853	225	CZE
UP1030	U3420	subsp. *dioica*	LR	0.573	4	1.3	1.5	7	N49.25802	E19.35884	502	SVK
UP1031	U3421	subsp. *dioica*	LR	0.576	4	0.9	1.5	4	N49.26060	E19.66255	869	SVK
UP1032	U3422	subsp. *dioica*	SM	0.565	4	1.5	1.7	5	N49.41103	E19.50434	597	SVK
	U3423	subsp. *dioica*	SM	0.575	4	1.6	1.4	5	N49.41103	E19.50434	597	SVK
UP1033	U3424	subsp. *dioica*	LR	0.588	4	0.8	1.4	3	N48.63086	E14.20893	725	CZE
UP1034	U3425	subsp. *dioica*	FK	0.571	4	1.3	2.0	5	N51.74460	W1.24750	58	GBR
UP1035	U3426	subsp. *dioica*	RB	0.570	4	1.7	1.5	6	N48.97592	E15.48048	504	CZE
UP1036	U3427	subsp. *dioica*	RB	0.570	4	1.2	1.7	5	N48.98448	E15.48140	589	CZE
UP1037	U3428	subsp. *dioica*	RB	0.574	4	1.3	1.7	5	N48.98645	E15.46177	493	CZE
UP1038	U3429	subsp. *dioica*	JC	0.568	4	1.4	2.5	1	N40.92873	E24.08205	1578	GRC
UP1039	U3431	subsp. *dioica*	LR	0.567	4	1.3	1.6	5	N50.38588	E13.10847	439	CZE
UP1040	U3432	subsp. *dioica*	RB	0.573	4	1.0	1.7	5	N49.87694	E14.43689	211	CZE
UP1041	U3433	subsp. *dioica*	FK	0.557	4	1.4	1.5	5	N49.41173	E20.44884	460	POL

ID number of population	ID number of analysis	Taxon	Collector	Relative fluorescence intensi	DNA-ploidy level	CV of standard	CV of sample	N. of individuals in analysis	Latitude (WGS-84)	Longitude (WGS-84)	Altitude (m a.s.l.)	Country (ISO 3166-1)
UP1042	U3434	subsp. *dioica*	LR	0.572	4	1.5	1.9	4	N49.38320	E20.45695	312	SVK
UP1043	U3435	subsp. *dioica*	LR	0.565	4	1.5	1.8	6	N49.41665	E20.44759	485	POL
UP1044	U3436	subsp. *dioica*	LR	0.562	4	1.4	1.5	8	N49.40072	E20.42767	485	POL
UP1045	U3437	subsp. *dioica*	TU	0.566	4	1.2	1.4	4	N49.41481	E20.41742	903	POL
UP1046	U3438	subsp. *dioica*	RB	0.572	4	1.1	1.8	5	N49.87784	E14.42312	220	CZE
UP1047	U3439	subsp. *dioica*	JC	0.569	4	1.0	1.2	1	N48.54874	E20.42373	247	SVK
	U3440	subsp. *dioica*	JC	0.574	4	0.9	1.2	1	N48.54874	E20.42373	247	SVK
UP1048	U3441	subsp. *dioica*	TU	0.567	4	1.6	1.7	5	N50.63117	E14.71847	271	CZE
	U3442	subsp. *dioica*	TU	0.577	4	1.1	1.7	5	N50.63117	E14.71847	271	CZE
UP1049	U3443	subsp. *dioica*	RB	0.566	4	1.5	2.9	4	N49.74139	E13.40778	315	CZE
UP1050	U3444	subsp. *dioica*	PV, TU	0.547	4	0.9	1.4	3	N52.31524	E13.49545	39	DEU
UP1051	U3445	subsp. *dioica*	FK	0.574	4	1.5	1.5	2	N49.20029	E19.72378	2111	SVK
UP1052	U3446	subsp. *dioica*	VC	0.565	4	1.2	1.8	1	N49.67824	E13.97619	512	CZE
UP1053	U3447	subsp. *dioica*	ML	0.564	4	0.8	1.1	3	N49.36828	E16.71380	330	CZE
UP1054	U3448	subsp. *dioica*	JC	0.576	4	1.2	1.5	1	N48.77653	E20.33808	967	SVK
UP1055	U3449	subsp. *dioica*	HC	0.572	4	0.9	1.1	5	N47.69643	E14.10802	630	AUT
UP1056	U3450	subsp. *dioica*	HC	0.567	4	1.0	1.3	3	N47.70370	E14.08340	1372	AUT
UP1057	U3451	subsp. *dioica*	HC	0.574	4	0.9	1.1	6	N47.69972	E14.08743	1214	AUT
UP1058	U3452	subsp. *subinermis*	FK	0.305	2	1.5	2.3	5	N48.67792	E16.94604	166	CZE
UP1059	U3453	subsp. *dioica*	FK	0.572	4	1.8	1.9	5	N49.42659	E16.36681	355	CZE
UP1060	U3454	subsp. *dioica*	LR	0.560	4	0.8	1.2	7	N49.50856	E13.99431	503	CZE
UP1061	U3455	subsp. *dioica*	FK	0.577	4	1.3	1.5	5	N48.73150	E16.84789	179	CZE
UP1062	U3456	subsp. *dioica*	RB	0.572	4	1.0	1.4	5	N49.51019	E14.65849	518	CZE

ID number of population	ID number of analysis	Taxon	Collector	Relative fluorescence intensi	DNA-ploidy level	CV of standard	CV of sample	N. of individuals in analysis	Latitude (WGS-84)	Longitude (WGS-84)	Altitude (m a.s.l.)	Country (ISO 3166-1)
UP1063	U3457	subsp. *dioica*	RB	0.584	4	1.1	1.4	5	N49.88020	E14.25263	563	CZE
UP1064	U3458	subsp. *dioica*	RB	0.580	4	1.0	1.5	5	N49.91337	E14.29908	431	CZE
UP1065	U3459	subsp. *dioica*	RB	0.564	4	0.7	1.4	4	N50.04606	E14.37841	318	CZE
UP1066	U3460	subsp. *dioica*	RB	0.574	4	1.1	1.5	5	N49.96983	E14.12995	252	CZE
UP1067	U3461	subsp. *dioica*	RB	0.570	4	0.7	1.4	5	N49.94670	E14.15664	301	CZE
UP1068	U3462	subsp. *dioica*	RB	0.579	4	1.2	1.4	3	N49.94636	E14.16995	365	CZE
UP1069	U3463	subsp. *dioica*	RB	0.564	4	1.3	1.4	5	N49.94769	E14.18222	308	CZE
UP1070	U3464	subsp. *dioica*	RB	0.556	4	1.4	2.0	5	N50.78383	E15.17549	745	CZE
	U3465	subsp. *dioica*	RB	0.575	4	1.5	1.7	6	N50.78383	E15.17549	745	CZE
UP1071	U3466	subsp. *dioica*	RB	0.573	4	1.0	1.8	5	N49.78898	E13.88350	461	CZE
UP1072	U3467	subsp. *dioica*	JPT	0.572	4	0.8	0.9	6	N50.03202	E13.86680	262	CZE
	U3468	subsp. *dioica*	JPT	0.569	4	0.8	1.3	7	N50.03202	E13.86680	262	CZE
UP1073	U3469	subsp. *dioica*	TU	0.565	4	1.0	1.7	5	N50.17132	E14.37658	173	CZE
UP1074	U3470	subsp. *dioica*	HP	0.560	4	0.8	1.1	7	N50.30864	E15.18272	199	CZE
UP1075	U3471	subsp. *dioica*	TU	0.565	4	1.1	1.3	5	N50.13796	E17.20993	631	CZE
	U3472	subsp. *dioica*	TU	0.563	4	0.8	1.2	5	N50.13796	E17.20993	631	CZE
UP1076	U3473	subsp. *dioica*	TU	0.563	4	1.9	1.4	3	N50.11451	E17.24338	938	CZE
UP1077	U3474	subsp. *dioica*	TU	0.557	4	0.9	1.3	6	N50.08613	E17.28470	1009	CZE
UP1078	U3475	subsp. *dioica*	TU	0.572	4	1.2	1.9	2	N50.94769	E13.70072	402	DEU
UP1079	U3486	subsp. *dioica*	FK	0.575	4	1.7	1.7	1	N48.16494	E24.27932	537	UKR
UP1080	U3488	subsp. *dioica*	LR	0.568	4	1.9	1.7	3	N48.43192	E21.80889	96	SVK
UP1081	U3490	subsp. *dioica*	LR	0.570	4	1.5	2.2	1	N48.47369	E22.10936	101	SVK
UP1082	U3494	subsp. *dioica*	LR	0.568	4	1.1	2.6	2	N48.50175	E22.04572	99	SVK

ID number of population	ID number of analysis	Taxon	Collector	Relative fluorescence intensi	DNA-ploidy level	CV of standard	CV of sample	N. of individuals in analysis	Latitude (WGS-84)	Longitude (WGS-84)	Altitude (m a.s.l.)	Country (ISO 3166-1)
UP1083	U3496	subsp. *dioica*	LR	0.573	4	1.5	2.5	6	N48.48619	E22.03809	97	SVK
UP1084	U3497	subsp. *dioica*	LR	0.578	4	1.5	2.2	2	N48.48737	E21.87638	97	SVK
UP1085	U3498	subsp. *dioica*	LR	0.571	4	1.4	1.9	6	N48.34578	E21.83408	94	HUN
UP1086	U3500	subsp. *dioica*	TF	0.574	4	1.7	1.4	1	N57.53519	W6.19606	186	GBR
UP1087	U3501	subsp. *dioica*	TU	0.568	4	1.3	1.5	5	N50.73474	E14.58078	297	CZE
UP1088	U3502	subsp. *dioica*	FK	0.573	4	1.0	1.8	5	N52.28069	E14.69829	37	POL
UP1089	U3503	subsp. *dioica*	TU	0.581	4	1.1	1.7	1	N50.30324	E14.47972	165	CZE
UP1090	U3504	subsp. *dioica*	RB	0.565	4	1.3	1.6	5	N48.86092	E16.64485	343	CZE
UP1091	U3505	subsp. *dioica*	VC	0.562	4	1.7	1.3	1	N49.99489	E14.39894	191	CZE
UP1092	U3506	subsp. *subinermis*	LR	0.300	2	1.6	2.8	1	N48.64465	E16.93118	150	CZE
UP1093	U3507	subsp. *subinermis*	LR	0.297	2	1.1	2.5	1	N48.64827	E16.93423	160	CZE
	U3508	subsp. *dioica*	LR	0.573	4	1.2	1.7	1	N48.64827	E16.93423	160	CZE
UP1094	U3509	subsp. *subinermis*	LR	0.300	2	1.3	2.0	1	N48.64750	E16.93443	158	CZE
UP1095	U3510	subsp. *subinermis*	LR	0.301	2	1.5	2.7	1	N48.65507	E16.94273	158	CZE
UP1096	U3511	subsp. *dioica*	LR	0.574	4	1.4	1.7	1	N48.65342	E16.92287	150	CZE
	U3512	subsp. *subinermis*	LR	0.297	2	1.2	2.3	1	N48.65342	E16.92287	150	CZE
UP1097	U3513	subsp. *dioica*	JPR	0.567	4	2.3	1.7	5	N42.27803	W2.96736	1480	ESP
UP1098	U3514	subsp. *dioica*	FK	0.562	4	1.3	1.8	5	N43.63925	E21.89619	330	SRB
UP1099	U3515	subsp. *dioica*	FK	0.569	4	0.8	1.3	5	N43.16094	E22.13836	542	SRB
UP1100	U3516	subsp. *dioica*	MDU	0.575	4	1.3	1.1	8	N50.31558	E15.49397	244	CZE
	U3517	subsp. *dioica*	MDU	0.574	4	0.7	1.2	9	N50.31558	E15.49397	244	CZE
	U3518	subsp. *dioica*	MDU	0.577	4	0.6	0.9	9	N50.31558	E15.49397	244	CZE
UP1101	U3519	subsp. *dioica*	TU	0.564	4	1.2	1.6	5	N50.59135	E14.63483	270	CZE

ID number of population	ID number of analysis	Taxon	Collector	Relative fluorescence intensi	DNA-ploidy level	CV of standard	CV of sample	N. of individuals in analysis	Latitude (WGS-84)	Longitude (WGS-84)	Altitude (m a.s.l.)	Country (ISO 3166-1)
UP1102	U3520	subsp. *dioica*	TF	0.564	4	1.5	2.8	5	N49.41424	E21.24733	501	SVK
UP1103	U3521	subsp. *dioica*	FK	0.568	4	1.4	1.9	6	N49.82975	E14.44353	242	CZE
UP1104	U3522	subsp. *dioica*	FK	0.578	4	0.9	1.2	1	N48.96031	E20.38328	597	SVK
UP1105	U3523	subsp. *dioica*	GF	0.566	4	1.3	1.6	1	N48.90635	E14.88913	466	CZE
UP1106	U3524	subsp. *dioica*	PK	0.575	4	1.2	1.6	5	N50.38917	E13.98833	180	CZE
	U3525	subsp. *dioica*	PK	0.575	4	1.2	2.0	5	N50.38917	E13.98833	180	CZE
	U3526	subsp. *dioica*	PK	0.578	4	1.6	1.7	5	N50.38917	E13.98833	180	CZE
	U3527	subsp. *dioica*	PK	0.579	4	1.4	1.5	5	N50.38917	E13.98833	180	CZE
UP1107	U3528	subsp. *dioica*	LR	0.564	4	0.7	0.9	4	N48.65237	E14.45241	631	CZE
UP1108	U3529	subsp. *dioica*	FK	0.568	4	1.3	1.5	5	N47.60378	E18.41935	410	HUN
UP1109	U3530	subsp. *dioica*	FK	0.573	4	0.8	1.3	1	N49.25778	E19.70078	1068	SVK
UP1110	U3531	subsp. *dioica*	LR	0.568	4	1.2	1.3	3	N49.29034	E19.07965	633	SVK
UP1111	U3532	subsp. *dioica*	TU	0.581	4	1.6	1.7	5	N50.12241	E14.39781	176	CZE
UP1112	U3533	subsp. *dioica*	TU	0.569	4	1.3	1.9	1	N50.71397	W1.74882	15	GBR
UP1113	U3534	subsp. *dioica*	TU	0.549	4	1.1	1.3	1	N50.32944	E13.08614	500	CZE
UP1114	U3537	subsp. *dioica*	LR	0.562	4	0.9	1.7	8	N50.30595	E16.40378	1090	CZE
UP1115	U3538	subsp. *dioica*	LR	0.573	4	1.2	1.4	6	N50.30282	E16.40007	1105	CZE
UP1116	U3539	subsp. *dioica*	LR	0.566	4	1.0	1.3	3	N50.19017	E16.42113	570	CZE
UP1117	U3540	subsp. *dioica*	LR	0.568	4	1.5	1.6	5	N50.15467	E16.81568	734	CZE
UP1118	U3541	subsp. *dioica*	TU	0.565	4	1.7	1.4	5	N50.51662	E15.23110	441	CZE
UP1119	U3542	subsp. *subinermis*	TU	0.301	2	1.5	1.9	5	N48.75494	E17.01278	162	CZE
	U3741	subsp. *subinermis*	TU	0.308	2	0.7	1.7	1	N48.75494	E17.01278	180	CZE
	U3742	subsp. *subinermis*	TU	0.311	2	1.0	2.0	1	N48.75494	E17.01278	180	CZE

ID number of population	ID number of analysis	Taxon	Collector	Relative fluorescence intensi	DNA-ploidy level	CV of standard	CV of sample	N. of individuals in analysis	Latitude (WGS-84)	Longitude (WGS-84)	Altitude (m a.s.l.)	Country (ISO 3166-1)
	U3743	subsp. *subinermis*	TU	0.300	2	0.9	1.9	1	N48.75494	E17.01278	180	CZE
	U3744	subsp. *subinermis*	TU	0.304	2	0.9	1.8	1	N48.75494	E17.01278	180	CZE
	U3745	subsp. *subinermis*	TU	0.306	2	0.8	2.3	1	N48.75494	E17.01278	180	CZE
	U3746	subsp. *subinermis*	TU	0.301	2	1.0	2.0	1	N48.75494	E17.01278	180	CZE
	U3747	subsp. *subinermis*	TU	0.299	2	1.1	3.1	1	N48.75494	E17.01278	180	CZE
	U3748	subsp. *subinermis*	TU	0.302	2	0.8	1.9	1	N48.75494	E17.01278	180	CZE
UP1120	U3543	subsp. *dioica*	LR	0.562	4	1.6	1.9	5	N49.17228	E17.49214	305	CZE
UP1121	U3544	subsp. *dioica*	LR	0.547	4	1.2	1.8	5	N49.22339	E17.49944	189	CZE
UP1122	U3545	subsp. *dioica*	LR	0.549	4	1.3	2.4	5	N49.37406	E17.68289	495	CZE
UP1123	U3546	subsp. *dioica*	LR	0.553	4	1.5	1.6	5	N49.46386	E17.47283	219	CZE
UP1124	U3547	subsp. *dioica*	LR	0.559	4	0.9	1.9	5	N49.46364	E17.47928	210	CZE
UP1125	U3548	subsp. *dioica*	LR	0.539	4	1.4	1.8	5	N49.52522	E17.32967	235	CZE
UP1126	U3549	subsp. *dioica*	LR	0.544	4	1.5	1.8	5	N49.65614	E17.21794	221	CZE
UP1127	U3550	subsp. *dioica*	LR	0.536	4	1.3	2.0	5	N49.71458	E17.02497	249	CZE
UP1128	U3551	subsp. *dioica*	LR	0.565	4	1.5	1.6	1	N49.87303	E14.86442	394	CZE
UP1129	U3552	subsp. *dioica*	LR	0.563	4	1.6	1.9	1	N49.87133	E14.86717	422	CZE
UP1130	U3553	subsp. *subinermis*	LR	0.294	2	1.2	2.3	1	N48.81786	E16.79511	165	CZE
	U3554	subsp. *subinermis*	LR	0.290	2	1.4	2.2	1	N48.81786	E16.79511	165	CZE
	U3583	subsp. *subinermis*	LR	0.293	2	1.7	2.3	1	N48.81786	E16.79511	165	CZE
	U3584	subsp. *subinermis*	LR	0.292	2	1.8	2.2	1	N48.81786	E16.79511	165	CZE
	U3585	subsp. *subinermis*	LR	0.294	2	1.4	1.8	1	N48.81786	E16.79511	165	CZE
	U3587	subsp. *subinermis*	LR	0.297	2	1.1	2.0	1	N48.81786	E16.79511	165	CZE
	U3589	subsp. *subinermis*	LR	0.299	2	1.7	2.2	1	N48.81786	E16.79511	165	CZE

ID number of population	ID number of analysis	Taxon	Collector	Relative fluorescence intensi	DNA-ploidy level	CV of standard	CV of sample	N. of individuals in analysis	Latitude (WGS-84)	Longitude (WGS-84)	Altitude (m a.s.l.)	Country (ISO 3166-1)
UP1131	U3560	subsp. *dioica*	LR	0.552	4	0.8	1.3	5	N48.84814	E16.72608	172	CZE
UP1132	U3562	subsp. *dioica*	LR	0.537	4	1.2	1.7	5	N48.86386	E17.10486	176	CZE
UP1133	U3563	subsp. *dioica*	PT	0.574	4	1.5	1.5	1	N49.80392	E12.63092	547	CZE
	U3564	subsp. *dioica*	PT	0.583	4	1.0	1.5	1	N49.80392	E12.63092	547	CZE
	U3565	subsp. *dioica*	PT	0.580	4	0.9	1.7	1	N49.80392	E12.63092	547	CZE
	U3566	subsp. *dioica*	PT	0.583	4	1.0	1.5	1	N49.80392	E12.63092	547	CZE
UP1134	U3567	subsp. *dioica*	RB	0.570	4	1.2	1.6	3	N49.86995	E14.45417	239	CZE
UP1135	U3568	subsp. *dioica*	AK	0.562	4	1.0	1.4	1	N50.05727	E17.23674	1308	CZE
UP1136	U3569	subsp. *dioica*	FK	0.566	4	1.5	2.1	5	N49.57099	E15.93968	566	CZE
UP1137	U3572	subsp. *subinermis*	LR	0.247	2	1.4	2.3	1	N48.69164	E16.99350	160	CZE
	U3573	subsp. *subinermis*	LR	0.245	2	1.3	1.8	1	N48.69164	E16.99350	160	CZE
	U3574	subsp. *subinermis*	LR	0.294	2	1.6	2.1	1	N48.69164	E16.99350	160	CZE
	U3575	subsp. *subinermis*	LR	0.302	2	1.4	2.1	1	N48.69164	E16.99350	160	CZE
	U3576	subsp. *subinermis*	LR	0.305	2	2.2	2.3	1	N48.69164	E16.99350	160	CZE
	U3577	subsp. *subinermis*	LR	0.293	2	1.9	2.0	1	N48.69164	E16.99350	160	CZE
UP1138	U3578	subsp. *dioica*	LR	0.548	4	1.2	1.5	5	N49.71961	E17.03003	245	CZE
UP1139	U3579	subsp. *subinermis*	LR	0.293	2	1.8	1.6	1	N48.69203	E16.99444	169	CZE
	U3580	subsp. *subinermis*	LR	0.295	2	1.7	2.3	1	N48.69203	E16.99444	169	CZE
	U3581	subsp. *subinermis*	LR	0.295	2	1.9	1.9	1	N48.69203	E16.99444	169	CZE
	U3582	subsp. *subinermis*	LR	0.300	2	2.8	2.0	1	N48.69203	E16.99444	169	CZE
	U3699	subsp. *subinermis*	TU	0.296	2	1.0	2.2	1	N48.69203	E16.99444	155	CZE
	U3700	subsp. *subinermis*	TU	0.299	2	1.3	1.8	1	N48.69203	E16.99444	155	CZE
	U3701	subsp. *subinermis*	TU	0.303	2	1.4	2.2	1	N48.69203	E16.99444	155	CZE

ID number of population	ID number of analysis	Taxon	Collector	Relative fluorescence intensi	DNA-ploidy level	CV of standard	CV of sample	N. of individuals in analysis	Latitude (WGS-84)	Longitude (WGS-84)	Altitude (m a.s.l.)	Country (ISO 3166-1)
	U3702	subsp. *subinermis*	TU	0.304	2	0.8	2.3	1	N48.69203	E16.99444	155	CZE
	U3703	subsp. *subinermis*	TU	0.301	2	0.8	1.7	1	N48.69203	E16.99444	155	CZE
	U3704	subsp. *subinermis*	TU	0.296	2	0.9	2.1	1	N48.69203	E16.99444	155	CZE
	U3705	subsp. *subinermis*	TU	0.300	2	2.6	1.2	1	N48.69203	E16.99444	155	CZE
	U3706	subsp. *subinermis*	TU	0.296	2	1.4	1.9	1	N48.69203	E16.99444	155	CZE
	U3707	subsp. *subinermis*	TU	0.291	2	0.7	1.9	1	N48.69203	E16.99444	155	CZE
	U3708	subsp. *subinermis*	TU	0.295	2	0.9	1.4	1	N48.69203	E16.99444	155	CZE
	U3709	subsp. *subinermis*	TU	0.297	2	0.9	1.9	1	N48.69203	E16.99444	155	CZE
	U3710	subsp. *subinermis*	TU	0.297	2	0.8	2.3	1	N48.69203	E16.99444	155	CZE
	U3711	subsp. *subinermis*	TU	0.304	2	1.1	2.3	1	N48.69203	E16.99444	155	CZE
	U3712	subsp. *subinermis*	TU	0.298	2	0.9	1.8	1	N48.69203	E16.99444	155	CZE
	U3713	subsp. *subinermis*	TU	0.300	2	0.9	1.8	1	N48.69203	E16.99444	155	CZE
	U3714	subsp. *subinermis*	TU	0.298	2	1.0	2.2	1	N48.69203	E16.99444	155	CZE
	U3715	subsp. *subinermis*	TU	0.295	2	0.9	2.2	1	N48.69203	E16.99444	155	CZE
	U3716	subsp. *subinermis*	TU	0.295	2	0.9	2.2	1	N48.69203	E16.99444	155	CZE
	U3717	subsp. *subinermis*	TU	0.295	2	0.7	2.3	1	N48.69203	E16.99444	155	CZE
	U3718	subsp. *subinermis*	TU	0.310	2	0.7	1.8	1	N48.69203	E16.99444	155	CZE
	U3719	subsp. *subinermis*	TU	0.296	2	0.9	2.0	1	N48.69203	E16.99444	155	CZE
	U3720	subsp. *subinermis*	TU	0.294	2	0.8	2.1	1	N48.69203	E16.99444	155	CZE
	U3721	subsp. *subinermis*	TU	0.302	2	0.7	1.7	1	N48.69203	E16.99444	155	CZE
	U3722	subsp. *subinermis*	TU	0.302	2	0.7	1.9	1	N48.69203	E16.99444	155	CZE
	U3723	subsp. *subinermis*	TU	0.294	2	1.1	1.8	1	N48.69203	E16.99444	155	CZE
UP1140	U3586	subsp. *dioica*	LR	0.559	4	1.4	1.4	1	N48.76867	E20.08392	663	SVK

ID number of population	ID number of analysis	Taxon	Collector	Relative fluorescence intensi	DNA-ploidy level	CV of standard	CV of sample	N. of individuals in analysis	Latitude (WGS-84)	Longitude (WGS-84)	Altitude (m a.s.l.)	Country (ISO 3166-1)
UP1141	U3588	subsp. *dioica*	LR	0.559	4	1.6	1.6	1	N48.70996	E19.97911	684	SVK
UP1142	U3590	subsp. *subinermis*	LR	0.292	2	1.8	2.1	1	N48.84436	E16.72625	174	CZE
	U3591	subsp. *subinermis*	LR	0.290	2	1.1	2.6	1	N48.84436	E16.72625	174	CZE
	U3592	subsp. *subinermis*	LR	0.295	2	1.3	2.4	1	N48.84436	E16.72625	174	CZE
UP1143	U3598	subsp. *dioica*	HP	0.574	4	1.5	1.9	5	N46.49161	E23.36917	1116	ROU
UP1144	U3599	subsp. *dioica*	TU	0.569	4	1.0	1.4	5	N47.19958	E12.60794	1104	AUT
UP1145	U3600	subsp. *dioica*	TU	0.566	4	1.4	1.8	5	N47.25683	E12.56569	816	AUT
UP1146	U3601	subsp. *dioica*	TF	0.578	4	1.3	1.5	1	N60.34993	E6.06243	383	NOR
UP1147	U3602	subsp. *dioica*	TF	0.566	4	1.3	1.5	5	N48.08440	E19.29908	141	SVK
UP1148	U3603	subsp. *dioica*	TF	0.573	4	1.3	1.5	5	N48.22987	E20.03014	217	SVK
UP1149	U3604	subsp. *dioica*	TF	0.573	4	1.0	1.9	5	N48.15012	E18.22183	125	SVK
UP1150	U3605	subsp. *dioica*	HP	0.566	4	1.2	1.5	5	N44.92503	E5.32158	1231	FRA
UP1151	U3606	subsp. *dioica*	HP	0.575	4	1.1	1.7	5	N46.98603	E4.65158	464	FRA
UP1152	U3607	subsp. *dioica*	TF	0.569	4	1.3	1.5	5	N49.00777	E18.99454	538	SVK
UP1153	U3608	subsp. *dioica*	TU	0.571	4	0.7	1.6	5	N50.26283	E13.67439	468	CZE
UP1154	U3609	subsp. *dioica*	TF	0.566	4	1.5	1.3	1	N58.68263	E8.89998	160	NOR
UP1155	U3610	subsp. *dioica*	TU	0.562	4	1.3	2.0	5	N48.79511	E16.84546	177	CZE
UP1156	U3611	subsp. *dioica*	TF	0.557	4	1.4	1.8	5	N48.52903	E18.65988	347	SVK
UP1157	U3612	subsp. *dioica*	TU	0.567	4	1.9	2.6	5	N48.80234	E16.83860	160	CZE
UP1158	U3613	subsp. *dioica*	TF	0.583	4	1.3	1.9	5	N48.19424	E19.53029	165	SVK
UP1159	U3614	subsp. *dioica*	TF	0.566	4	1.1	1.9	5	N48.18147	E20.02580	275	SVK
UP1160	U3615	subsp. *dioica*	TF	0.583	4	1.1	2.5	1	N61.08068	E6.68336	1533	NOR
UP1161	U3616	subsp. *dioica*	TF	0.568	4	0.9	1.4	5	N48.12791	E19.81045	278	HUN

ID number of population	ID number of analysis	Taxon	Collector	Relative fluorescence intensi	DNA-ploidy level	CV of standard	CV of sample	N. of individuals in analysis	Latitude (WGS-84)	Longitude (WGS-84)	Altitude (m a.s.l.)	Country (ISO 3166-1)
UP1162	U3617	subsp. *dioica*	TF	0.559	4	1.4	1.6	5	N52.29428	E13.67026	37	DEU
UP1163	U3618	subsp. *dioica*	TF	0.560	4	1.1	1.3	5	N48.16767	E19.85739	434	SVK
UP1164	U3619	subsp. *dioica*	FK	0.561	4	1.2	1.5	5	N49.36692	E14.51387	376	CZE
UP1165	U3620	subsp. *dioica*	TF	0.565	4	1.2	1.7	1	N61.05617	E6.53475	265	NOR
UP1166	U3621	subsp. *dioica*	FK	0.565	4	0.9	1.3	5	N51.71787	E10.96383	285	DEU
UP1167	U3622	subsp. *dioica*	TU	0.560	4	1.3	1.7	5	N48.80024	E16.83799	163	CZE
UP1168	U3623	subsp. *dioica*	FK	0.573	4	1.1	1.8	5	N41.02797	E20.13103	408	ALB
UP1169	U3624	subsp. *dioica*	FK	0.560	4	0.6	0.9	5	N50.01969	E13.99410	310	CZE
UP1170	U3625	subsp. *dioica*	FK	0.568	4	0.9	1.6	5	N50.28292	E14.51444	168	CZE
UP1171	U3626	subsp. *subinermis*	GF	0.311	2	1.2	2.0	5	N46.27935	E13.83443	527	SVN
UP1172	U3627	subsp. *dioica*	GF	0.576	4	1.0	1.3	5	N44.52922	E15.18808	946	HRV
UP1173	U3631	subsp. *dioica*	LR	0.556	4	1.2	2.2	3	N48.83785	E16.07926	207	CZE
UP1174	U3632	subsp. *dioica*	LR	0.570	4	1.3	1.9	2	N48.82202	E16.09208	250	CZE
UP1175	U3633	subsp. *dioica*	LR	0.580	4	1.5	2.6	3	N50.33100	E15.14342	265	CZE
UP1176	U3634	subsp. *dioica*	LR	0.570	4	1.1	1.6	2	N50.32974	E15.14127	261	CZE
UP1177	U3635	subsp. *dioica*	LR	0.568	4	1.4	1.9	3	N50.34716	E15.12031	254	CZE
UP1178	U3636	subsp. *dioica*	TU	0.564	4	1.2	1.7	5	N47.16406	E12.61414	1847	AUT
UP1179	U3637	subsp. *dioica*	LR	0.593	4	1.6	2.4	2	N48.80670	E16.64669	364	CZE
UP1180	U3639	subsp. *dioica*	HP	0.564	4	1.5	1.9	5	N45.44117	E5.91872	1522	FRA
UP1181	U3640	subsp. *dioica*	HP	0.563	4	1.3	1.6	5	N46.69050	E5.63856	397	FRA
UP1182	U3641	subsp. *dioica*	JR	0.562	4	0.8	1.8	3	N49.17515	E16.22023	367	CZE
UP1183	U3642	subsp. *dioica*	JR	0.571	4	1.2	1.7	3	N49.14487	E16.24577	294	CZE
UP1184	U3643	subsp. *dioica*	JR	0.564	4	0.9	1.2	4	N49.17501	E16.16400	369	CZE

ID number of population	ID number of analysis	Taxon	Collector	Relative fluorescence intensi	DNA-ploidy level	CV of standard	CV of sample	N. of individuals in analysis	Latitude (WGS-84)	Longitude (WGS-84)	Altitude (m a.s.l.)	Country (ISO 3166-1)
UP1185	U3644	subsp. *dioica*	JR	0.573	4	0.7	1.1	1	N49.16998	E16.16611	339	CZE
UP1186	U3645	subsp. *dioica*	JR	0.562	4	0.6	0.9	1	N49.16796	E16.17160	335	CZE
UP1187	U3646	subsp. *dioica*	JR	0.558	4	0.7	1.7	1	N49.15542	E16.16894	310	CZE
UP1188	U3647	subsp. *dioica*	FK	0.570	4	0.9	1.6	5	N46.56625	E23.67342	504	ROU
UP1189	U3648	subsp. *dioica*	LR	0.567	4	1.0	1.5	4	N49.17654	E14.30722	429	CZE
UP1190	U3649	subsp. *dioica*	LR	0.558	4	0.9	1.3	5	N49.17473	E14.30588	430	CZE
UP1191	U3650	subsp. *dioica*	LR	0.571	4	1.5	1.7	6	N49.17421	E14.30265	429	CZE
	U3651	subsp. *dioica*	LR	0.563	4	1.0	1.4	3	N49.17421	E14.30265	429	CZE
UP1192	U3652	subsp. *dioica*	TF	0.570	4	1.1	1.5	5	N49.10220	E19.24182	461	SVK
UP1193	U3653	subsp. *dioica*	TF	0.569	4	1.9	1.4	5	N48.91019	E19.09019	1478	SVK
UP1194	U3654	subsp. *dioica*	FK	0.571	4	0.9	1.1	1	N60.04506	E6.46348	482	NOR
UP1195	U3655	subsp. *dioica*	FK	0.573	4	1.2	2.2	4	N50.02938	E14.56324	256	CZE
UP1196	U3657	subsp. *dioica*	LM	0.565	4	0.7	1.9	3	N48.88197	E19.09267	1178	SVK
UP1197	U3658	subsp. *dioica*	TU	0.576	4	0.8	1.6	5	N48.20125	E17.83014	118	SVK
UP1198	U3659	subsp. *dioica*	TU	0.576	4	1.6	1.3	5	N48.20592	E17.83425	119	SVK
UP1199	U3660	subsp. *dioica*	AK	0.555	4	1.4	1.6	5	N50.74931	E15.54836	1334	CZE
UP1200	U3661	subsp. *subinermis*	TU	0.300	2	1.1	2.3	5	N48.52083	E16.94933	148	SVK
UP1201	U3662	subsp. *dioica*	TU	0.583	4	1.8	2.2	5	N48.77911	E16.79110	182	CZE
UP1202	U3663	subsp. *dioica*	LR	0.571	4	1.7	1.7	5	N49.32120	E14.70193	397	CZE
UP1203	U3664	subsp. *dioica*	LR	0.564	4	1.3	1.6	5	N49.28469	E14.69965	399	CZE
UP1204	U3665	subsp. *dioica*	LR	0.564	4	1.1	1.8	5	N49.23439	E14.71557	403	CZE
UP1205	U3666	subsp. *dioica*	LR	0.569	4	1.1	1.5	5	N49.20391	E14.70973	408	CZE
UP1206	U3667	subsp. *dioica*	LR	0.567	4	1.3	1.7	5	N49.12221	E14.73877	422	CZE

ID number of population	ID number of analysis	Taxon	Collector	Relative fluorescence intensi	DNA-ploidy level	CV of standard	CV of sample	N. of individuals in analysis	Latitude (WGS-84)	Longitude (WGS-84)	Altitude (m a.s.l.)	Country (ISO 3166-1)
UP1207	U3668	subsp. *dioica*	LR	0.577	4	1.2	1.8	5	N49.04412	E14.80038	425	CZE
UP1208	U3669	subsp. *dioica*	RB	0.574	4	1.0	1.3	5	N49.19341	E14.71564	412	CZE
UP1209	U3670	subsp. *dioica*	FK	0.571	4	1.1	1.7	1	N51.80278	E10.30406	484	DEU
UP1210	U3671	subsp. *dioica*	RB	0.570	4	1.3	1.7	3	N50.56705	E13.97290	666	CZE
UP1211	U3672	subsp. *dioica*	FK	0.581	4	1.6	1.4	1	N50.06512	E14.44752	204	CZE
UP1212	U3674	subsp. *dioica* triploid	LR	0.426	3	1.2	1.7	1	N50.17098	E14.86100	190	CZE
UP1213	U3675	subsp. *dioica*	LR	0.563	4	1.7	2.2	1	N50.17027	E14.85982	190	CZE
UP1214	U3676	subsp. *dioica*	TF	0.566	4	1.5	2.4	5	N48.47768	E17.43095	238	SVK
UP1215	U3678	subsp. *dioica*	RB	0.568	4	0.9	1.6	5	N47.33304	E13.29504	842	AUT
UP1216	U3679	subsp. *dioica*	RB	0.570	4	1.1	1.6	5	N56.14470	E10.17401	4	DNK
UP1217	U3680	subsp. *dioica*	RB	0.559	4	0.9	1.7	4	N56.17545	E10.22845	15	DNK
UP1218	U3681	subsp. *dioica*	TU	0.578	4	1.2	2.5	1	N46.23859	E13.81246	1880	SVN
UP1231	U3843	subsp. *dioica*	TU	0.563	4	1.5	2.9	1	N56.60083	E61.05650	207	RUS

Collectors: AK - Adam Knotek, AR - Adéla Rejlová, BS - Bohuna Senius, CP - Clemens Pachschwöll, DH - Dana Hubková, DR - Danijela Rostohar, EZ - Eliška Záveská, FK - Filip Kolář, GF - Gábina Fuxová, HC - Hana Chudáčková, HD - Hana Daneck, HJ - Hana Jirsáková, HP - Hana Přívozníková, IR - Iva Riegerová, JA - Jana Aichlerová, JB - Jana Bajerová, JC - Jindřich Chrtek, JH - Johana Hanzlíčková, JP - Jan Ponert, JPR - Jan Prančl, JPT - Jan Ptáček, JR - Jan Rydlo, JS - Jan Smyčka, JV - Jana Vítová, KH - Kristýna Hanušová, KK - Klára Kabátová, KS - Kristýna Šemberová, KSE - Kari Senius, KSR - Kamila Šrédlová, LM - Lenka Macková, LR - Ludmila Rejlová, MD - Martin Dudáš, MDU - Michal Ducháček, ML - Magdalena Lučanová, OK - Ondřej Kouklík, PK - Pavel Kúr, PT - Pavel Trávníček, PV - Petr Vít, RB - Romana Bartošová, SM - Štefan Murín, SP - Soňa Píšová, SS - Šárka Svobodová, TF - Tomáš Figura, TR - Tamara Rejlová, TU - Tomáš Urfus, VC - Václav Černohorský, VK - Veronika Konečná, VR - Vojtěch Rejl, ZC - Zuzana Chumová, ZK - Zdeněk Kaplan.
