# Supplementary material for: Polyploid evolution: The ultimate way to grasp the nettle
Source: PLoS One. 2019 Jul 1;14(7):e0218389. doi: 10.1371/journal.pone.0218389 (PMC6602185; doi:10.1371/journal.pone.0218389)
Supplement: S2 Table — For each population, the following information is provide: geographic coordinates in the WGS-84 system, elevation, country abbreviation, collector’s initials, absolute genome size—2C-value (pg), ploidy level, and coefficient of variance of standard and sample peaks. (PDF) [file pone.0218389.s002.pdf]

Table S2. List of analysis (absolute genome size) of *Urtica dioica* (sorted by recognized taxa and identification number of population). For each population the information is provided about geography coordinates in WGS-84 system, altitude and abbreviation of country, collectors initials, absolute genome size – 2C-value (pg), ploidy level, coefficient of variance of standard and samples.

ID number of population	ID number of analysis	Taxon	Collector	2C-value (pg)	Ploidy level	CV of standard	CV of sample	Latitude (WGS-84)	Longitude (WGS-84)	Altitude (m a.s.l.)	Country (ISO 3166-1)
UP1219	U3831	subsp. *cypria*	PT, ZC	1.66	3	1.2	1.8	N34.93594	E33.02818	1077	CYP
	U3832	subsp. *cypria*	PT, ZC	1.67	3	1.3	1.9	N34.93594	E33.02818	1077	CYP
	U3833	subsp. *cypria*	PT, ZC	1.66	3	1.2	1.7	N34.93594	E33.02818	1077	CYP
	U3834	subsp. *cypria*	PT, ZC	1.67	3	1.3	2.0	N34.93594	E33.02818	1077	CYP
	U3835	subsp. *cypria*	PT, ZC	1.67	3	1.3	1.8	N34.93594	E33.02818	1077	CYP
	U3836	subsp. *cypria*	PT, ZC	1.67	3	1.3	1.7	N34.93594	E33.02818	1077	CYP
UP0113	U3777	subsp. *dioica*	EZ, FK	2.17	4	1.3	1.7	N54.28356	E16.13995	2	POL
UP0201	U3765	subsp. *dioica*	PT, ZC	2.18	4	1.3	1.7	N37.85794	E13.38822	1130	ITA
UP0202	U3767	subsp. *dioica*	PT, ZC	2.10	4	1.2	2.1	N37.91019	E13.99032	860	ITA
UP0435	U3778	subsp. *dioica*	EZ, FK	2.20	4	2.0	2.5	N69.65605	E18.93484	100	NOR
UP0621	U3772	subsp. *dioica*	PV, TU	2.19	4	1.3	1.6	N51.10167	E17.07150	132	POL
UP0626	U3773	subsp. *dioica*	PV, TU	2.15	4	1.0	1.4	N52.48602	E12.83499	29	DEU
UP0629	U3755	subsp. *dioica*	FK	2.18	4	1.7	2.0	N36.66113	E51.32025	190	IRN
UP0630	U3750	subsp. *dioica*	LR	2.19	4	1.3	1.4	N47.62380	E12.97902	615	DEU
UP0654	U3787	subsp. *dioica*	LR	2.21	4	1.2	1.8	N46.23650	E10.22507	530	ITA
UP0664	U3749	subsp. *dioica*	LR	2.22	4	1.2	1.9	N45.81215	E6.96012	1266	ITA
UP0685	U3751	subsp. *dioica*	LR	2.20	4	1.2	1.8	N45.93151	E6.91746	1916	FRA
UP0725	U3752	subsp. *dioica*	RB, TU	2.27	4	1.5	1.8	N41.19679	E22.77564	146	GRC
UP1220	U3753	subsp. *dioica*	JC, TU	2.13	4	1.5	1.8	N40.70466	E40.65491	1492	TUR

ID number of population	ID number of analysis	Taxon	Collector	2C-value (pg)	Ploidy level	CV of standard	CV of sample	Latitude (WGS-84)	Longitude (WGS-84)	Altitude (m a.s.l.)	Country (ISO 3166-1)
UP1222	U3776	subsp. *dioica*	HC	2.17	4	2.2	2.2	N45.72544	E10.84376	2168	ITA
UP1224	U3770	subsp. *dioica*	FK	2.09	4	1.2	1.4	N47.60377	E18.41934	410	HUN
UP1227	U3764	subsp. *dioica*	FK	2.21	4	1.8	2.9	N48.75449	E16.89148	157	CZE
UP1228	U3771	subsp. *dioica*	PV, TU	2.19	4	1.2	1.4	N51.57042	E13.92891	125	DEU
UP1230	U3763	subsp. *dioica*	LR, SH	2.17	4	1.1	2.0	N55.67540	E13.33463	66	SWE
UP1231	U3754	subsp. *dioica*	TU	2.14	4	1.4	1.9	N56.60083	E61.05650	207	RUS
UP1233	U3762	subsp. *dioica*	LR, SH	2.13	4	1.5	1.4	N59.02181	E14.59265	106	SWE
UP1234	U3756	subsp. *dioica*	LR, SH	2.22	4	1.4	2.0	N59.38068	E27.5539	1	EST
UP1235	U3757	subsp. *dioica*	LR, SH	2.23	4	1.3	1.8	N60.23938	E24.65983	19	FIN
UP1241	U3758	subsp. *dioica*	LR, SH	2.18	4	1.2	1.8	N62.97150	E21.4927	4	FIN
UP1242	U3761	subsp. *dioica*	LR, SH	2.25	4	1.2	1.9	N65.37790	E21.29759	24	SWE
UP1243	U3760	subsp. *dioica*	LR, SH	2.17	4	1.7	2.1	N65.88580	E22.95945	55	SWE
UP1244	U3759	subsp. *dioica*	LR, SH	2.25	4	1.3	1.6	N68.43923	E22.46148	313	SWE
UP1246	U3779	subsp. *dioica*	EZ, FK	2.19	4	1.4	1.8	N69.54531	E19.01271	79	NOR
UP1249	U3775	subsp. *dioica*	PT	2.22	4	1.4	1.9	N55.67545	E12.52854	62	DNK
	U3766	subsp. *dioica*	PT, ZC	2.17	4	1.0	2.1	N37.85794	E13.38822	1130	ITA
	U3768	subsp. *dioica*	PT, ZC	2.17	4	1.2	2.3	N37.91019	E13.99032	860	ITA
	U3769	subsp. *dioica*	PT, ZC	2.14	4	1.2	2.0	N37.91019	E13.99032	860	ITA
	U3774	subsp. *dioica*	FK	2.16	4	1.6	2.2	N36.66114	E51.32025	190	IRN
UP0525	U3784	subsp. *kurdistanica*	JC, TU	1.20	2	1.4	2.7	N38.52032	E35.52553	2196	TUR
UP0527	U3780	subsp. *kurdistanica*	JC, TU	1.18	2	1.3	2.5	N37.35832	E34.69034	1765	TUR
	U3785	subsp. *kurdistanica*	JC, TU	1.16	2	1.7	2.8	N38.52032	E35.52553	2196	TUR
	U3781	subsp. *kurdistanica*	JC, TU	1.19	2	1.3	2.4	N37.35832	E34.69034	1765	TUR
	U3782	subsp. *kurdistanica*	JC, TU	1.19	2	1.2	2.7	N37.35832	E34.69034	1765	TUR

ID number of population	ID number of analysis	Taxon	Collector	2C-value (pg)	Ploidy level	CV of standard	CV of sample	Latitude (WGS-84)	Longitude (WGS-84)	Altitude (m a.s.l.)	Country (ISO 3166-1)
	U3783	subsp. *kurdistanica*	JC, TU	1.16	2	1.4	3.0	N37.35832	E34.69034	1765	TUR
UP0006	U3801	subsp. *pubescens*	PT, TU	1.10	2	1.0	2.0	N46.56411	E11.51614	433	ITA
UP0014	U3802	subsp. *pubescens*	PT, TU	1.10	2	1.0	1.8	N44.76683	E11.85939	1	ITA
UP0656	U3799	subsp. *pubescens*	LR	1.18	2	1.4	3.2	N45.86217	E9.42163	444	ITA
UP0657	U3798	subsp. *pubescens*	LR	1.19	2	1.3	2.8	N45.80548	E9.25597	265	ITA
UP0669	U3797	subsp. *pubescens*	LR	1.21	2	1.3	2.5	N44.37202	E8.07663	465	ITA
UP0673	U3796	subsp. *pubescens*	LR	1.17	2	1.3	2.3	N44.73902	E8.51898	122	ITA
UP0674	U3786	subsp. *pubescens*	LR	1.17	2	1.5	3.0	N45.04970	E8.62982	86	ITA
UP0678	U3794	subsp. *pubescens*	LR	1.15	2	1.4	2.9	N44.88212	E9.61557	342	ITA
UP0679	U3792	subsp. *pubescens*	LR	1.18	2	1.3	2.2	N44.66752	E9.61743	758	ITA
UP0682	U3790	subsp. *pubescens*	LR	1.16	2	1.3	2.5	N44.93050	E10.36508	42	ITA
UP0683	U3789	subsp. *pubescens*	LR	1.21	2	1.2	2.7	N44.90670	E10.51723	14	ITA
UP0684	U3788	subsp. *pubescens*	LR	1.17	2	1.3	2.7	N45.06637	E10.84375	10	ITA
UP1221	U3791	subsp. *pubescens*	LR	1.15	2	1.1	2.8	N44.66751	E9.61743	758	ITA
	U3800	subsp. *pubescens*	LR	1.17	2	1.2	2.5	N45.86217	E9.42163	444	ITA
	U3795	subsp. *pubescens*	LR	1.16	2	1.4	2.4	N45.04970	E8.62982	86	ITA
	U3793	subsp. *pubescens*	LR	1.16	2	1.3	2.7	N44.66752	E9.61743	758	ITA
UP0436	U3804	subsp. *sondenii*	LR, SH	1.15	2	1.7	3.2	N69.67887	E18.89820	1	NOR
UP1245	U3803	subsp. *sondenii*	LR, SH	1.14	2	1.4	2.1	N68.48526	E22.29748	328	FIN
	U3805	subsp. *sondenii*	LR, SH	1.16	2	1.7	3.0	N69.67887	E18.89820	1	NOR
	U3806	subsp. *sondenii*	LR, SH	1.12	2	1.5	2.9	N69.54531	E19.01271	79	NOR
UP0528	U3808	subsp. *subinermis*	TU	1.25	2	1.5	2.7	N37.51418	E34.62849	1208	TUR
UP0529	U3807	subsp. *subinermis*	TU	1.19	2	1.5	2.5	N38.71923	E37.35649	1250	TUR
UP0772	U3815	subsp. *subinermis*	RB, TU	1.12	2	1.0	2.1	N42.80928	E9.48922	7	FRA

ID number of population	ID number of analysis	Taxon	Collector	2C-value (pg)	Ploidy level	CV of standard	CV of sample	Latitude (WGS-84)	Longitude (WGS-84)	Altitude (m a.s.l.)	Country (ISO 3166-1)
UP1223	U3819	subsp. *subinermis*	FK	1.12	2	1.4	2.2	N46.19805	E18.85194	96	HUN
UP1225	U3818	subsp. *subinermis*	FK	1.14	2	1.1	2.4	N48.67931	E16.94617	13	CZE
UP1226	U3817	subsp. *subinermis*	FK	1.10	2	1.1	2.4	N48.69702	E16.95968	154	CZE
UP1232	U3814	subsp. *subinermis*	LR, SH	1.16	2	1.4	2.3	N56.95191	E23.51314	2	LVA
UP1236	U3811	subsp. *subinermis*	LR, SH	1.15	2	1.2	2.4	N61.48451	E29.48244	88	FIN
UP1237	U3812	subsp. *subinermis*	LR, SH	1.18	2	1.5	2.6	N61.84828	E29.17859	129	FIN
UP1238	U3810	subsp. *subinermis*	LR, SH	1.13	2	1.4	2.2	N62.06351	E27.56121	127	FIN
UP1239	U3813	subsp. *subinermis*	LR, SH	1.14	2	1.3	2.2	N62.16090	E28.15618	91	FIN
UP1240	U3809	subsp. *subinermis*	LR, SH	1.16	2	1.3	2.5	N62.90926	E24.82408	150	FIN
	U3816	subsp. *subinermis*	RB, TU	1.11	2	1.2	2.1	N42.80928	E9.48922	7	FRA
	U3820	subsp. *subinermis*	FK	1.15	2	1.1	2.3	N46.19805	E18.85194	96	HUN
	U3821	subsp. *subinermis*	FK	1.17	2	1.5	2.4	N46.19805	E18.85194	96	HUN
	U3822	subsp. *subinermis*	FK	1.14	2	1.8	2.9	N46.19805	E18.85194	96	HUN
	U3823	subsp. *subinermis*	FK	1.14	2	1.1	2.4	N46.19805	E18.85194	96	HUN
	U3824	subsp. *subinermis*	FK	1.16	2	1.1	2.4	N46.19805	E18.85194	96	HUN
	U3825	subsp. *subinermis*	FK	1.14	2	1.0	2.3	N46.19805	E18.85194	96	HUN
UP0774	U3826	*U. atrovirens*	RB, TU	1.19	2	1.2	2.3	N42.30477	E9.15027	460	FRA
UP0776	U3828	*U. atrovirens*	RB, TU	1.18	2	1.4	2.2	N42.37303	E9.14494	440	FRA
UP0777	U3829	*U. atrovirens*	RB, TU	1.19	2	1.1	2.3	N42.24014	E8.84380	790	FRA
	U3827	*U. atrovirens*	RB, TU	1.19	2	1.2	2.0	N42.30477	E9.15027	460	FRA
UP1250	U3830	*U. bianorii*	ML	1.65	2	1.5	2.4	Lučanová unpubl.	Lučanová unpubl.	–	ESP
UP0688	U3837	*U. kioviensis*	LR	1.41	2	1.3	2.4	N48.24330	E17.25355	134	SVK
UP1229	U3840	*U. kioviensis*	PV, TU	1.43	2	1.1	2.0	N52.56075	E14.67413	13	POL
	U3838	*U. kioviensis*	LR	1.43	2	1.4	2.4	N48.24330	E17.25355	134	SVK

ID number of population	ID number of analysis	Taxon	Collector	2C-value (pg)	Ploidy level	CV of standard	CV of sample	Latitude (WGS-84)	Longitude (WGS-84)	Altitude (m a.s.l.)	Country (ISO 3166-1)
	U3839	*U. kioviensis*	LR	1.41	2	1.2	2.2	N48.24330	E17.25355	134	SVK
	U3841	*U. kioviensis*	FK	1.36	2	1.0	2.3	N48.67931	E16.94617	13	CZE
UP1248	U3842	*U. simensis*	FK	1.48	2	3.4	3.5	N7.04800	E39.76110	3332	ETH

Collectors: EZ - Eliška Záveská, FK - Filip Kolář, HC - Hana Chudáčková, JC - Jindřich Chrtek, LR - Ludmila Rejlová, ML - Magdalena Lučanová, PT - Pavel Trávníček, PV - Petr Vít, RB - Romana Bartošová, SH - Šárka Hořčicová, TU - Tomáš Urfus, ZC - Zuzana Chumová.
