# Supplementary figures and images for: Polyploid evolution: The ultimate way to grasp the nettle
Source: PLoS One. 2019 Jul 1;14(7):e0218389. doi: 10.1371/journal.pone.0218389 (PMC6602185; doi:10.1371/journal.pone.0218389)

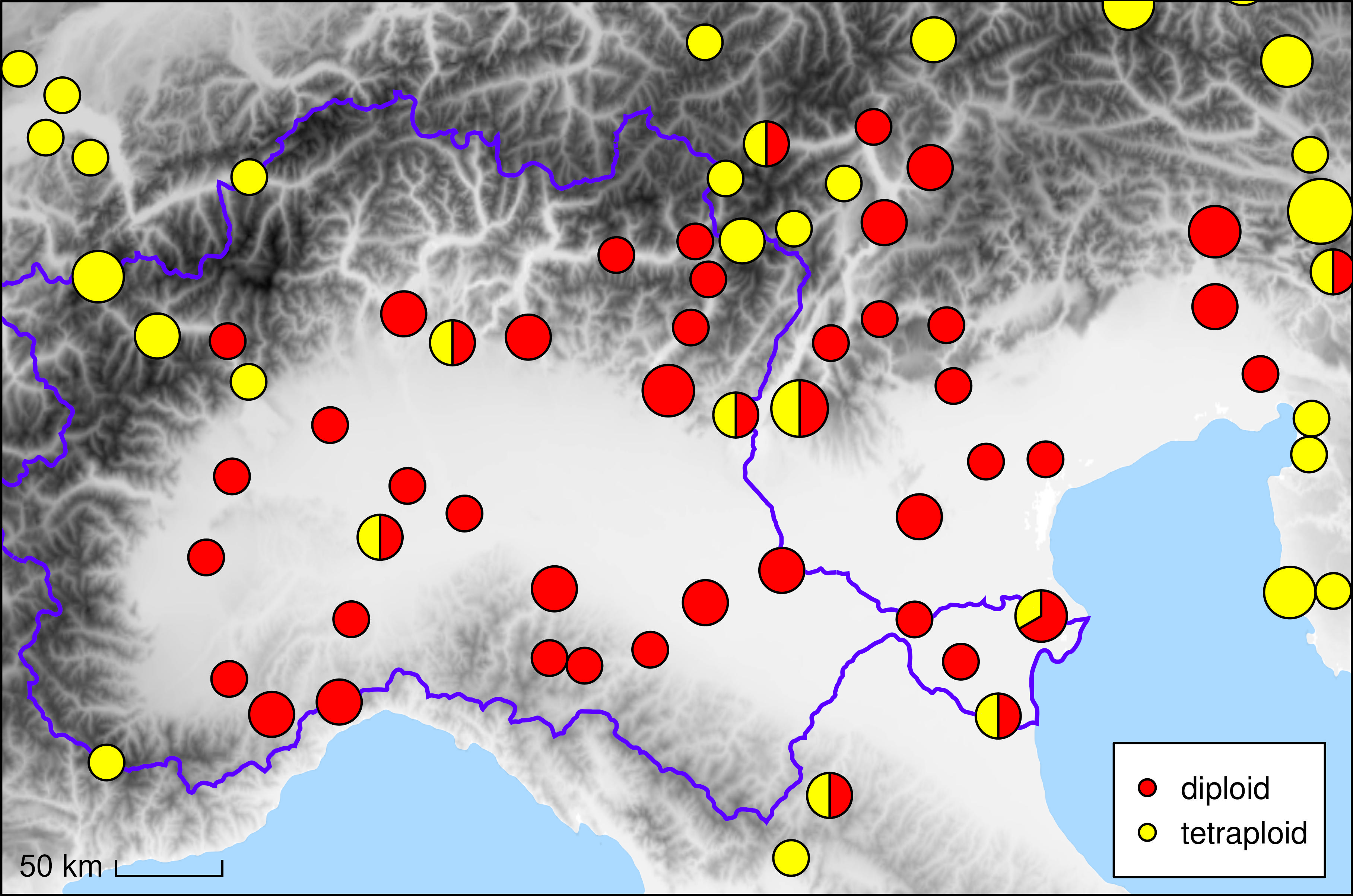

Supplement: S1 Fig — The size of the circles reflects the number of populations. The blue line indicates the outline of the Po river basin. (TIF) [file pone.0218389.s003.tif]

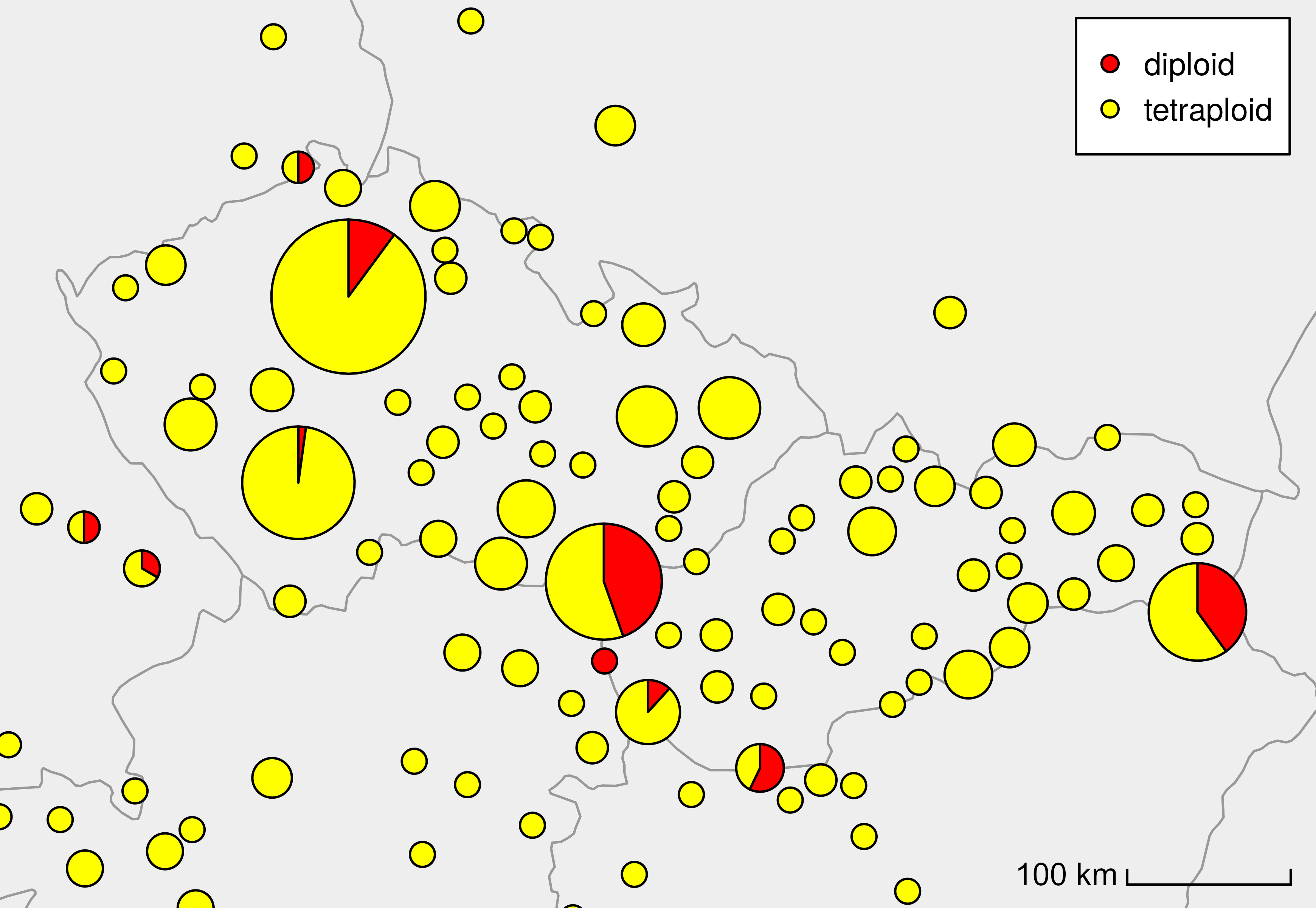

Supplement: S2 Fig — The size of the circles reflects the number of populations. (TIF) [file pone.0218389.s004.tif]

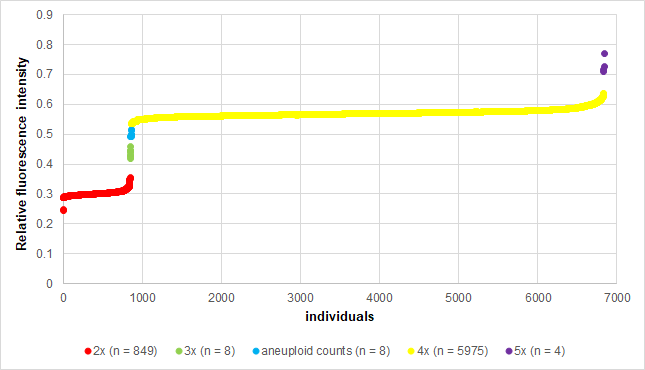

Supplement: S3 Fig — Two dominant ploidy levels were detected (red—2x and yellow—4x). (TIF) [file pone.0218389.s005.tif]

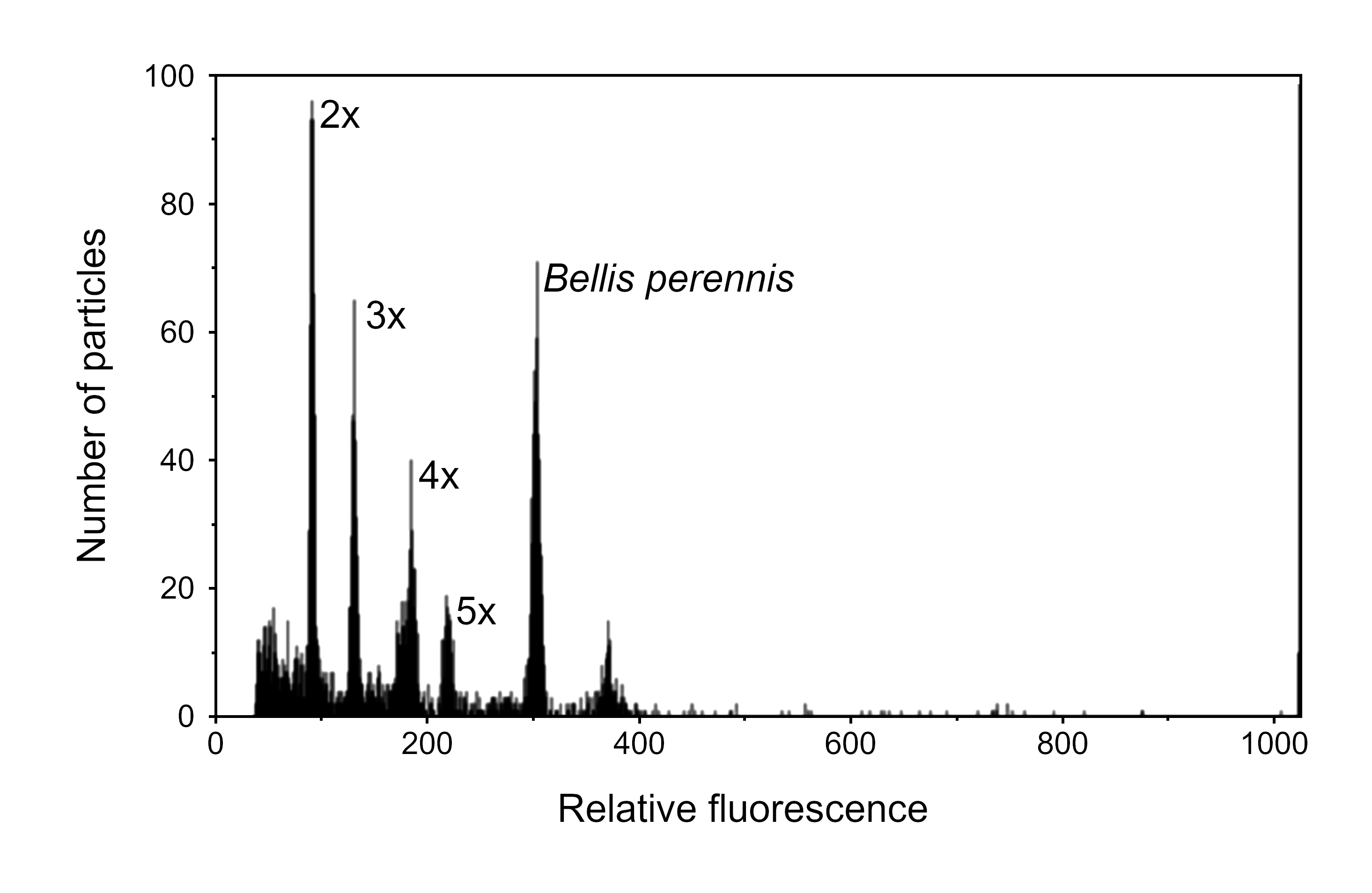

Supplement: S4 Fig — Simultaneous analysis—from the left: 2x—diploid cytotype, 3x—triploid, 4x—tetraploid, 5x—pentaploid, Bellis perennis—the internal standard. (TIF) [file pone.0218389.s006.tif]

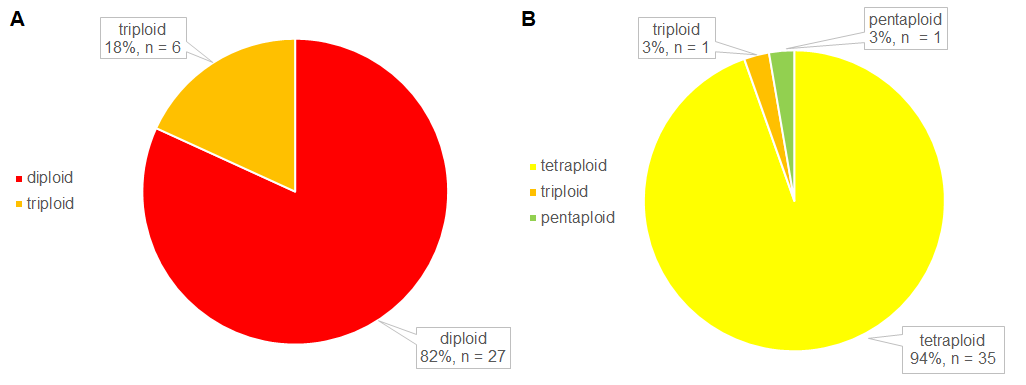

Supplement: S5 Fig — (A) Ratio of diploid and triploid seeds from a 2x maternal plant (from the mixed-ploidy population); (B) Ratio of triploid, tetraploid and pentaploid seeds from a 4x maternal plant (from a mixed-ploidy population). (TIF) [file pone.0218389.s007.tif]

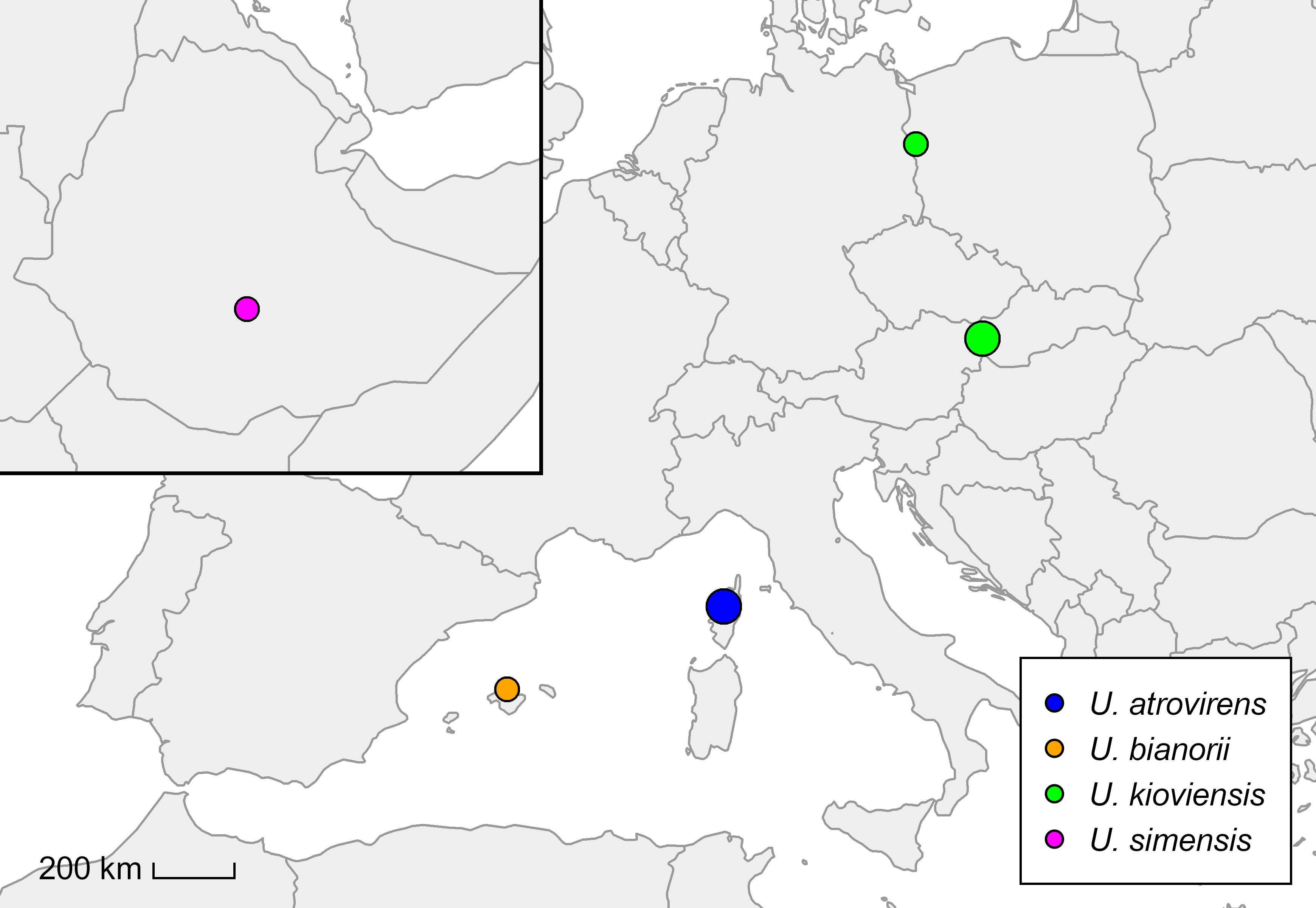

Supplement: S6 Fig — Species closely related to the U. dioica clade in recent phylogenies, namely: U. atrovirens, U. bianorii, U. kioviensis. The top-left section shows the one population of U. simensis in Ethiopia. The size of the circles reflects the number of populations. For more details see S2 Table. (TIF) [file pone.0218389.s008.tif]

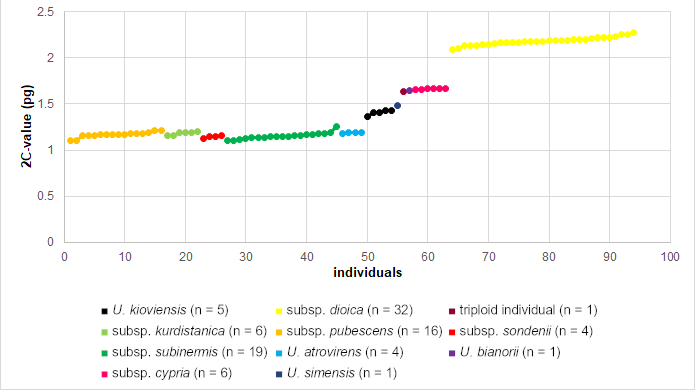

Supplement: S7 Fig — Diploid cytotype—U. d. subsp. kurdistanica, subsp. pubescens, subsp. sondenii and subsp. subinermis; tetraploid cytotype—U. d. subsp. dioica; closely related species—U. atrovirens, U. bianorii, U. d. subsp. cypria, U. kioviensis and U. simensis). Numbers of analysed individuals are presented in parentheses. (TIF) [file pone.0218389.s009.tif]
